# Expanding Steroid Glycodiversity: Tandem Steroid Glucosylation and Acetylation via Enzymatic Cascade

**DOI:** 10.3390/ijms27125232

**Published:** 2026-06-09

**Authors:** Agata Matera, Kinga Dulak, Sandra Sordon, Ewa Huszcza, Tomasz Janeczko, Jarosław Popłoński

**Affiliations:** 1 Department of Food Chemistry and Biocatalysis, Faculty of Biotechnology and Food Science, Wrocław University of Environmental and Life Sciences, C.K. Norwida 25, 50-375 Wrocław, Poland; agata.matera@upwr.edu.pl (A.M.); kinga.dulak@upwr.edu.pl (K.D.); sandra.sordon@upwr.edu.pl (S.S.); ewa.huszcza@upwr.edu.pl (E.H.); tomasz.janeczko@upwr.edu.pl (T.J.); 2 Department of Chemical Biology, Faculty of Biotechnology, University of Wrocław, Joliot-Curie 14a, 50-383 Wrocław, Poland

**Keywords:** regioselectivity, glucosyltransferase, acetyltransferase, epitestosterone, androstanes, estrogens, pregnanes, corticosteroids, sucrose synthase

## Abstract

Steroid glycosides constitute an important class of bioactive molecules, yet their selective synthesis remains challenging. Here, we established a screening platform for nucleotide sugar-dependent glycosyltransferases (GTs) coupled with sucrose synthase (SuSy) for in situ UDP-glucose regeneration, enabling cost-efficient steroid glucosylation. A library of GTs comprising literature-derived enzymes and newly mined archaeal and fungal candidates was constructed using sequence filtering, AlphaFold3 modeling, and docking-guided prioritization. The resulting panel was screened against 31 structurally diverse steroids (androgens, estrogens, pregnanes, and corticosteroids) using crude *Escherichia coli* lysates as catalysts and UPLC-DAD, LC-MS and NMR analytics. YjiC and OleD glycosyltransferases emerged as the most promiscuous biocatalysts, while Sbaic7OGT and SgUGT74AC1_M7 displayed greater selectivity toward estrogens and selected testosterone derivatives. Product assignment for representative reactions was validated using authenticated reference standards or NMR (1D/2D) analysis, confirming regioisomeric estradiol monoglucosides (3-*O*- and 17-*O*-), estrone 3-*O*-glucoside, and an unexpected product diversification for 17α-testosterone by endogenous *E. coli* enzyme, where the major product was identified as a 6′-*O*-acetylated glucoside. Finally, SuSy-coupled cascades were applied in semi-preparative scale and evaluated under optimized conditions and co-immobilization formats.

## 1. Introduction

Steroids represent a large and widely used class of pharmaceuticals. Their structural diversity—including the degree of ring saturation and the type and distribution of functional groups (e.g., hydroxyl, methyl, acetyl, alkyl, glycosyl)—underlies a broad spectrum of biological activities, such as anti-inflammatory [1], immunosuppressive [2], sedative [3], and anabolic effects [4]. Among these, steroid glycosides warrant particular attention. Cardiac glycosides, such as digitoxin, are well established in the treatment of congestive heart failure and cardiac arrhythmias through inhibition of the cellular Na^+^/K^+^-ATPase pump and regulation of intracellular Na^+^ and K^+^ concentrations [5]. More recently, they have been shown to suppress cancer cell proliferation by inducing immunogenic cell death [6], highlighting their potential as anticancer agents [7]. Other classes of steroid glycosides also demonstrate therapeutic promise: androgen glycosides exhibit neuroprotective effects and may enhance cellular ATP production [8], while acetylated testosterone-17-*O*-β-glycoside displays greater cytotoxicity against several human tumor cell lines compared with testosterone [9]. Dexamethasone-β-D-glucoside has been shown to be a potent prodrug for the colonic delivery of the anti-inflammatory agent dexamethasone, with increased chemical and enzymatic stability in the stomach and small intestine [10]. Collectively, steroid glycoconjugates represent a valuable reservoir of potential therapeutics. The development of efficient biosynthetic pathways for these compounds is essential to ensure biosustainable production and facilitate rigorous clinical evaluation.

Enzymatic glycosylation offers an attractive alternative to expensive and time-consuming chemical synthesis methods, which often require complex protection/deprotection manipulations [11], and lack of selectivity, particularly for complex substrates like steroids [12]. Nucleotide sugar-dependent glycosyltransferases (GTs) catalyze the transfer of sugar moieties from nucleotide sugars to nucleophilic glycosyl acceptors, forming glycosidic bonds with regio- and stereoselectivity [13]. The high specificity of glycosyltransferases makes them desirable tools for applications in the pharmaceutical industry [14]. Coupling glucosyltransferase with sucrose synthase (SuSy) in a one-pot cascade reaction creates an in situ recycling system of uridine diphosphate glucose (UDP-glucose) from UDP and sucrose [15]. This method increases the efficiency of glucosylation and noticeably reduces its cost by minimizing dependence on external supplementation with the expensive cofactor. Moreover, the regeneration of sugar donors prevents reverse glucosylation and product inhibition caused by the released nucleotides [16], thereby significantly enhancing the applicability of enzymatic glucosylation. Numerous glucosyltransferases have been characterized, including dozens that are active against steroidal compounds (SGTs). However, the majority of studies restrict their investigation to the native substrates of the enzyme, while broader substrate exploration has remained limited.

In this study, we investigated eleven glycosyltransferases—eight previously characterized and three uncharacterized enzymes of fungal and archaeal origins. Screening against a panel of 31 structurally diverse steroids provided a deeper understanding of their substrate specificity and revealed previously unexplored promiscuity towards steroid scaffolds. The best-performing enzymes were then leveraged in a SuSy-coupled UDP-glucose regeneration cascade to define operational optima, assess robustness, and demonstrate steroid glucoside synthesis in semi-preparative format, including co-immobilized biocatalyst systems.

## 2. Results

### 2.1. Building SGTs Library

A diverse library of glucosyltransferases was established to enable the glucosylation of structurally diverse steroidal compounds. A core set of seven GTs was selected based on literature and preliminary screening of in-house enzymes (Table 1). To address the scarcity of characterized fungal and archaeal glucosyltransferases, a sequence-based search for putative enzymes was conducted in NCBI databases, as these kingdoms represent a vast reservoir of biocatalytic diversity. Using ten confirmed steroid-active GTs of bacterial, fungal, and plant origin as query sequences (Table S2), the search was targeted to the Archaea kingdom and four fungal orders (Sordariales, Eurotiales, Mucorales, and Hypocreales). This initial search yielded 69 sequences putatively encoding steroid glucosyltransferases. Subsequent filtering based on the prediction of soluble expression in *Escherichia coli* using SoluProt analysis narrowed the list to 26 sequences (Table S3).

The cladogram, reconstructed from the provided sequence alignment, revealed distinct evolutionary relationships among the analyzed glucosyltransferases (Figure S1). 3D models of the 26 preselected sequences were generated using AlphaFold3. Subsequent evaluation excluded ten more candidates due to low confidence scores (pLDDT < 70) or a lack of structural homology to known glycosyltransferase folds. The remaining 16 candidates were subjected to molecular docking with prednisolone and UDP-glucose to probe the topology and steric compatibility of their active site cavities. Based on the docking results, the three most promising enzymes were selected for experimental characterization of their activity and substrate scope (Table 1).

### 2.2. Substrate Scope Investigation

**Figure 1:**
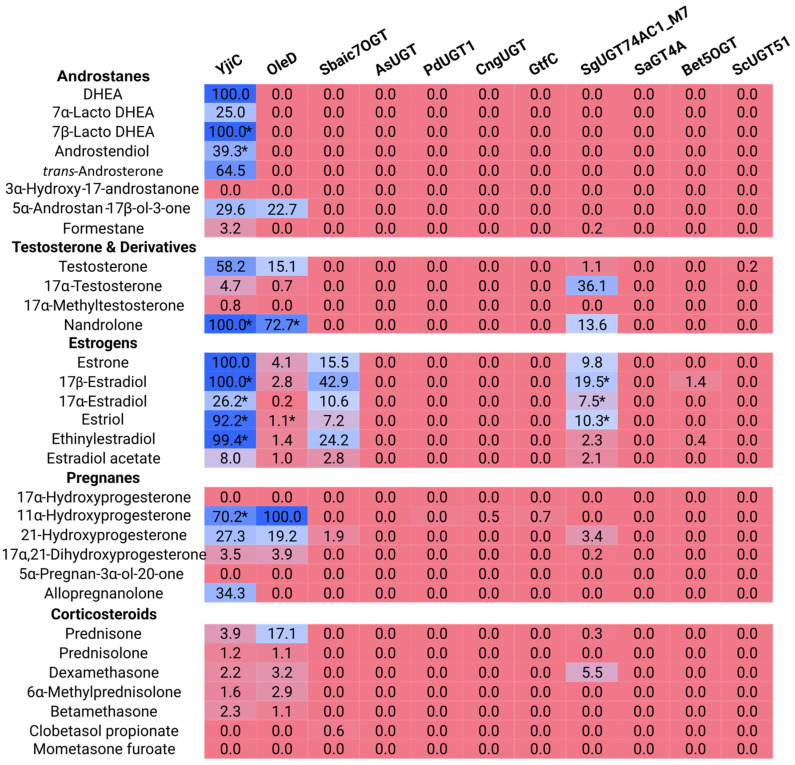
Heat map representing conversions (%) of the panel of steroid compounds by the investigated glucosyltransferases. Asterisk (*) indicates reactions where more than one product was detected. Conversion was based on the UHPLC analysis. Chromatograms, UV–Vis, and LC–MS analyses are provided in the Supplementary Materials (Figures S3–S107). Original NMR spectra (^1^H, ^13^C, COSY, HSQC, HMBC) of the reference standards are provided in the section ‘Reference standards and NMR data’ (Figures S108–S137).

The selected glycosyltransferases were heterologously expressed in *E. coli* BL21 strain. SDS-PAGE analysis of cell lysates confirmed the overexpression of five enzymes: YjiC, OleD, Sbaic7OGT, SgUGT74AC1_M7, and Bet5OGT (Figure S2A,B,E). Analysis of the cell pellets revealed that YjiC, OleD, Sbaic7OGT, GtfC, and SgUGT74AC1_M7 were partially retained in the insoluble fraction (Figure S2C,E). The remaining target enzymes were not detected, suggesting poor expression or instability. Nevertheless, the crude lysates from all expression cultures were utilized in the subsequent substrate screening analysis to avoid excluding potentially active enzymes that were present at low levels. The screening encompassed a panel of 31 steroid compounds, including androstanes, testosterone and its derivatives, estrogens, pregnanes and corticosteroids (Table S4). Glucosylation reactions were performed using GTs coupled with sucrose synthase from *Glycine max* (GmSuSy) in an enzymatic cascade. This approach ensured a continuous surplus of UDP-glucose through the in situ regeneration from sucrose and catalytic amounts of UDP. The reaction results are summarized in Figure 1.

The screened glucosyltransferases exhibited distinct substrate specificities (Figure 1). YjiC and OleD were the most promiscuous, demonstrating broad activity across multiple steroid classes, including androstanes, estrogens, and pregnanes. A more selective profile was observed for Sbaic7OGT and SgUGT74AC1_M7, both of which glucosylated estrogens. SgUGT74AC1_M7 displayed additional activity against selected testosterone derivatives. In contrast, seven GTs—Bet5OGT, AsUGT, PdUGT1, CngUGT, GtfC, SaGT4A and ScUGT51—showed negligible activity. The lack of observable soluble protein for the latter six GTs (Figure S2) indicates that their inactivity is likely a consequence of poor expression in *E. coli*. Furthermore, in vivo reactions conducted with these enzymes also failed to yield detectable product, supporting the conclusion that these enzymes were not functionally expressed under the tested conditions.

A detailed analysis of the substrate scope revealed distinct structure-activity relationships. YjiC demonstrated exceptional promiscuity, showing activity towards steroids with free hydroxyl groups at the C3-, C4-, C11-, C17- and C21-positions, achieving quantitative or near-quantitative conversion of substrates such as DHEA, nandrolone, estrone, and 17β-estradiol. The formation of several glycosylation products resulted from the presence of more than one hydroxyl group in the substrate structure, each of which could act as a potential glucosylation site. As glucose was conjugated exclusively to hydroxyl groups, estradiol, bearing hydroxyl groups at C-3 and C-17, afforded two distinct glucosides. In contrast, OleD exhibited a narrower specificity, with high conversion efficiency confined to a limited set of substrates, including testosterone, nandrolone, and 11α-hydroxyprogesterone. Notably, Sbaic7OGT and SgUGT74AC1_M7 were identified as the most effective catalysts for the glucosylation of 17β-estradiol and 17α-testosterone, respectively.

The stereochemical configuration of the hydroxy group was a critical determinant of catalytic activity. This was shown by the full conversion of 7β-Lacto DHEA over its 7α-epimer by YjiC. The stereospecificity was further confirmed by distinct conversion rates for other epimeric pairs: YjiC preferred the 3β-OH configuration (e.g., *trans*-androsterone, 5α-pregnan-3α-ol-20-one) over the 3α-OH configuration (e.g., 3α-hydroxy-17-androstanone, and allopregnanolone). A general preference for the 17β-OH configuration over the 17α-OH configuration was observed across all active enzymes, as demonstrated by comparison of 17β-estradiol versus 17α-estradiol and testosterone versus 17α-testosterone. A notable exception was SgUGT74AC1_M7, which exhibited a strong preference for the 17α-OH configuration of testosterone while maintaining its selectivity for 17β-estradiol.

The introduction of bulky or complex side chains, as found in 17α-methyltestosterone or corticosteroids such as prednisolone and dexamethasone, drastically reduced conversion across all tested enzymes, indicating that steric hindrance is a primary determinant of substrate specificity.

For representative estrogen and androgen substrates, product identities were assigned using authenticated reference standards confirmed by 1D/2D NMR and matched by retention time/UV-Vis spectra, including verification by co-injection where applicable. In all confirmed glucosides, the glucose anomeric proton (H-1′) appeared as a doublet with J in the 7–8 Hz range, supporting β-configuration. Regioselectivity was verified by HMBC, where H-1′ showed long-range correlation to the aglycone carbon bearing the glycosyl substituent (C-3 or C-17, depending on the product), and HSQC/COSY provided complete resonance assignment for both the steroid and carbohydrate moieties. The original ^1^H/^13^C and 2D NMR spectra (COSY, HSQC and HMBC) for all reference standards/characterized products are provided in the Supplementary Materials (Reference standards and NMR data). Estradiol yielded two monoglucoside regioisomers (3-*O*- and 17-*O*-), and estrone and estradiol 17-acetate afforded the corresponding 3-*O*-glucosides. In addition, reactions with estradiol produced a more polar species consistent with a diglucosylated product. Preparative TLC enabled separation of the diglucoside fraction from monoglucosides.

Notably, preparative TLC fractions showed distinct solubility behavior: material dissolving in acetone-*d_6_* corresponded predominantly to the 17-*O*-glucoside, whereas the residue requiring dissolution in DMSO-*d_6_* contained mainly the 3-*O*-glucoside, facilitating practical enrichment of regioisomers for NMR confirmation.

### 2.3. Optimization of Reaction Conditions

**Figure 2:**
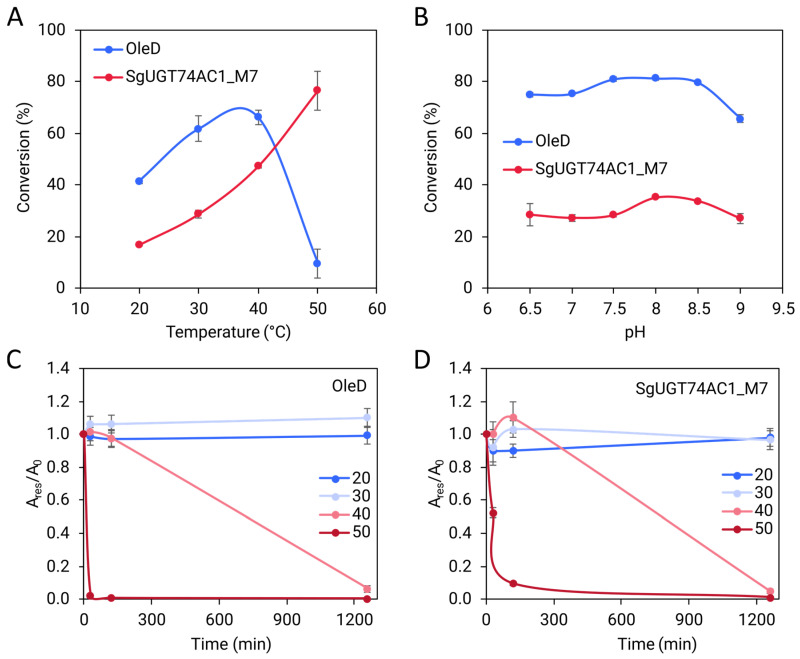
Conversion of nandrolone by OleD-GmSuSy cascade and 17α-testosterone by SgUGT74AC1_M7-GmSuSy cascade in different (**A**) temperatures and (**B**) pH. The residual activity of (**C**) OleD-GmSuSy and (**D**) SgUGT74AC1_M7-GmSuSy after incubation in different temperatures (°C). Error bars represent the standard deviations obtained from three individual replicates.

Having established the substrate scope and identified the most promising biocatalysts, we next optimized the reaction conditions for steroid glucosylation. The bacterial GT OleD and the plant GT SgUGT74AC1_M7 were selected for this purpose, and their catalytic performance was evaluated using nandrolone and 17α-testosterone as model substrates, respectively. As in the substrate scope experiments, reactions were performed using a UGT-GmSuSy cascade. The catalytic performance of the enzymes was characterized across a temperature range of 20–50 °C and a pH range of 6.5–9.0 (Figure 2). For OleD, initial endpoint measurements indicated a temperature optimum between 30 and 40 °C (Figure 2A), while time-course analyses revealed sustained activity from 30 to 50 °C (Figure S105A). OleD exhibited a broad pH optimum between 6.5 and 8.5, with a slight decline in activity under more alkaline conditions (Figure 2B). SgUGT74AC1_M7 exhibited a narrower pH profile than OleD, with an optimum at approximately pH 8.0 (Figure 2B), and preference for higher temperature (Figure 2A), as confirmed by time-course analysis (Figure S105B).

The operational stability of both enzymes was assessed by measuring their residual activity after incubation at various temperatures (Figure 2C,D). OleD demonstrated good stability at 20 and 30 °C and moderate stability at 40 °C, but its activity decreased rapidly at 50 °C, with a half-life of less than 30 min (Figure 2C). In stark contrast, SgUGT74AC1_M7 exhibited better thermostability, retaining 50% of its activity after 30 min at 50 °C (Figure 2D). Considering the combined metrics of catalytic activity and operational stability, 30 °C was established as the optimal reaction temperature for both enzymes.

### 2.4. Optimization of Cascade Immobilization

To improve the operational stability of the glucosylation cascade, a co-immobilization strategy based on His-tagged proteins and IMAC was employed. The GmSuSy-OleD system was used to screen a series of Chromalite^®^ MIDA resins functionalized with Ni^2+^, Co^2+^, Cu^2+^, Fe^2+^, or Zn^2+^ ions. The immobilization efficiency was determined by quantifying specific and recovered activity of the catalyst in nandrolone glucosylation assays.

**Figure 3:**
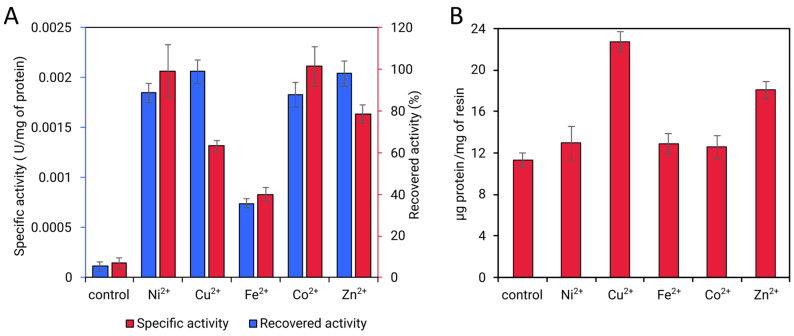
(**A**) Specific and recovered activities of OleD-GmSuSy cascade after the immobilization process on different resins. (**B**) Protein loading efficiency on the tested immobilization resins. The control represents the resin without divalent cations. Error bars indicate the standard deviation from three individual replicates.

The efficiency of the immobilization process was dependent on the type of metal ion used for resin chelation (Figure 3). The Cu^2+^-chelated resin exhibited the highest protein loading capacity (22.7 µg/mg of resin) which correlated with the highest total activity recovery (99.0%). Similarly, the Zn^2+^ resin showed strong binding (18.06 µg/mg of resin) and high recovery (98.0%). However, high protein loading did not strictly translate to the highest specific activity. The Ni^2+^ and Co^2+^ resins, despite having significantly lower protein binding capacities (12.9 and 12.5 µg/mg of resin, respectively) compared to Cu^2+^, achieved the highest specific activities (0.0021 U/mg) This suggests that Ni^2+^ and Co^2+^ ions offer superior selectivity for the target enzymes. In contrast, the Fe^2+^-chelated resin proved inefficient. Although it bound a comparable amount of protein (12.9 µg/mg of resin) to the highly active Ni^2+^ resin, it retained only 35.7% of the initial activity. This indicates likely enzyme inactivation or low selectivity for His-tagged proteins, making it the least suitable carrier for this specific enzyme cascade. The control resin showed the lowest binding (11.3 µg/mg of resin) and negligible activity, confirming that the immobilization process is driven by specific metal affinity interactions.

### 2.5. Semi-Preparative Glucosylation

**Figure 4:**
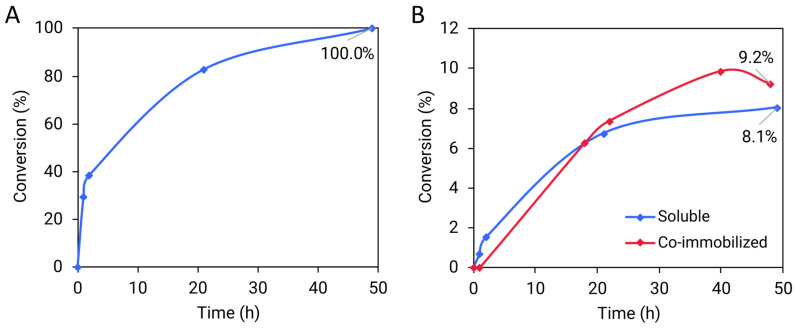
Time course of glucosylation of (**A**) 20 mg of 17α-testosterone by SgUGT74AC1-M7- GmSuSy cascade and (**B**) 100 mg of betamethasone by YjiC-GmSuSy cascade in soluble and co-immobilized state.

**Figure 5:**
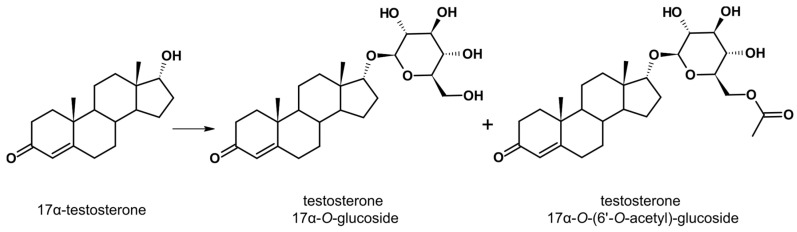
The scheme of tandem glucosylation and acetylation of 17α-testosterone by *E. coli* lysate containing recombinant SgUGT74AC1_M7-GmSuSy cascade.

The scalability of the most promising GTs was evaluated in semi-preparative-scale glucosylation cascades with sucrose synthase (GmSuSy). Two systems were tested: one coupling GmSuSy with SgUGT74AC1_M7 for the glucosylation of 17α-testosterone, and a second coupling GmSuSy with YjiC for the glucosylation of betamethasone. The SgUGT74AC1_M7 cascade achieved quantitative conversion of 17α-testosterone within 49 h (Figure 4A). Although only one hydroxyl group is available for glycosylation, two major products were detected chromatographically (Figure S106)—a result not observed during the 30 min substrate scope analysis. Purification of both products demonstrated that the minor product is the epitestosterone glucoside, while the dominant product results from glucosylation accompanied by additional acylation on the sugar moiety—identified as a 6′-*O*-acetylated glucoside by NMR. The proposed tandem glucosylation and acetylation pathway of 17α-testosterone is shown in Figure 5. For the major epitestosterone-derived product, additional acetyl resonances (^1^H ~2.00 ppm; ^13^C ~170 and ~20 ppm) together with the sugar-region 2D correlations were consistent with 6′-*O*-acylation of the glucose moiety (see reference standards and NMR data in Supplementary Materials).

In contrast, the YjiC-catalyzed glucosylation of betamethasone proceeded with low efficiency, reaching only 8% conversion after 49 h (Figure 4B and Figure S107). To address this limitation, a co-immobilized system was implemented. However, it yielded only a marginal improvement, increasing conversion to just 9% after a 48 h reaction period (Figure 4B).

## 3. Discussion

Enzymatic glucosylation is of significant interest in biocatalysis research, as evidenced by the numerous in vivo and in vitro enzymatic routes developed over the past two decades [17,18,19,20,21,22]. These efforts have largely focused on facilitating the cost-efficient glycosylation of natural products [23,24,25]. However, the glucosylation of steroids remains a greatly under-investigated area. Currently, the scarcity of steroid glycosyltransferases (SGTs) with well-characterized substrate scopes hinders the broad application of these methods. A notable exception is OcUGT1 from *Ornithogalum caudatum*, which was screened against 25 steroidal compounds [26]. We addressed this gap by investigating novel biocatalytic routes for steroid glucosylation. Our results unveil regio- and stereoselectivities previously undescribed for established enzymes, such as OleD and YjiC. Moreover, less-characterized GTs, such as SgUGT74AC1_M7 and Sbaic7OGT, exhibited unique and previously undetected catalytic profiles. In parallel, we employed sequence mining to identify novel SGTs originating from potent enzymatic reservoirs, including archaea and fungi. However, no functional enzymes were obtained, as the selected candidates were poorly expressed in the bacterial host. This outcome highlights the bottleneck in the discovery of novel steroid glycosyltransferases, particularly regarding their functional expression in heterologous hosts. Based on our experience, routine optimization of expression parameters (such as temperature, induction time, and inducer concentration) or straightforward *E. coli* strain screening rarely yields acceptable results, while being inherently time- and resource-consuming. More sophisticated molecular strategies can also be deployed, including (a) evaluating alternative expression hosts, such as yeast, insect cells, or cell-free platforms; (b) employing solubility-enhancing fusion tags (e.g., MBP, SUMO, GST) equipped with a specific protease cleavage site; (c) co-expressing molecular chaperones (GroEL/ES, DnaK/DnaJ/GrpE) to facilitate proper folding; and (d) screening alternative vectors, promoters, and ribosome-binding sites to finely tune expression levels with folding capacity. Implementing these advanced workflows can significantly increase the soluble enzyme fraction, potentially uncovering catalytic activity that is masked under the current screening setup. However, these steps inevitably increase biocatalyst production costs, which is undesirable from an industrial perspective. Given the massive number of putative UGT sequences, investing efforts into variants that exhibit poor initial expression due to misfolding, truncation, aggregation, or toxicity is counterproductive. To streamline this workflow, more accurate predictive algorithms would be highly beneficial to filter out unpromising candidates already at the in silico stage, minimizing the necessity for expensive and tedious trial-and-error optimization downstream.

### 3.1. Substrate Scope and Selectivity

The glycosyltransferases YjiC from *B. licheniformis* and OleD from *S. antibioticus* are established as “workhorse” biocatalysts in the glucosylation of natural products. Their broad substrate promiscuity has been confirmed against a diverse scaffolds, including flavonoids [27,28], stilbenoids [29,30], macrolides [31,32], or phenols [33,34]. However, despite this versatility, they have not yet been screened against a comprehensive library of steroidal compounds. This is noteworthy, given that the YjiC homolog from *Terribacillus* sp. PAMC 23288 (52.4% sequence identity) was reported to perform quantitative conversion of testosterone and nandrolone to their corresponding 17β-glucosides [8]. Furthermore, this homolog exhibits minor activity towards prednisone and cortisone [8], and is capable of attaching a glucose moiety at the C21-OH position of corticosterone [35]. Similarly, Bs-YjiC from *Bacillus subtilis* 168 (57.3% sequence identity) has been shown to glycosylate protopanaxadiol (PPD) and PPD-type ginsenosides at the C3-OH and C12-OH positions [36]. More recently, YjiC itself was identified as an efficient catalyst for the C3 glucosylation of neuroactive 3α-OH-5β-H steroids, achieving a 93% yield when coupled with a sucrose synthase regeneration system [37]. In this study, YjiC from *B. licheniformis* confirmed activity towards the 3β-OH and 17β-OH positions, exhibiting strict stereoselectivity by yielding no or weak conversion of 3α-OH and 17α-OH substrates. Interestingly, the enzyme mediated the conversion of the 11α-hydroxyl group in 11α-hydroxyprogesterone and the C4-OH in formestane—regioselectivities that have not been previously reported. In contrast, YjiC displayed no activity towards the 11β-position characteristic of corticosteroids. Additionally, we observed minor activity towards the C21-OH group, a finding consistent with the activity profile reported for the *Terribacillus* sp. PAMC 23288 homolog.

The OleD glucosyltransferase, particularly its engineered variant OleD ASP (P67T, S132F, A242V [38]), has been effectively utilized for the glucosylation of steroid scaffolds, with a primary focus on cardiotonic steroids (CTS). Previous structure-activity analyses revealed that OleD ASP favors CTS substrates featuring a 3β-OH group and *cis*-A/B ring fusion [39]. Kinetic data further suggested that while a C14–C15 epoxide enhances turnover, substituents at C16 and C17 exert little influence on the biocatalysis of substrates such as resibufogenin. Additionally, the wild-type OleD was reported to catalyze the C12-glucosylation of digoxigenin, effectively overcoming the significant steric hindrance associated with this site [40]. In the present study, we explored the catalytic potential of wild-type OleD against a broader library beyond CTS scaffolds. Our results confirmed a stringent selectivity towards the 17β-OH moiety, with only marginal activity observed against 17α-OH-epimers. However, unlike the ASP mutant, which readily glycosylates 2-methoxyestradiol at the C3-OH and 17β-OH positions [30], the wild-type enzyme exhibited limited efficacy towards estrogens. Most notably, OleD demonstrated unprecedented activity towards the 11α-OH moiety of 11α-hydroxyprogesterone, which similarly to YjiC is first time observed. Furthermore, the enzyme successfully glycosylated the C21-hydroxyl group of 21-hydroxyprogesterone and prednisone, expanding its known substrate scope to include these previously undescribed biotransformations.

Sbaic7OGT is a specialized 7-*O*-glycosyltransferase evolved for the flavonoid biosynthetic pathway in *Scutellaria baicalensis*. Its active site is shaped to accommodate the polyphenolic structure of flavones such as baicalein and wogonin [41]. Notably, this enzyme was recently employed in an engineered *E. coli* strain for the high-titer glucosylation of naringenin to prunin, achieving yields exceeding 9 g/L [42]. Despite its robust activity and favorable heterologous expression in bacterial hosts, this glycosyltransferase has remained largely under-investigated since its initial discovery in 2000. Driven by preliminary data suggesting a broad substrate scope across various flavonoids (data not published) and chalcones [43], we evaluated its catalytic potential against a library of steroidal compounds. Sbaic7OGT proved to be active almost exclusively on estrogen scaffolds, where it exhibited a distinct selectivity toward the β-isomers. The only exception to this estrogen-specific profile was its minor activity toward 21-hydroxyprogesterone.

The SgUGT74AC1_M7 is a septuple mutant (T79Y/L48M/R28H/L109I/S15A/M76L/H47R [44]) of a mogroside 3-*O*-glucosyltransferase from *Siraitia grosvenori*. Beyond its native substrate, this biocatalyst has been shown to accept flavonoids such as naringenin and quercetin [45]. The M7 mutant demonstrated more than 400-fold increase in catalytic efficiency toward mogrol compared to the wild-type enzyme, attributed to a more flexible binding pocket capable of accommodating various tetracyclic triterpenoids [44]. Furthermore, it can utilize both UDP-glucose and UDP-*N*-acetylglucosamine. In the present study, we established that this mutant enzyme is an efficient catalyst for the glucosylation of 17-α-testosterone, representing the first reported biocatalytic synthesis of this specific glucoside. Additionally, the mutant displayed moderate activity not only toward nandrolone but also across the entire panel of tested estrogens.

The substrate scope screen revealed pronounced differences in promiscuity across the evaluated GTs. Consistent with prior observations for broad-specificity bacterial GTs, YjiC and OleD displayed the widest activity range across diverse steroid scaffolds, supporting their role as practical “workhorse” enzymes for glycodiversification. In contrast, several enzymes in the panel exhibited more selective behavior, showing preferences for particular steroid subclasses or for specific functional group patterns. Such behavior is expected for GTs whose acceptor-binding sites accommodate only a limited set of steric and electronic features. For steroid substrates, accessibility and orientation of hydroxyl groups are particularly decisive: an equatorial hydroxyl group can be more readily approached by the catalytic base and aligned for nucleophilic attack, whereas axial positioning, neighboring substituents, and conformational constraints may disfavor productive binding. The heat map–type representation used here is therefore useful not only as a screening readout but also as an empirical structure–activity relationship (SAR) map that can guide future enzyme engineering and substrate selection.

### 3.2. Process Optimization

As the ultimate objective of the current investigation, we undertook the scale-up of the enzymatic process to a semi-preparative level. This transition was preceded by a systematic optimization of reaction and immobilization parameters using selected bacterial (OleD) and plant-derived (SgUGT74AC1) glucosyltransferases coupled with GmSuSy. While OleD displayed a classic mesophilic profile with a broad pH tolerance (6.5–8.5), the SgUGT74AC1_M7 variant exhibited a significantly higher thermal optimum and superior robustness at 50 °C. This observations are consistent with its previously reported activity toward its native substrate, mogrol [46]. As there are currently no records regarding the pH and thermal parameters of the wild-type SgUGT74AC1, it remains unclear whether this enhanced stability stems from the protein engineering efforts or the native characteristics of the scaffold. The former is more likely, as plant-derived enzymes typically do not exhibit such high thermal tolerance. Regardless, these favorable thermophilic attributes distinguish SgUGT74AC1_M7 as a robust biocatalyst, highly suitable for implementation in large-scale preparative conditions.

The observed protein loading capacity across the functionalized Chromalite^®^ MIDA resins closely followed the Irving-Williams series (Fe^2+^ < Co^2+^ < Ni^2+^ < Cu^2+^ > Zn^2+^), with the Cu^2+^-chelated resin achieving the highest sequestration of the glucosylation cascade. This superior loading is attributed to the high stability constants characteristic of Cu^2+^ complexes [47]. However, despite the quantitative recovery achieved with Cu^2+^, the specific activity was significantly lower than that observed for Ni^2+^ and Co^2+^. The high non-specific affinity of Cu^2+^ may promote “crowding” on the resin surface, leading to detrimental protein–protein interactions. In contrast, the Ni^2+^ and Co^2+^ resins, which favor a more symmetrical, rigid octahedral coordination, demonstrated superior selectivity. This likely ensured a more favorable, oriented display of the His-tagged enzymes, thereby maximizing the effective specific activity despite the lower total protein loading. While the Fe^2+^-chelated resin exhibited protein loading levels comparable to Ni^2+^, it proved to be the least effective carrier, retaining only 35.7% of the initial activity. This poor performance can be attributed to the lower coordination stability of Fe^2+^ within the Irving-Williams series and largest ionic radius, resulting in weaker and less selective interactions with the His-tagged cascade.

The transition to semi-preparative scales yielded contrasting results that highlight the inherent challenges of steroid biocatalysis. We evaluated the optimized processes for the glucosylation of betamethasone using the YjiC-GmSuSy cascade, which had previously shown limited conversion during the screening phase, and 17α-testosterone using SgUGT74AC1_M7, as its corresponding glucoside has not yet been reported via biocatalytic synthesis. Despite process optimization, the glucosylation of betamethasone reached a maximum conversion of only 8% using cell lysates. The application of a co-immobilized catalyst provided only a marginal improvement of approximately 1%, suggesting that the primary bottleneck is not enzyme instability, but rather substrate inhibition or the steric hindrance of the highly substituted betamethasone scaffold. Furthermore, the poor solubility of the corticosteroid in the aqueous reaction medium likely limited its accessibility to the catalytic center. These findings emphasize that while ‘workhorse’ enzymes like YjiC are highly versatile, their efficacy remains constrained when applied to complex, bulky steroidal cores. Even extending the reaction time to 48 h failed to enhance conversion, suggesting that steric limitations and/or poor substrate accessibility may define the conversion limit under these conditions. In stark contrast, the SgUGT74AC1_M7 catalyzed glucosylation of 17α-testosterone was highly efficient, achieving quantitative conversion. However, the scale-up process revealed the emergence of two distinct products instead of the single product detected during the initial screening, likely due to the significant shorter duration of the preliminary assays (30 min vs. 48 h). Structural elucidation by NMR confirmed that, in addition to the anticipated 17α-glucoside, the reaction yielded an unexpected 6-acetyl-glucoside derivative. Similar observations were reported by Liu and Kong, who utilized *E. coli* cell lysates for the glucosylation of testosterone and estradiol. They identified acetylated glucosides as the final products and demonstrated that the endogenous *E. coli* galactoside *O*-acetyltransferase (LacA) possesses steroidal glycoside acyltransferase activity, specifically catalyzing the acetylation of the sugar moieties of steroid 17β-glucosides [48]. While other studies have shown that *E. coli* maltose *O*-acetyltransferase can acetylate glucosides of various flavonoids and iridoids [49], Liu and Kong did not observe such activity toward steroid glucosides. Our results suggest that a similar mechanism, likely mediated by the native LacA, is at play here. However, this represents the first reported instance of the acetylation of a steroid 17α-glucoside, and need to be further investigated to confirm LacA action. It is widely recognized that glycosylation enhances the solubility and bioavailability of hydrophobic compounds while favorably modifying their pharmacokinetics. Furthermore, the acylation of the sugar moiety has been shown to increase the stability of specific glycosides. Consequently, these intriguing results not only identify a novel activity for native *E. coli* enzymes toward 17α-substrates but also establish a unique biocatalytic pathway for the production of novel compounds with significant pharmaceutical potential.

## 4. Materials and Methods

### 4.1. Materials

Unless otherwise stated microbiological media components and other chemicals were bought from Sigma-Aldrich (St Louis, MO, USA) and SERVA Electrophoresis Gmb (Heidelberg, Germany). Antibiotics were purchased from Cayman Chemical Company (Ellsworth, ME, USA). Steroids were bought from Pol-Aura (Morąg, Poland), Sigma Aldrich (St Louis, MO, USA) Thermo Fisher Scientific (Waltham, MA, USA) or were synthesized at the Department of Food Chemistry and Biocatalysis at the Wrocław University of Environmental and Life Sciences [50]. The UPLC-grade solvents (acetonitrile (≥99.9% purity), and methanol (99.9% purity)) were bought from Merck KGaA (Darmstadt, Germany). Molecular biology reagents, including T4 DNA ligase, restriction enzymes (BsaI-HFv2, BbsI-HF), Plasmid Miniprep Kit, Taq PCR Kit and chemically competent *E. coli* cells—NEB 5-alpha, and BL21 (DE3) were bought from New England Biolabs Inc. (NEB, Ipswich, MA, USA). All genes were codon-optimized and synthesized by Invitrogen GeneArt Gene Synthesis (Thermo Fisher Scientific, Waltham, MA, USA). Primers utilized for PCR and sequencing were acquired from Sigma-Aldrich (St Louis, MO, USA). All primers, genes, plasmids and *E. coli* strains utilized within this study are listed in Table S1. Purity grade of utilized reagents was as follows: UDP-glucose (≥98%), UDP (95%), sucrose (≥99.5%), 6α-methylprednisolone (≥95%), betamethasone (97%), mometasone furoate (≥96%), clobetasol propionate (≥98%), dexamethasone (≥98%), prednisone (≥98%), prednisolone (≥99%), nandrolone (≥99.0%), dehydroepiandrosterone (DHEA) (≥98%), 7α-lacto DHEA (≥97%), 7β-lacto DHEA (≥97%), androstendiol (≥98%), trans-androsterone (≥98%), 3α-hydroxyl-17-androstanone (≥97%), 5α-andorstan-17B-ol-3-one (≥98%), formestane (≥99%), testosterone (≥99%), 17α-testosterone (≥99%), 17α-methyltestosterone (≥99%), estrone (≥99%), 17β-estradiol (≥99%), 17α-estradiol (≥98%), estriol (≥98%), ethinylestradiol (≥98%), estradiol acetate (≥99%), 17α-hydroxyprogesterone (≥98%), 11α-hydroxyprogesterone (≥97%), 21-hydroxyprogesterone (≥96%), 17α,21-dihydroxyprogesterone (≥98%), 5α-pregnan-3α-ol-20-one (≥97%), allopregnanolone (≥98%). Authentic reference steroid glucosides used for chromatographic peak assignment were confirmed by 1D/2D NMR (^1^H, ^13^C, COSY, HSQC, HMBC) and are provided in the Supplementary Materials (Section “Reference standards and NMR data”).

### 4.2. Alignments and Modeling

Amino acid (aa) sequences of OleD [51], YjiC [52], SaGT4A [53], SgUGT74AC1_M7 [44], UGT74AN2 [54], UGT74AN3 [55], PpUGT6 [56], ScUGT51 [57], UGT80A40 [58], UGT80A41 [58] glycosyltransferases (GT) with confirmed activity against steroidal compounds (SGT) were subjected to BLASTp (protein-protein BLAST, https://blast.ncbi.nlm.nih.gov/Blast.cgi, accessed on 5 June 2026) analysis against the genomes of Archaea kingdom (taxid 2157) and fungi orders: Sordariales (taxid:5139), Eurotiales (taxid:5042), Mucorales (taxid:4827), Hypocreales (taxid:5125) deposited in the National Center for Biotechnology Information non-redundant protein sequences (nr) database. From each protein alignment, the first ≤ 5 matches (sorted by E value) with less than 60% identity, recognized as glycosyltransferase-type protein and which had 350–650 aa, were selected. Subsequently, duplicates from parallel alignments were removed, resulting in 22 unique protein sequences of archaeal origin and 47 of fungal origin. Next, the SoluProt analysis (https://loschmidt.chemi.muni.cz/soluprot/, accessed on 5 June 2026 [59]) was performed for all selected sequences. Sequences with a score of less than 0.45 were excluded from further analysis, reducing the dataset to 4 archaea and 22 fungal protein sequences (Table S2). Multiple Sequence Alignment (MSA) of selected sequences with input sequences were performed with the Clustal Omega algorithm (https://www.ebi.ac.uk/jdispatcher/msa/clustalo, accessed on 5 June 2026) [60] to build cladogram using iTOL software (https://itol.embl.de/, accessed on 5 June 2026) [61] (Figure S1). The 3D models of remaining protein sequences were constructed using AlphaFold3 model (https://alphafoldserver.com, accessed on 5 June 2026) [62]. A comparison of models to the GT structures subjected to the BLASTp was made. The analysis focused on the similarities in the active site area. Models lacking a standard active site cavities were excluded from further analysis. All molecular docking experiments were performed using the UCSF Chimera 1.16 [63] with AutoDock Vina 1.1.235 [64] and PyMOL Molecular Graphics System, Version 3.0.5 Schrödinger, LLC. Docking of UDP-glucose was performed on structures aligned to OleD (PDB: 7XX4). A volume box encompassing the sugar donor cavity was individually defined for each model to ensure proper coverage. Docking of prednisolone, as a model sugar acceptor, was then performed in the adjacent cavity near the UDP-glucose binding site, with a separately defined volume box for each model to ensure precision. All selected docking positions were based on the highest score and did not clash or come into contact with the UDP-glucose cofactor and were in orientation and distance indicating chance of reactivity.

### 4.3. Plasmid Cloning and Transformation

Codon-optimized genes were cloned into pRhaBAD_12 vector [65,66] along with N-His_6x_-tag according to the Golden Standard Modular Cloning (GS MoClo) assembly [67]. Plasmids were transformed into the chemically competent *E. coli* cells—NEB 5-alpha for plasmid maintenance, or BL21 (DE3) (NEB, Ipswich, MA, USA) for protein production. Positive clones were identified through blue-white screening on selective media supplemented with X-Gal (20 µg/mL) and the appropriate antibiotic (kanamycin (30 mg/L)). The plasmids sequences were confirmed through Sanger sequencing conducted by Macrogen Europe BV (Amsterdam, The Netherlands).

### 4.4. Enzyme Production and Purification

The enzyme production was carried out overnight in 0.5 L M9 minimal medium supplemented with kanamycin (30 mg/L) in a 2 L baffled flask at 25 °C with 120 rpm agitation. Production medium was inoculated with an 1% (*v/v*) of overnight preculture. Enzyme expression was induced when the OD_600_ reached 0.6–0.8. The overnight culture was centrifuged (4000× *g*, 30 min, 4 °C) and the cell pellet was resuspended in 35 mL of Reaction Buffer (50 mM HEPES, 50 mM KCl, 10 mM MgCl_2_, pH 7.5), sonicated (6 min program with 30 s pulse and 30 s pause, 85% amplitude) in an ice bath using Vibra-Cell Ultrasonic Liquid Processor VCX 130 (Sonics & Materials, Inc., Newtown, CT, USA), and centrifuged (14,000× *g*, 45 min, 4 °C). The cell lysate was either stored at –80 °C or subjected to the purification or immobilization protocol. The total protein concentration of the cell lysate was determined using the Bradford assay with BSA as a reference for the calibration curve. For purification, 6 mL of cell lysate was applied to a His SpinTrap columns (His-SpinTrapTM, Cytiva, Uppsala, Sweden) and proceeded according to the manufacturer’s protocol. The His_6x_-tagged enzyme was eluted in Elution Buffer 50 mM HEPES, 50 mM KCl, 10 mM MgCl_2_, 500 mM NaCl, 500 mM imidazole, pH 7.5). The purified enzyme was directly used for 10% SDS-PAGE analysis, with proteins stained by Coomassie Brilliant Blue (Cepham Life Sciences, Inc., Fulton, MD, USA). PageRuler Plus Prestained Protein Ladder (Thermo Fisher Scientific, Waltham, MA, USA) was used as a molecular size marker.

### 4.5. Enzyme Co-Immobilization

The enzymes were co-immobilized on Chromalite^®^ MIDA for affinity resins (EcoLab, Saint Paul, MN, USA) pre-charged with Ni^2+^, Co^2+^, Cu^2+^, Fe^2+^ or Zn^2+^ ions. Resin preparation followed the manufacturer’s protocol using HEPES buffer (50 mM, 50 mM KCl, 300 mM NaCl, pH 7.5). Cell lysates containing GTs and GmSuSy were supplemented with 20 mM imidazole and 300 mM NaCl and combined with the resins. Immobilization was performed for 1–2 h at 4 °C with orbital shaking (100 rpm). The resins were then centrifuged (500× *g*, 5 min) and washed twice with Wash Buffer (50 mM HEPES, 50 mM KCl, 300 mM NaCl, 5 mM imidazole, pH 7.5). Supernatants and washing solutions were collected and analyzed for total protein concentration (Bradford assay) and enzyme activity (cascade assay described below). Prepared resins were directly used in the cascade reactions. Recovered activity was defined as the percentage of initial lysate enzymatic activity retained on the resin (%). Specific activity was expressed as units of enzymatic activity per milligram of immobilized protein (U/mg).

### 4.6. Cascade Assay

Screening reactions were performed in 0.2 mL Reaction Buffer with 0.5 mM uridine diphosphate (UDP), 500 mM sucrose, 0.05 mM steroidal substrate (dissolved in 1% (*v/v*) DMSO), and cell lysate as catalyst. Reactions were conducted in U-bottom 96-well polypropylene microplates (Greiner Bio-One, Kremsmünster, Austria) at 30 °C with 800 rpm agitation for 30 min. Semi-preparative reactions were conducted in 20 or 40 mL of Reaction Buffer in 50 mL falcon tubes with 0.5 mM UDP, 500 mM sucrose, 3.5 mM (1 g/L) 17α-testosterone, or 6.4 mM (2.5 g/L) betamethasone (all dissolved in 1% *v*/*v* DMSO), and cell lysate or co-immobilized enzymes as catalyst source. Reaction samples (20 μL) were withdrawn through the catalysis. Reactions were stopped by addition of 3x excess of ice-cold acetonitrile and left for 1 h in 4 °C. Precipitated proteins were centrifuged (4000× *g*, 60 min, 4 °C (microplates) or 14,000× *g*, 5 min, 4 °C (tubes)). Supernatants were diluted 2-fold with MQ water prior to analysis. The reaction progress was evaluated by the UPLC-DAD assay performed on Dionex Ultimate 3000 UHPLC+ instrument (Thermo Fisher Scientific, Waltham, MA, USA) according to specifics described in a previously published paper [65]. The identification of substrates and products was based on authentic standard compounds, determined by their retention times, UV-Vis spectra and by NMR analysis in DMSO-d6: ^1^H-NMR, ^13^C-NMR, 1H-1H-NMR (COSY) and ^1^H−^13^C-NMR (HSQC, HMBC), which were recorded on a DRX Bruker Avance TM 600 (600 MHz) instrument. Where available, peak identity was additionally verified by co-injection (spiking) of reaction mixtures with authenticated reference standards. LC–MS analysis was carried out on an LC–MS 8045 system (Shimadzu, Kyoto, Japan) equipped with a triple quadrupole and diode array detector, operated by LabSolutions software. Separation was achieved on a Kinetex C18 column (3 mm × 100 mm, 2.6 µm, 100 Å) with a pre-column at 35 °C. The mobile phase consisted of water containing 0.01% formic acid (A) and acetonitrile (B) at a flow rate of 0.4 mL/min. The gradient elution program was set as follows: 0–3 min, 20% B; 3–10 min, linear gradient from 20% to 100% B; 10–15 min, isocratic wash at 100% B; 15–17.5 min, linear decrease to 20% B; followed by an equilibration step at 20% B until 23 min. The injection volume was 2 µL. MS operating parameters in positive ionization mode were: nebulizing gas 1.5 L/min, heating gas 10 L/min, drying gas 10 L/min, interface temperature 300 °C, and acquisition in Q1 SIM mode with masses corrected for compound+[H^+^] for all analyzed aglycones, glucosides and acetylglucosides.

### 4.7. Accession Numbers

Synthetic gene sequences will be deposited in the NCBI GenBank database upon manuscript acceptance. All nucleotide and amino-acid sequences used in this study are provided in the Supplementary Materials (Tables S1 and S2).

## 5. Conclusions

In conclusion, this study establishes an integrated screening-to-process platform for steroid glucosylation based on glycosyltransferases coupled with sucrose synthase for in situ UDP-glucose regeneration. Screening of 11 GTs against a panel of 31 structurally diverse steroids revealed pronounced differences in substrate scope and selectivity, identifying YjiC and OleD as the most promiscuous enzymes, whereas Sbaic7OGT and SgUGT74AC1_M7 displayed narrower but potentially synthetically useful selectivity toward estrogens and selected testosterone derivatives. The results further demonstrated that glucosylation efficiency strongly depends on the presence, position, and stereochemical configuration of hydroxyl groups, while bulky substituents and highly substituted corticosteroid scaffolds markedly reduce conversion.

Among the tested systems, SgUGT74AC1_M7 proved particularly effective for the glucosylation of 17α-testosterone and enabled quantitative conversion under semi-preparative conditions, highlighting its potential as a practical biocatalyst for steroid glycoside synthesis. In contrast, the low conversion observed for betamethasone, even after process optimization and co-immobilization, underscored the limitations imposed by substrate sterics and solubility. At the same time, the failure to obtain functionally active newly selected fungal and archaeal candidates under the tested conditions highlights heterologous expression as an crucial bottleneck in glycosyltransferase discovery and implementation. Importantly, structural characterization of representative products confirmed the formation of defined steroid glucosides and revealed an unforeseen 6′-*O*-acetylated epitestosterone glucoside, indicating that additional post-glycosylation modifications may arise in *E. coli*-based biocatalytic systems. Overall, the dataset generated here expands current knowledge of steroid-accepting GTs, provides practical guidance for enzyme selection, and supports the future development of scalable and selective biocatalytic routes to novel steroid glycoconjugates.

## Figures and Tables

**Table 1:** List of the investigated glucosyltransferases.

Origin	Name	Description
**Characterized**		
bacterial	YjiC	glycosyltransferase from *Bacillus licheniformis*
bacterial	OleD	macrolide glycosyltransferase from *Streptomyces antibioticus*
bacterial	GtfC	metagenome-derived glycosyltransferase C
plant	Sbaic7OGT	flavonoid 7-*O*-glucosyltransferase from *Scutellaria baicalensis*
plant	Bet5OGT	betanidin 5-*O*-glucosyltransferase from *Cleretum bellidiforme* (previously *Dorotheanthus bellidiformis*)
plant	SgUGT74AC1_M7	mutant (T79Y/L48M/R28H/L109I/S15A/M76L/H47R) SgUGT74AC1 glucosyltransferase from *Siraitia grosvenorii*
plant	SaGT4A	nuatigenin 3-beta-glucosyltransferase from *Solanum aculeatissimum*
fungal	ScUGT51	sterol glycosyltransferase from *Saccharomyces cerevisiae*
**Uncharacterized**		
fungal	AsUGT	sterol 3-beta-glucosyltransferase from *Apophysomyces* sp. BC1034
fungal	PdUGT1	glycosyltransferase family 1 from *Podospora didyma*
archaeal	CngUGT	glycosyltransferase from *Candidatus Nitrososphaera gargensis* Ga9.2

## Data Availability

All data generated or analyzed during this study are available at the following DOI: https://doi.org/10.57755/5fmf-ba41.

## Supplementary Materials

The following supporting information can be downloaded at: https://www.mdpi.com/article/10.3390/ijms27125232/s1.
