# Supplementary material for: Expanding Steroid Glycodiversity: Tandem Steroid Glucosylation and Acetylation via Enzymatic Cascade
Source: Int J Mol Sci. 2026 Jun 9;27(12):5232. doi: 10.3390/ijms27125232 (PMC13299239; doi:10.3390/ijms27125232)
Supplement: Supplementary file 1 [file ijms-27-05232-s001.zip › ijms-4355480-supplementary.pdf]

Expanding steroid glycodiversity: tandem steroid glucosylation and acetylation via enzymatic cascade

Agata Matera[1,2], Kinga Dulak[1], Sandra Sordon[1], Ewa Huszcza[1], Tomasz Janeczko[1], Jarosław Popłoński[1]

[1] Department of Food Chemistry and Biocatalysis, Wrocław University of Environmental and Life Sciences, C.K. Norwida 25, 50-375 Wrocław, Poland

[2] Department of Chemical Biology, Faculty of Biotechnology, University of Wrocław, Joliot-Curie 14a, 50-383 Wrocław, Poland

Table S1. Genes, primers, expression cassettes, plasmids and *E. coli* strains utilised within this study.

Genes, primers, plasmids and strains	Properties	Source or reference
**Gene**		
GmSuSy	sucrose synthase from *Glycine max*	Genbank: AAC39323.1, OP381218
YjiC	glycosyltransferase from *Bacillus licheniformis*	Genbank: AAU40842, OP381219
OleD	macrolide glycosyltransferase from *Streptomyces antibioticus*	Genbank: ABA42119.2 OR545369
Sbaic7OGT	flavonoid 7-*O*-glucosyltransferase from *Scutellaria baicalensis*	Genbank: BAA83484.1 OR545367
GtfC	glycosyltransferase from Elbe River sediment metagenome	Genbank: AGH18139
SgUGT74AC1_M7	mutant (T79Y/L48M/R28H/L109I/S15A/M76L/H47R) glucosyltransferase from *Siraitia grosvenorii*	Genbank: AEM42999 (WT)
SaGT4A	nuatigenin 3-beta-glucosyltransferase from *Solanum aculeatissimum*	Genbank: Q5H861.1
Bet5OGT	betanidin 5-*O*-glucosyltransferase from *Cleretum bellidiforme* (previously *Dorotheanthus bellidiformis*)	Genbank: CAB56231.1 OR545368
ScUGT51	sterol glycosyltransferase from *Saccharomyces cerevisiae*	Genbank: AJV62864.1
AsUGT	uncharacterised sterol 3-beta-glucosyltransferase from *Apophysomyces sp.* BC1034	Genbank: KAG0187195
PdUGT1	uncharacterised glycosyltransferase family 1 from *Podospora didyma*	Genbank: KAK3386858
CngUGT	putative, uncharacterised glycosyltransferase from *Candidatus Nitrososphaera gargensis* Ga9.2	Genbank: AFU58718
**Primers**		
PS1	AGGGCGGCGGATTTGTCC	[66]
PS2	GCGGCAACCGAGCGTTC	[66]
rha S middle F	GGACGGGATGGCTTTCTGCAATAA	[65]
**Plasmids**		
pRhaBAD_12	pBBR1 ori, KmR, *rhaS, rhaBAD* promoter, T7 terminator	[65]
T7RBS_BC	pUC ori, AmpR, T7 RBS	[67]
N1-6xHis-tag _CD	pUC ori, AmpR, N-His6x-tag	[67]
pMA-T-YjiC_DG	ColE1 ori, AmpR, *YjiC*	[66]
pMA-T-OleD_DG	ColE1 ori, AmpR, *OleD*	This study
pMA-T-Sbaic7OGT_DG	ColE1 ori, AmpR, *Sbaic7OGT*	This study
pSEVA182-GtfC_DG	pUC ori, AmpR, *GtfC*	This study

pSEVA182-SgUGT74AC1_M7_DG	pUC ori, AmpR, *SgUGT74AC1_M7*	This study
pMA-T- SaGT4A_DG	ColE1 ori, AmpR, *SaGT4A*	This study
pMA-T-Bet5OGT_DG	ColE1 ori, AmpR, *Bet5OGT*	This study
pMA-T-ScUGT51_DG	ColE1 ori, AmpR, *ScUGT51*	This study
pMA-T-AsUGT_DG	ColE1 ori, AmpR, *AsUGT*	This study
pMA-T-PdUGT1_DG	ColE1 ori, AmpR, *PdUGT1*	This study
pMA-T-CngUGT_DG	ColE1 ori, AmpR, *CngUGT*	This study
pRhaBAD-GmSuSy	pRhaBAD_12 carrying N-His$_{6x}$-*GmSuSy*	[65]
pRhaBAD-YjiC	pRhaBAD_12 carrying N-His$_{6x}$-*YjiC*	This study
pRhaBAD-OleD	pRhaBAD_12 carrying N-His$_{6x}$-*OleD*	This study
pRhaBAD-Sbaic7OGT	pRhaBAD_12 carrying N-His$_{6x}$-*Sbaic7OGT*	This study
pRhaBAD-GtfC	pRhaBAD_12 carrying N-His$_{6x}$-*GtfC*	This study
pRhaBAD-SgUGT74AC1_M7	pRhaBAD_12 carrying N-His$_{6x}$-*SgUGT74C1_M7*	This study
pRhaBAD- SaGT4A	pRhaBAD_12 carrying N-His$_{6x}$-*SaGT4A*	This study
pRhaBAD-Bet5OGT	pRhaBAD_12 carrying N-His$_{6x}$-*Bet5OGT*	This study
pRhaBAD-ScUGT51	pRhaBAD_12 carrying N-His$_{6x}$-*ScUGT51*	This study
pRhaBAD-AsUGT	pRhaBAD_12 carrying N-His$_{6x}$-*AsUGT*	This study
pRhaBAD-PdUGT1	pRhaBAD_12 carrying N-His$_{6x}$-*PdUGT1*	This study
pRhaBAD-CngUGT	pRhaBAD_12 carrying N-His$_{6x}$-*CngUGT*	This study
**Strains**		
5-alpha	F´ proA$^+$B$^+$ lacI^q Δ(lacZ)M15 zzf::Tn10 (Tet^R) / fhuA2Δ(argF-lacZ)U169 phoA glnV44 Φ80Δ(lacZ)M15 gyrA96 recA1 relA1 endA1 thi-1 hsdR17	New England Biolabs Inc.
BL21 (DE3)	fhuA2 [lon] ompT gal (λ DE3) [dcm] ΔhsdS λ DE3 = λ sBamHIo ΔEcoRI-B int::(lacI::PlacUV5::T7 gene1) i21 Δnin5	New England Biolabs Inc.

Table S2. List of glycosyltransferases that served as an input for the sequence-based search for putative GTs.

Origin	Name	Description	Source or reference
bacterial	*YjiC*	glycosyltransferase from *Bacillus licheniformis*	Genbank: AAU40842, OP381219
	OleD	macrolide glycosyltransferase from *Streptomyces antibioticus*	Genbank: ABA42119.2 OR545369
plant	*SgUGT74AC1_M7*	T79Y/L48M/R28H/L109I/S15A/M76L/H47R mutant of glucosyltransferase from *Siraitia grosvenorii*	Genbank: HQ259620.1
	SaGT4A	nuatigenin 3-beta-glucosyltransferase from *Solanum aculeatissimum*	Genbank: Q5H861.1
	UGT74AN3	steroid glycosyltransferase from *Catharanthus roseus*	Genbank: AYC35244.1

	UGT74AN2	steroid glycosyltransferase from *Catharanthus roseus*	Genbank: AYC35243.1
	PpUGT6	sterol glycosyltransferases from *Paris polyphylla var. Yunnanensis*	Genbank: WET17277.1
	UGT80A40	glucosyltransferase from *Paris polyphylla*	Genbank: WIL59758.1
	UGT80A41	glucosyltransferase from *Paris polyphylla*	Genbank: WIL59759.1
fungal	*ScUGT51*	sterol glycosyltransferase from *Saccaromyces cerevisiae*	Genbank: AJV62864.1

Table S3. List of preselected potential steroid glucosyltransferases.

Kingdom	Species	SoluProt score	Genbank No.
archaea	*Candidatus Nitrososphaera gargensis*	0.57	AFU58718
archaea	*Methanobacterium sp.*	0.535	HOI39757
archaea	*Salinirubellus sp. SYNS196*	0.458	WP_338915726
archaea	*Natronorubrum aibiense*	0.568	WP_152944063
fungal	*Triangularia setosa*	0.55	KAK4175036
fungal	*Cercophora samala*	0.533	KAK0667676
fungal	*Podospora didyma*	0.488	KAK3386858
fungal	*Canariomyces notabilis*	0.551	XP_064666084
fungal	*Triangularia setosa*	0.451	KAK4173154
fungal	*Podospora comata*	0.451	VBB84545
fungal	*Neurospora tetrasperma*	0.496	EGZ78062
fungal	*Penicillium macrosclerotiorum*	0.525	XP_056934836
fungal	*Apophysomyces ossiformis*	0.52	KAF7720861
fungal	*Apophysomyces sp.*	0.586	KAG0187195
fungal	*Lichtheimia hyalospora*	0.689	KAI7881798
fungal	*Backusella circina*	0.57	KAI8889533
fungal	*Triangularia verruculosa*	0.479	KAK4197011
fungal	*Cercophora newfieldiana*	0.457	KAK0643843
fungal	*Thermothielavioides terrestris*	0.500	XP_003652846
fungal	*Chaetomium sp.*	0.527	KAH6856223
fungal	*Rhypophila decipiens*	0.530	KAK4214958
fungal	*Podospora comata*	0.551	VBB78419
fungal	*Stachybotrys elegans*	0.599	KAH7311710
fungal	*Rhizopus microsporus var. microsporus*	0.564	ORE01858
fungal	*Apophysomyces sp.*	0.560	KAG0176993
fungal	*Talaromyces pinophilus*	0.563	GAM42806
fungal	*Rhizopus japonicus*	0.527	AOC55050
fungal	*Rhizopus stolonifer*	0.471	RCI06016
fungal	*Phycomyces blakesleeanus*	0.451	XP_018289456

Table S4. List of the tested steroid compounds.

Name	Structure
**Androstanes**	
DHEA	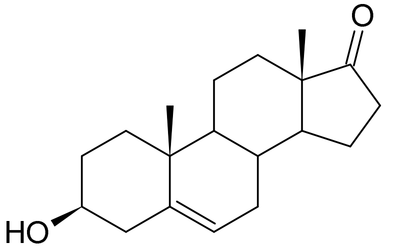
7α-Lacto DHEA	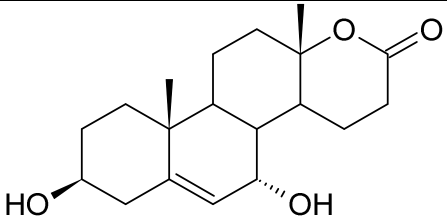
7β-Lacto DHEA	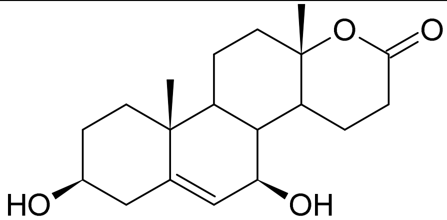
Androstenediol	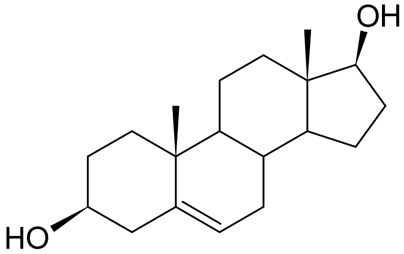
trans-Androsterone	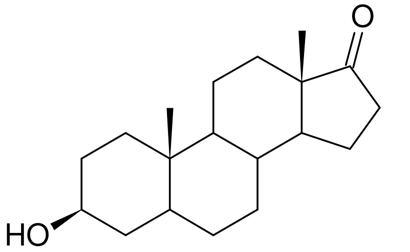
3α-Hydroxy-17-androstanone	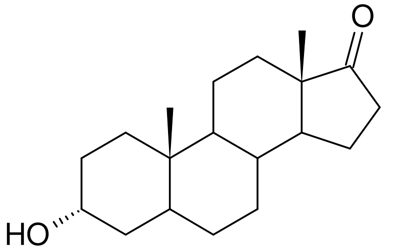

5α-Androstan-17β-ol-3-one	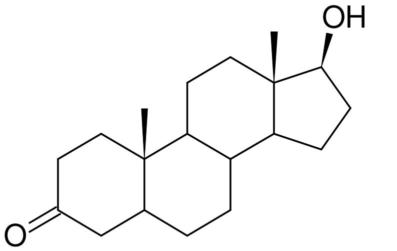
Formestane	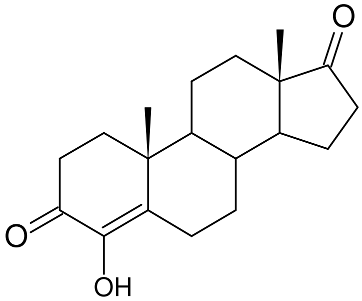
**Testosterone & Derivatives**	
Testosterone	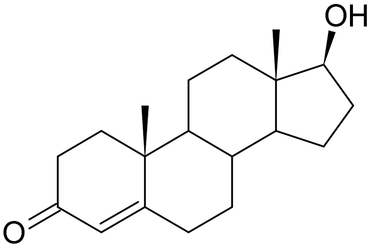
17α-Testosterone	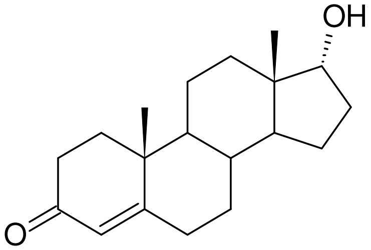
17α-Methyltestosterone	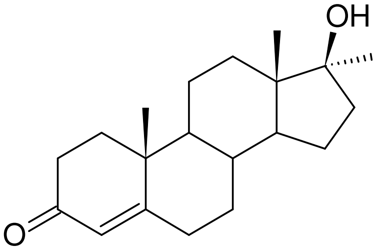
Nandrolone	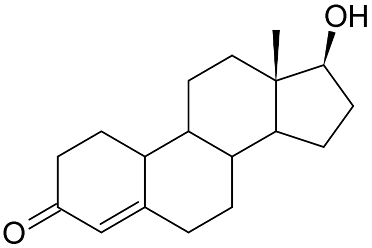
**Estrogens**	

Estrone	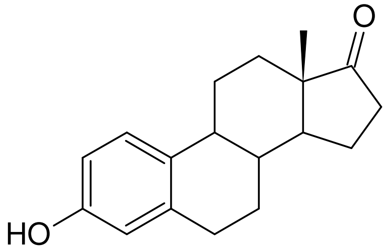
17β-Estradiol	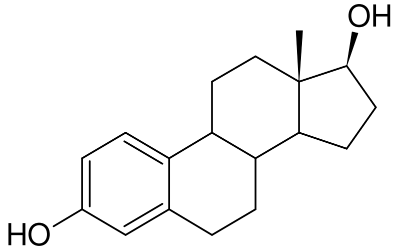
17α-Estradiol	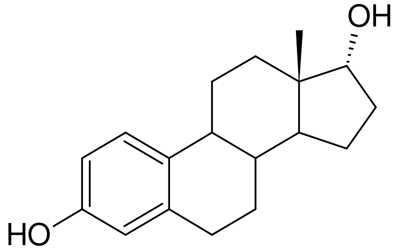
Estriol	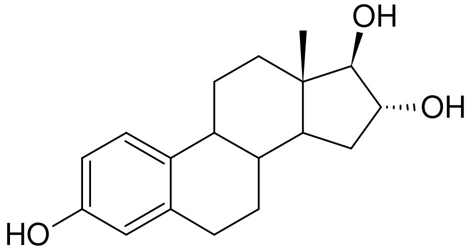
Ethinylestradiol	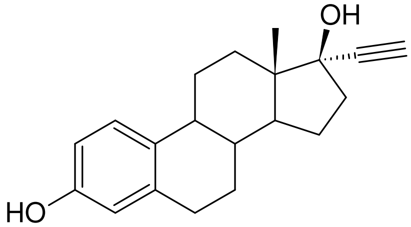
Estradiol acetate	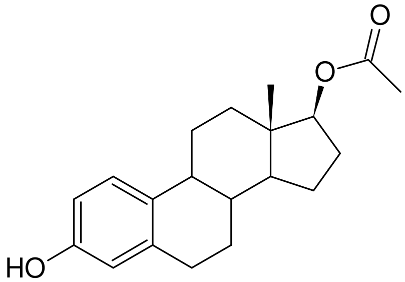
**Pregnanes**	

17α-Hydroxyprogesterone	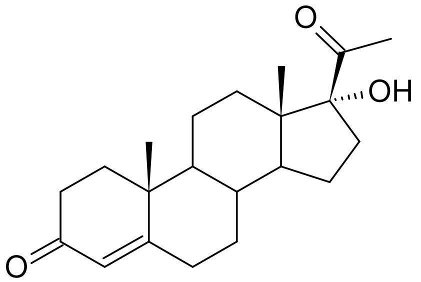
11α-Hydroxyprogesterone	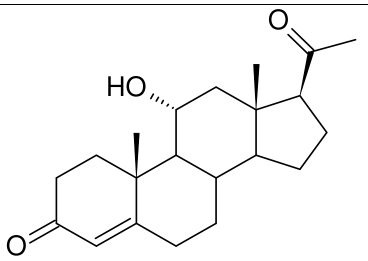
21-Hydroxyprogesterone	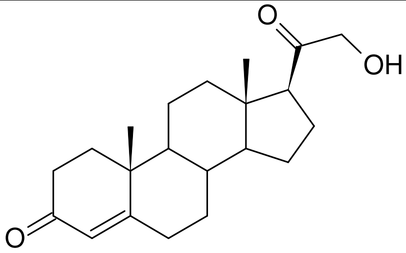
17α,21-Dihydroxyprogesterone	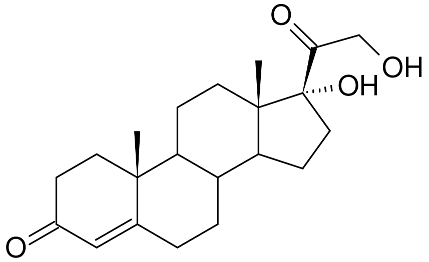
5α-Pregnan-3α-ol-20-one	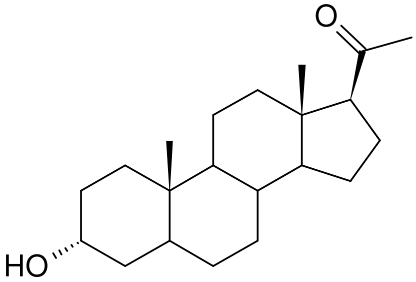
Allopregnanolone	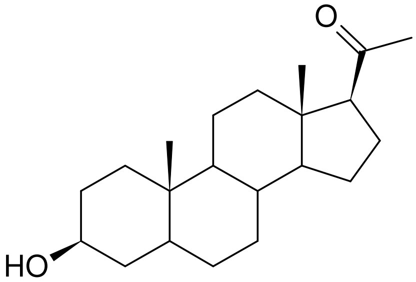
**Corticosteroids**	

Prednisone	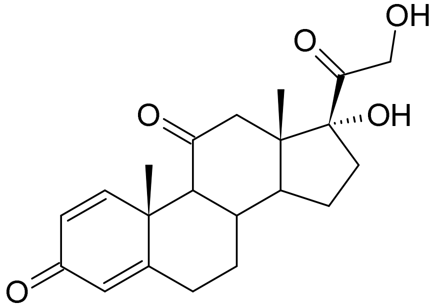
Prednisolone	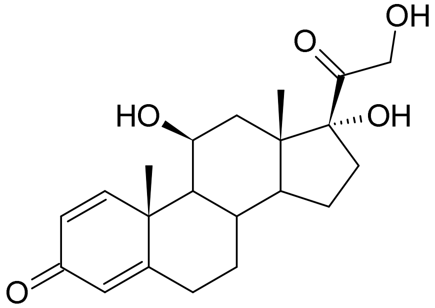
Dexamethasone	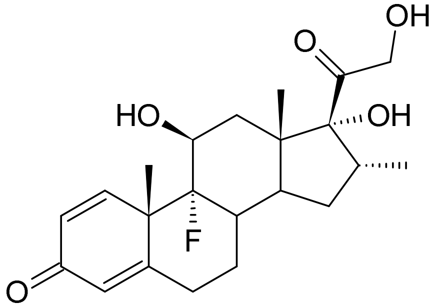
6α-Methylprednisolone	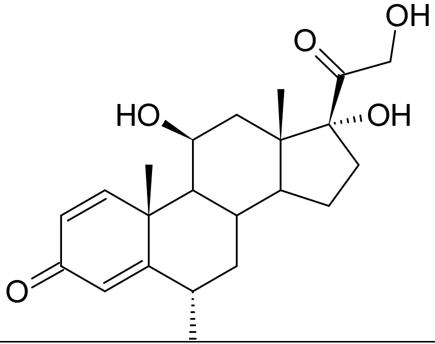
Betamethasone	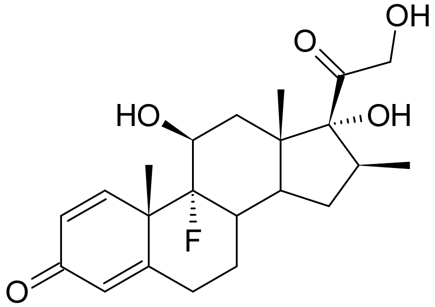

Clobetasol propionate	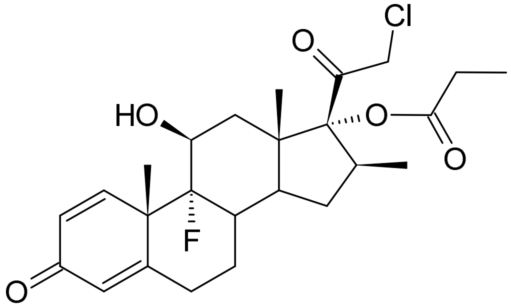
Mometasone furoate	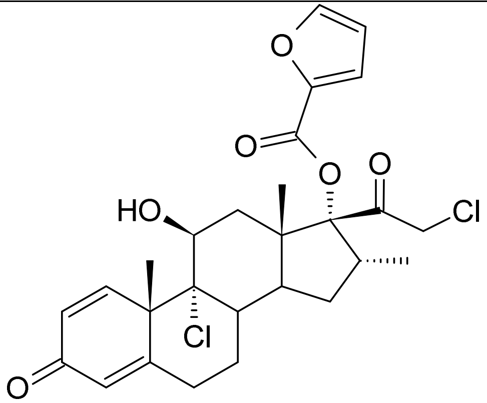

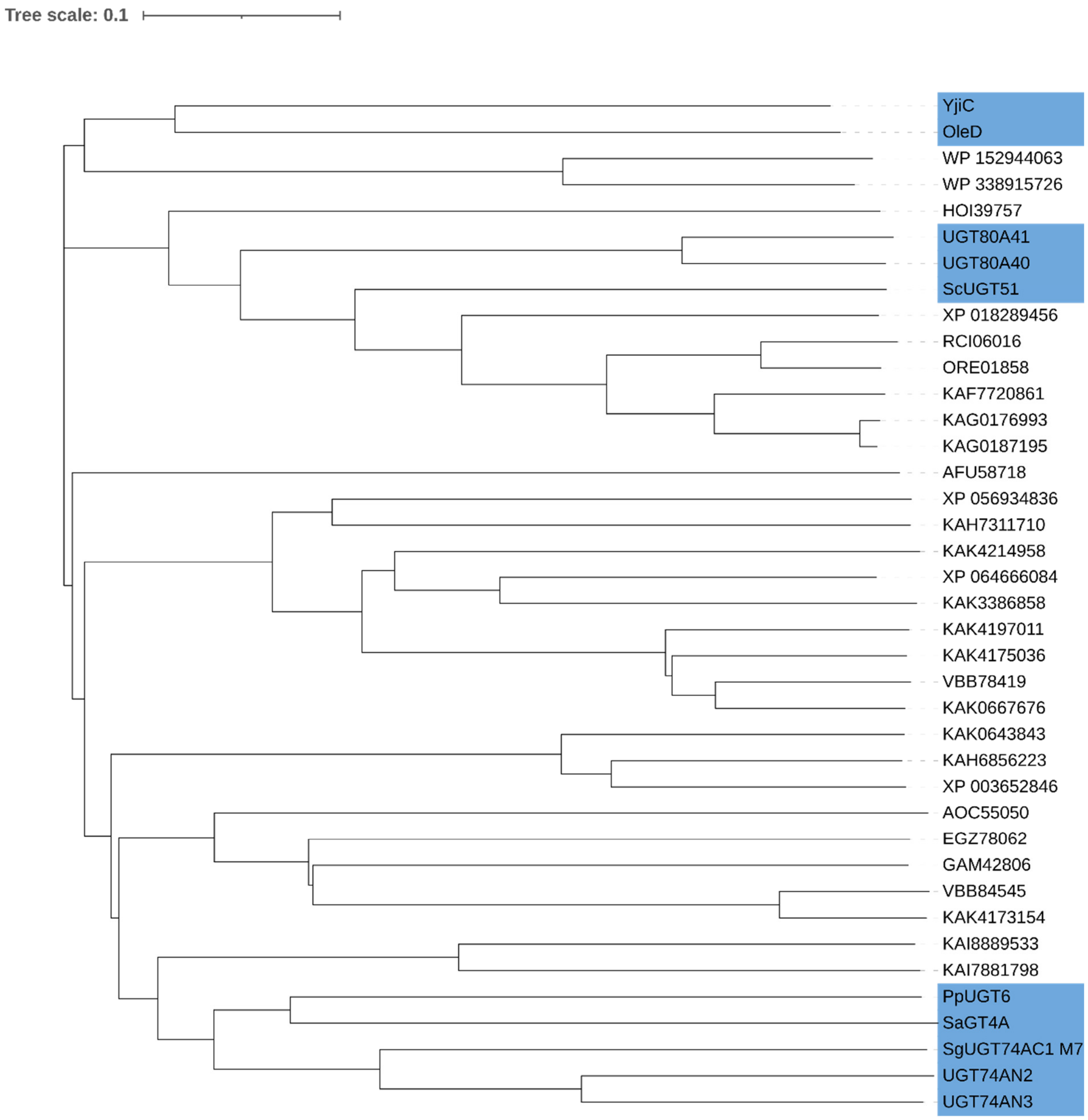

Figure S1 Cladogram of preselected sequences of potential steroid glucosyltransferases. Blue boxes mark input sequences. Multiple Sequence Alignment (MSA) was performed with the Clustal Omega algorithm (https://www.ebi.ac.uk/jdispatcher/msa/clustalo) to build cladogram using iTOL software (https://itol.embl.de/).

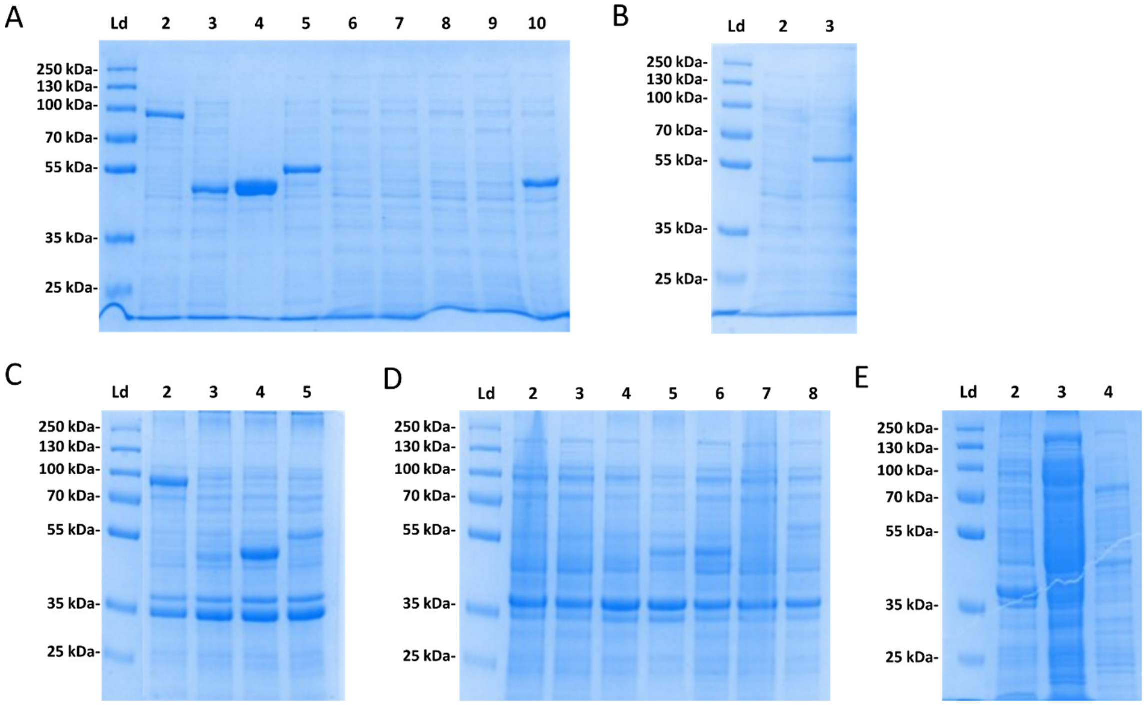

Figure S2. SDS-PAGE gels showing **A** purified on His-SpinTrap™ (Cytiva): Lane 2 – *Gm*SuSy; Lane 3 – YjiC; Lane 4 – OleD; Lane 5 – SbaiC7OGT; Lane 6 – *As*UGT; Lane 7 - *Pd*UGT1; Lane 8 – *Cng*UGT; Lane 9 – GtfC; Lane 10 - *Sg*UGT74C1_M7; **B** purified on His-SpinTrap™ (Cytiva): Lane 2 - *Sa*GT4A, Lane 3 - Bet5OGT; **C** cell pellets from induced *E. coli* strain expressing: Lane 2 – *Gm*SuSy; Lane 3 – YjiC; Lane 4 – OleD; Lane 5 – SbaiC7OGT; **D** cell pellets from induced *E. coli* strain expressing: Lane 2 – *As*UGT; Lane 3 - *Pd*UGT1; Lane 4 – *Cng*UGT; Lane 5 – GtfC; Line 6 - *Sg*UGT74C1_M7; Lane 7 - *Sa*GT4A, Lane 8 - Bet5OGT; **E** Lane 2 – cell pellet from induced *E. coli* strain expressing *Sc*UGT51; Lane 3 - cell lysate from induced *E. coli* strain expressing *Sc*UGT51; Lane 4 - purified on His-SpinTrap™ (Cytiva) *Sc*UGT51. Ld – PageRuler Plus Prestained Protein Ladder (Thermo Fisher Scientific Waltham, MA, USA). Proteins mass – *Gm*SuSy – 92.0 kDa, YjiC - 44.7 kDa, OleD - 45.3 kDa, Sbaic7OGT – 53.1 kDa, Bet5OGT – 55.2 kDa, GtfC - 51.9 kDa, *Sg*UGT74AC1_M7 - 51.3 kDa, *Sa*GT4A - 55.3 kDa, *Sc*UGT51 - 53.6 kDa, *As*UGT - 55.0 kDa, *Pd*UGT1 - 50.1 kDa, *Cng*UGT - 40.5 kDa.

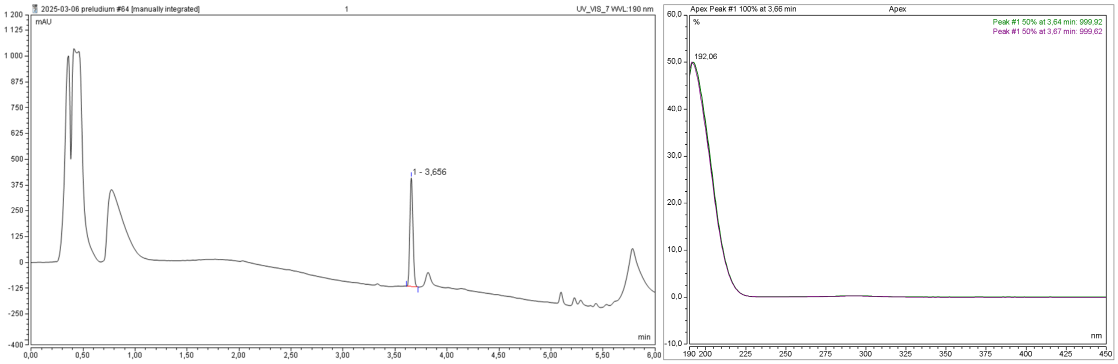

Figure S3. UPLC chromatogram and UV-Vis spectrum of DHEA.

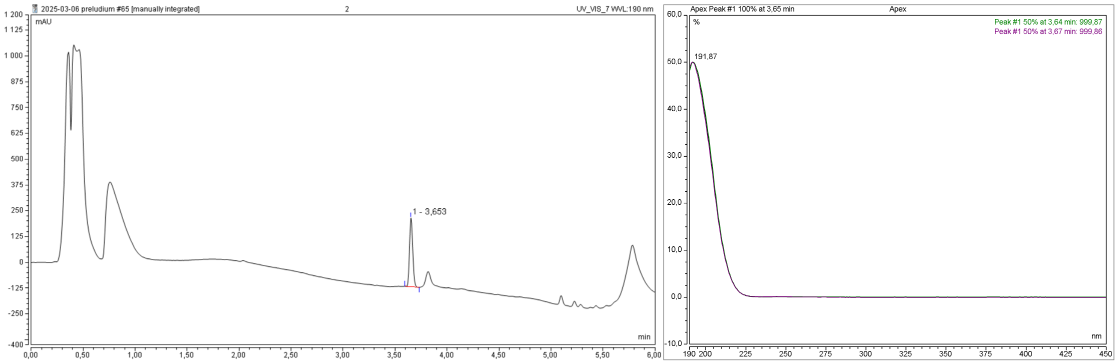

Figure S4. UPLC chromatogram and UV-Vis spectrum of Androstendiol.

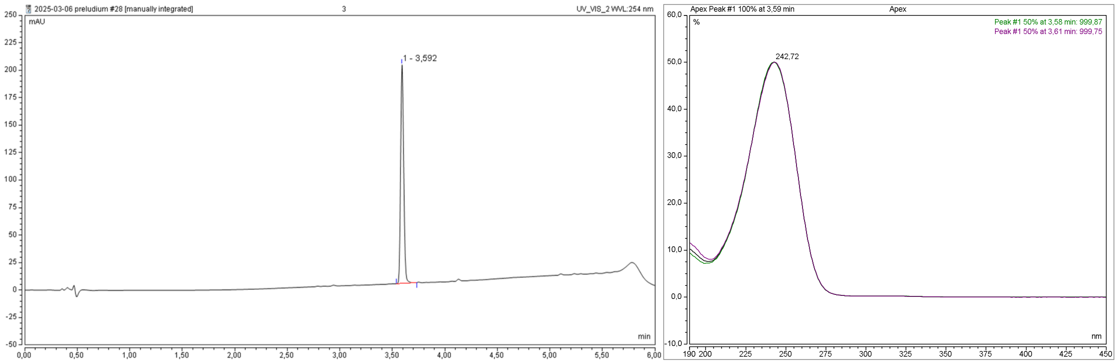

Figure S5. UPLC chromatogram and UV-Vis spectrum of 17α-Methyltestosterone.

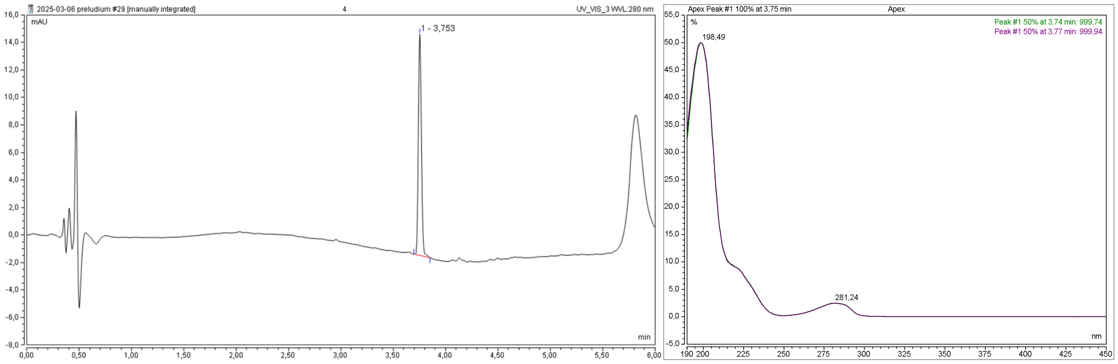

Figure S6. UPLC chromatogram and UV-Vis spectrum of Estrone.

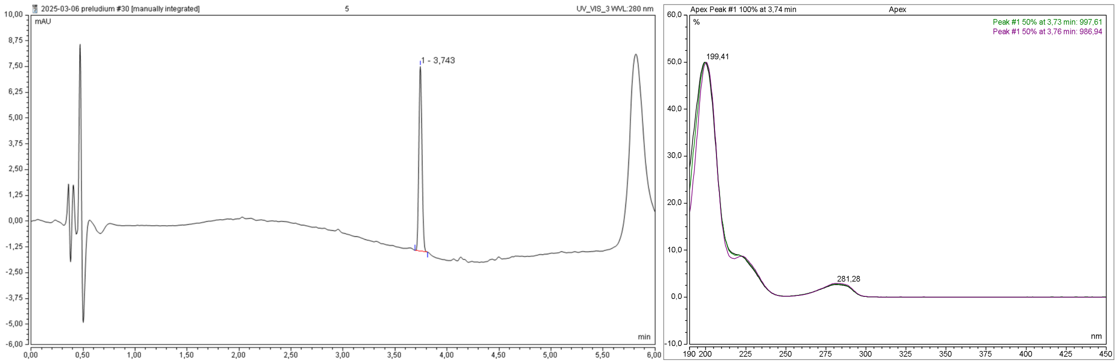

Figure S7. UPLC chromatogram and UV-Vis spectrum of 17β-Estradiol.

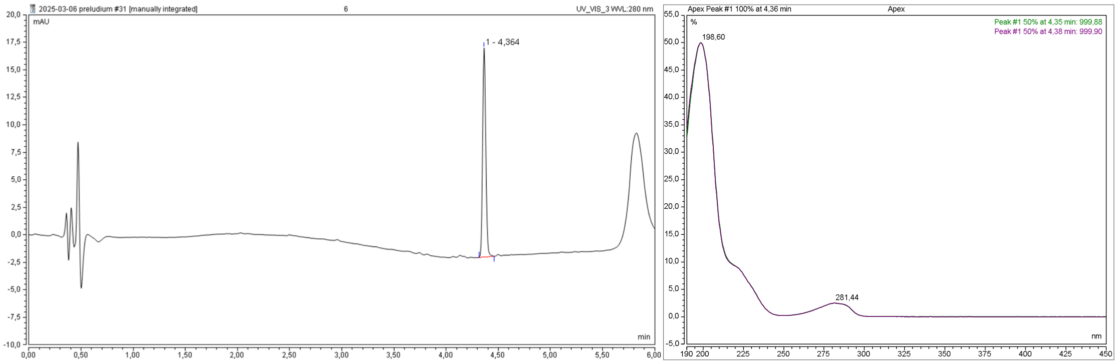

Figure S8. UPLC chromatogram and UV-Vis spectrum of Estradiol acetate.

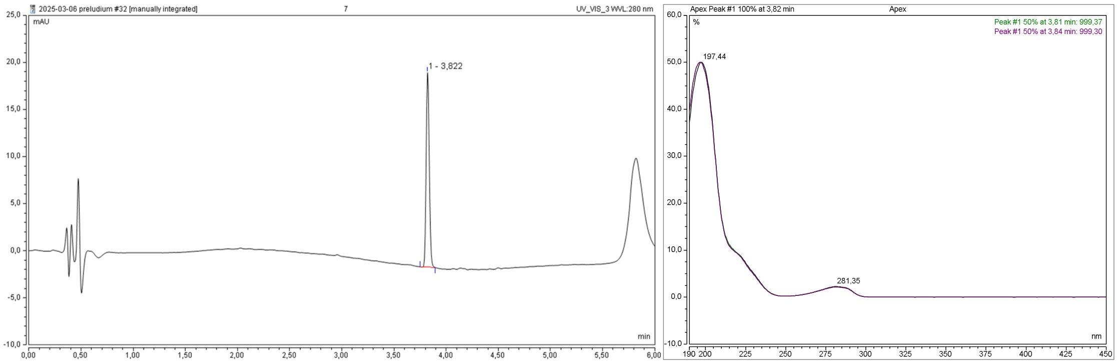

Figure S9. UPLC chromatogram and UV-Vis spectrum of Ethinylestradiol.

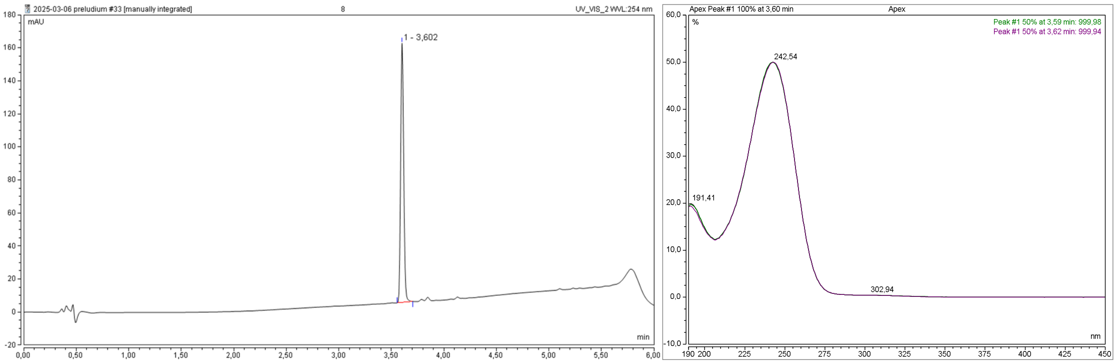

Figure S10. UPLC chromatogram and UV-Vis spectrum of 17α-Hydroxyprogesterone.

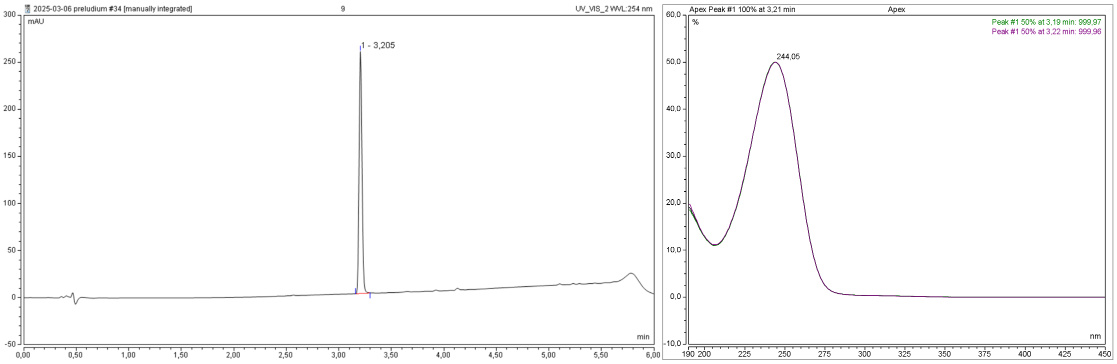

Figure S11. UPLC chromatogram and UV-Vis spectrum of 11α-Hydroxyprogesterone.

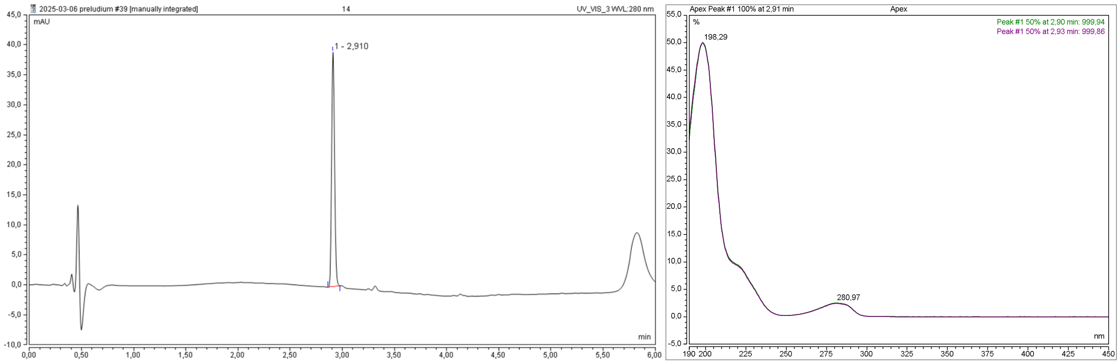

Figure S12. UPLC chromatogram and UV-Vis spectrum of Estriol.

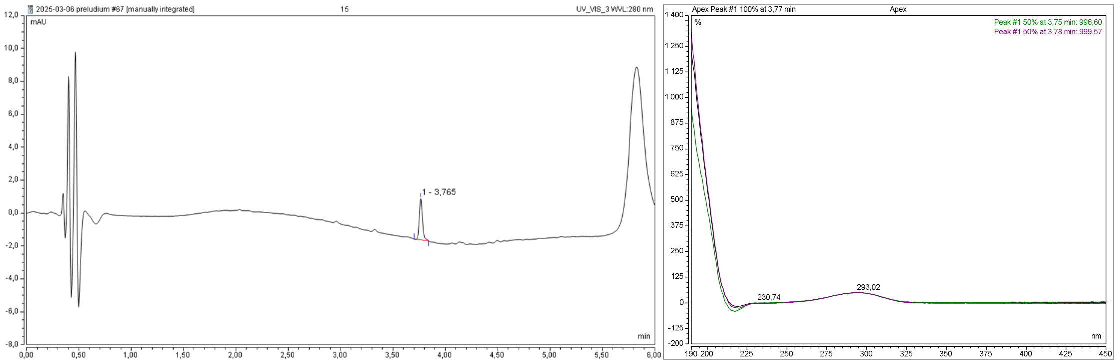

Figure S13. UPLC chromatogram and UV-Vis spectrum of *trans*-Androsterone.

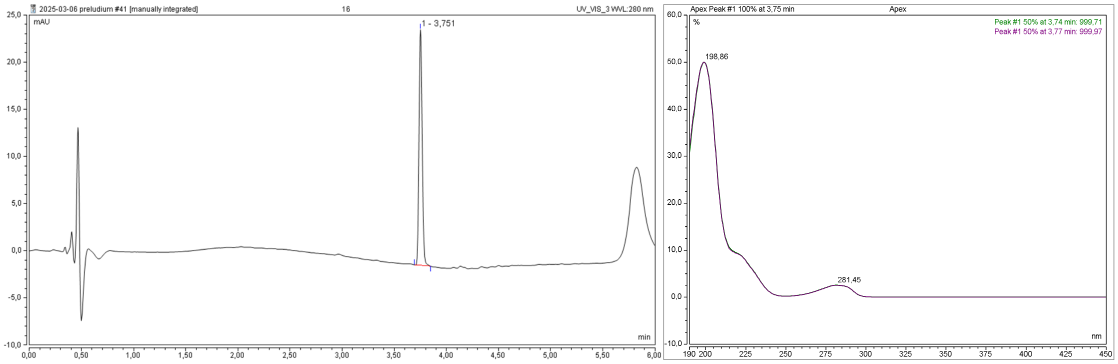

Figure S14. UPLC chromatogram and UV-Vis spectrum of 17α-Estradiol.

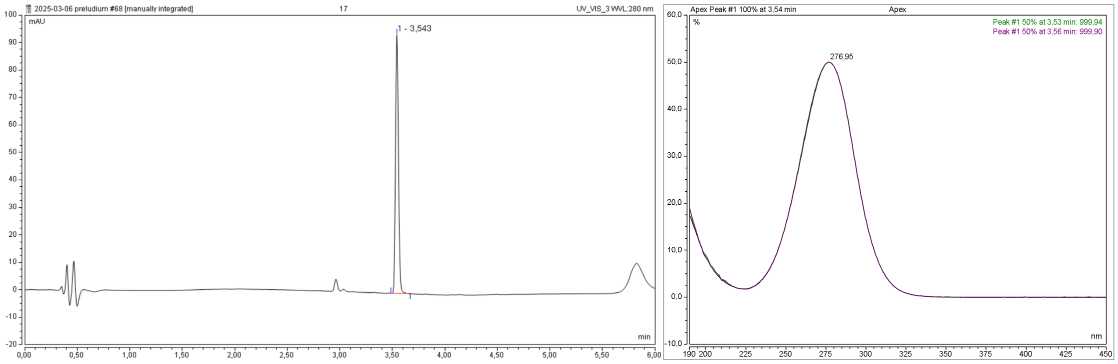

Figure S15. UPLC chromatogram and UV-Vis spectrum of Formestane.

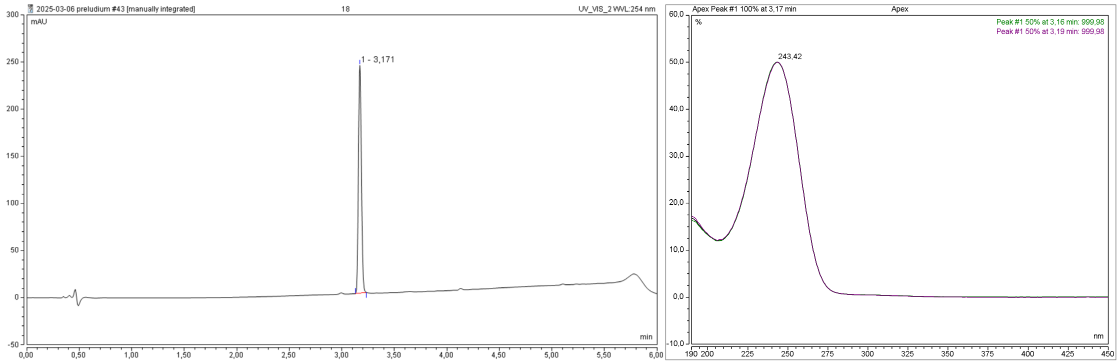

Figure S16. UPLC chromatogram and UV-Vis spectrum of 17α,21-Dihydroxyprogesterone.

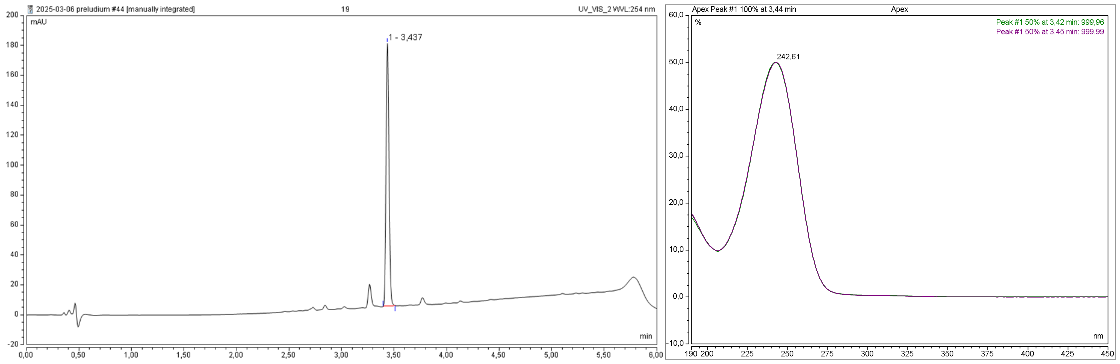

Figure S17. UPLC chromatogram and UV-Vis spectrum of 21-Hydroxyprogesterone.

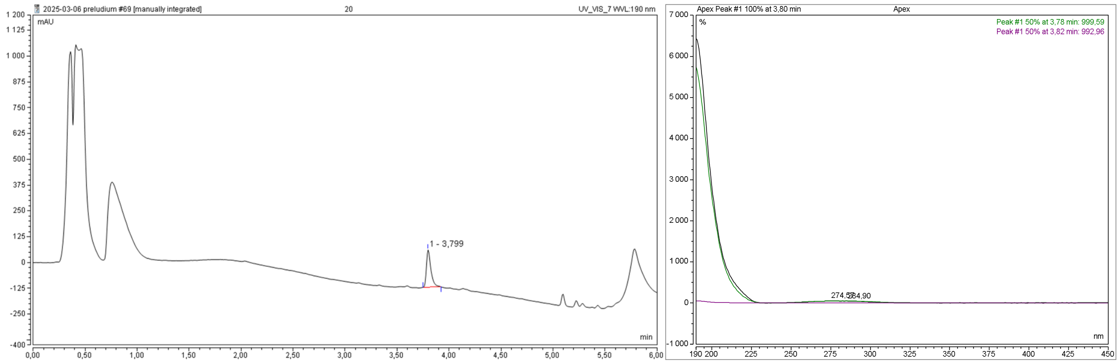

Figure S18. UPLC chromatogram and UV-Vis spectrum of 5α-Androstan-17β-ol-3-one.

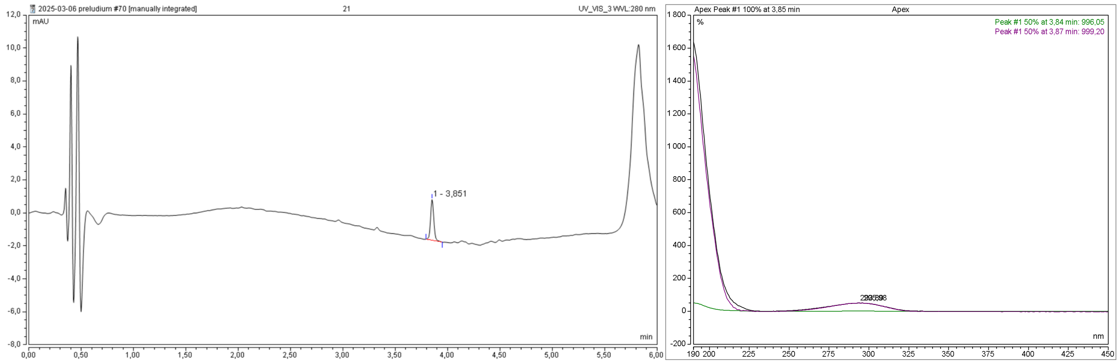

Figure S19. UPLC chromatogram and UV-Vis spectrum of 3α-Hydroxy-17-androstanone.

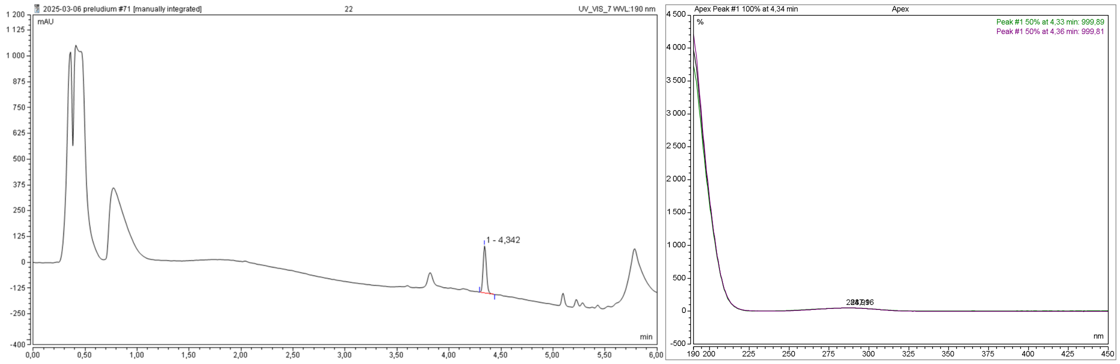

Figure S20. UPLC chromatogram and UV-Vis spectrum of 5α-Pregnan-3α-ol-20-one.

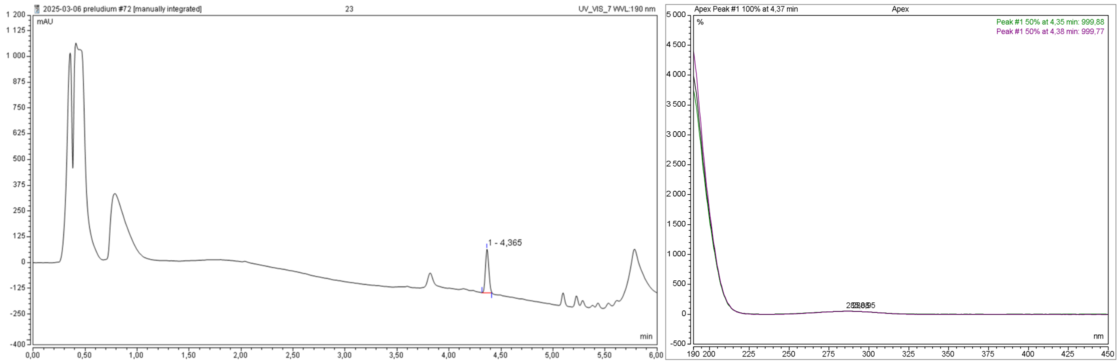

Figure S21. UPLC chromatogram and UV-Vis spectrum of Allopregnanolone.

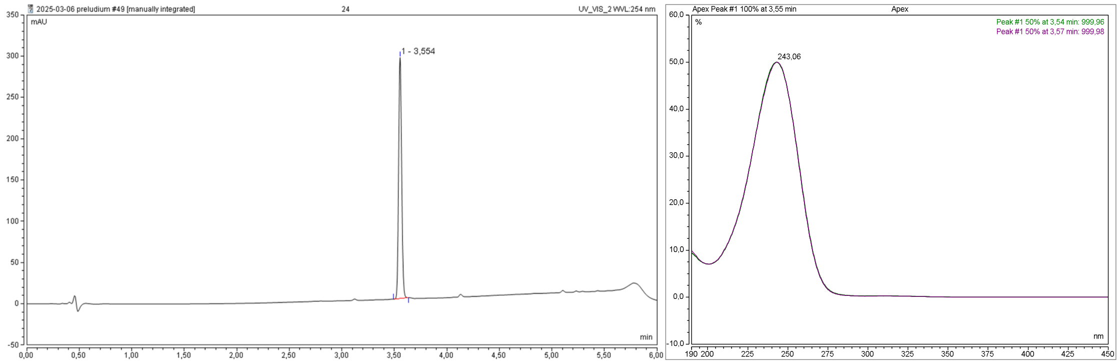

Figure S22. UPLC chromatogram and UV-Vis spectrum of 17α-Testosterone.

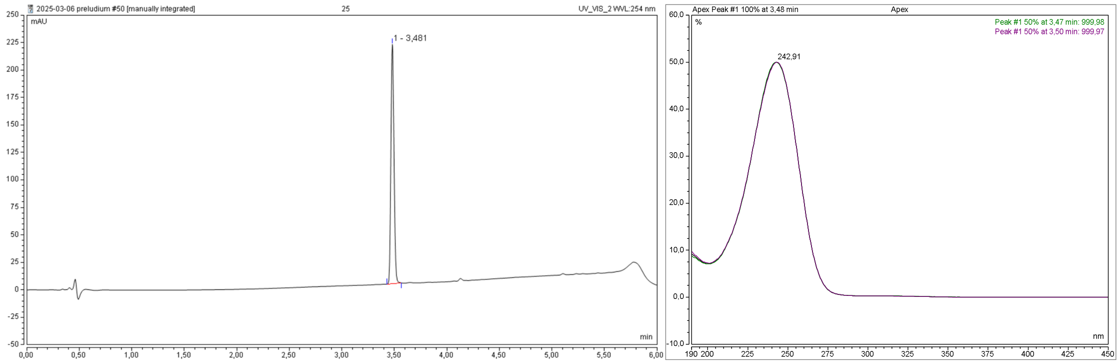

Figure S23. UPLC chromatogram and UV-Vis spectrum of Testosterone.

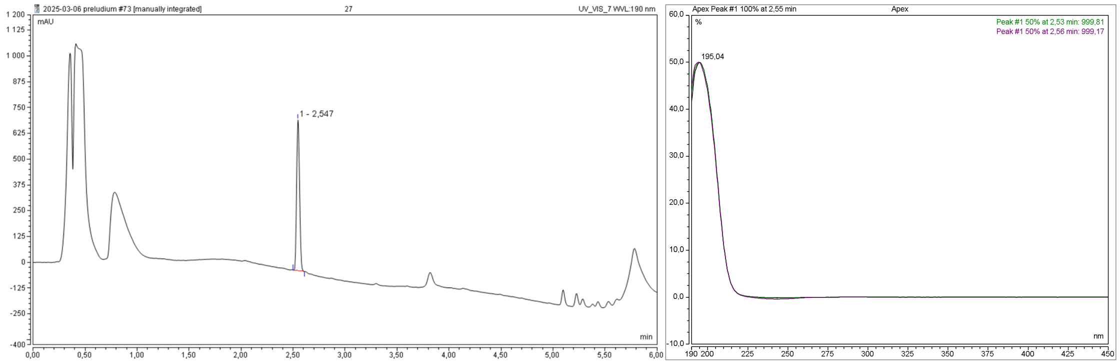

Figure S24. UPLC chromatogram and UV-Vis spectrum of 7α-Lacto DHEA.

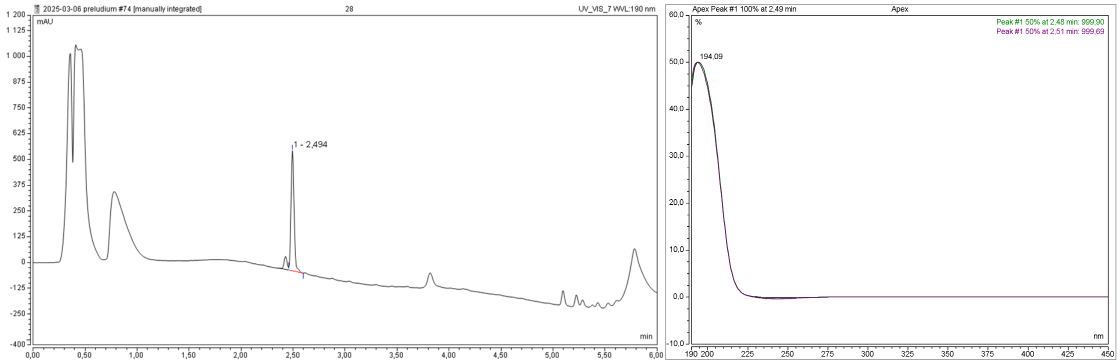

Figure S25. UPLC chromatogram and UV-Vis spectrum of 7β-Lacto DHEA.

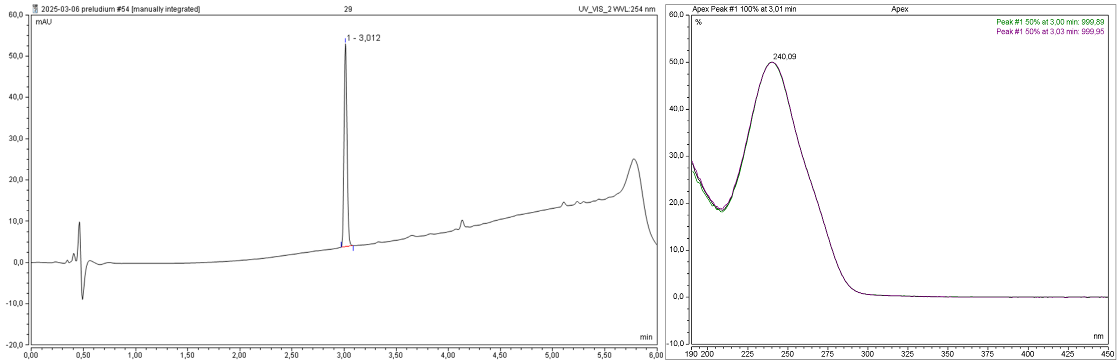

Figure S26. UPLC chromatogram and UV-Vis spectrum of Dexamethasone.

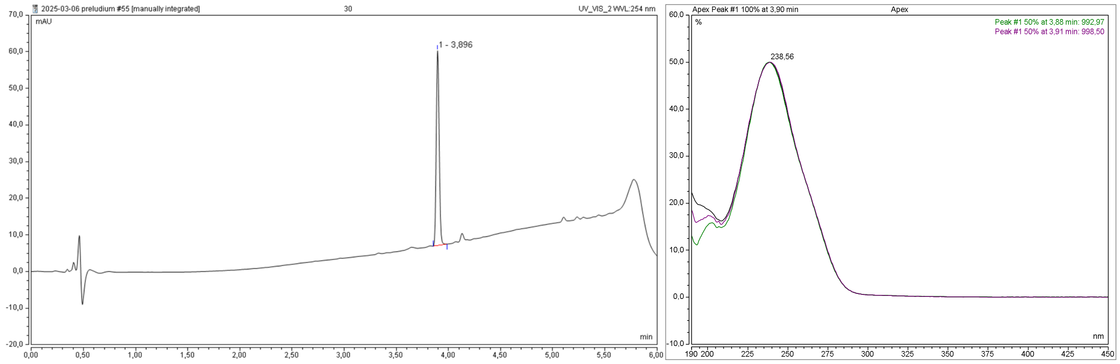

Figure S27. UPLC chromatogram and UV-Vis spectrum of Clobetasol propionate.

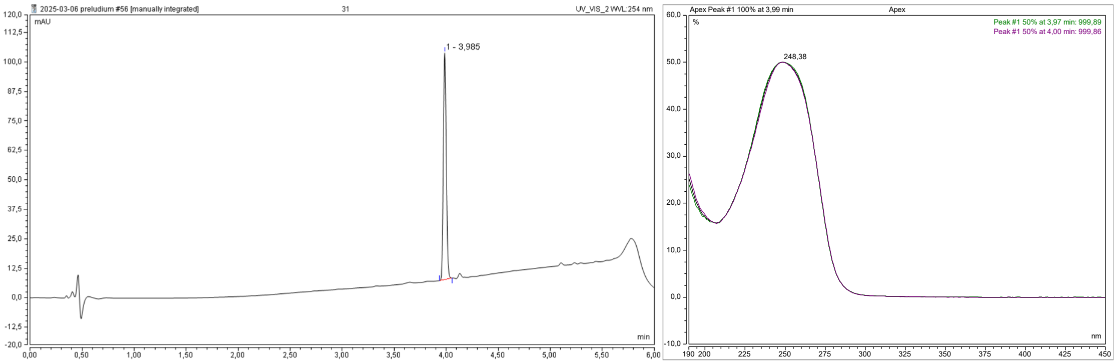

Figure S28. UPLC chromatogram and UV-Vis spectrum of Mometasone furoate

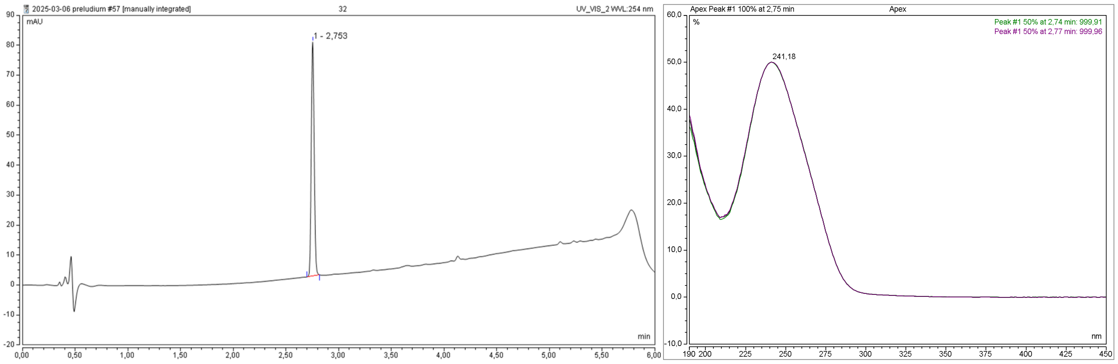

Figure S29. UPLC chromatogram and UV-Vis spectrum of Prednisone.

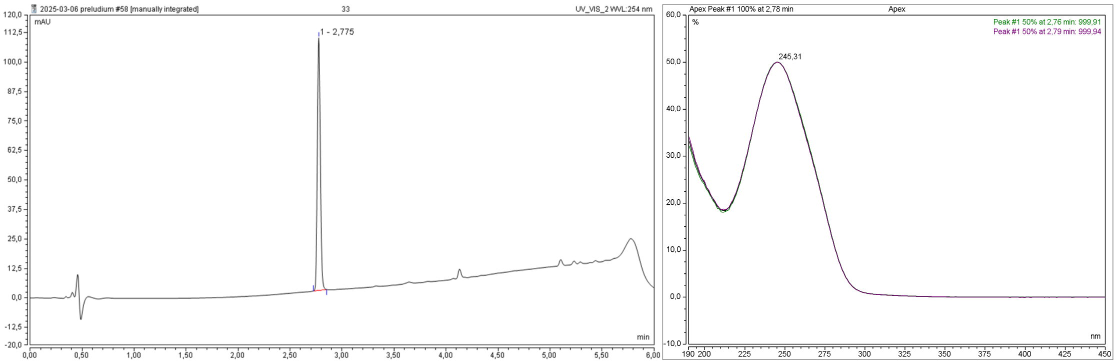

Figure S30. UPLC chromatogram and UV-Vis spectrum of Prednisolone.

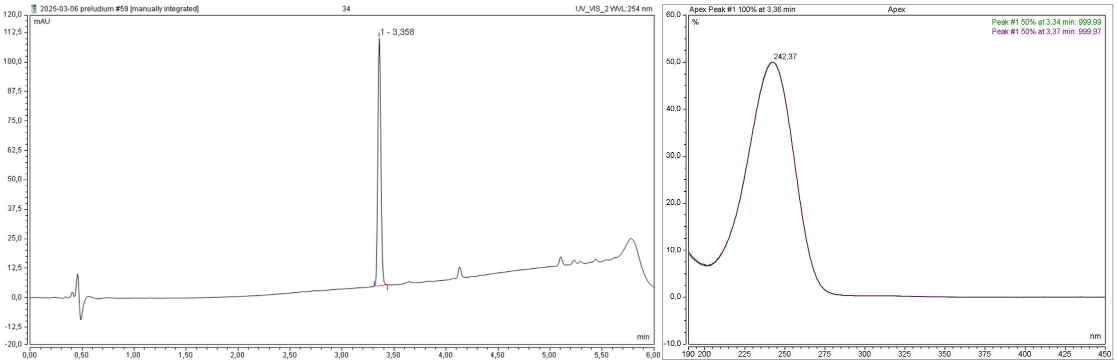

Figure S31. UPLC chromatogram and UV-Vis spectrum of Nandrolone.

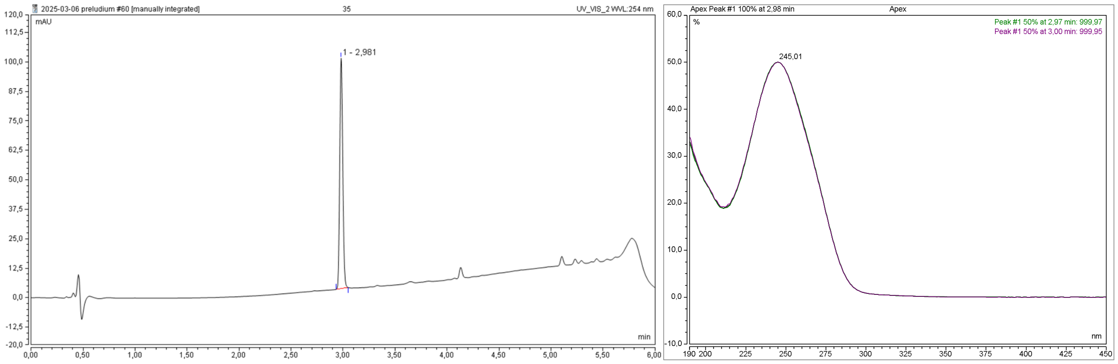

Figure S32. UPLC chromatogram and UV-Vis spectrum of 6α-Methylprednisolone.

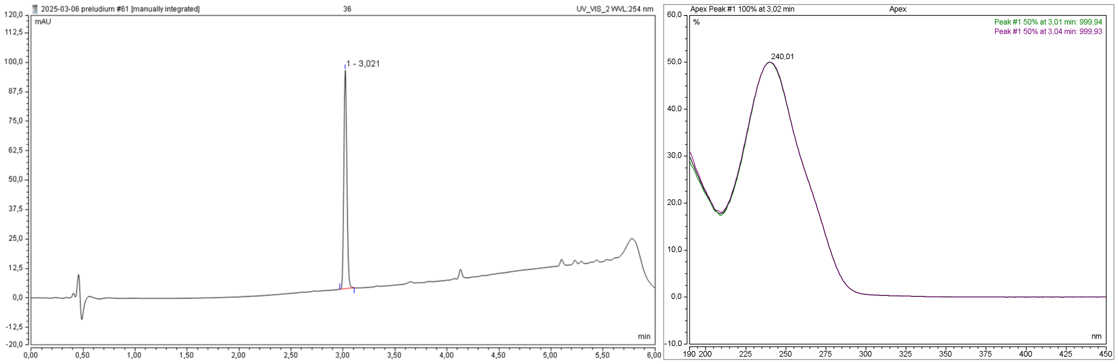

Figure S33. UPLC chromatogram and UV-Vis spectrum of Betamethasone.

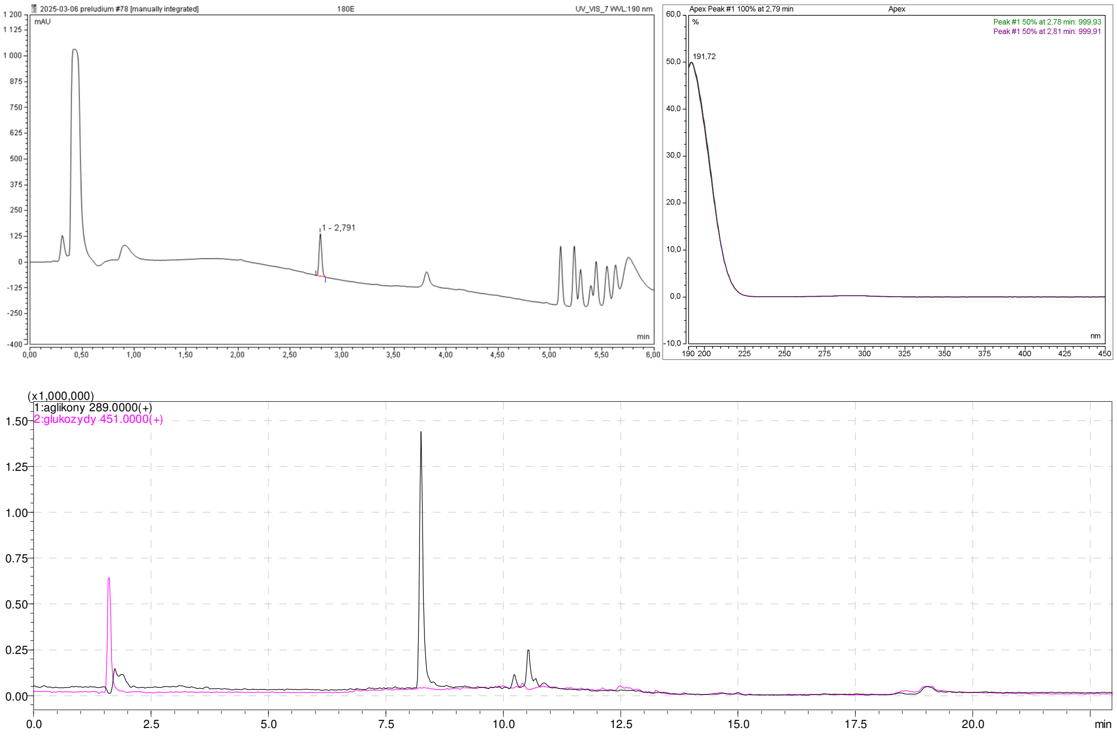

Figure S34. UPLC-DAD and LC-MS analysis of glucosylation reaction of DHEA by YjiC GT.

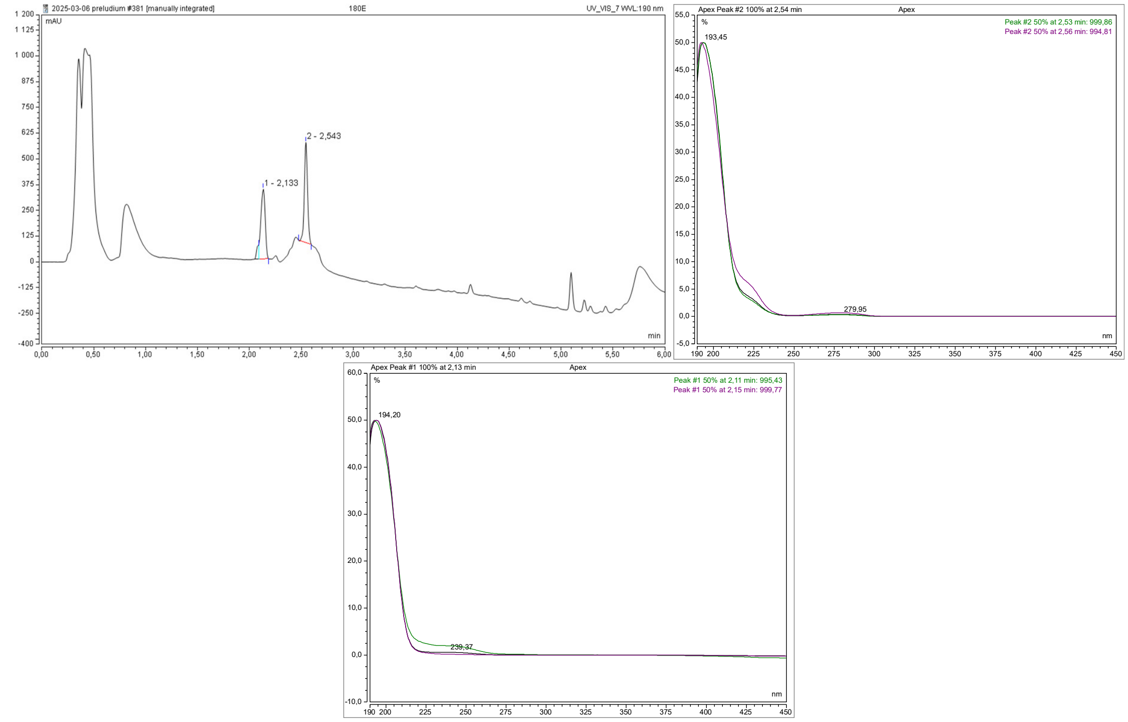

Figure S35. UPLC-DAD and LC-MS analysis of glucosylation reaction of 7α-Lacto DHEA by YjiC GT.

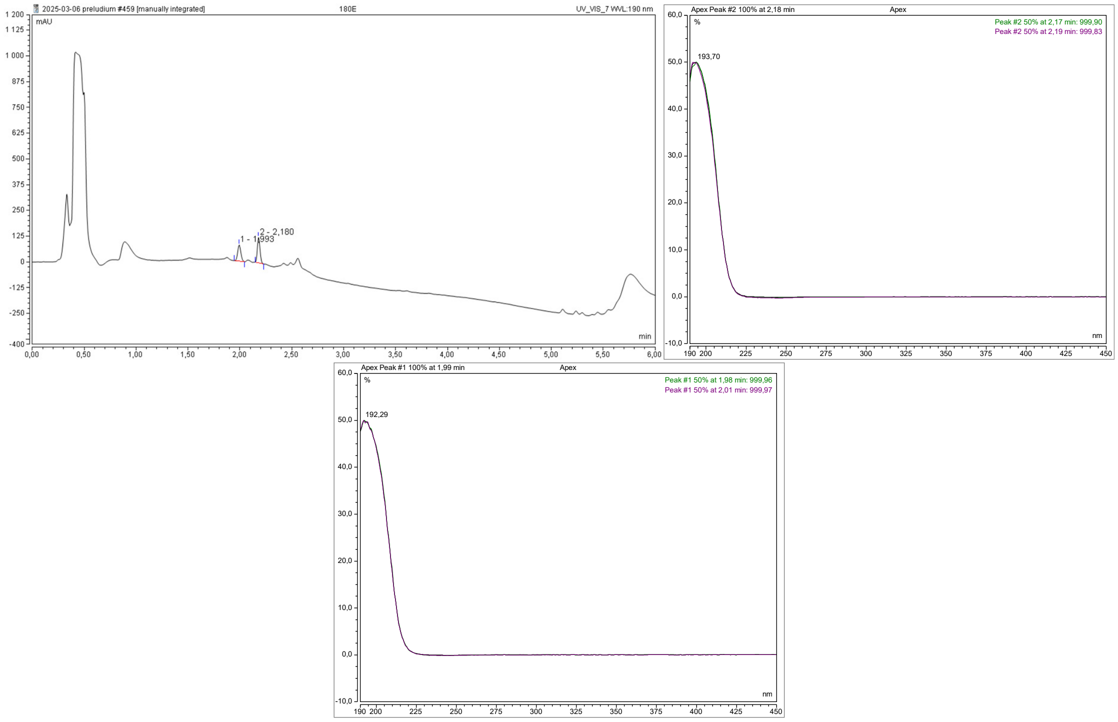

Figure S36. UPLC-DAD and LC-MS analysis of glucosylation reaction of 7β-Lacto DHEA by YjiC GT. Product proportions: 43.2:56.8%.

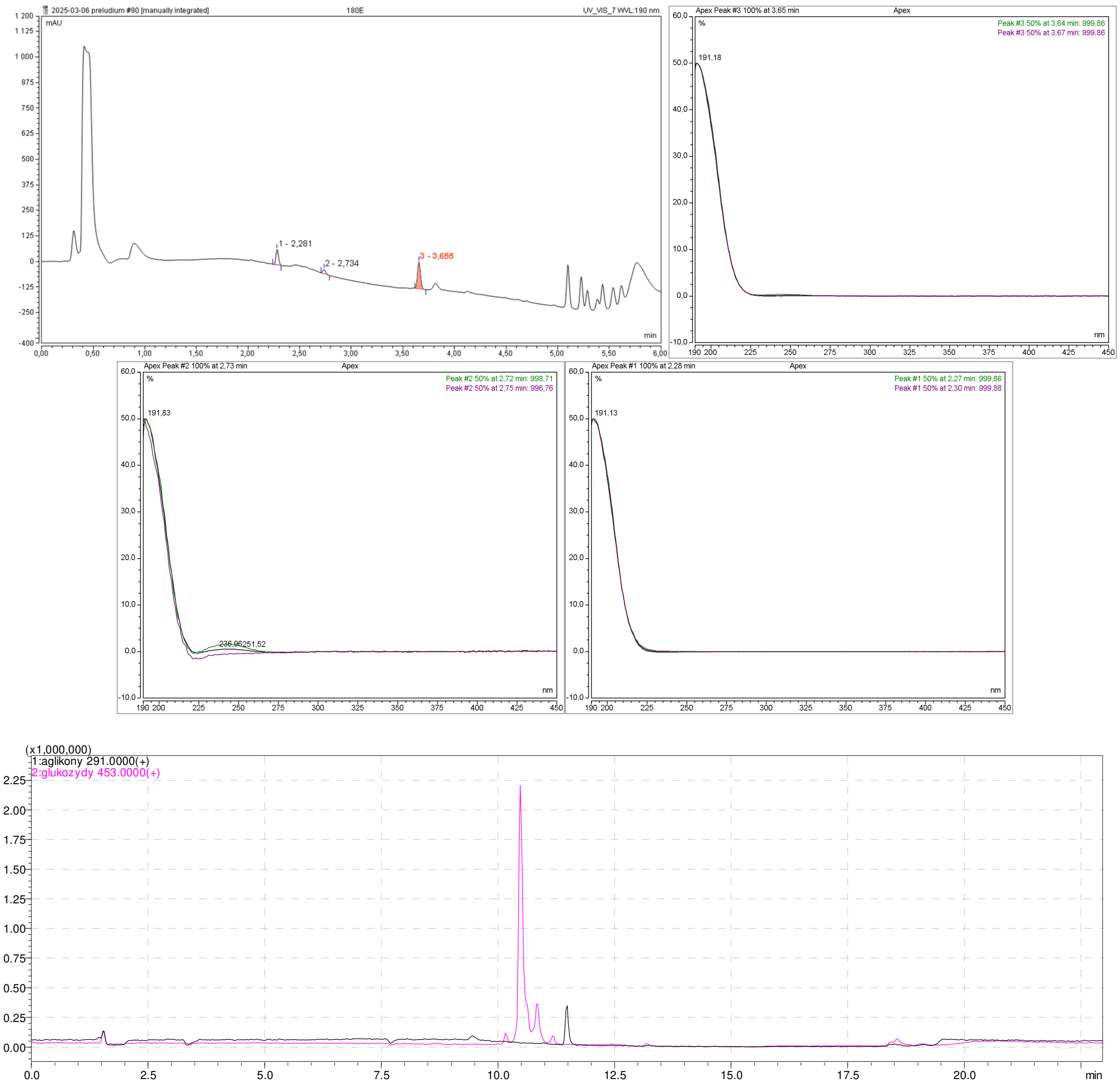

Figure S37. UPLC-DAD and LC-MS analysis of glucosylation of Androstendiol by YjiC GT. Product proportions – 76.0:24.0%.

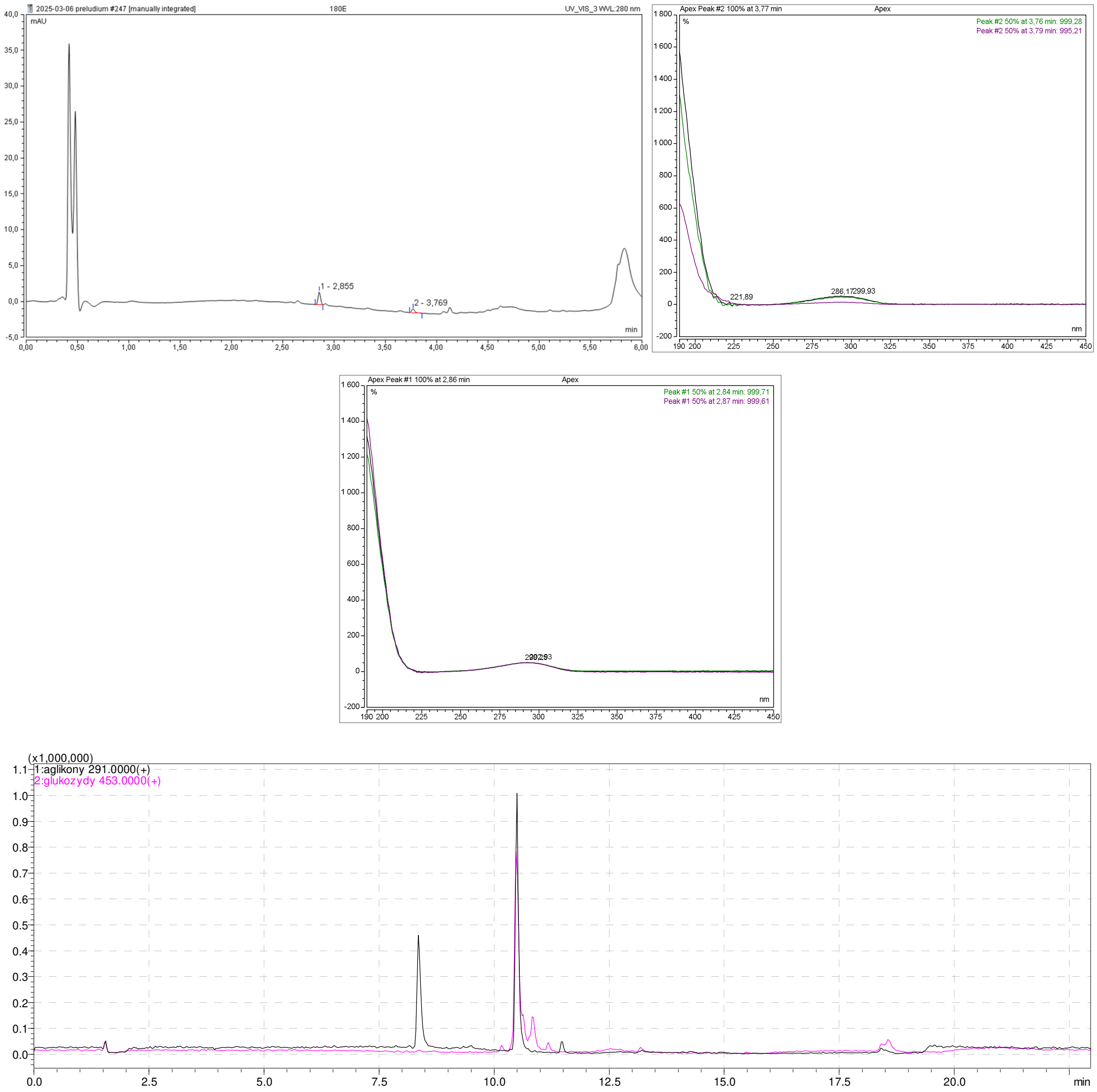

Figure S38. UPLC-DAD and LC-MS analysis of glucosylation of *trans*-Androsterone by YjiC GT.

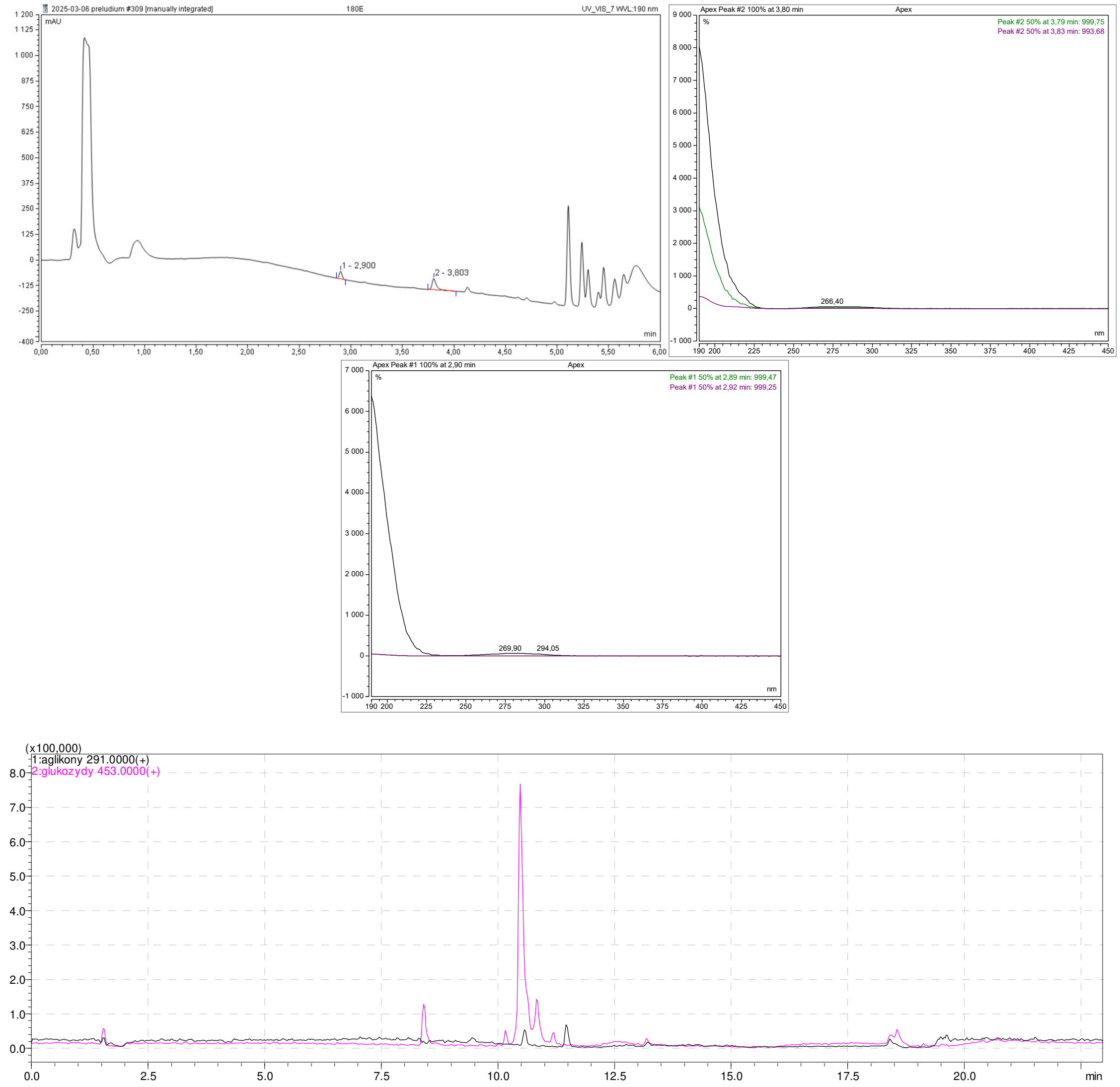

Figure S39. UPLC-DAD and LC-MS analysis of glucosylation of 5α-Androstan-17β-ol-3-one by YjiC GT.

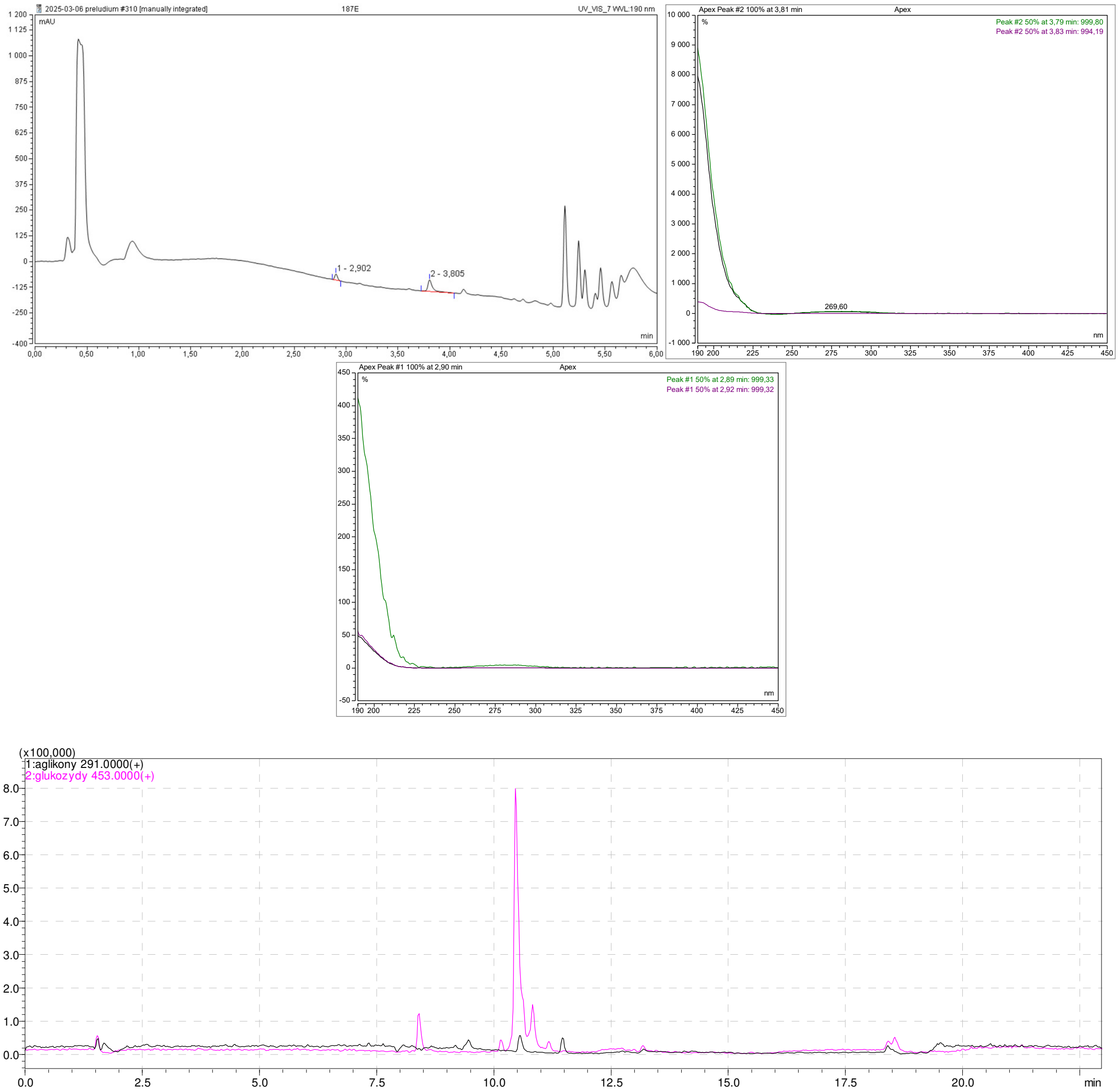

Figure S40. UPLC-DAD and LC-MS analysis of glucosylation of 5α-Androstan-17β-ol-3-one by OleD GT.

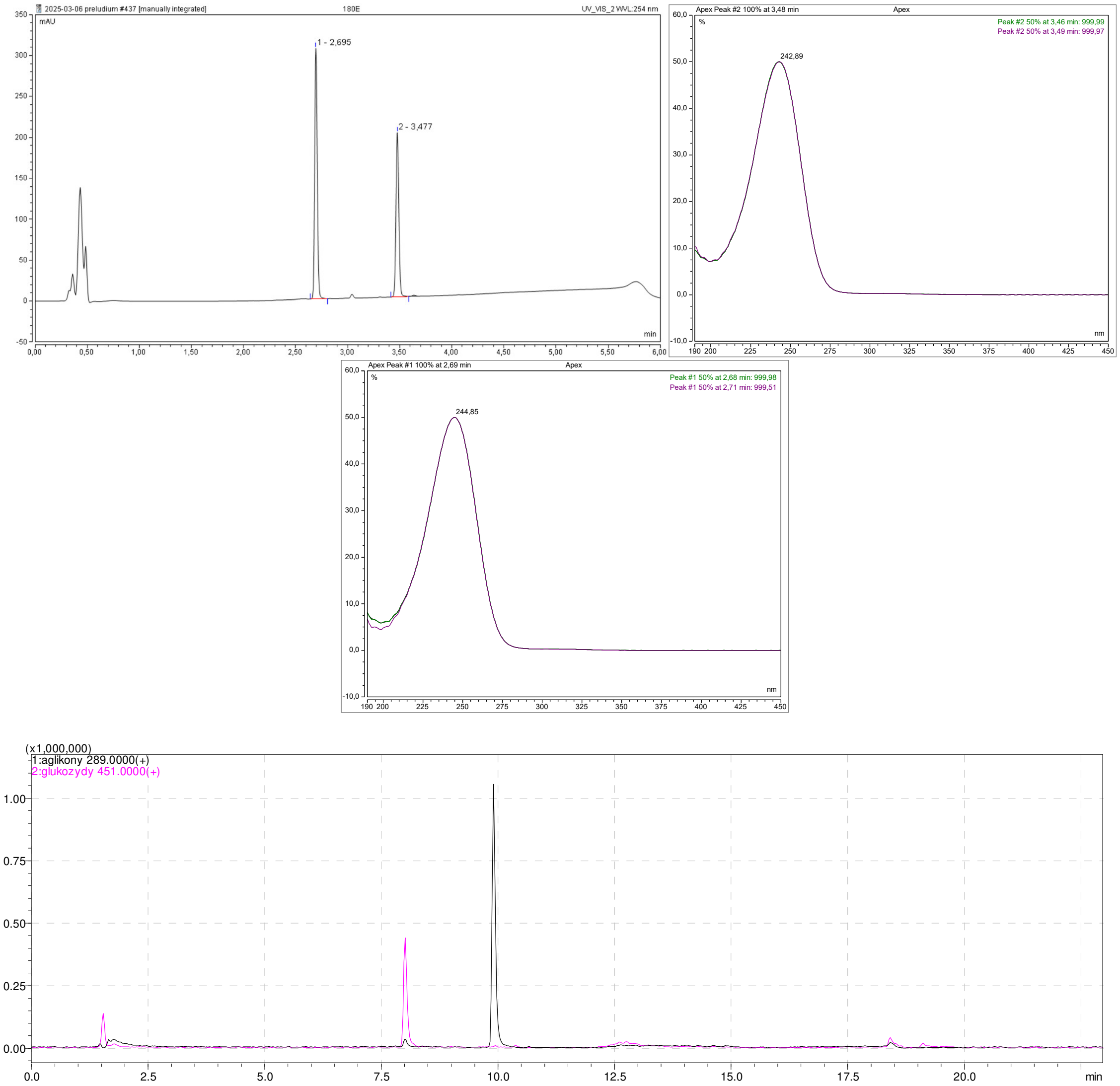

Figure S41. UPLC-DAD and LC-MS analysis of glucosylation of Testosterone by YjiC GT.

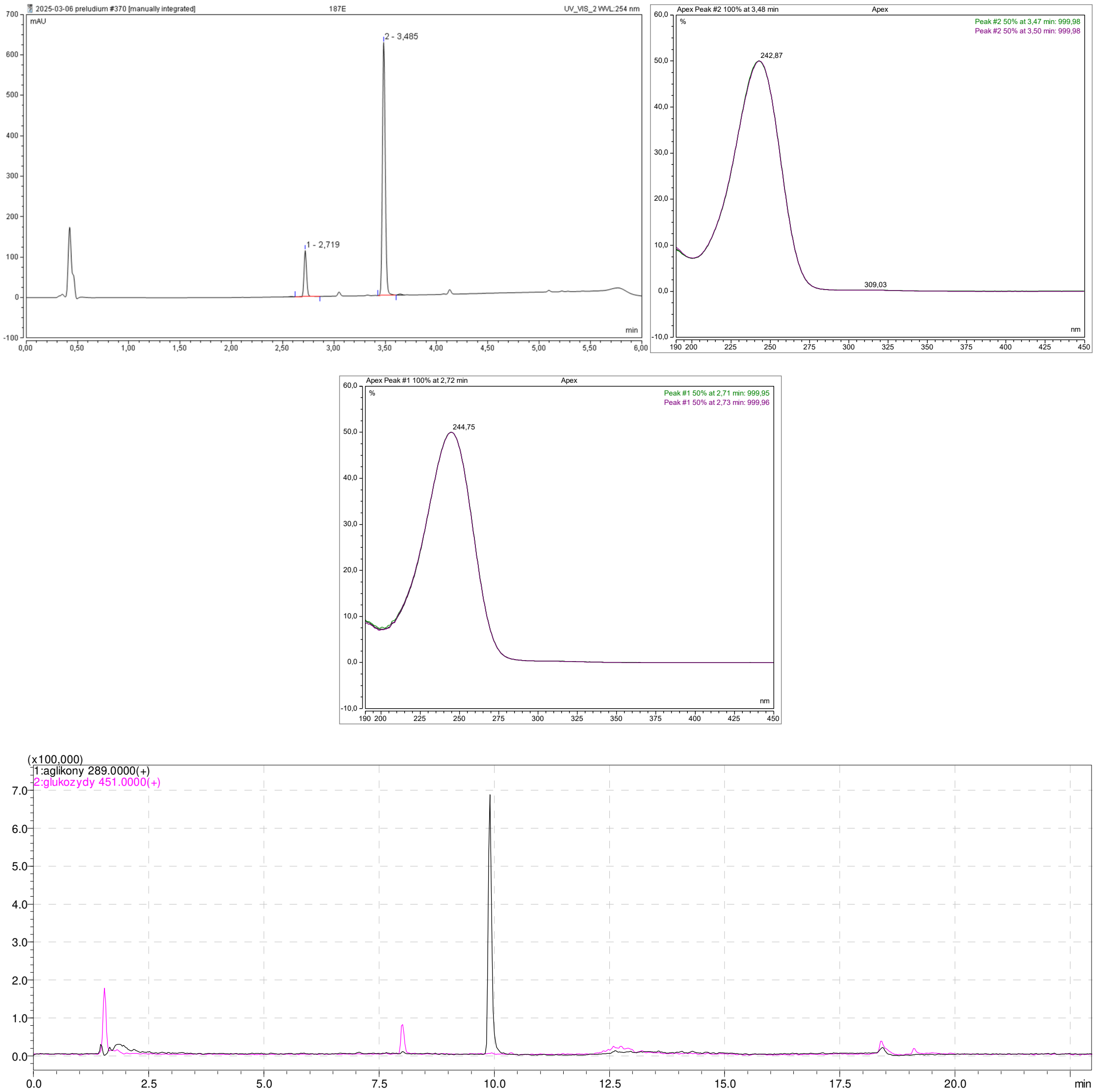

Figure S42. UPLC-DAD and LC-MS analysis of glucosylation of Testosterone by OleD GT.

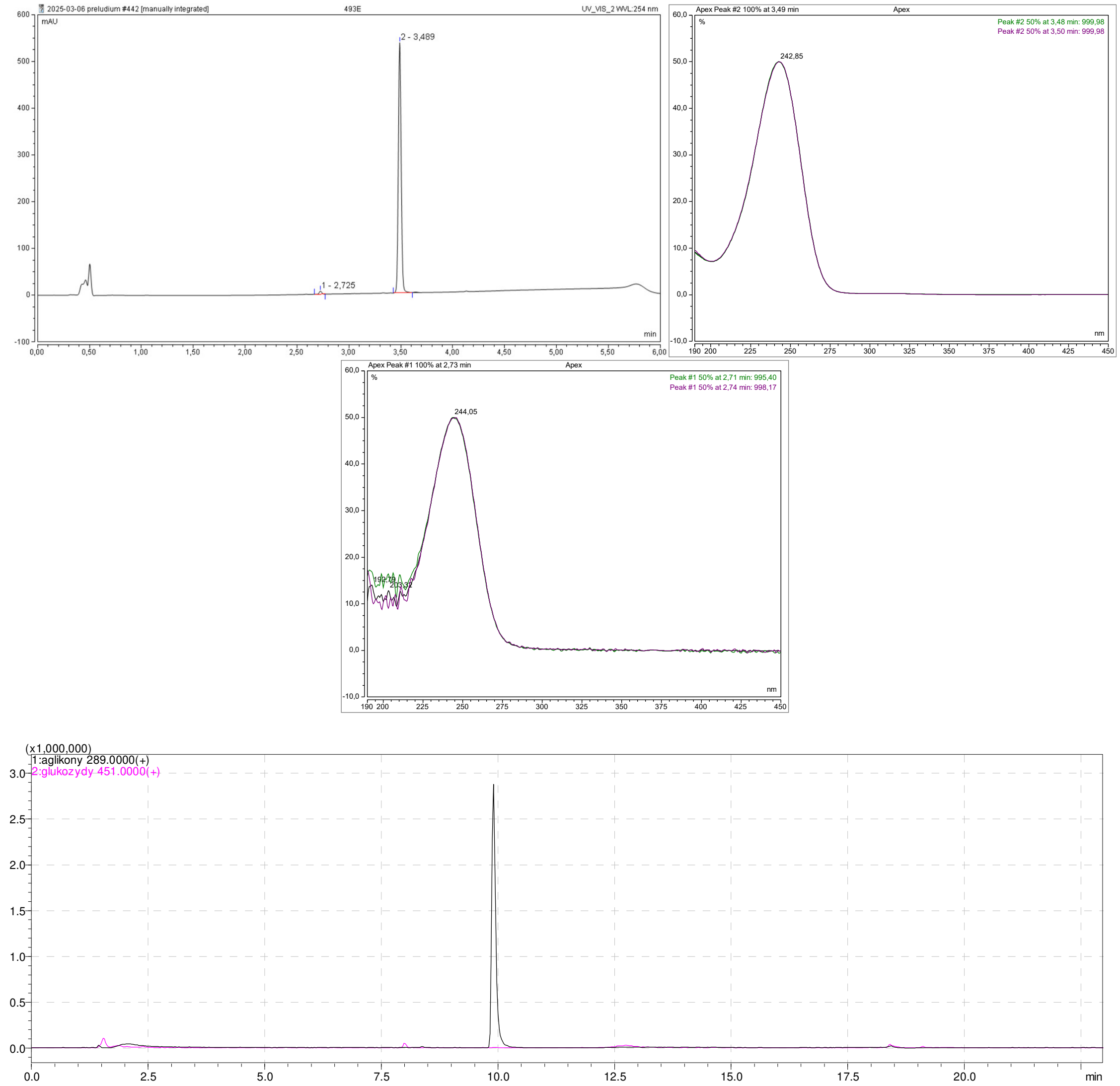

Figure S43. UPLC-DAD and LC-MS analysis of glucosylation of Testosterone by SgUGT74AC1_M7 GT.

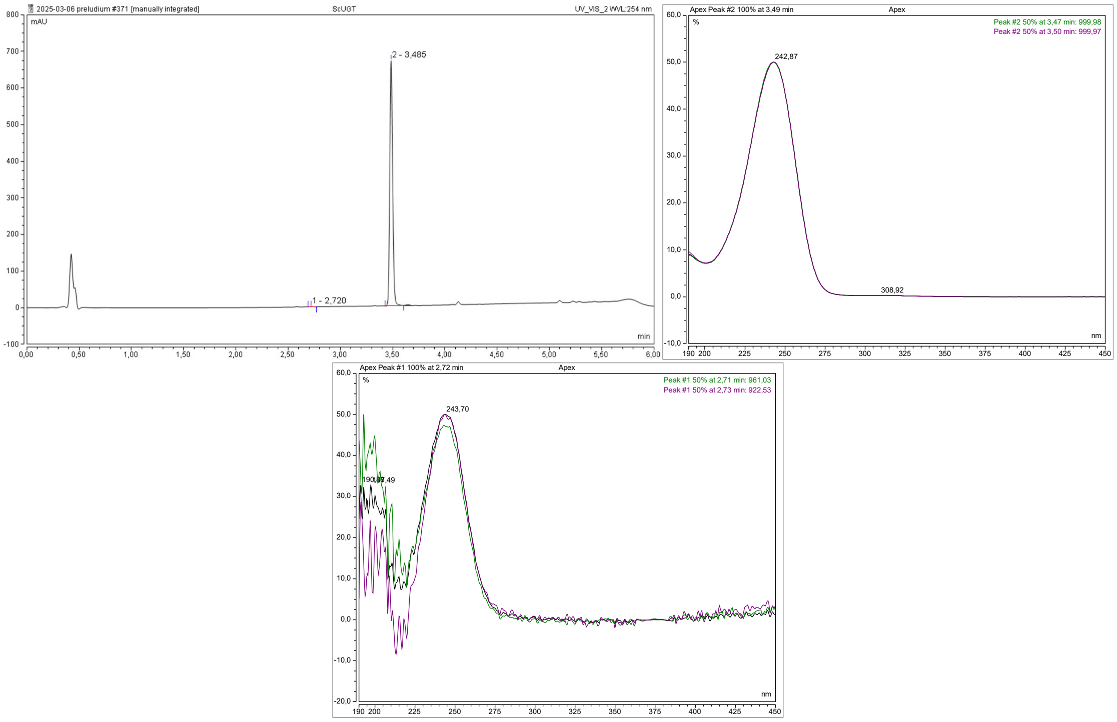

Figure S44. UPLC-DAD and LC-MS analysis of glucosylation of Testosterone by ScUGT51 GT.

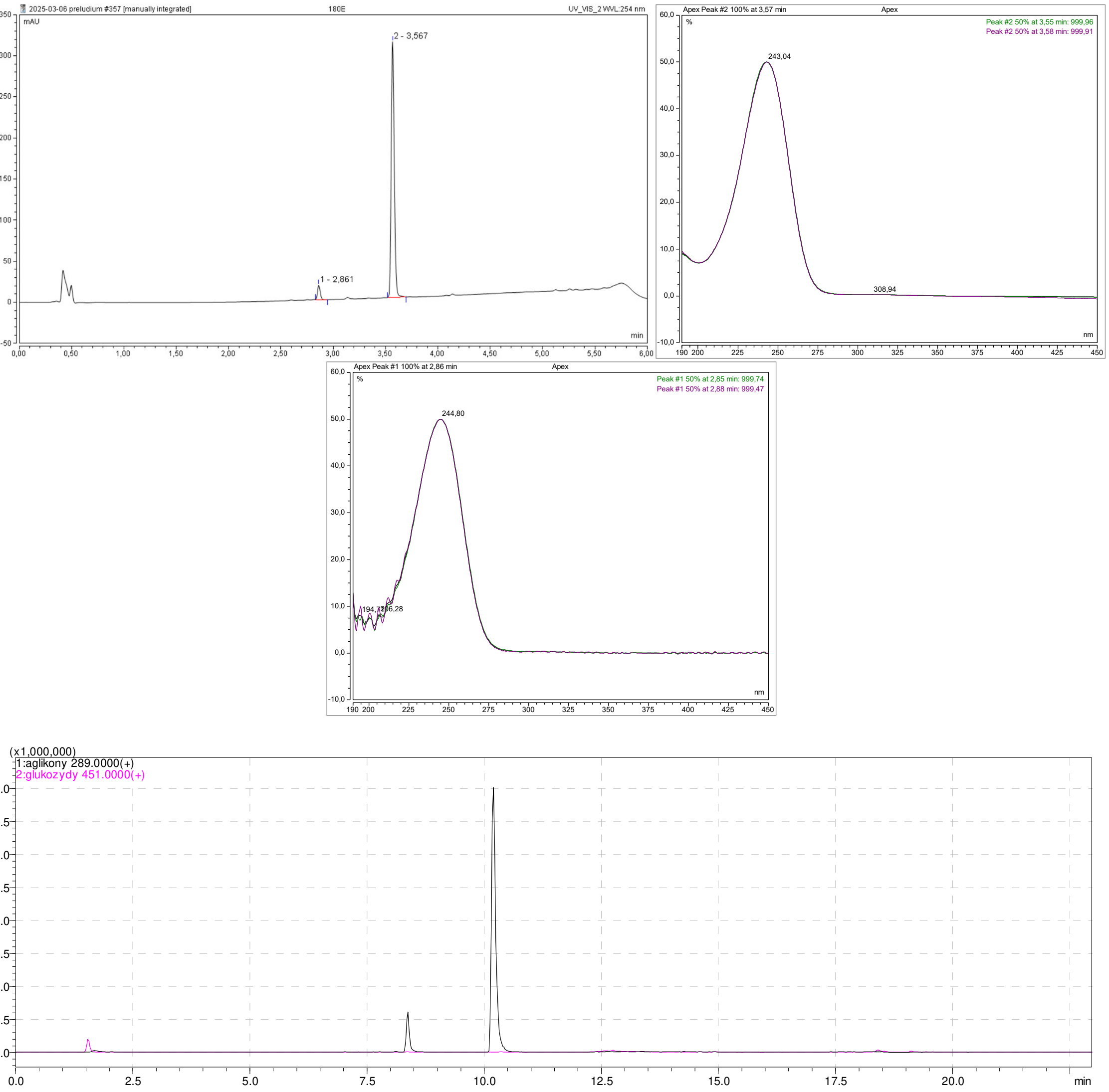

Figure S45. UPLC-DAD and LC-MS analysis of glucosylation of 17α-Testosterone by YjiC GT.

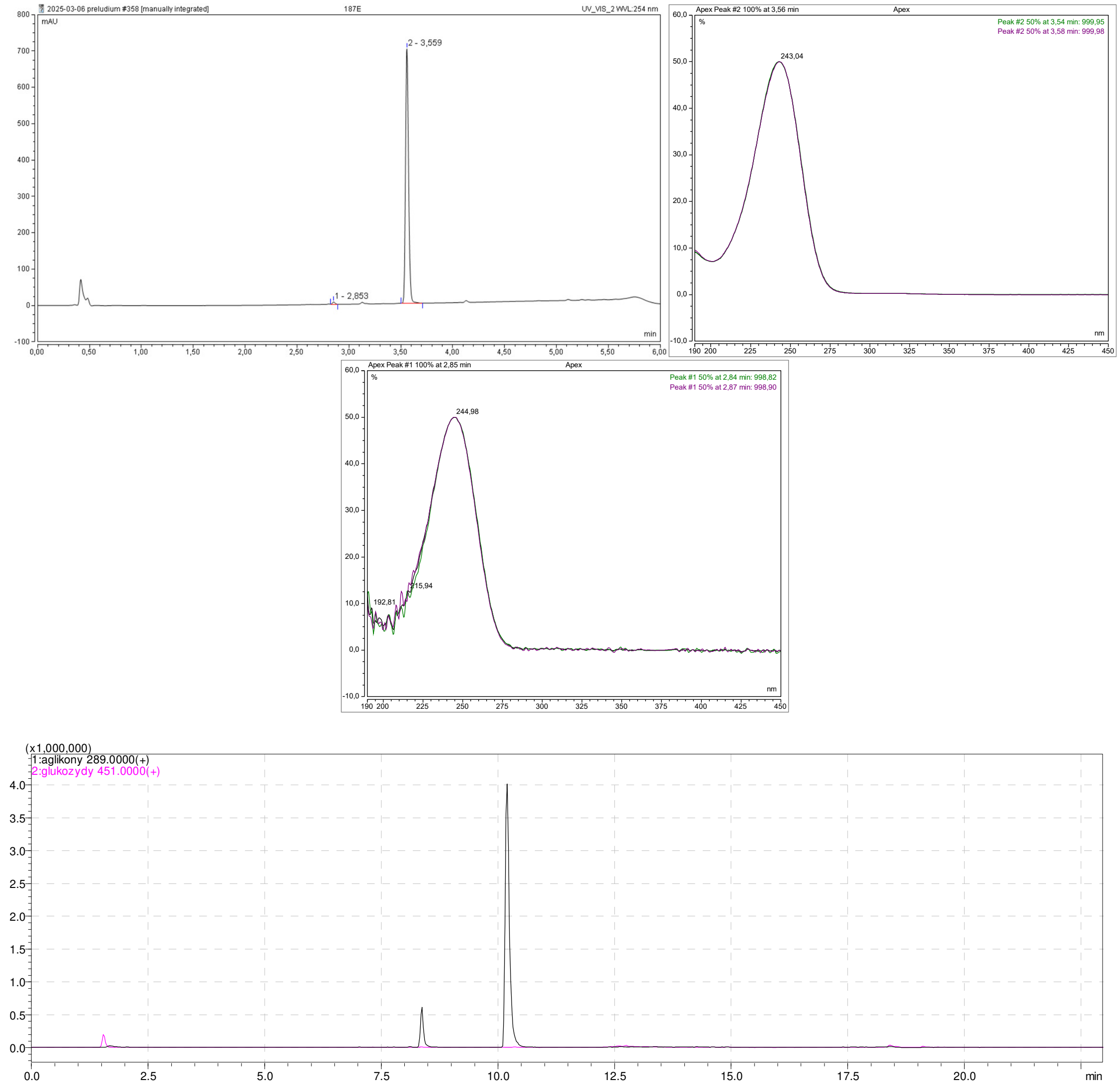

Figure S46. UPLC-DAD and LC-MS analysis of glucosylation of 17α-Testosterone by OleD GT.

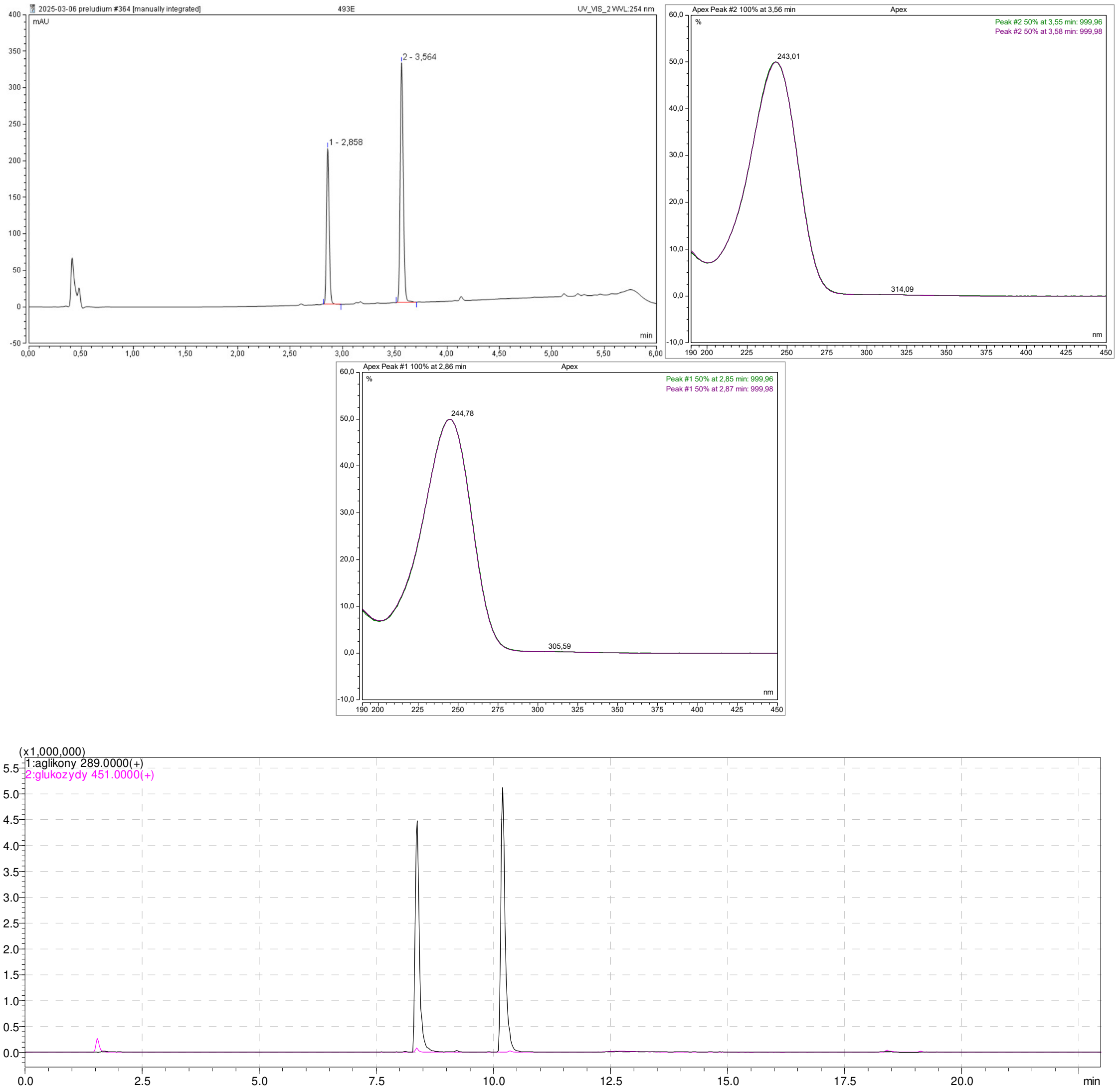

Figure S47. UPLC-DAD and LC-MS analysis of glucosylation of 17α-Testosterone by SgUGT74AC1_M7 GT.

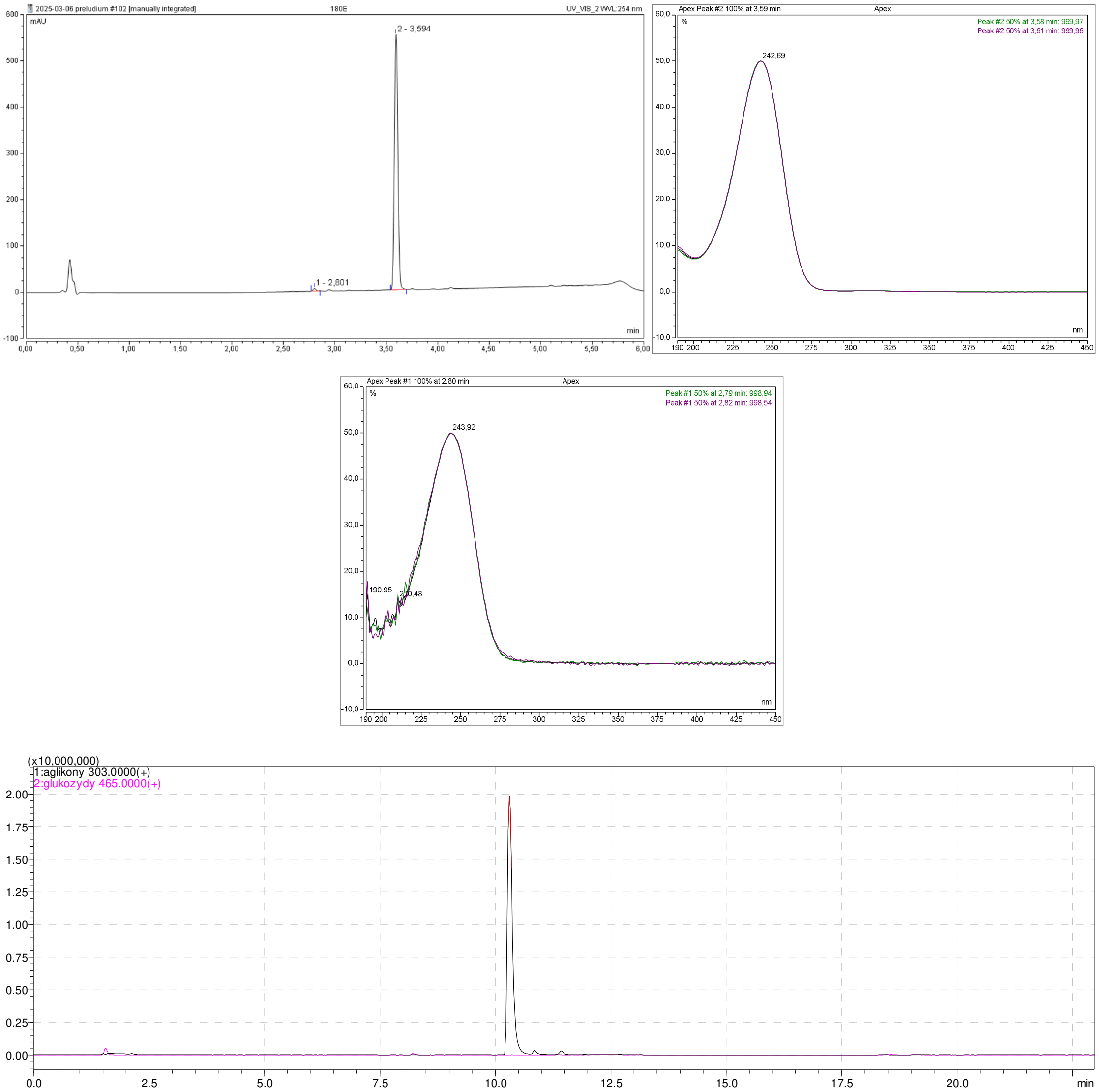

Figure S48. UPLC-DAD and LC-MS analysis of glucosylation of 17α-Methyltestosterone by YjiC GT.

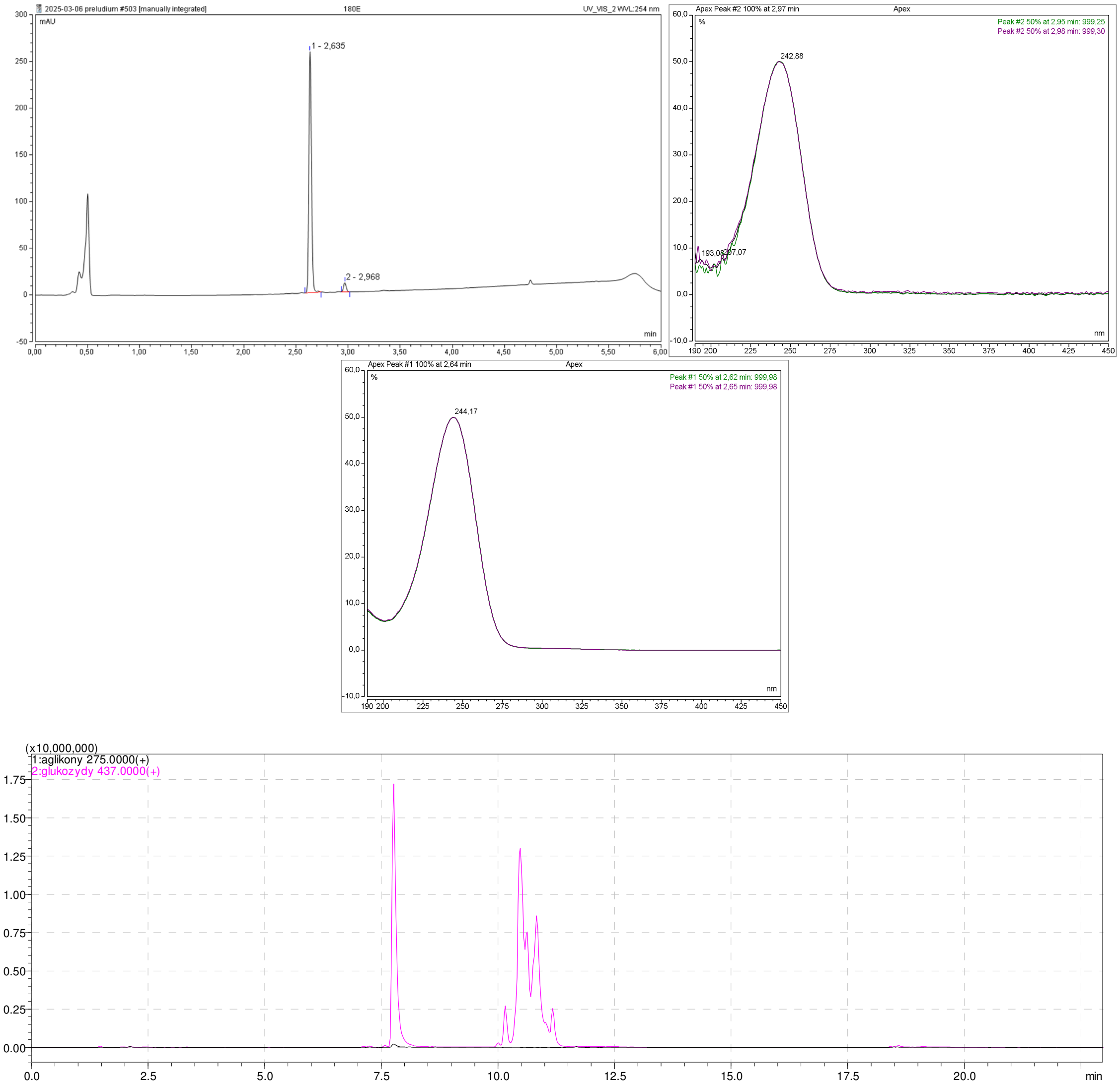

Figure S49. UPLC-DAD and LC-MS analysis of glucosylation of Nandrolone by YjiC GT. Product proportion – 96.3:3.7%

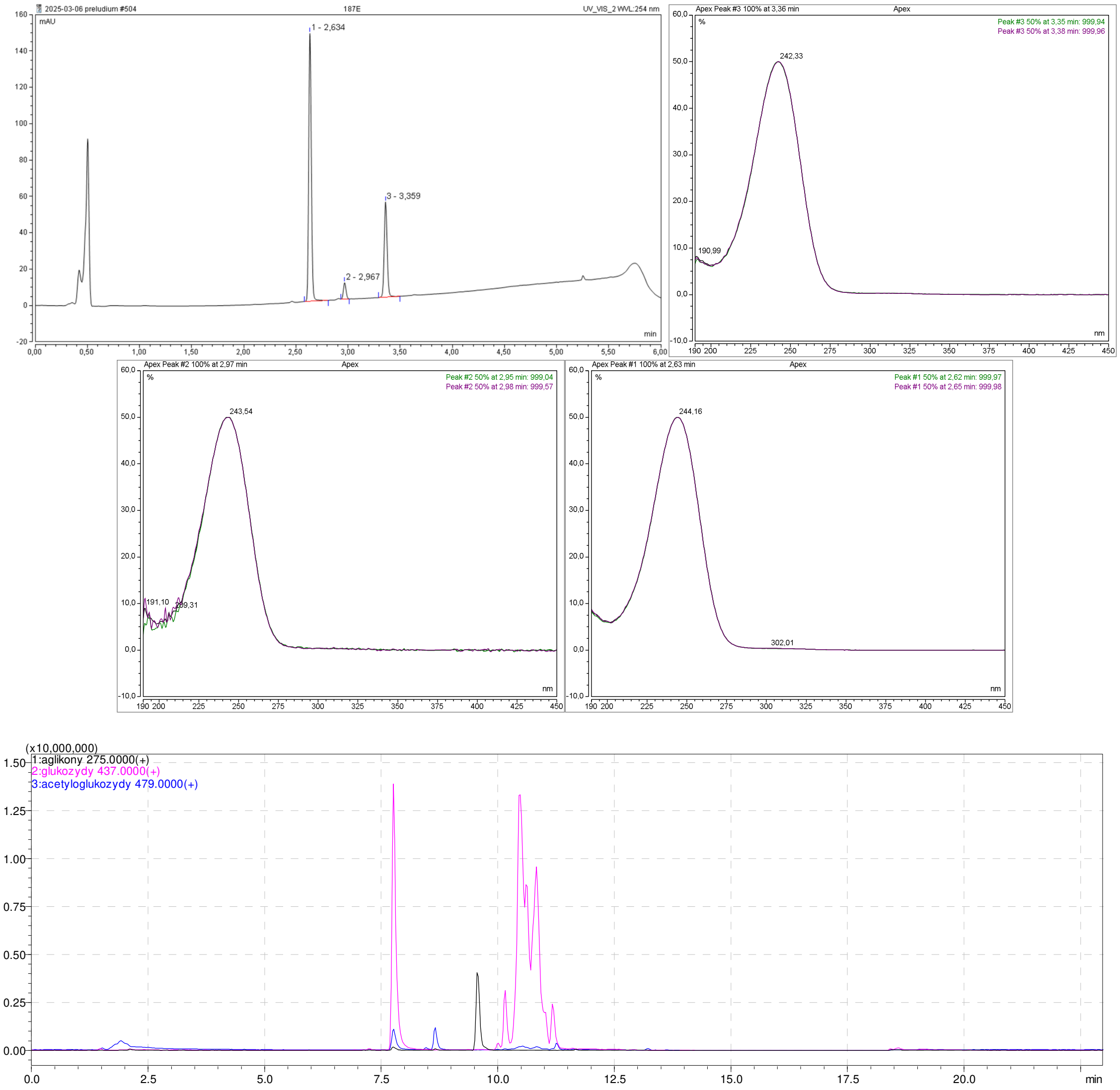

Figure S50. UPLC-DAD and LC-MS analysis of glucosylation of Nandrolone by OleD GT. Product proportions – 94.3:5.7%

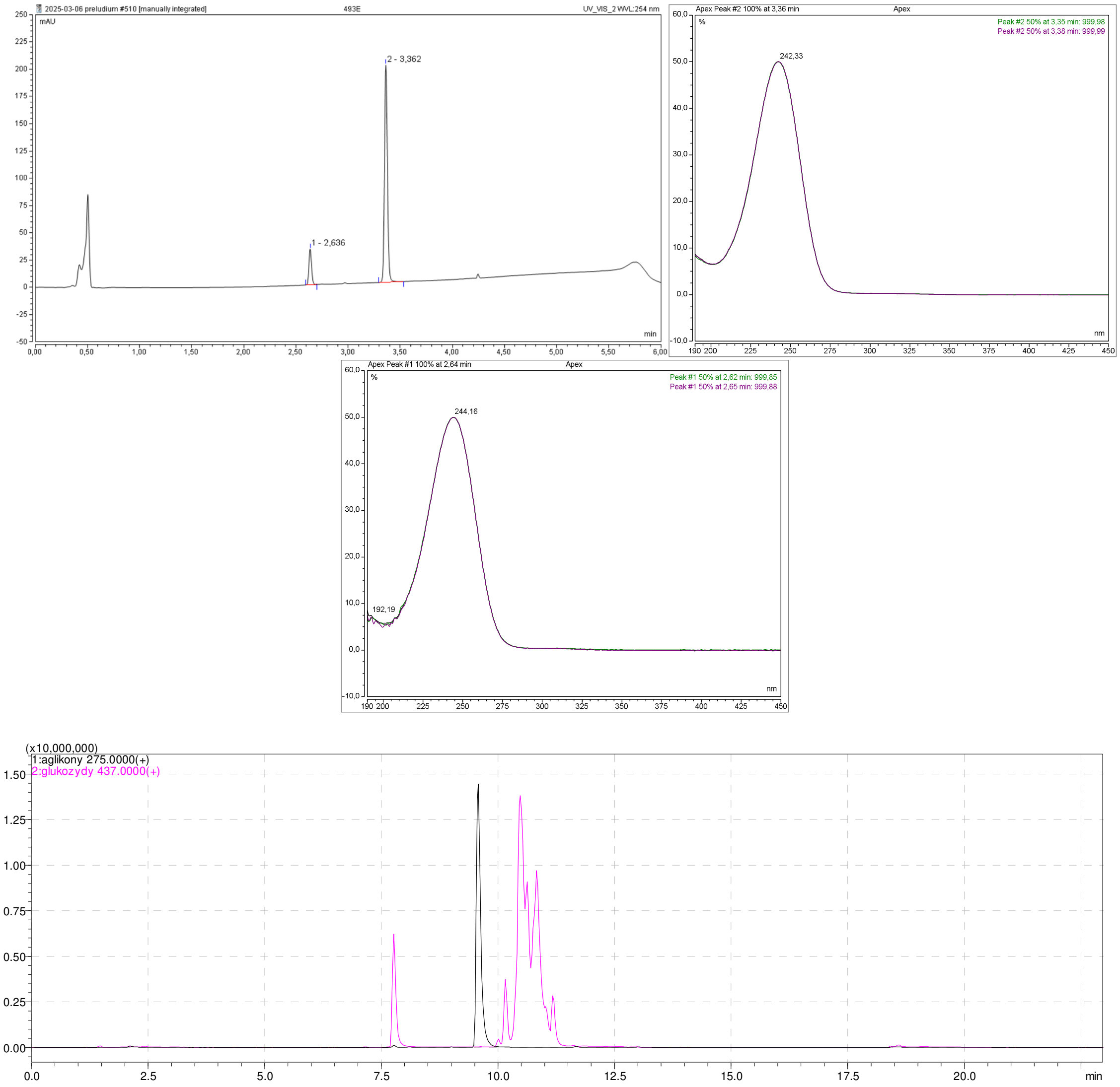

Figure S51. UPLC-DAD and LC-MS analysis of glucosylation of Nandrolone by SgUGT74AC1_M7 GT.

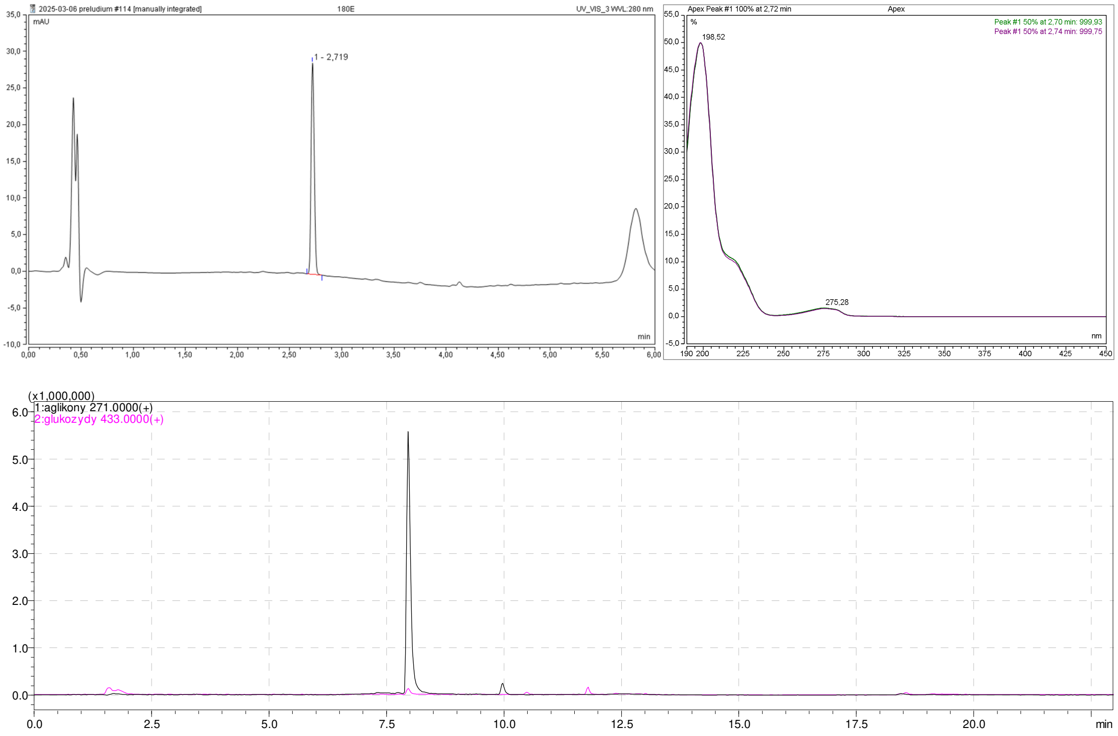

Figure S52. UPLC-DAD and LC-MS analysis of glucosylation of Estrone by YjiC GT.

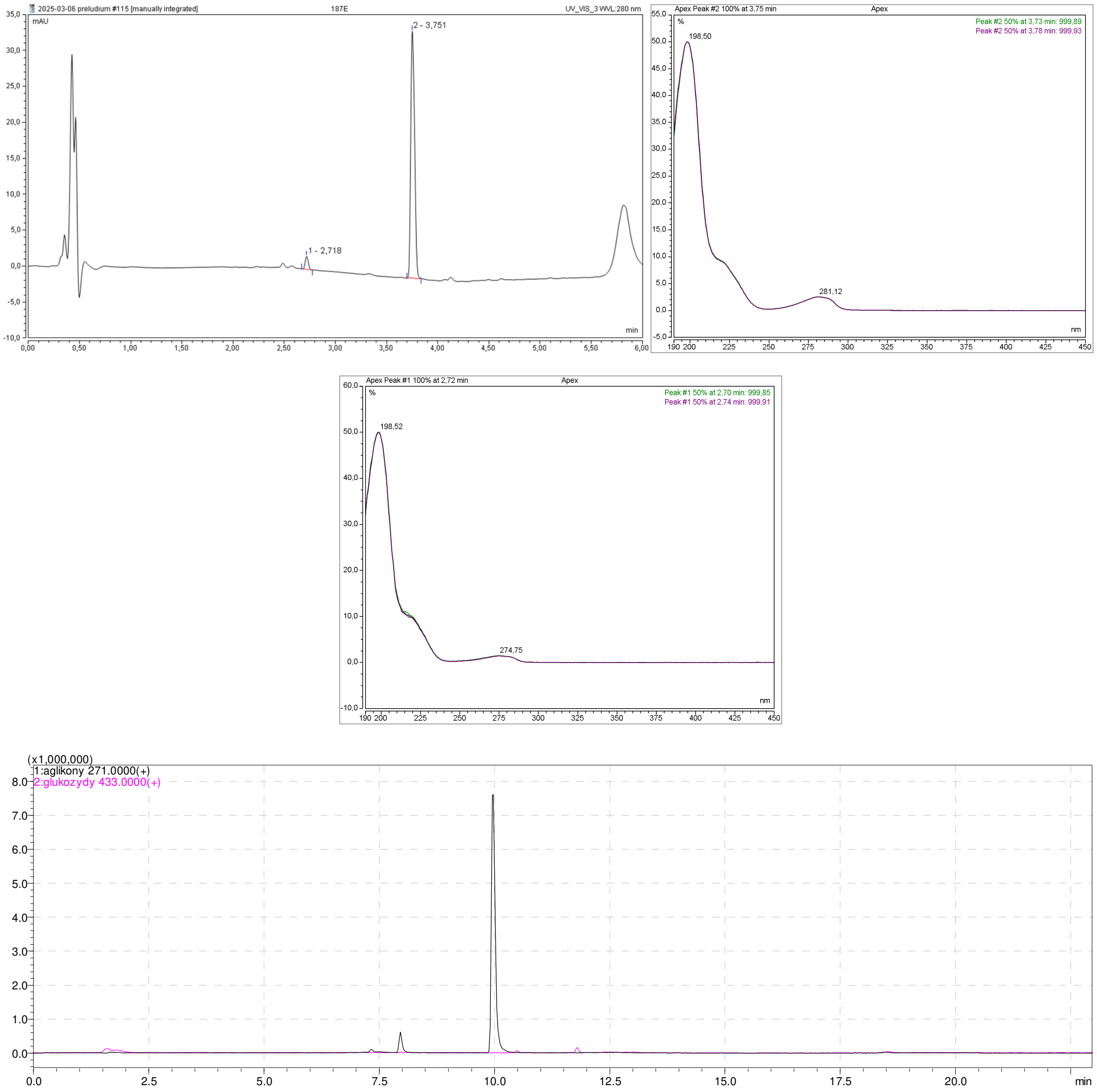

Figure S53. UPLC-DAD and LC-MS analysis of glucosylation of Estrone by OleD GT.

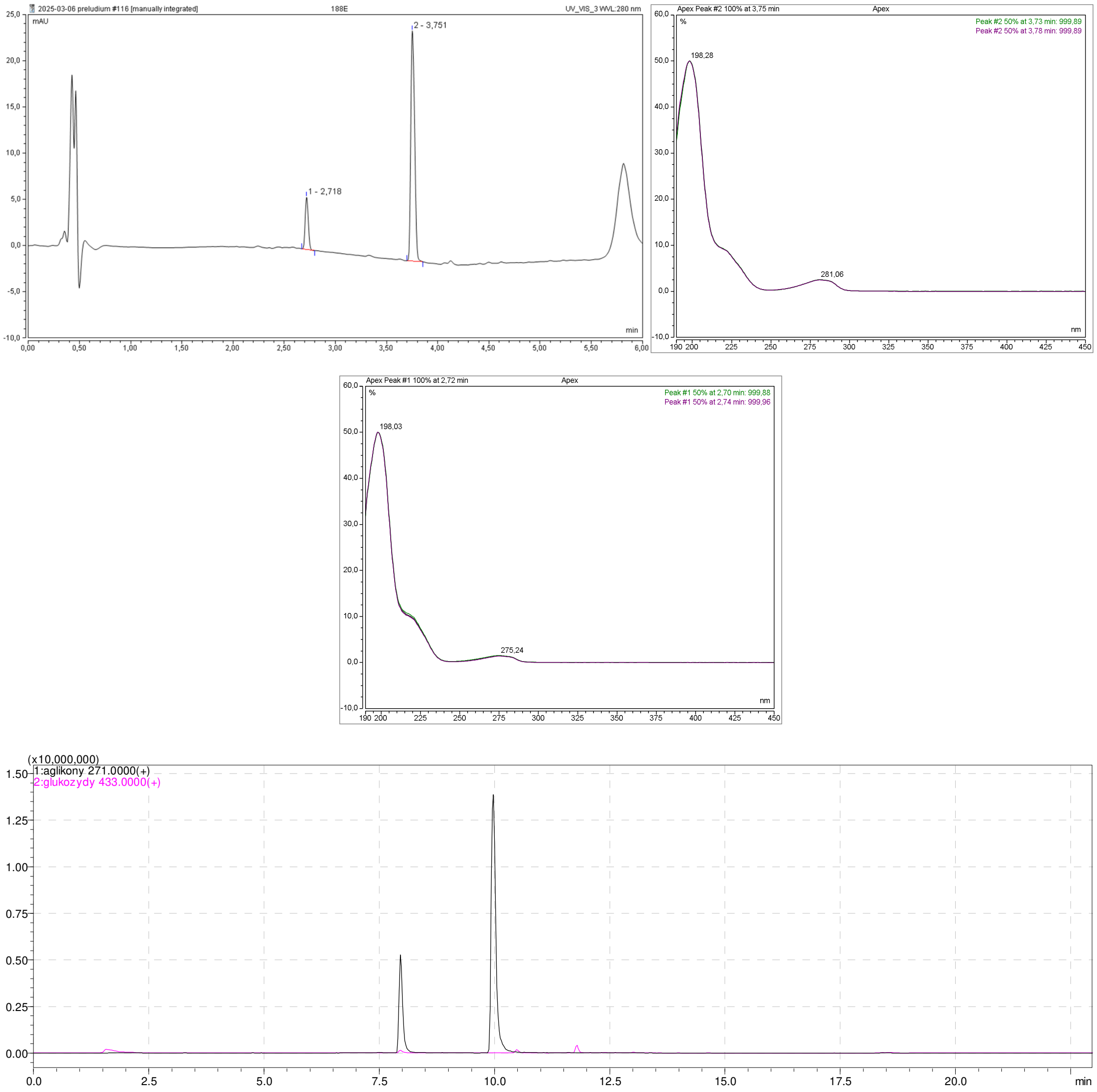

Figure S54. UPLC-DAD and LC-MS analysis of glucosylation of Estrone by Sbaic7OGT GT.

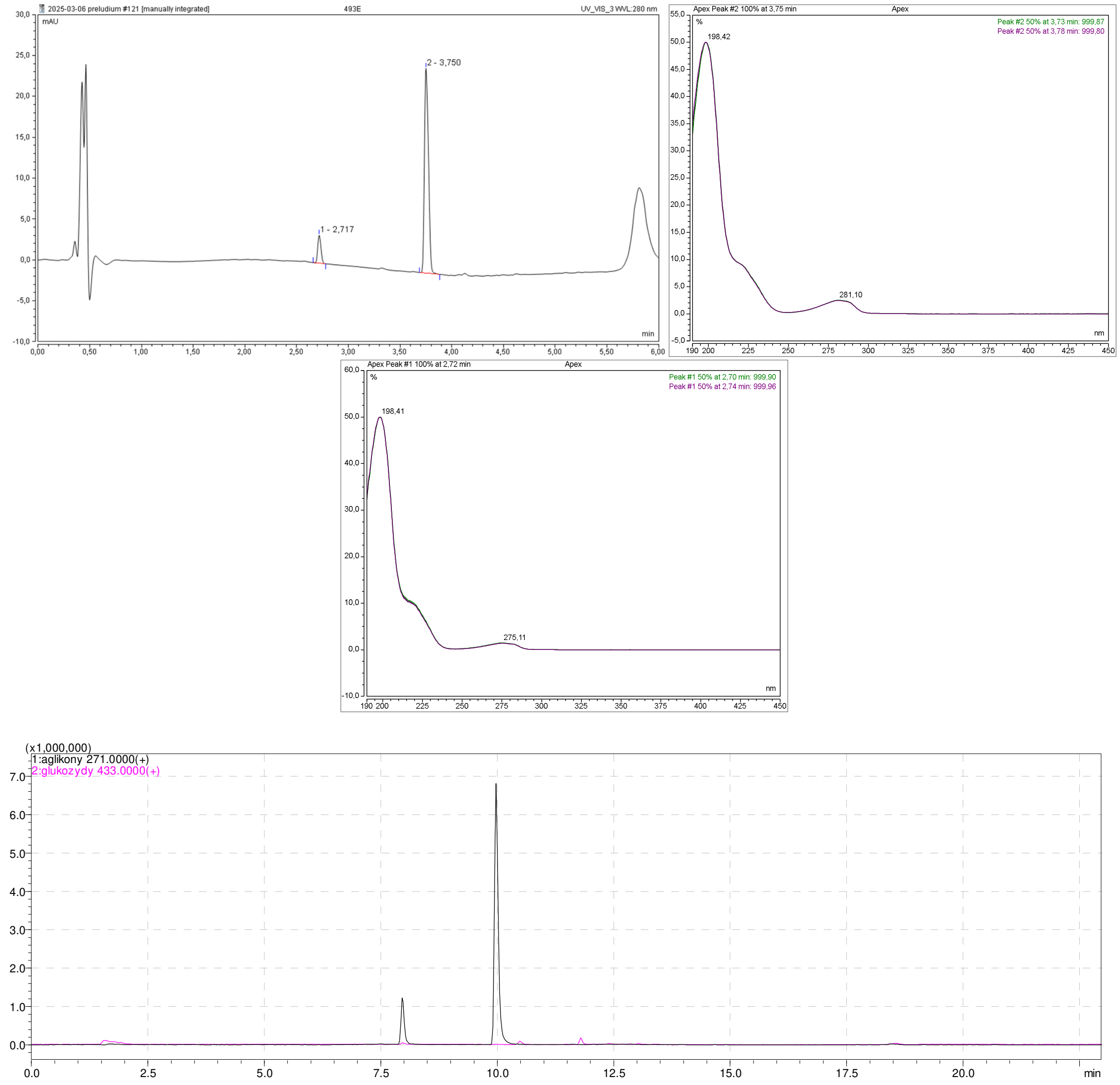

Figure S55. UPLC-DAD and LC-MS analysis of glucosylation of Estrone by SgUGT74AC1_M7 GT.

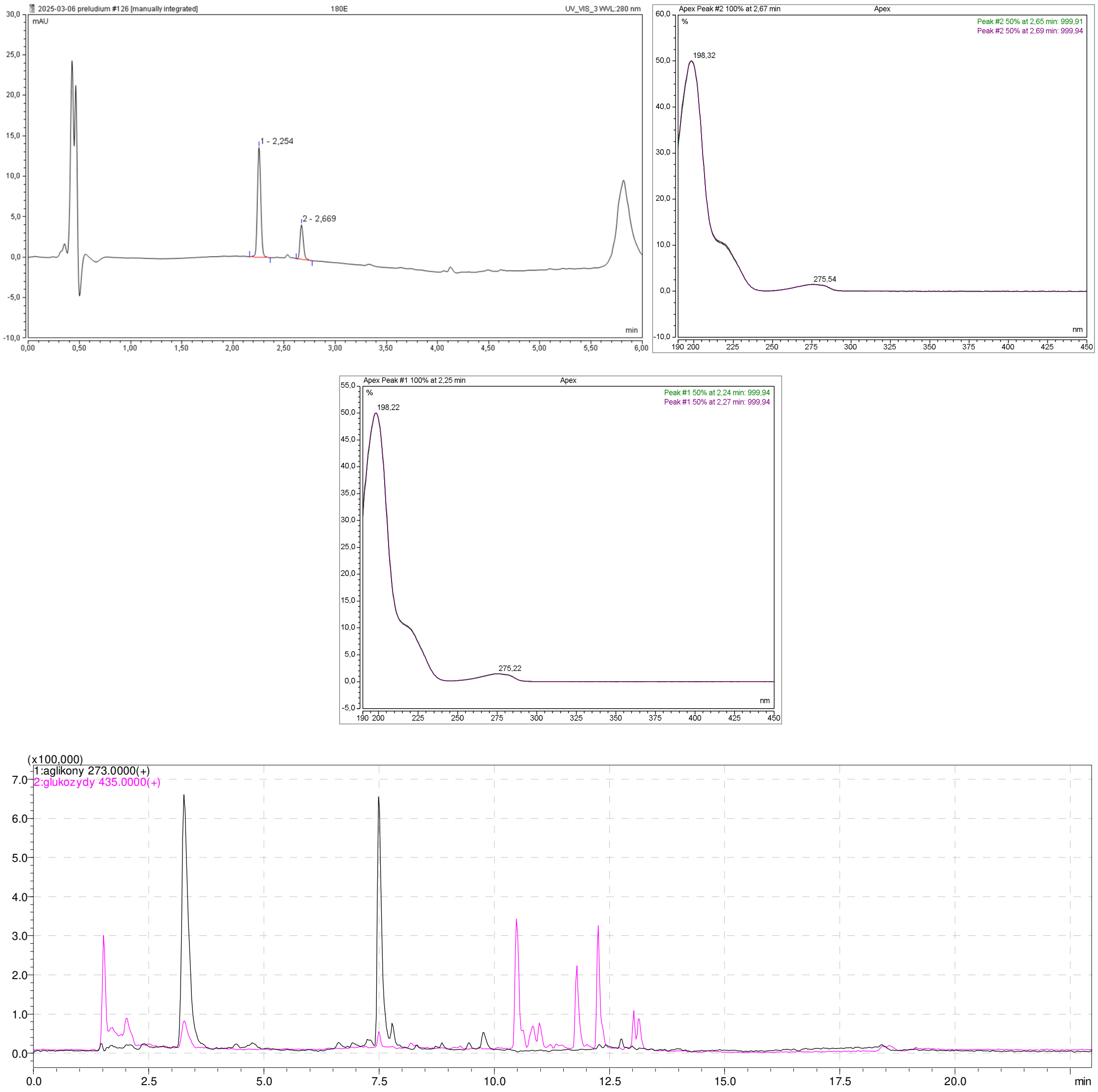

Figure S56. UPLC-DAD and LC-MS analysis of glucosylation of 17β-Estradiol by YjiC GT. Product proportions – 76.2:23.8%.

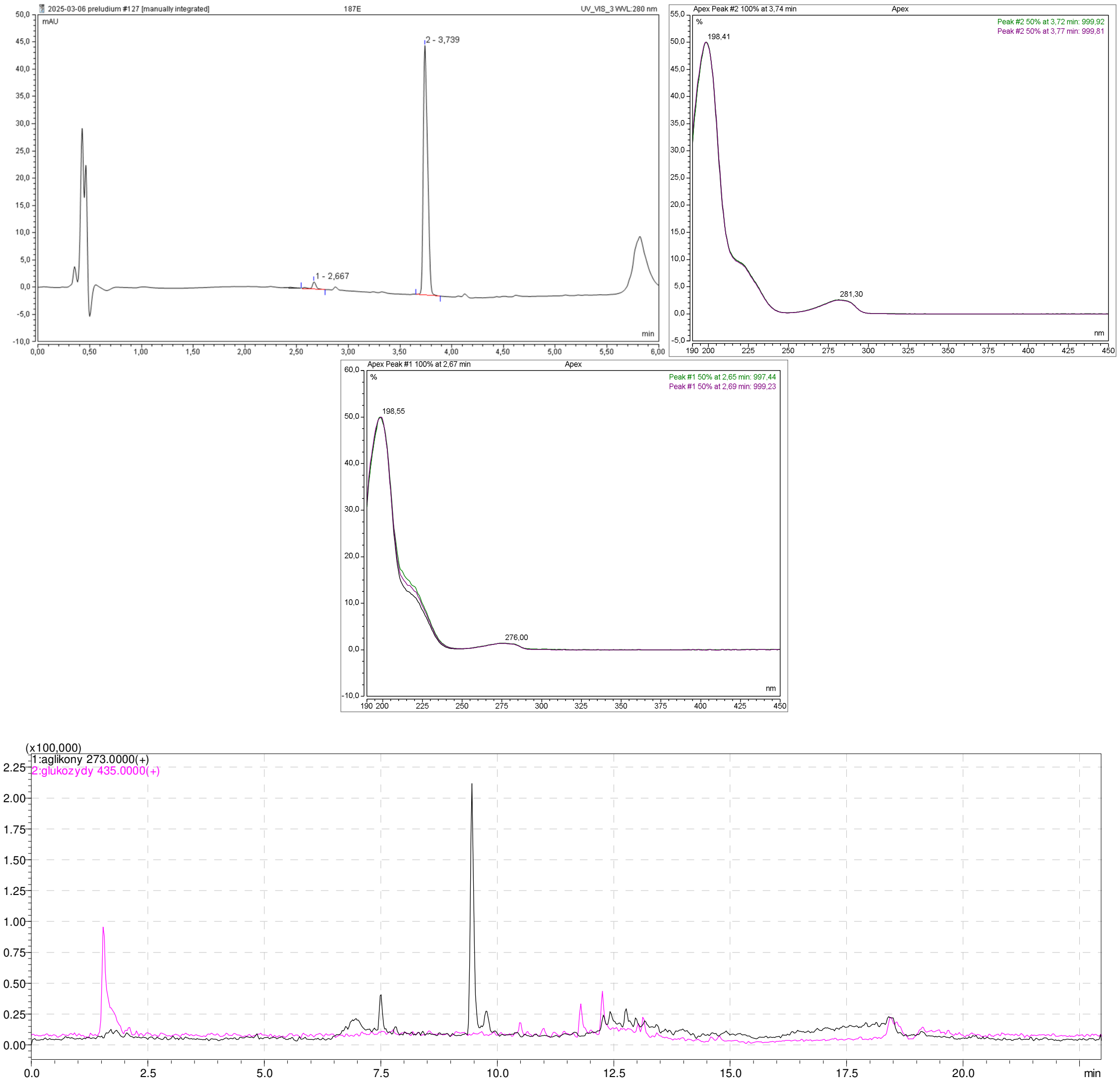

Figure S57. UPLC-DAD and LC-MS analysis of glucosylation of 17β-Estradiol by OleD GT.

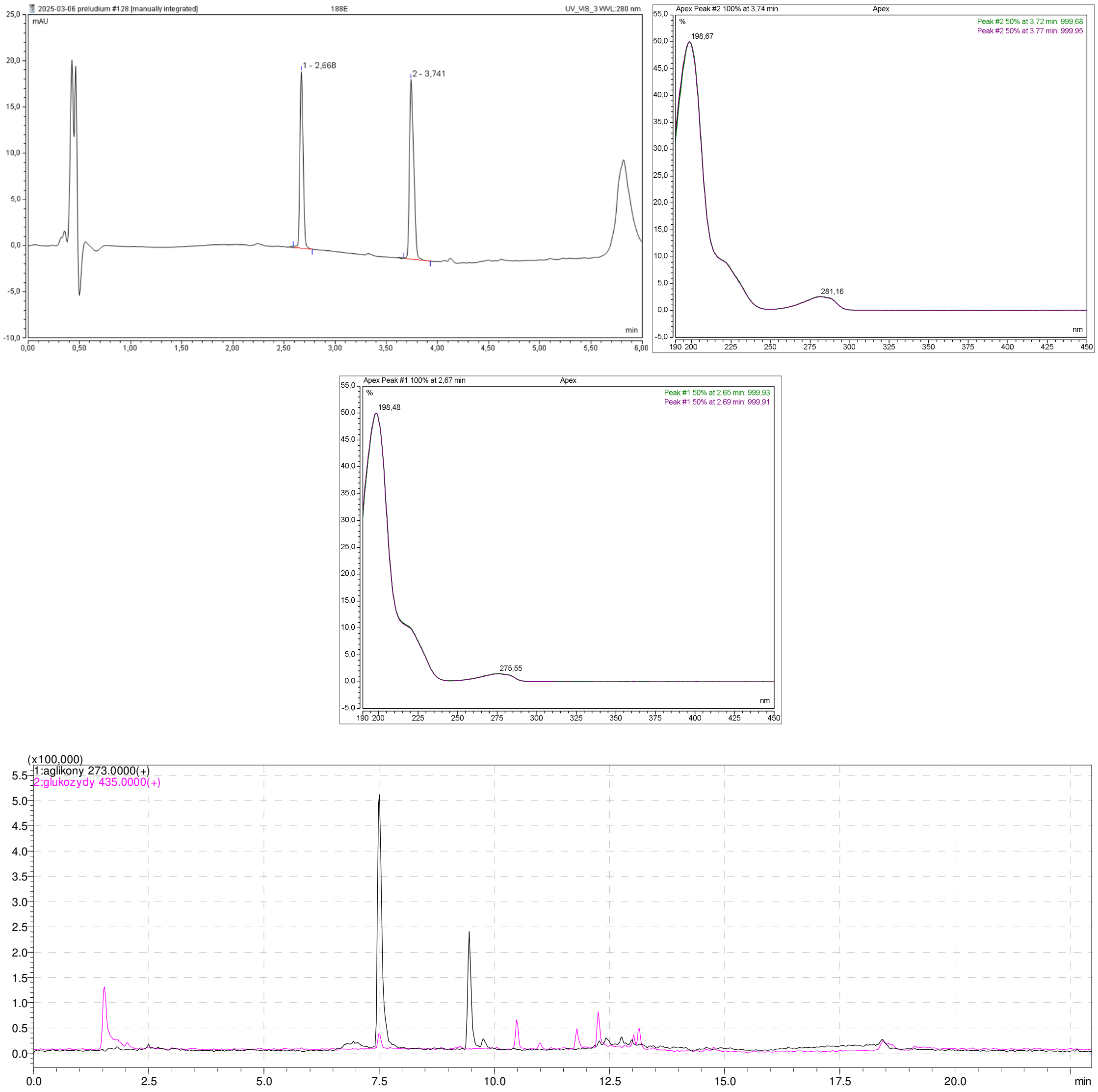

Figure S58. UPLC-DAD and LC-MS analysis of glucosylation of 17β-Estradiol by Sbaic7OGT GT.

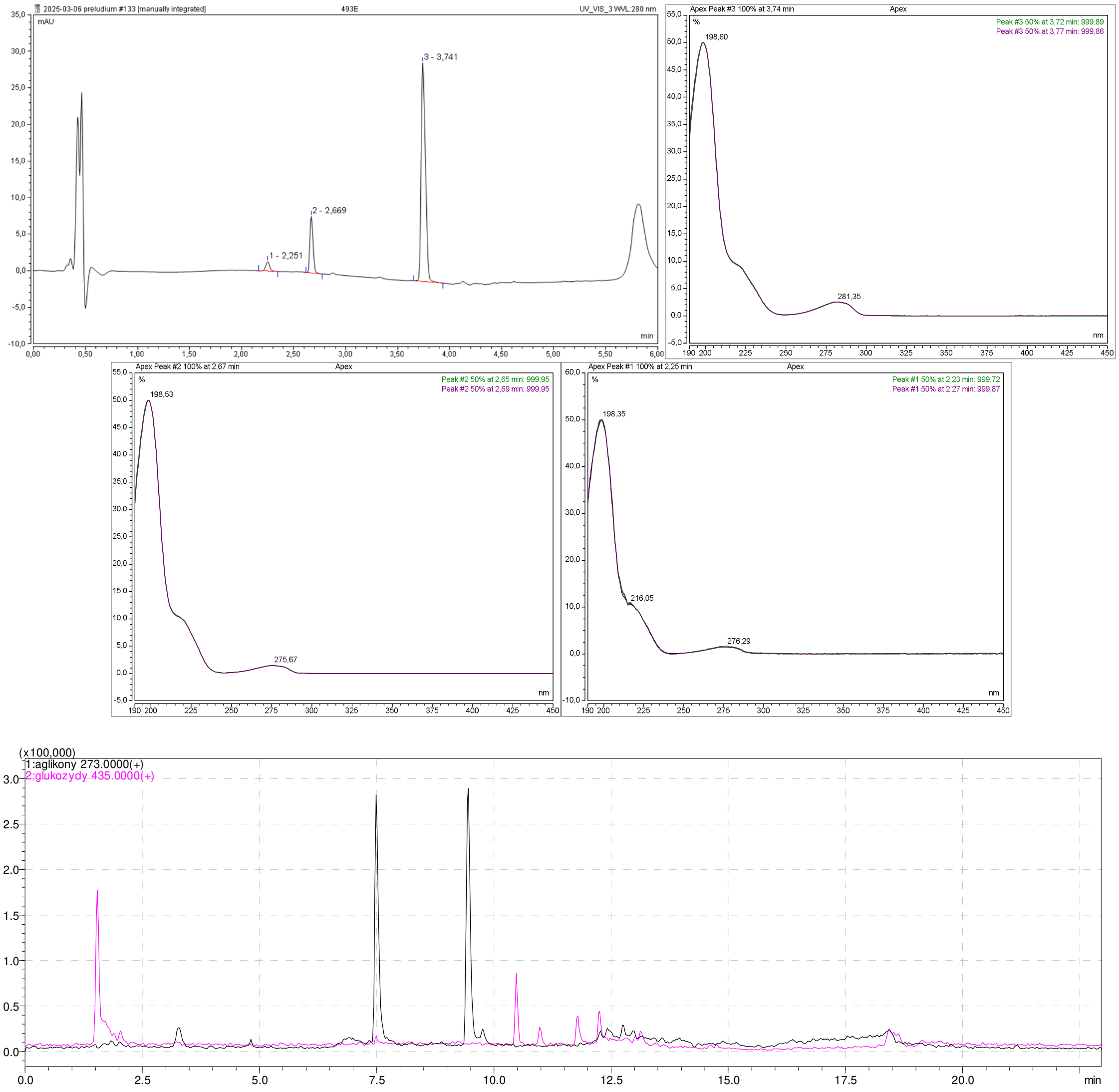

Figure S59. UPLC-DAD analysis of glucosylation of 17β-Estradiol by SgUGT74AC1_M7 GT. Product proportions – 17.1:82.9%

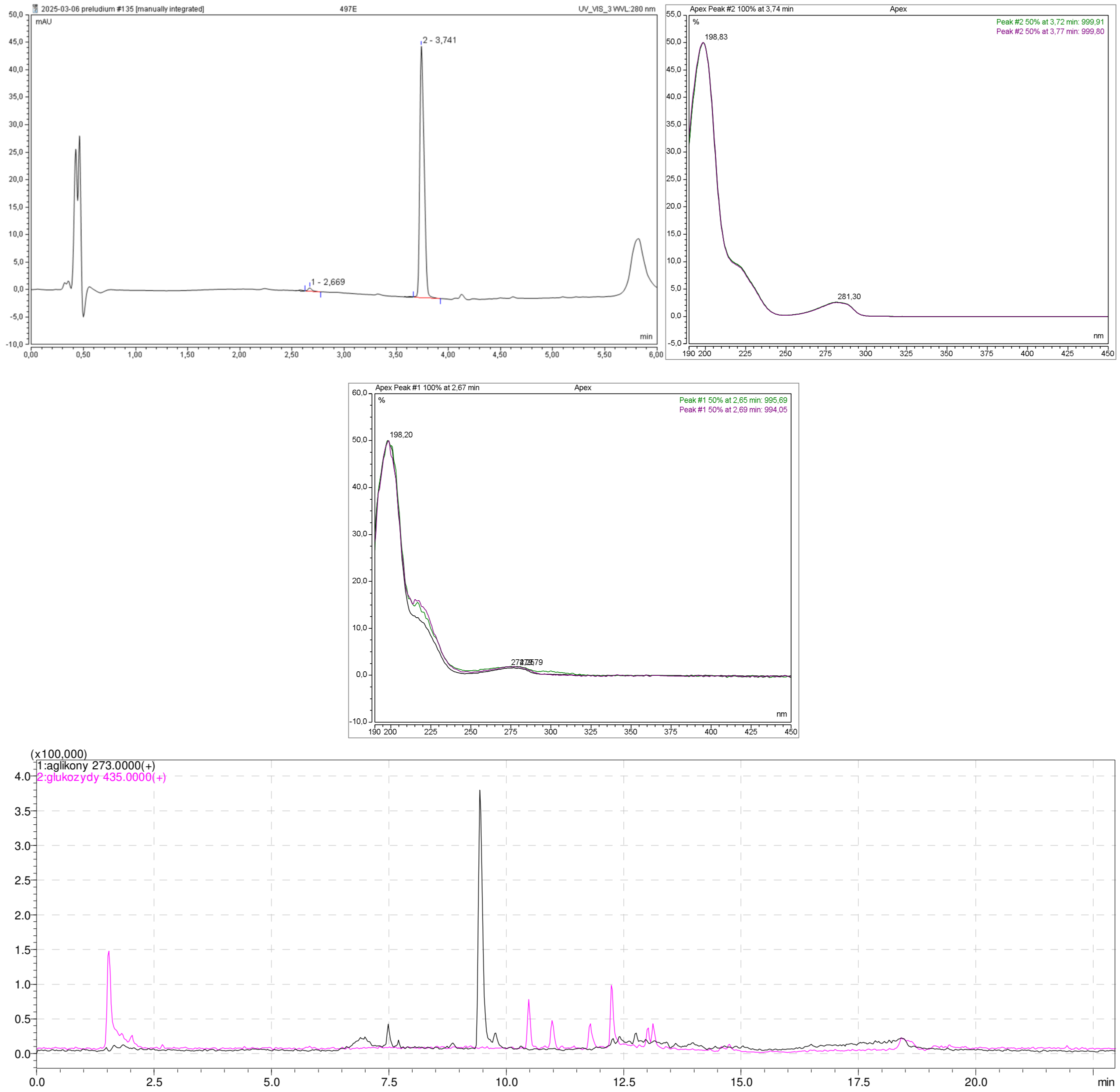

Figure S60. UPLC-DAD and LC-MS analysis of glucosylation of 17β-Estradiol by Bet5OGT GT.

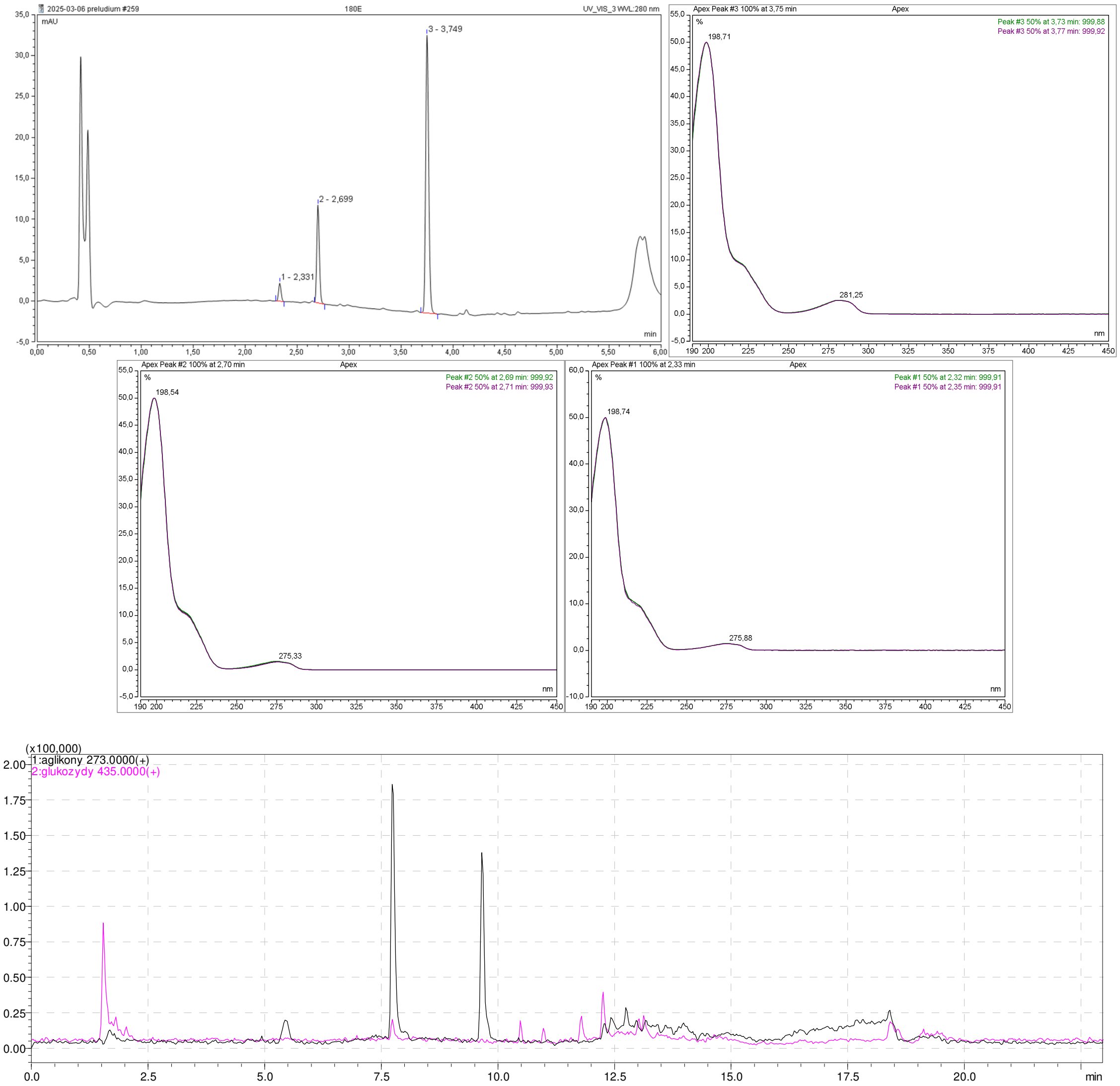

Figure S61. UPLC-DAD and LC-MS analysis of glucosylation of 17α-Estradiol by YjiC GT. Product proportions: 15.0:85.0%

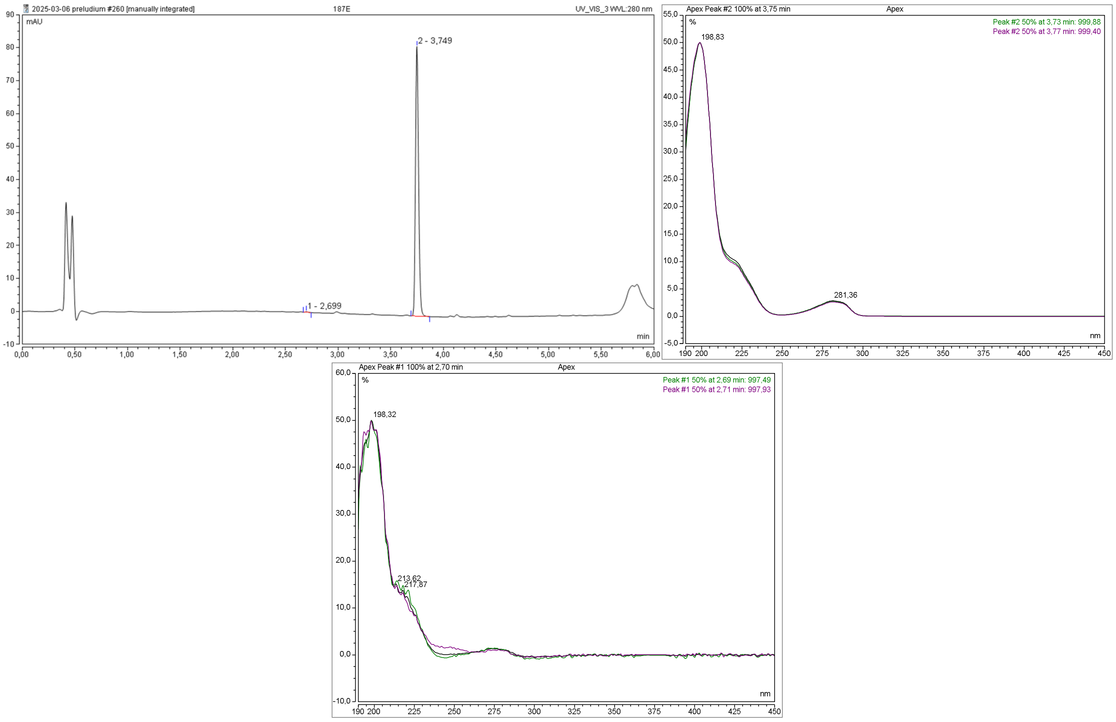

Figure S62. UPLC-DAD and LC-MS analysis of glucosylation of 17α-Estradiol by OleD GT

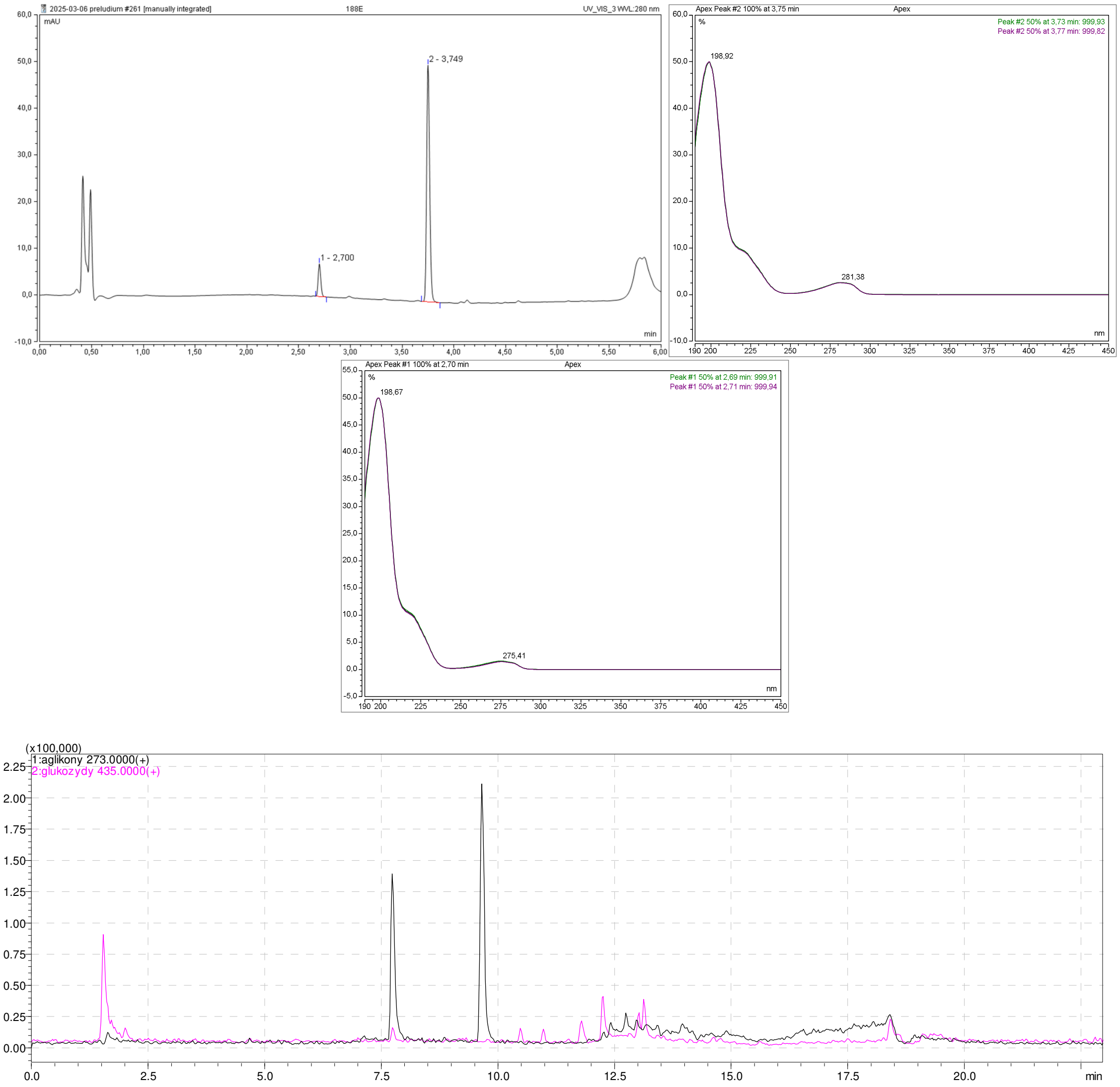

Figure S63. UPLC-DAD and LC-MS analysis of glucosylation of 17α-Estradiol by Sbaic7OGT GT

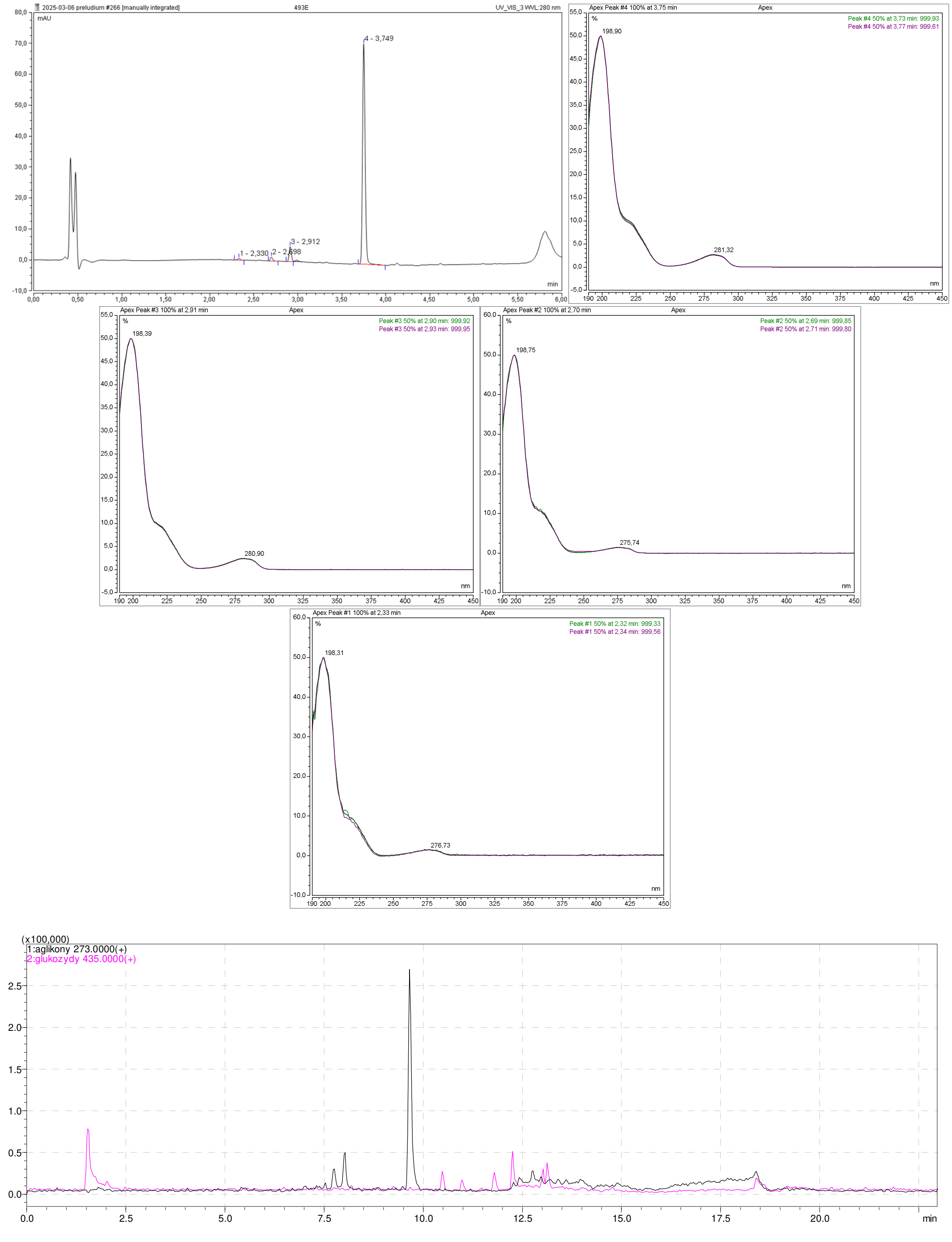

Figure S64. UPLC-DAD and LC-MS analysis of glucosylation of 17α-Estradiol by SgUGT74AC1_M7GT. Product proportions – 8.6:19.3:72.1%

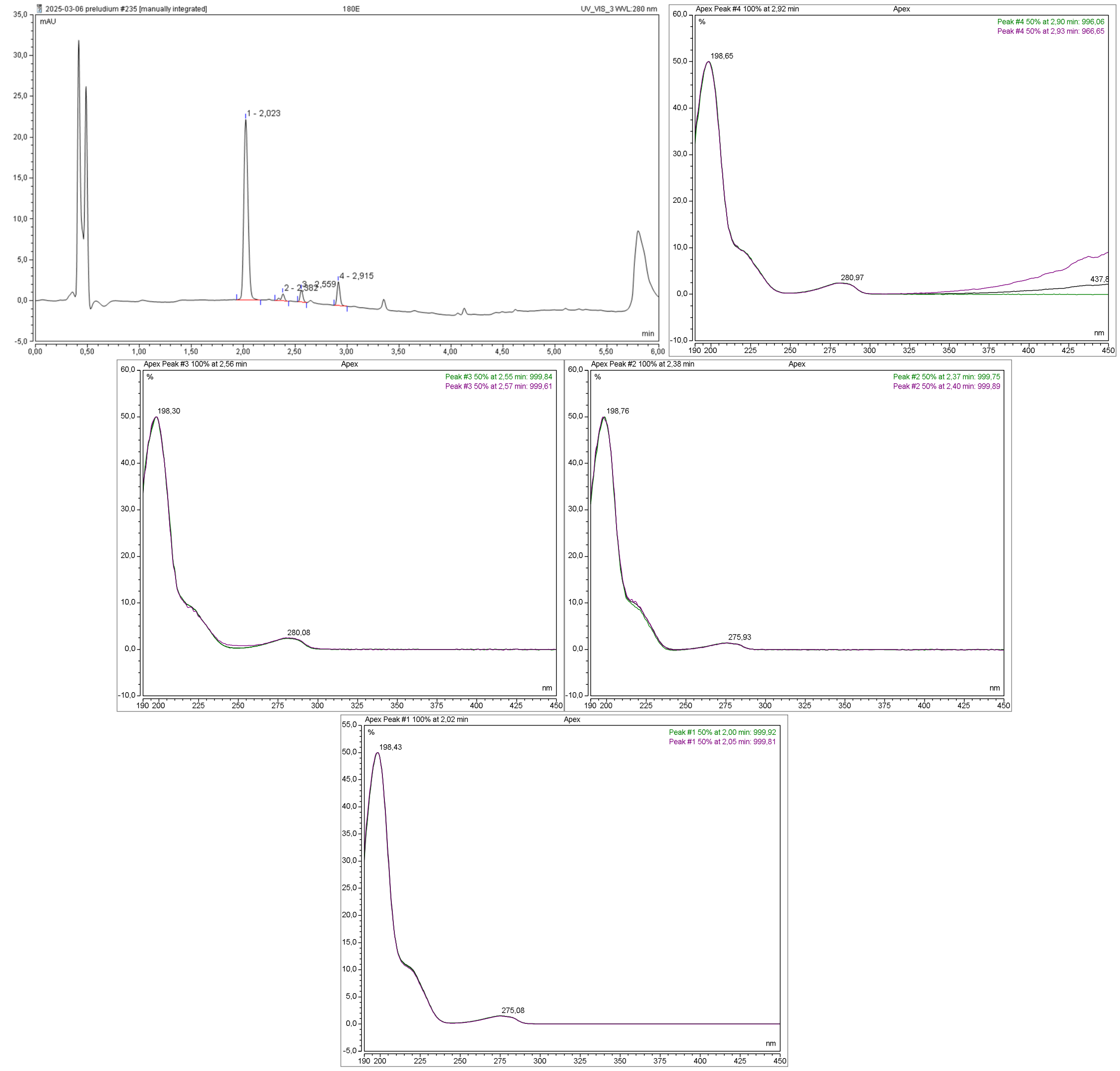

Figure S65. UPLC-DAD and LC-MS analysis of glucosylation of Estriol by YjiC GT. Product proportions – 93.0:2.9:4.1%.

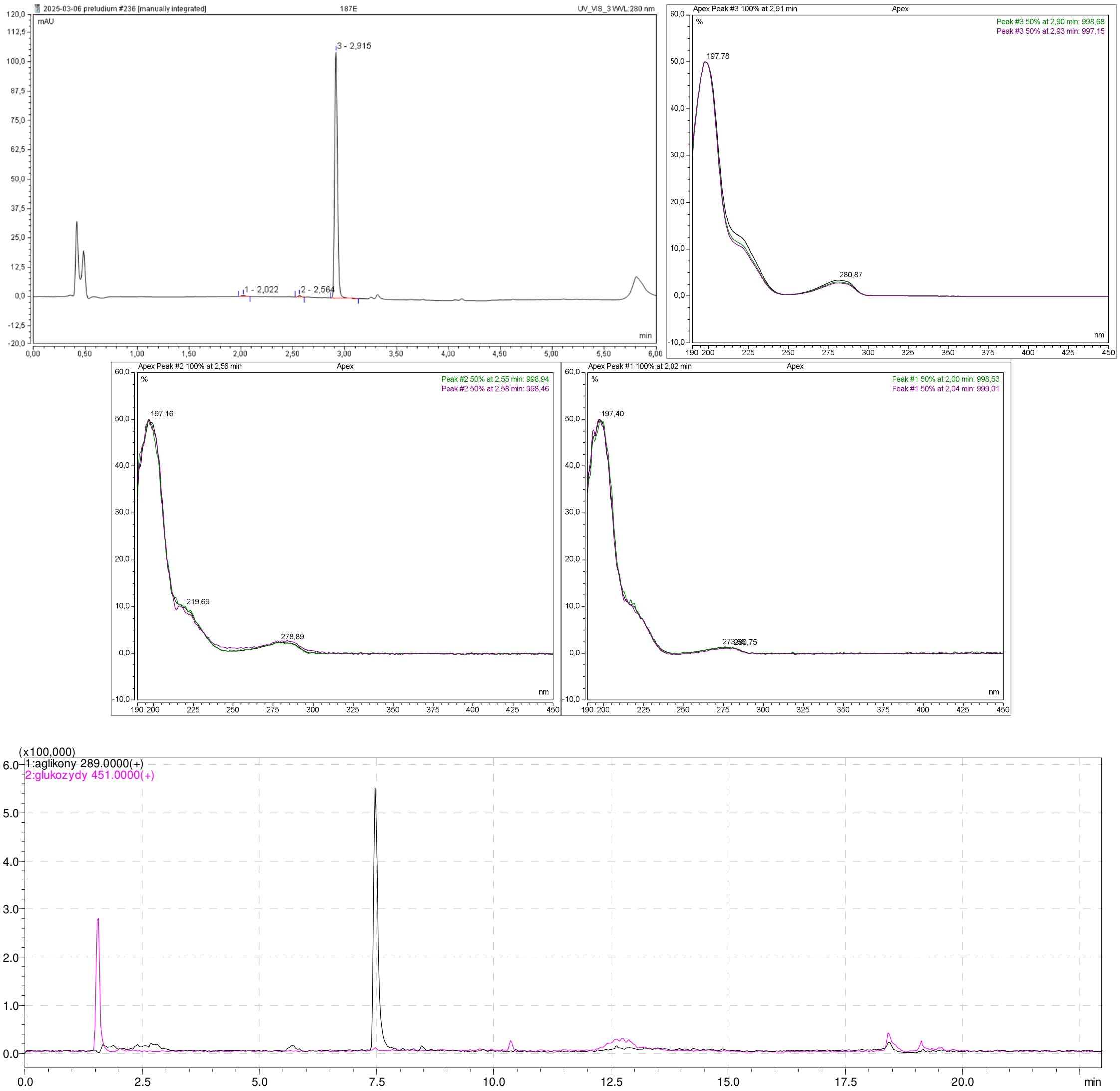

Figure S66. UPLC-DAD and LC-MS analysis of glucosylation of Estriol by OleD GT. Product proportions – 42.8:57.2%.

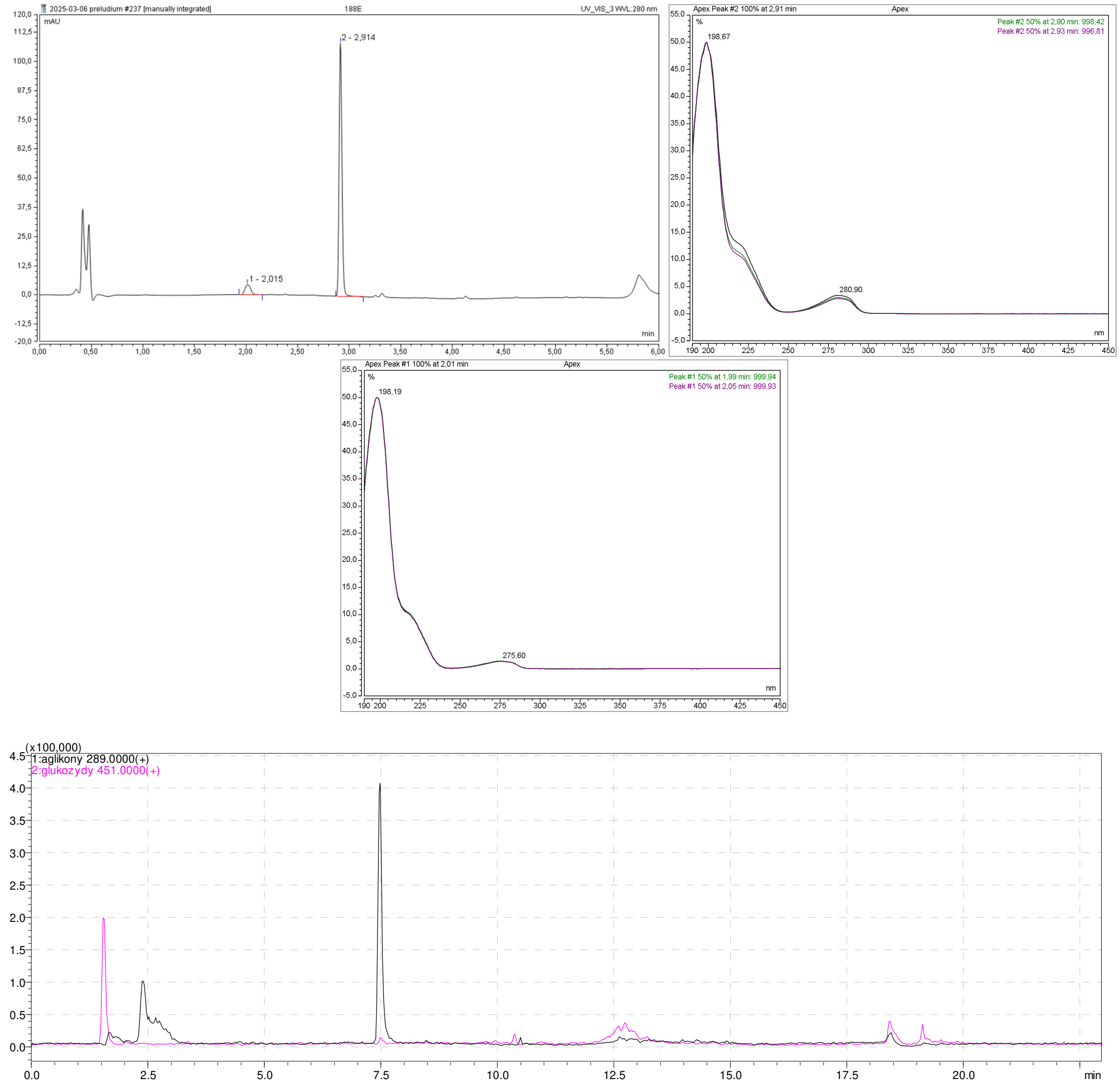

Figure S67. UPLC-DAD and LC-MS analysis of glucosylation of Estriol by Sbaic7OGT GT.

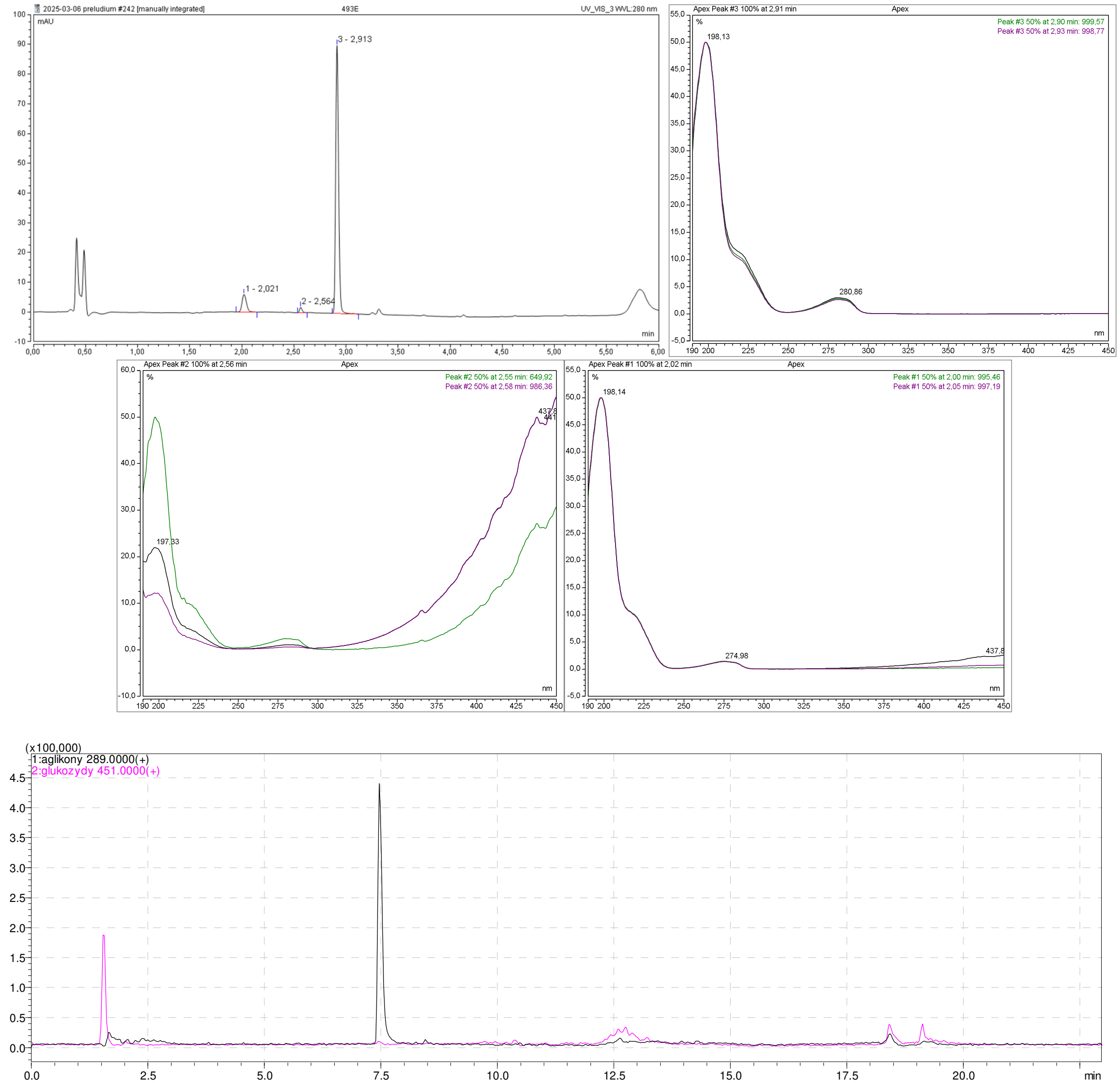

Figure S68. UPLC-DAD and LC-MS analysis of glucosylation of Estriol by SgUGT74AC1_M7 GT. Product proportions: 84.6:15.4%.

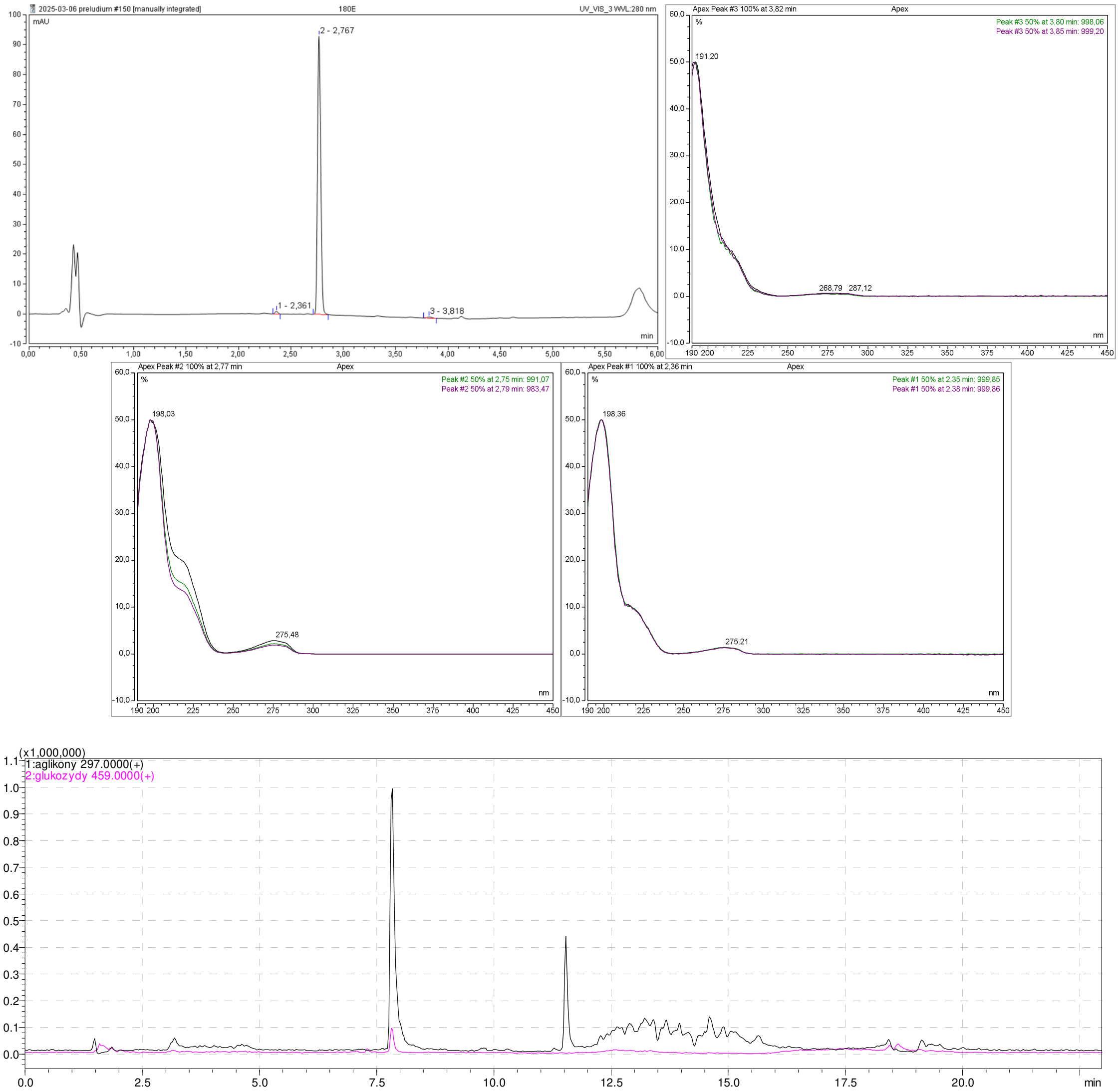

Figure S69. UPLC-DAD and LC-MS analysis of glucosylation of Ethinylestradiol by YjiC GT. Product proportions: 0.8:99.2%.

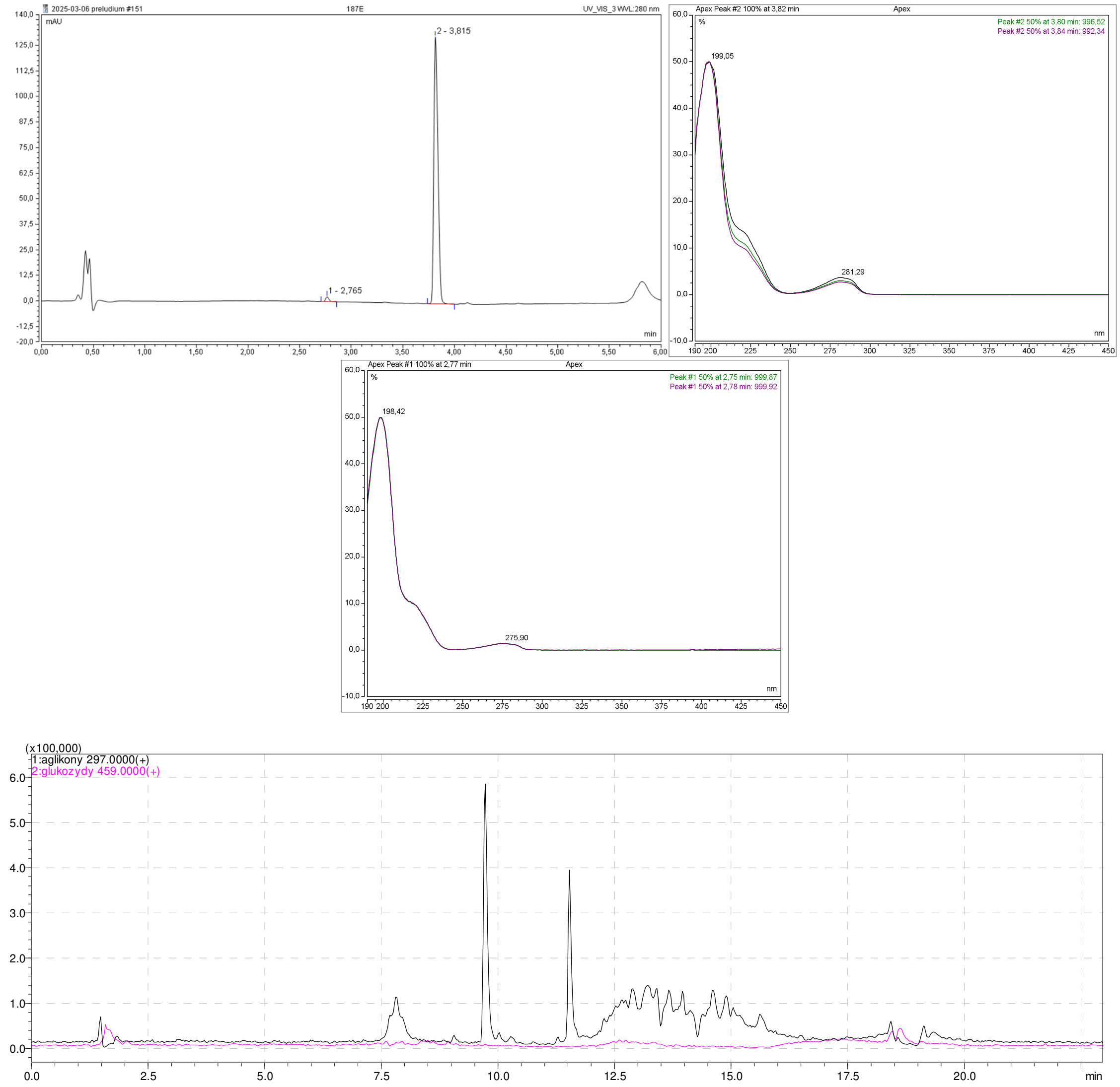

Figure S70. UPLC-DAD and LC-MS analysis of glucosylation of Ethinylestradiol by OleD GT.

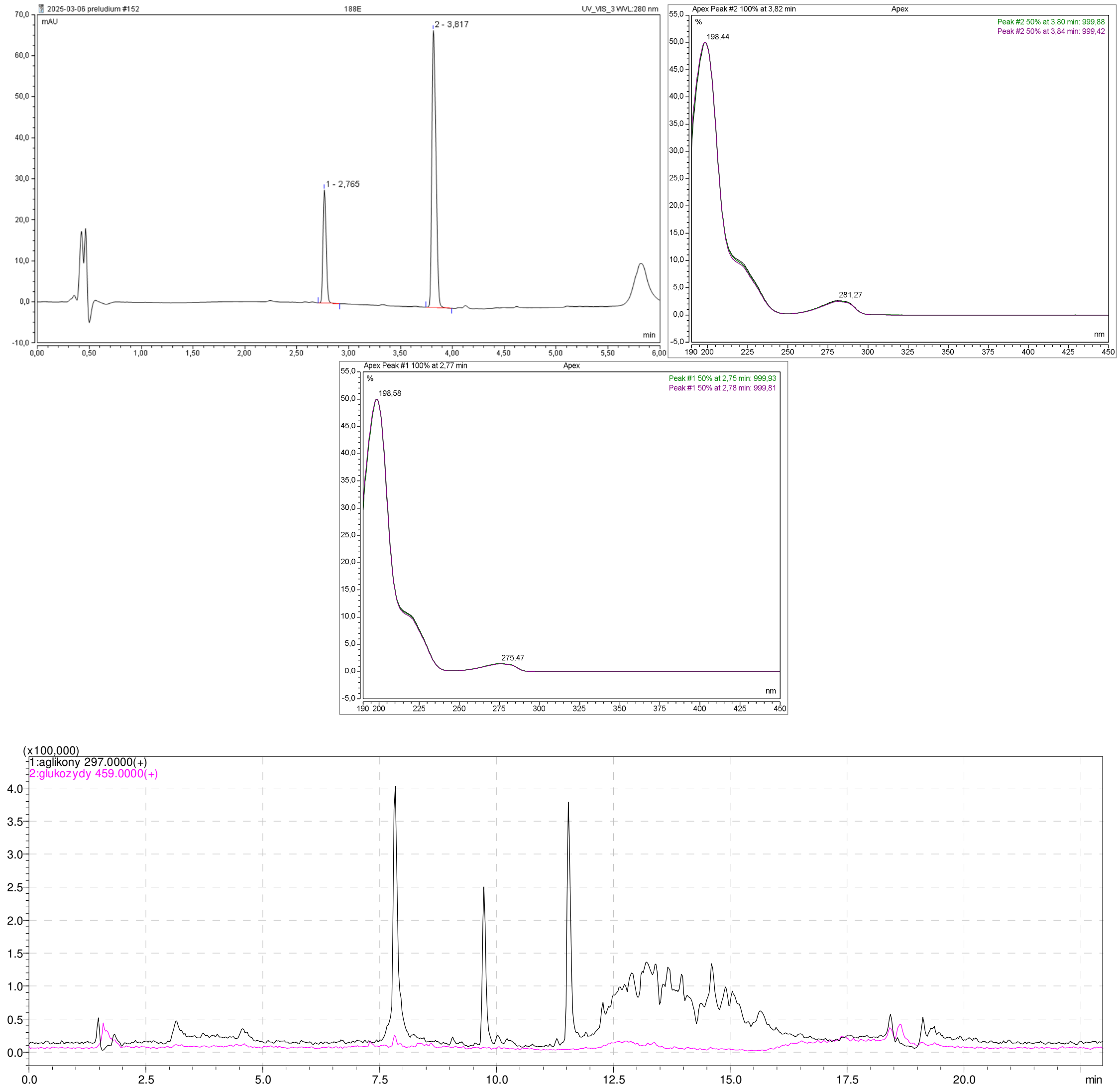

Figure S71. UPLC-DAD and LC-MS analysis of glucosylation of Ethinylestradiol by Sbaic7OGT GT.

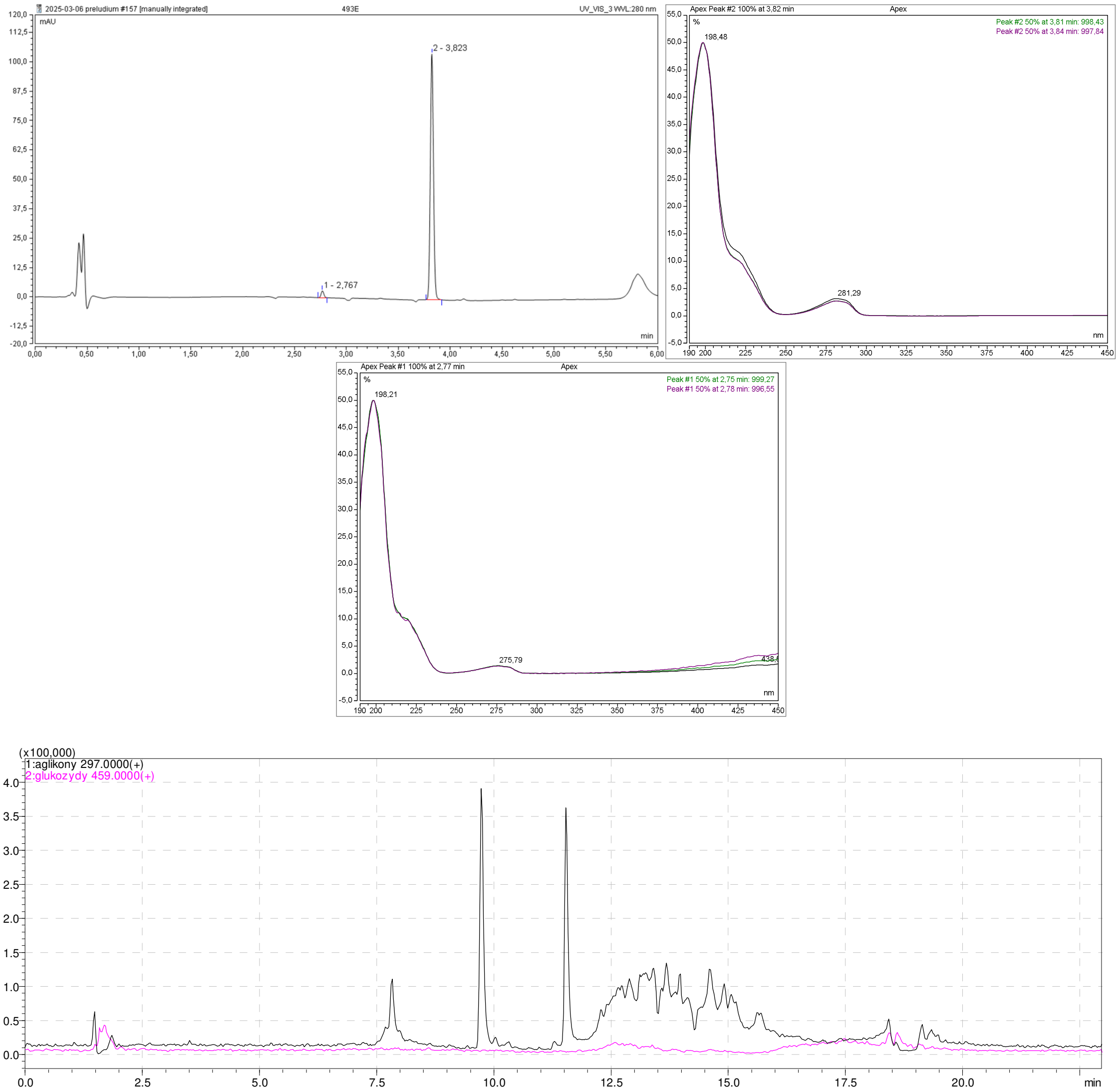

Figure S72. UPLC-DAD and LC-MS analysis of glucosylation of Ethinylestradiol by SgUGT74AC1_M7 GT.

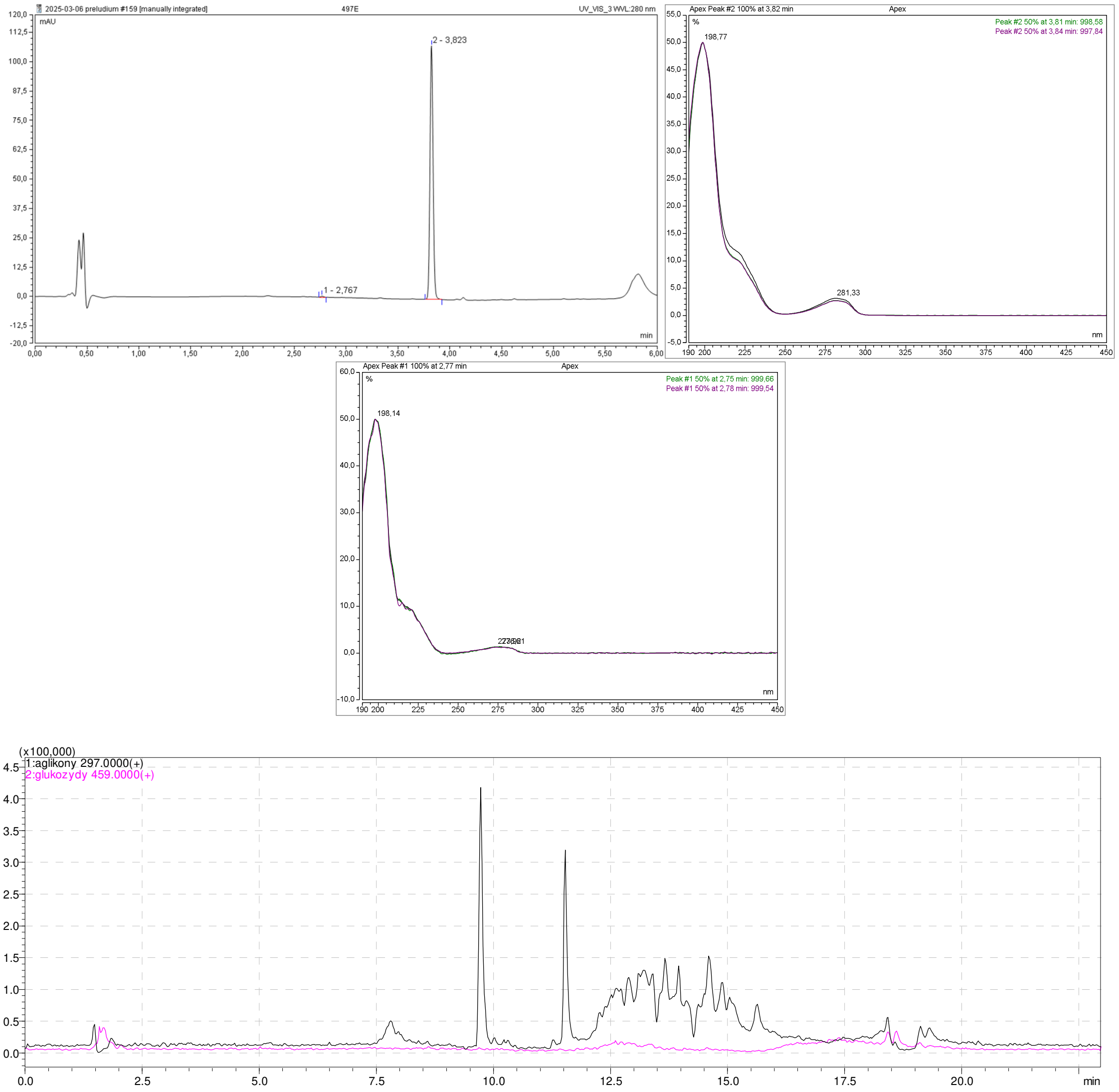

Figure S73. UPLC-DAD and LC-MS analysis of glucosylation of Ethinylestradiol by Bet5OGT GT.

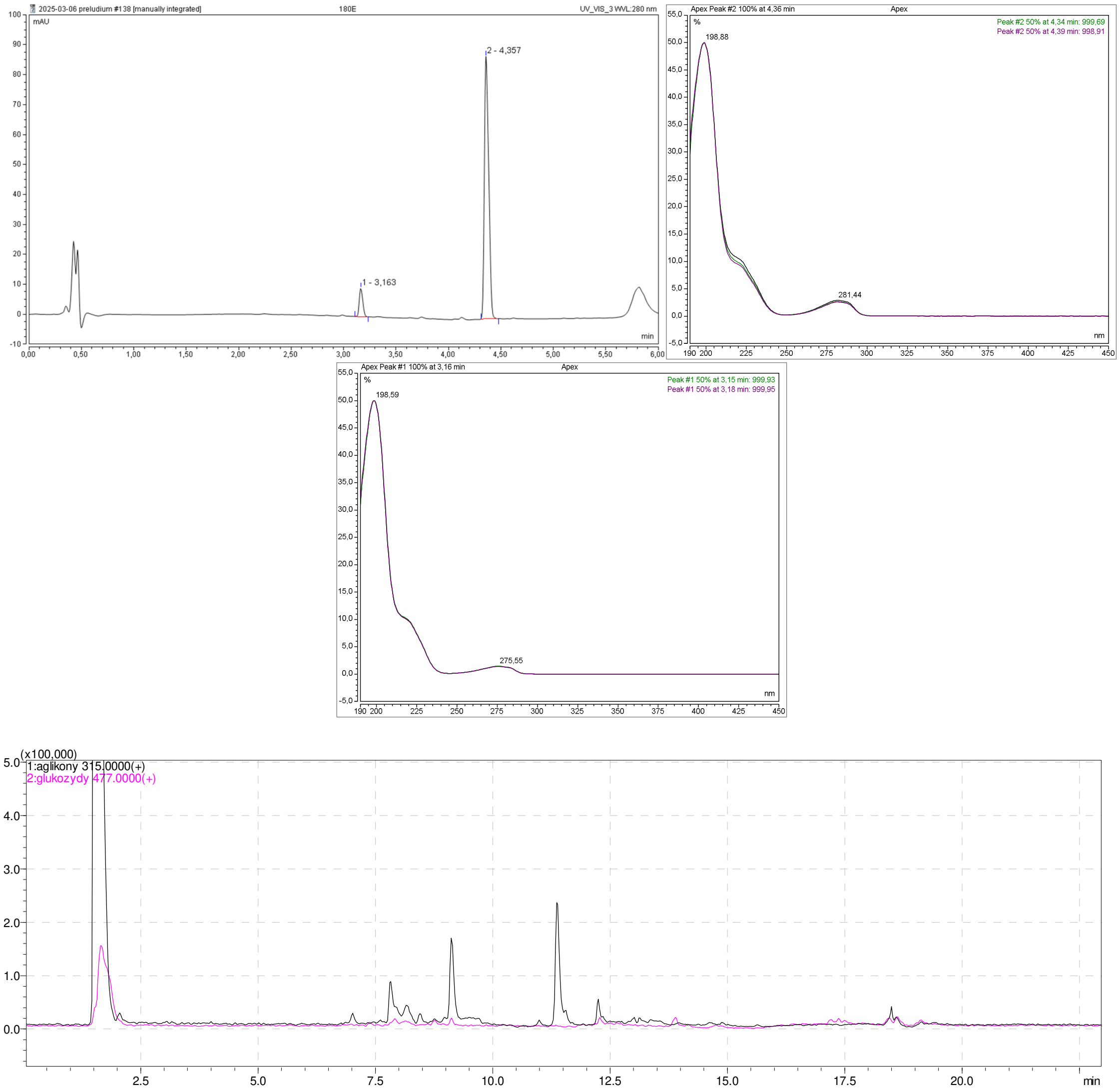

Figure S74. UPLC-DAD and LC-MS analysis of glucosylation of Estradiol acetate by YjiC GT.

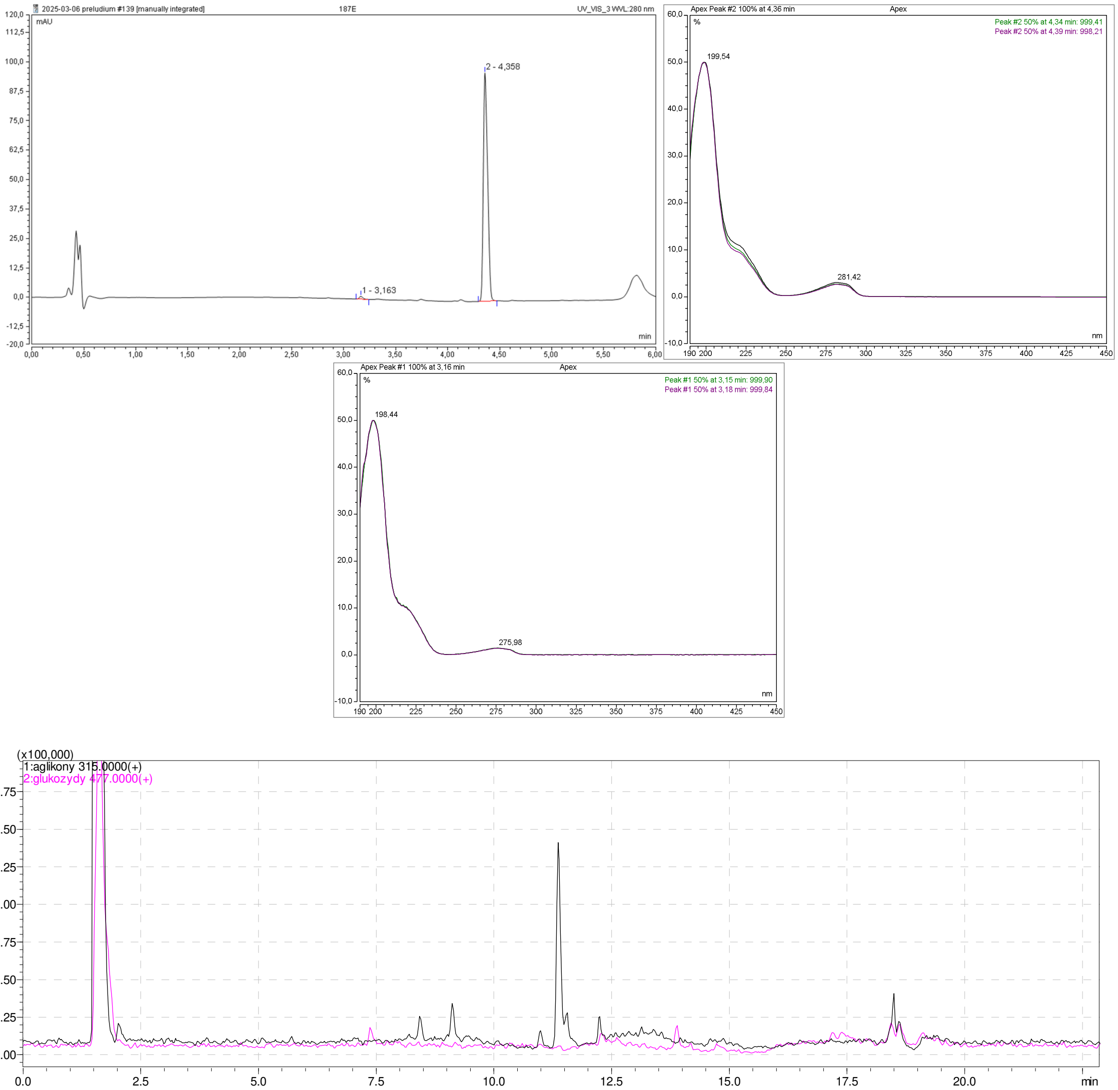

Figure S75. UPLC-DAD and LC-MS analysis of glucosylation of Estradiol acetate by OleD GT.

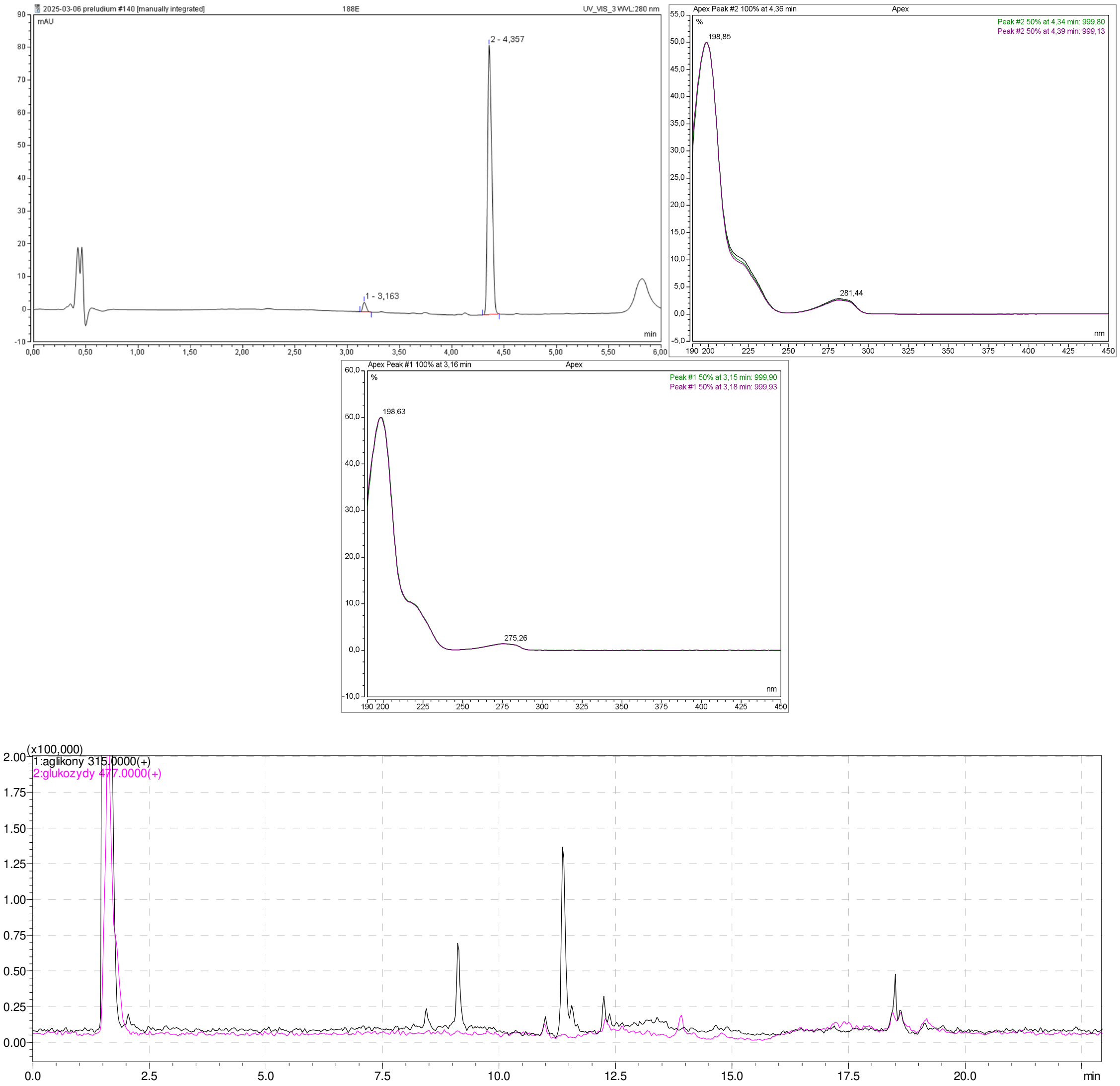

Figure S76. UPLC-DAD and LC-MS analysis of glucosylation of Estradiol acetate by Sbaic7OGT GT.

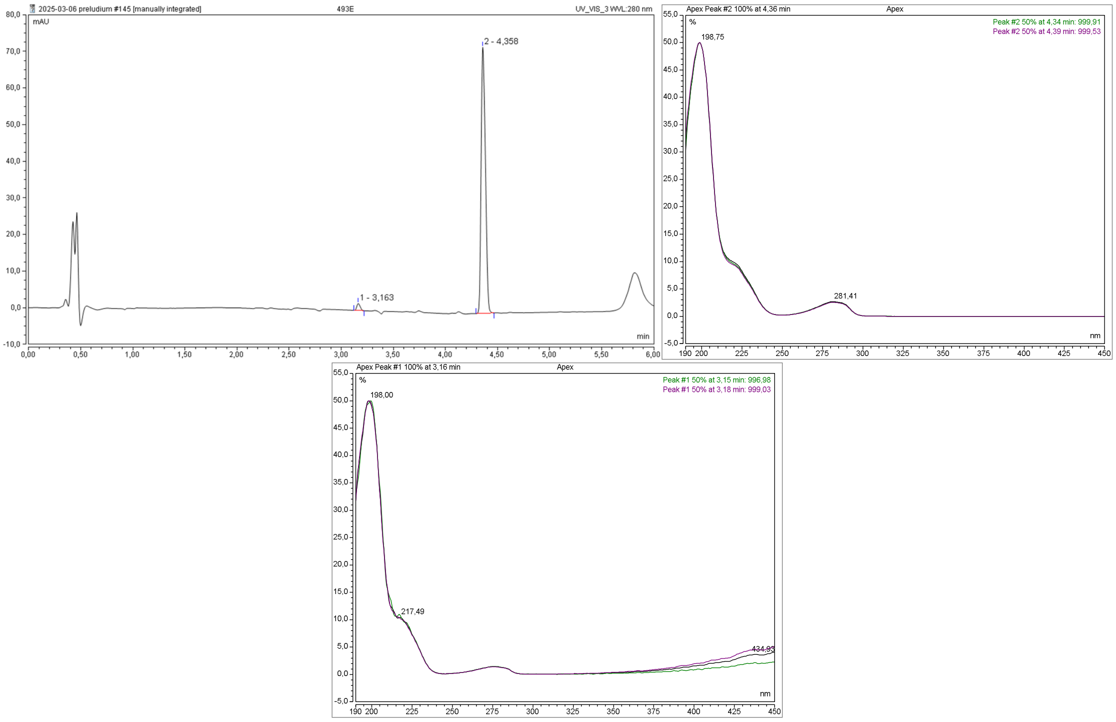

Figure S77. UPLC-DAD analysis of glucosylation of Estradiol acetate by SgUGT74C1_M7 GT.

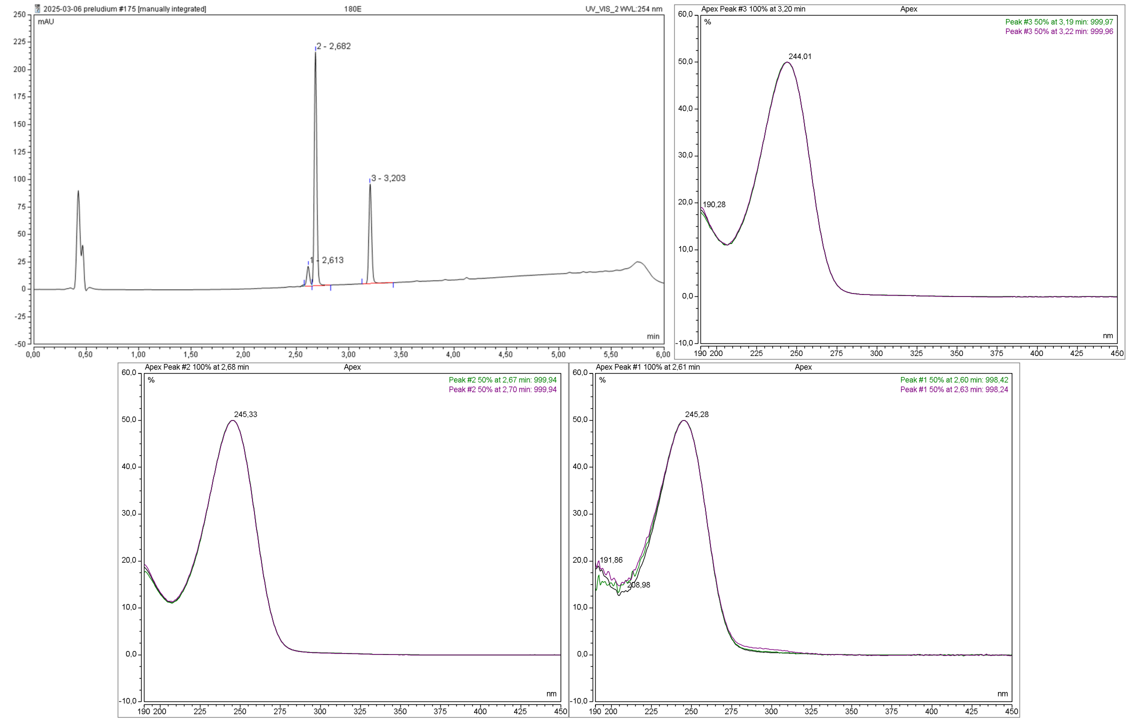

Figure S78. UPLC-DAD analysis of glucosylation of 11α-Hydroxyprogesterone by YjiC GT. Product proportions: 8.6-91.4%

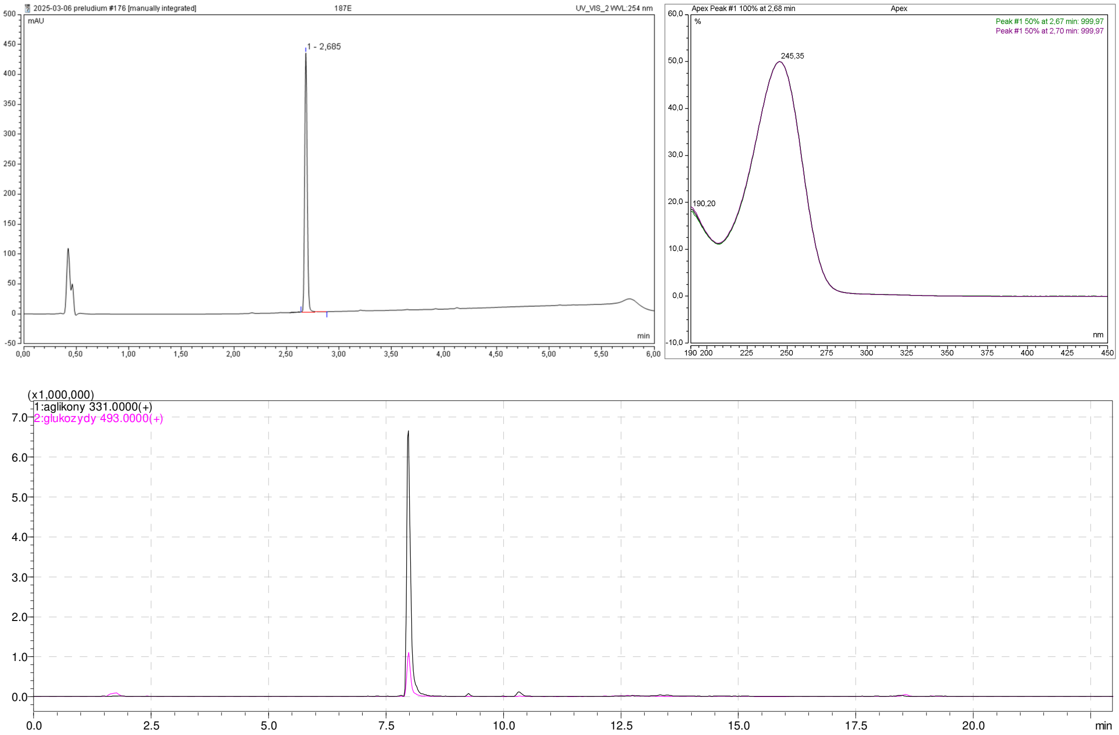

Figure S79. UPLC-DAD and LC-MS analysis of glucosylation of 11α-Hydroxyprogesterone by OleD GT.

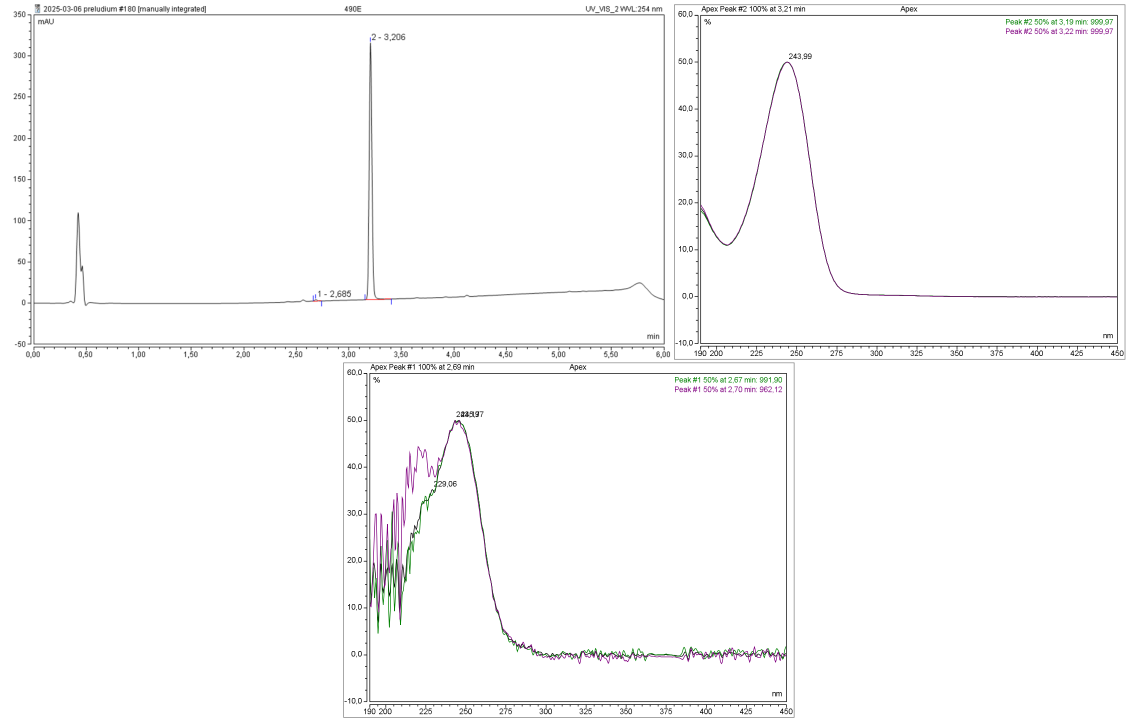

Figure S80. UPLC-DAD analysis of glucosylation of 11α-Hydroxyprogesterone by CngUGT GT.

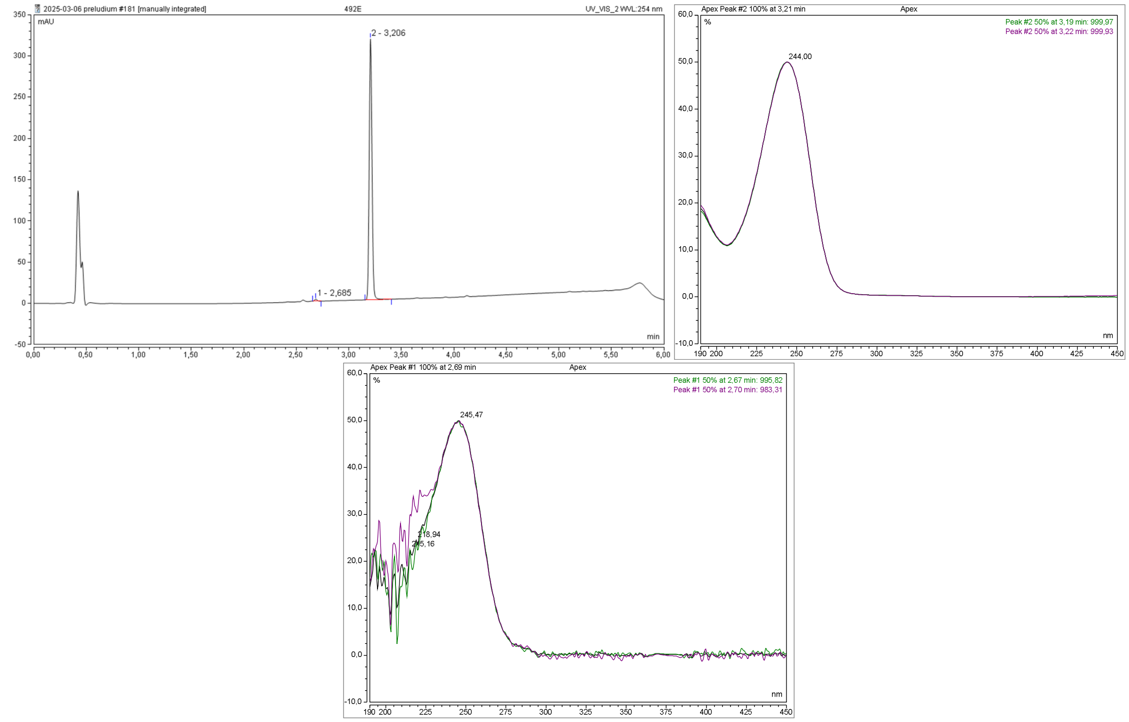

Figure S81. UPLC-DAD analysis of glucosylation of 11α-Hydroxyprogesterone by GtfC GT.

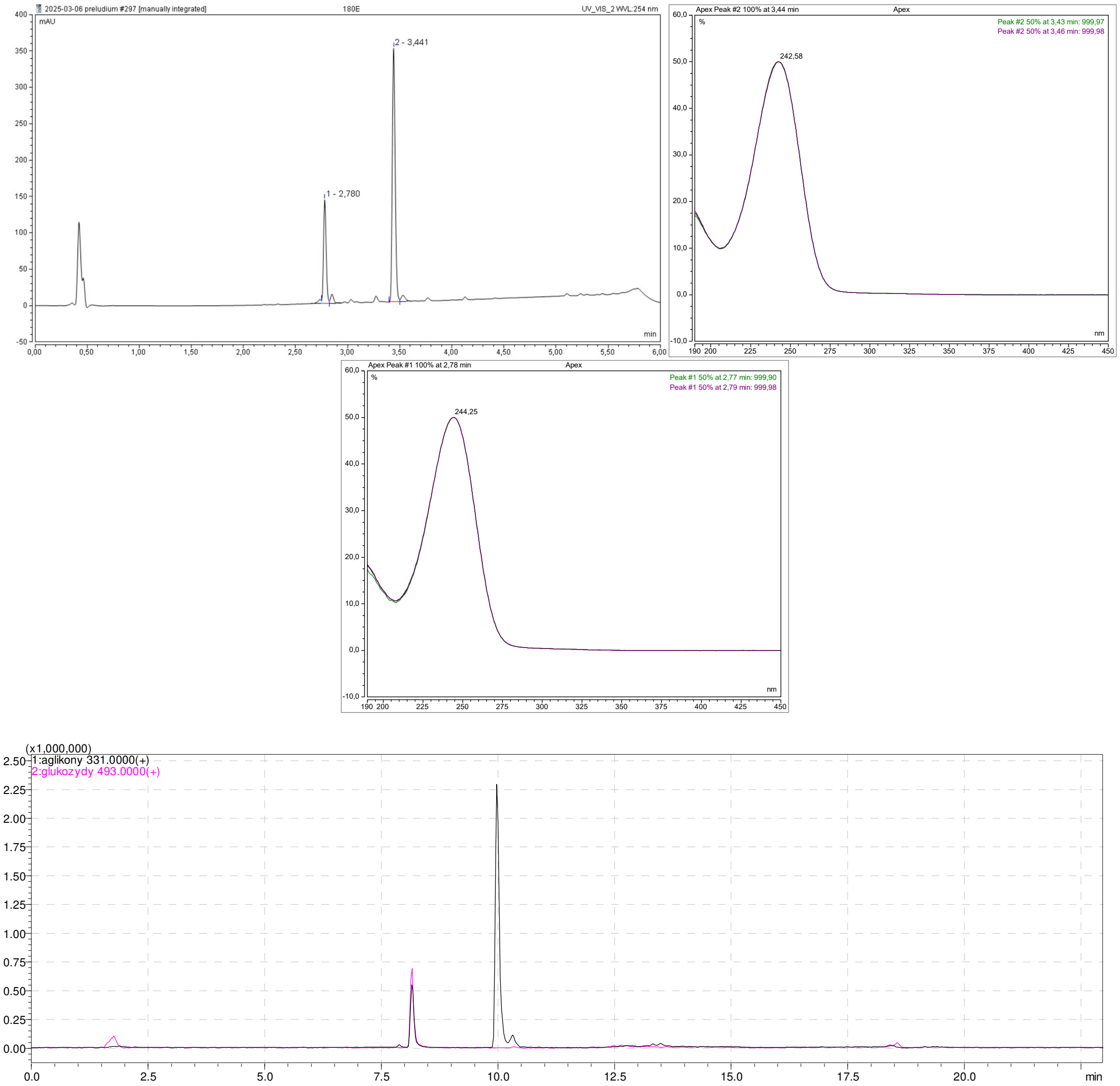

Figure S82. UPLC-DAD and LC-MS analysis of glucosylation of 21-Hydroxyprogesterone by YjiC GT.

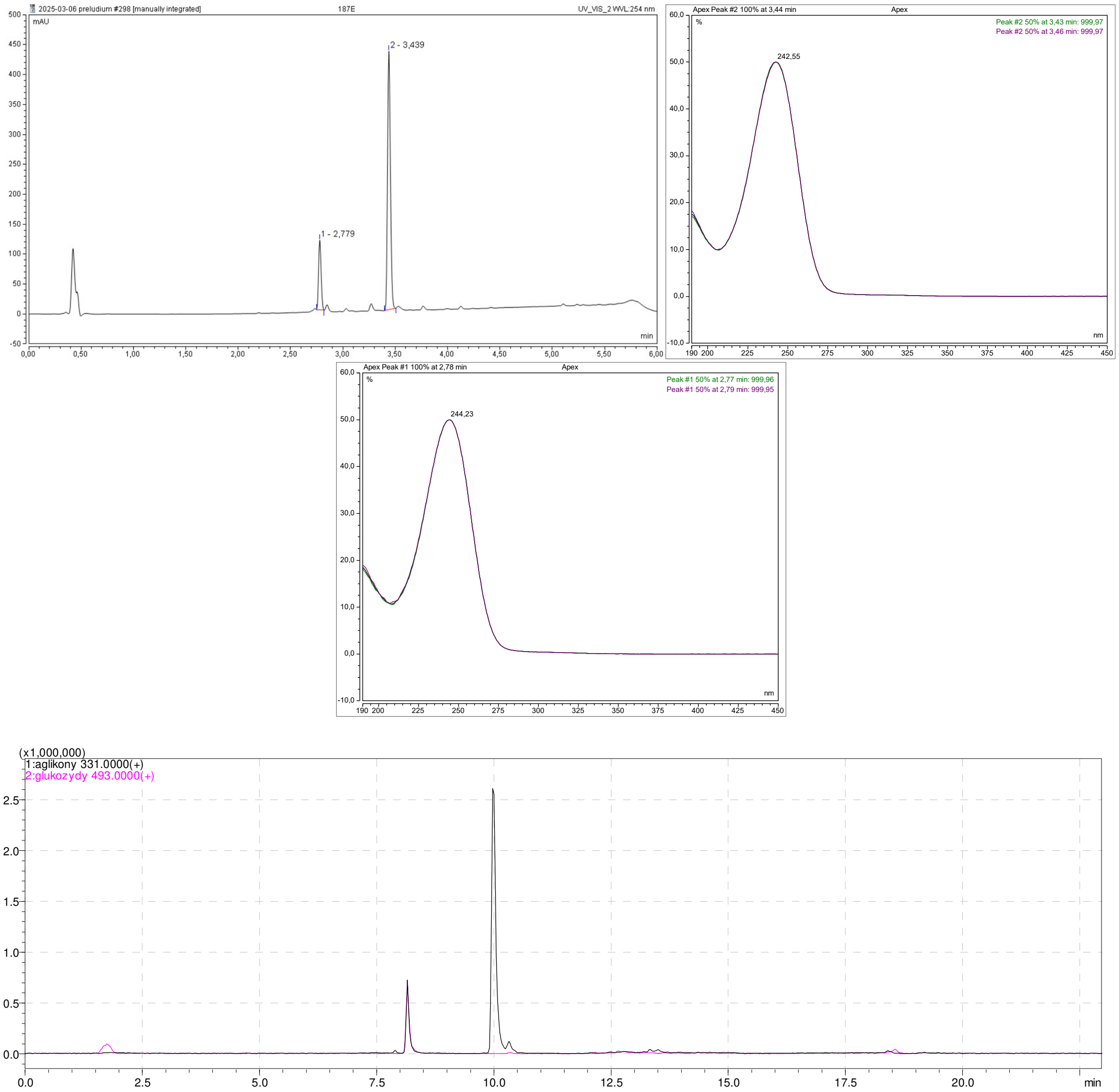

Figure S83. UPLC-DAD and LC-MS analysis of glucosylation of 21-Hydroxyprogesterone by OleD GT.

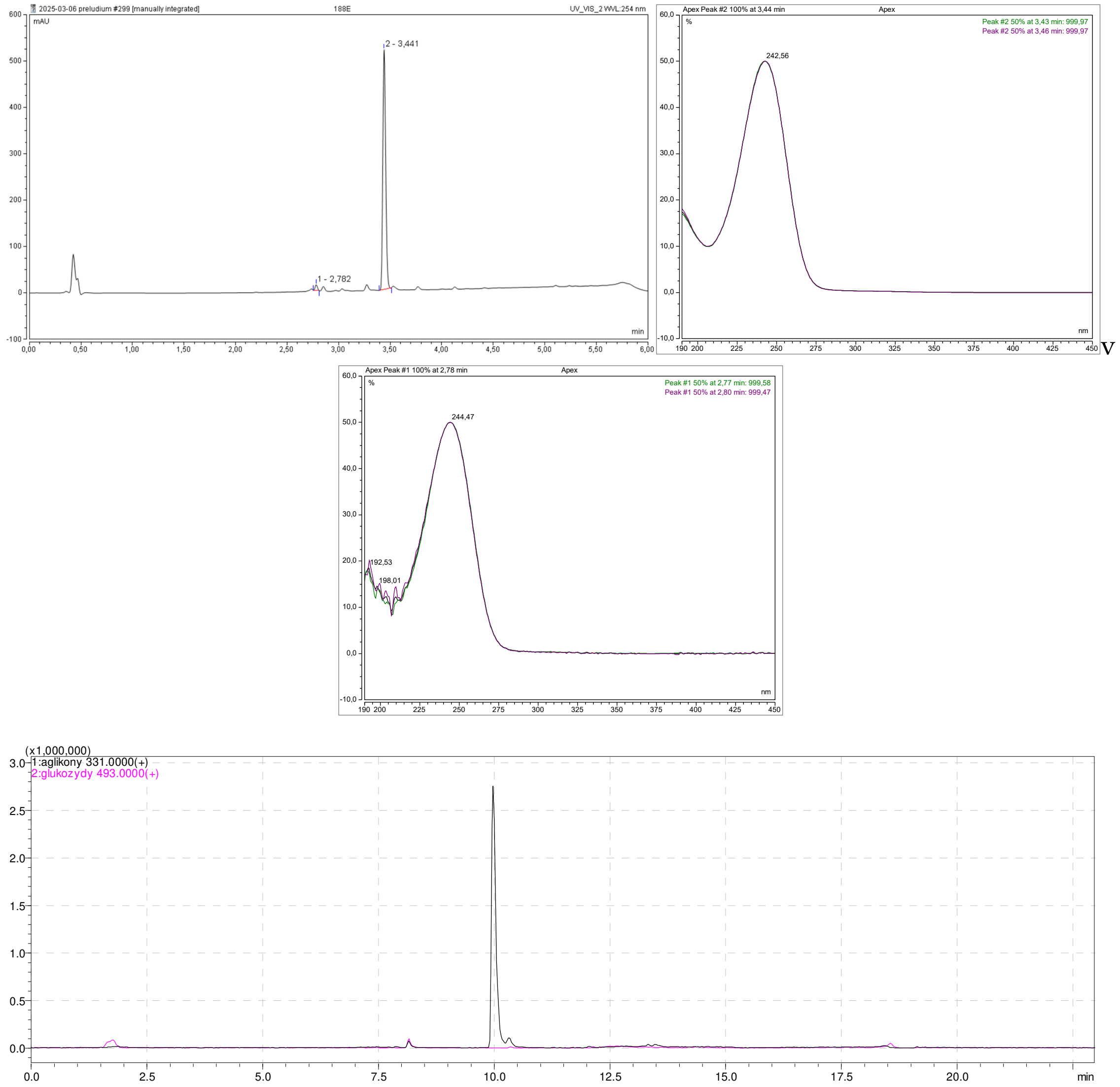

Figure S84. UPLC-DAD and LC-MS analysis of glucosylation of 21-Hydroxyprogesterone by Sbaic7OGT GT.

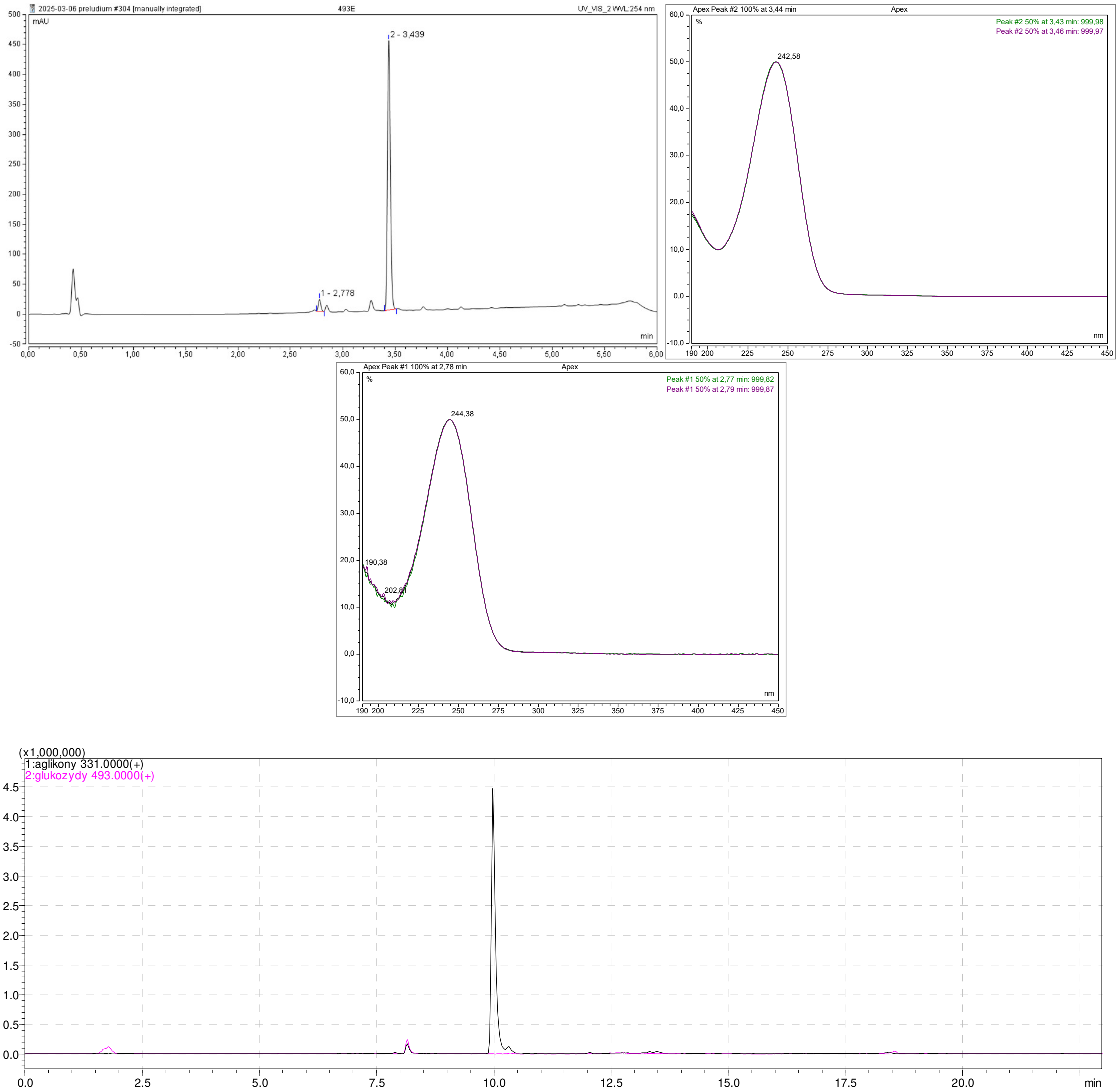

Figure S85. UPLC-DAD and LC-MS analysis of glucosylation of 21-Hydroxyprogesterone by SgUGT74AC1_M7 GT.

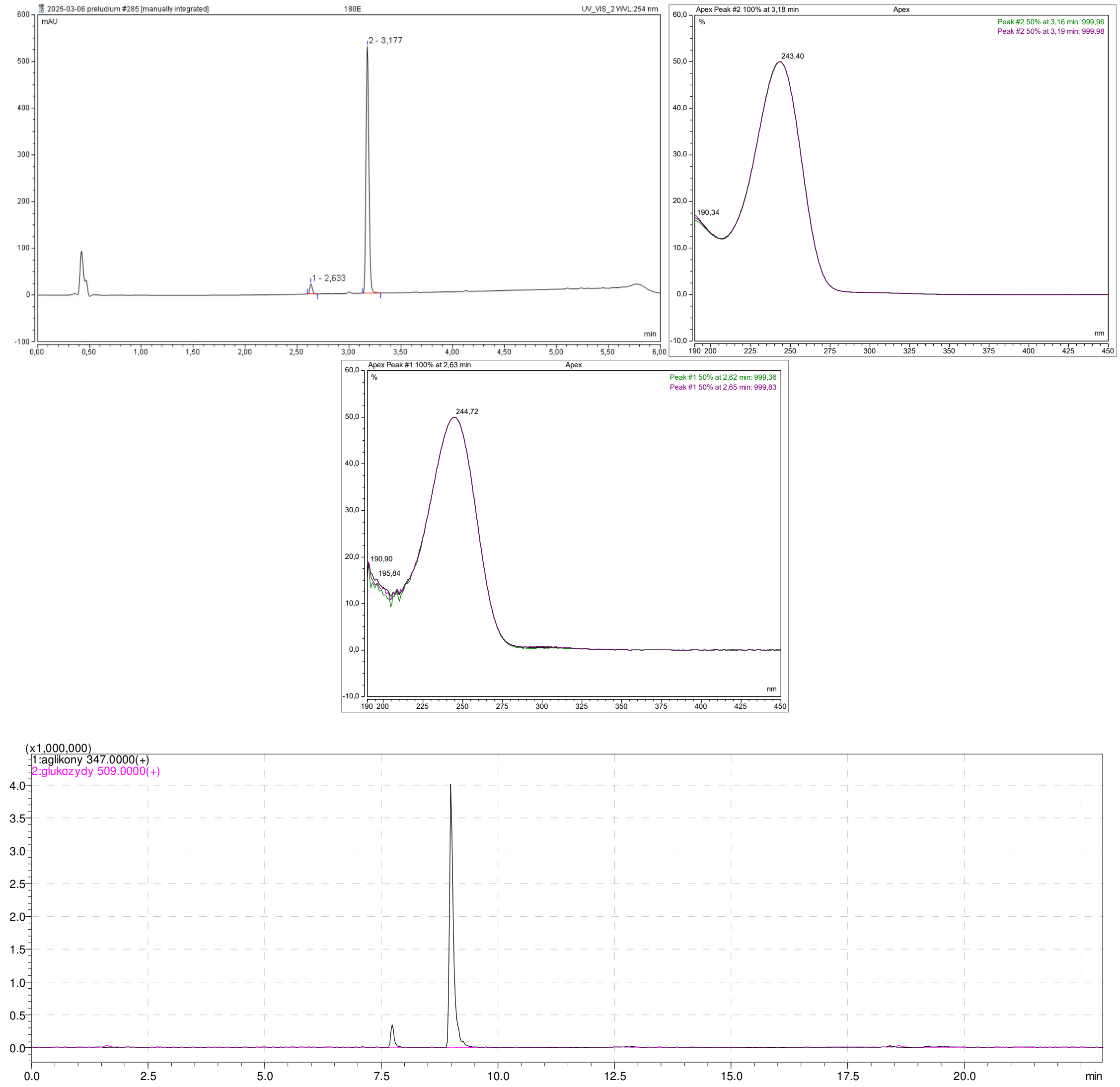

Figure S86. UPLC-DAD and LC-MS analysis of glucosylation of 17α,21-Dihydroxyprogesterone by YjiC GT.

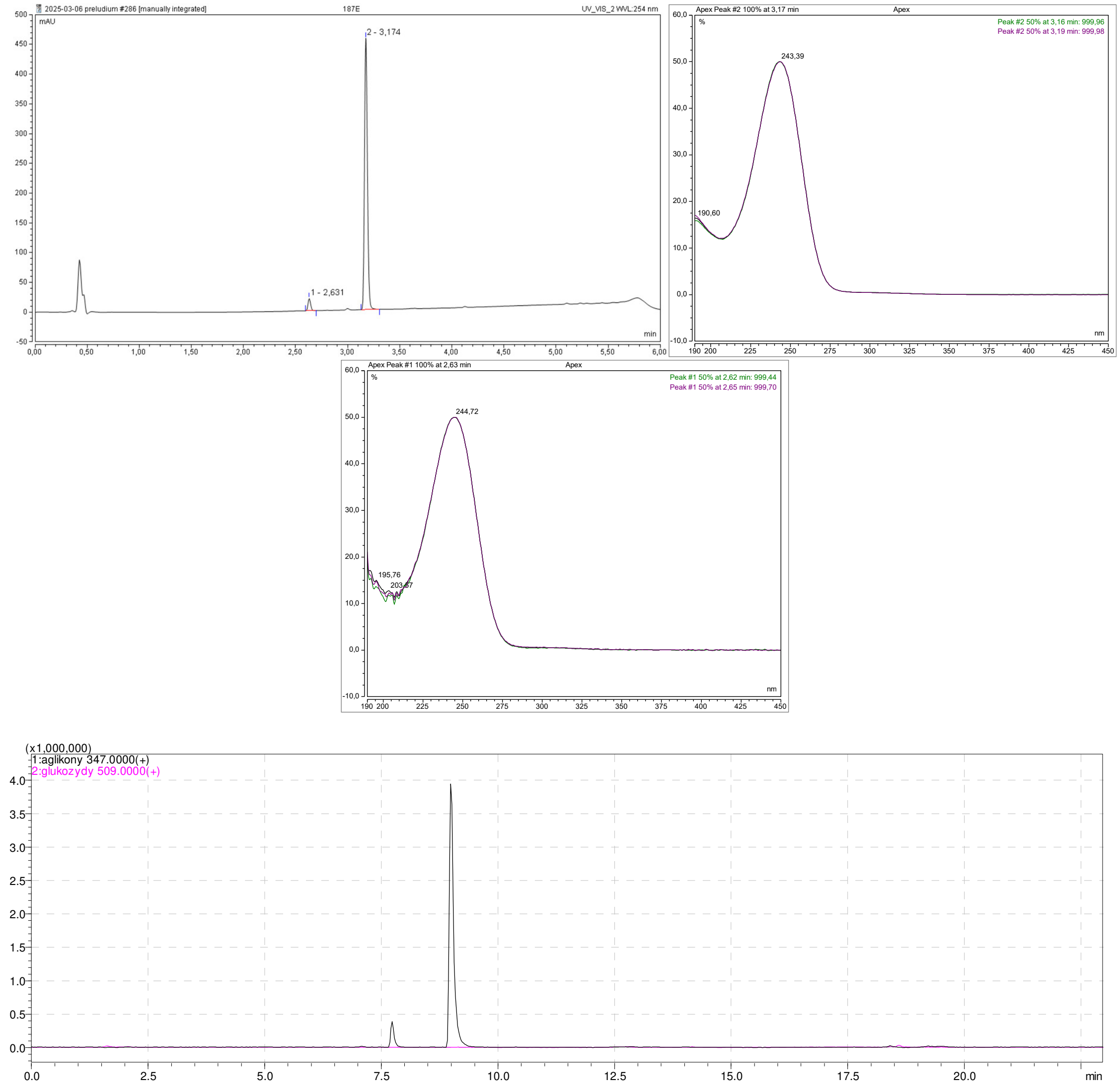

Figure S87. UPLC-DAD and LC-MS analysis of glucosylation of 17α,21-Dihydroxyprogesterone by OleD GT.

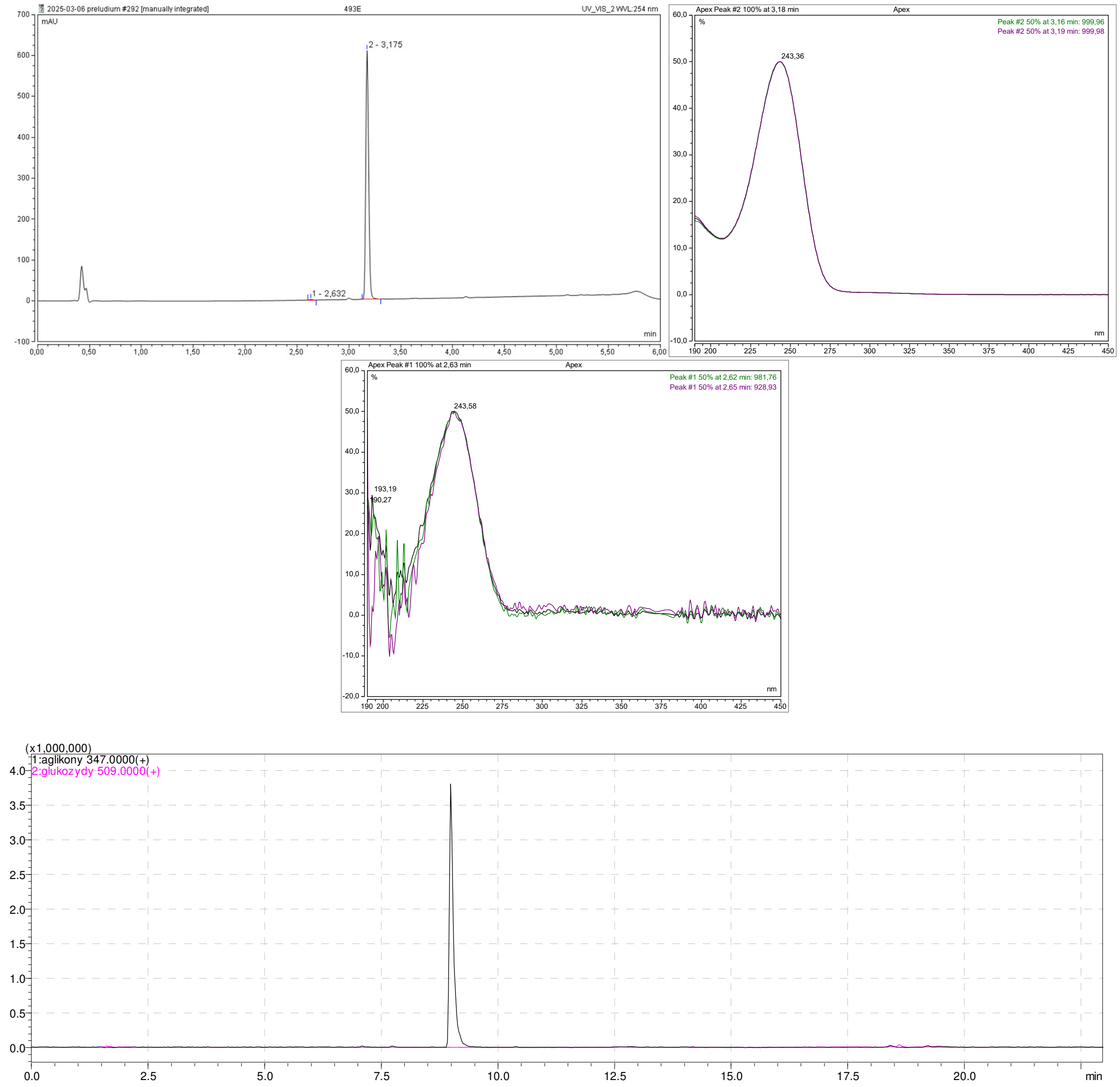

Figure S88. UPLC-DAD and LC-MS analysis of glucosylation of 17α,21-Dihydroxyprogesterone by SgUGT74AC1_M7 GT.

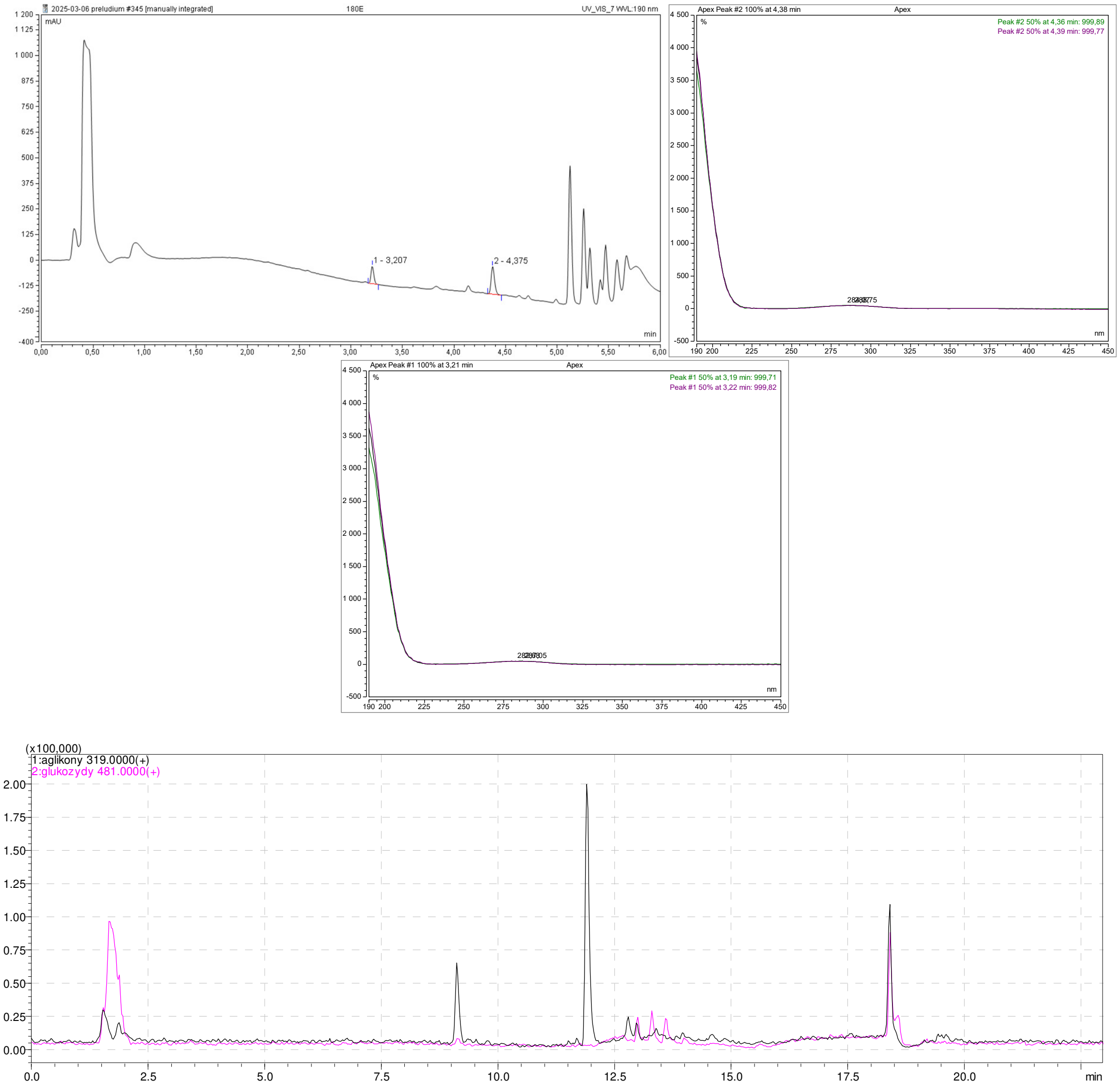

Figure S89. UPLC-DAD and LC-MS analysis of glucosylation of Allopregnanolone by YjiC GT.

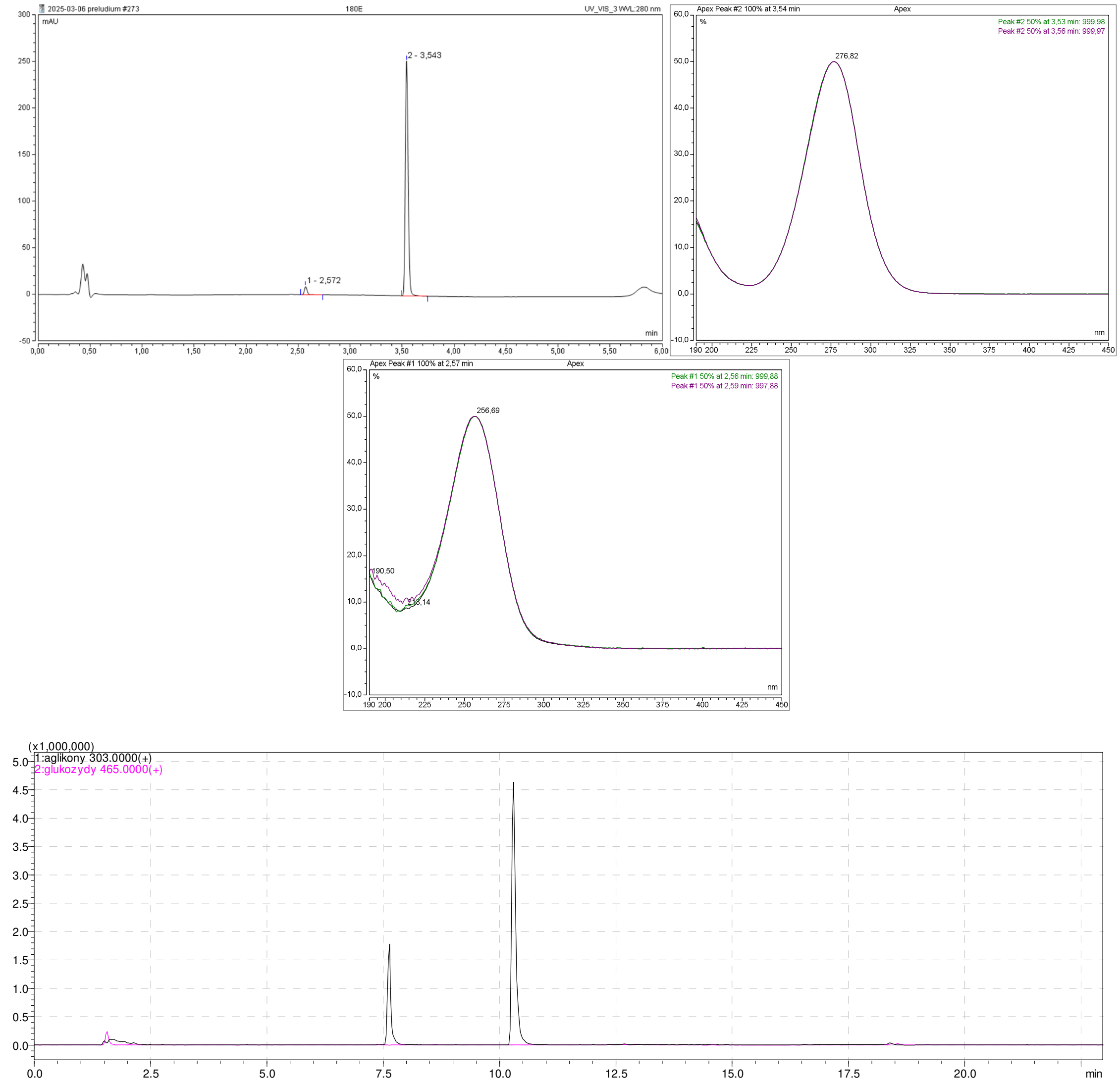

Figure S90. UPLC-DAD and LC-MS analysis of glucosylation of Formestane by YjiC GT.

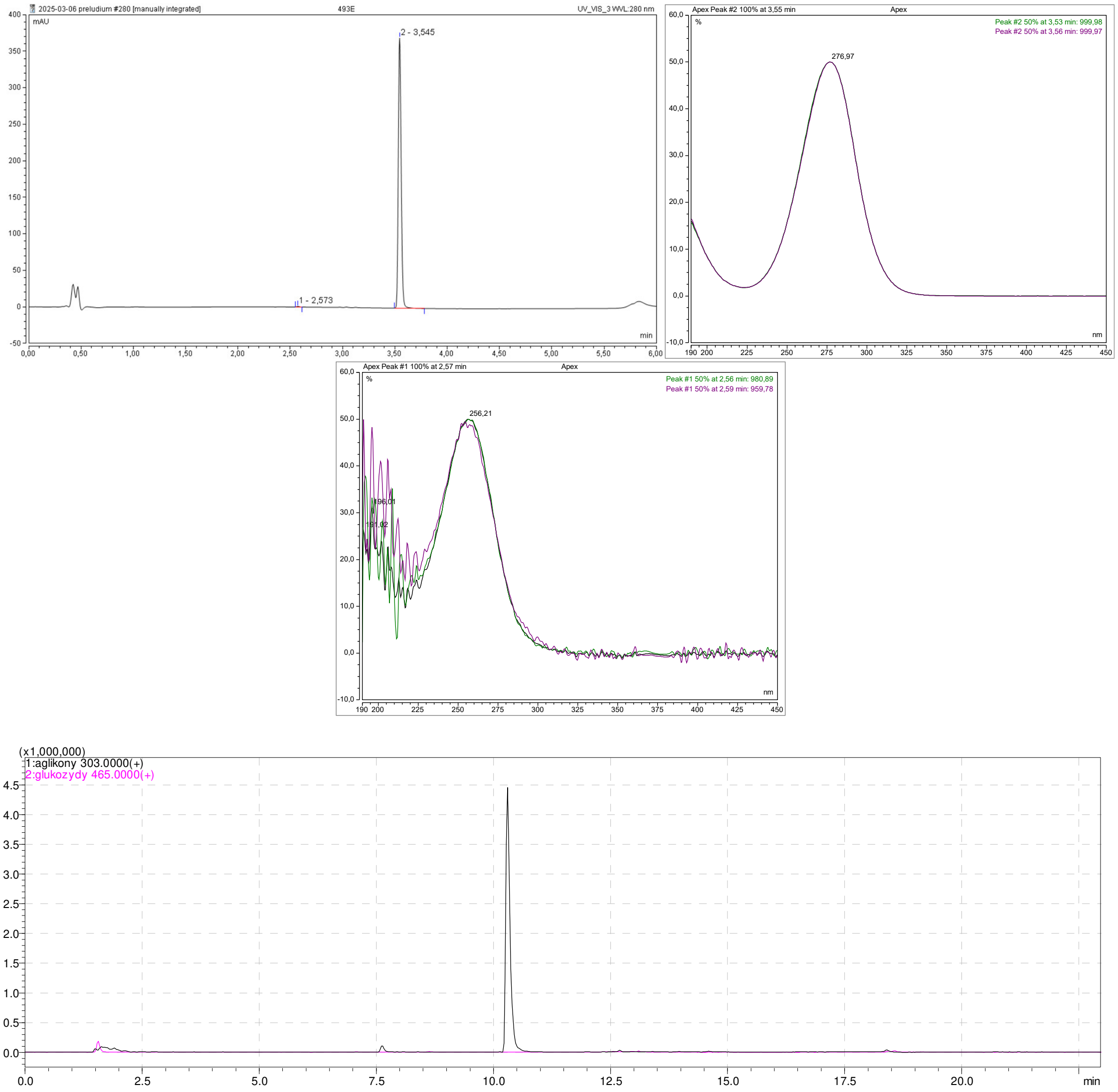

Figure S91. UPLC-DAD and LC-MS analysis of glucosylation of Formestane by SgUGT74AC1_M7 GT.

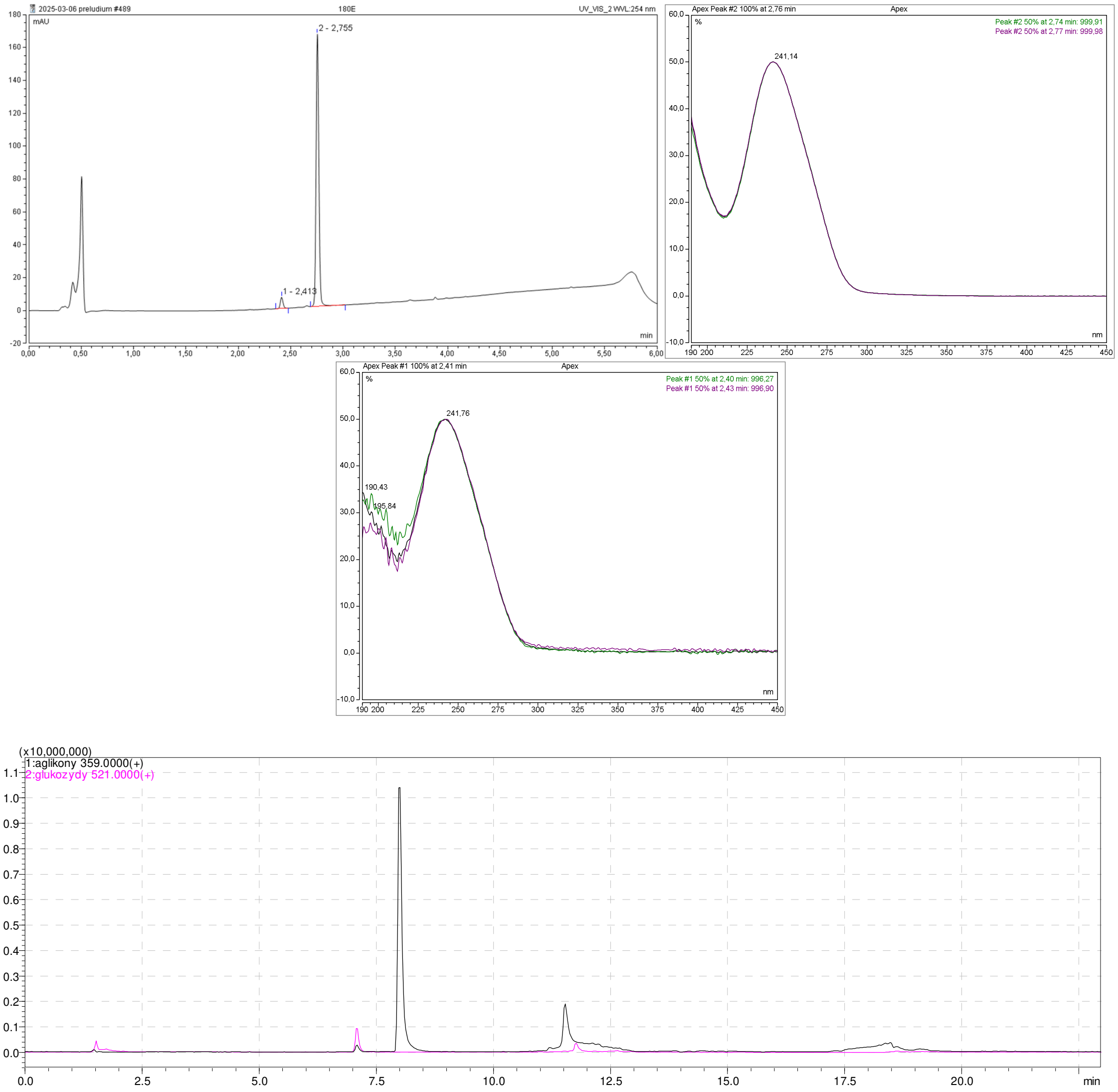

Figure S92. UPLC-DAD and LC-MS analysis of glucosylation of Prednisone by YjiC GT.

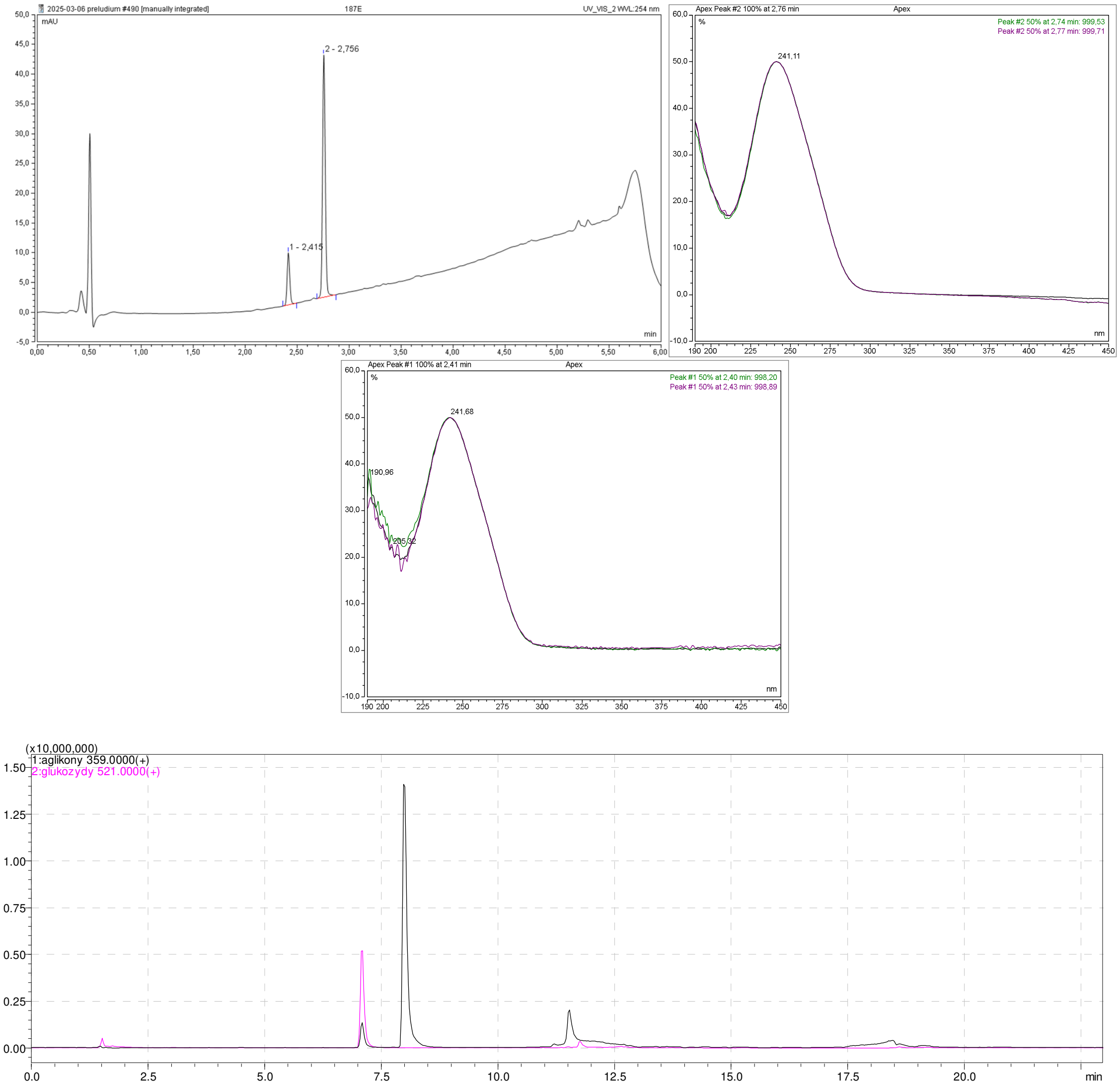

Figure S93. UPLC-DAD and LC-MS analysis of glucosylation of Prednisone by OleD GT.

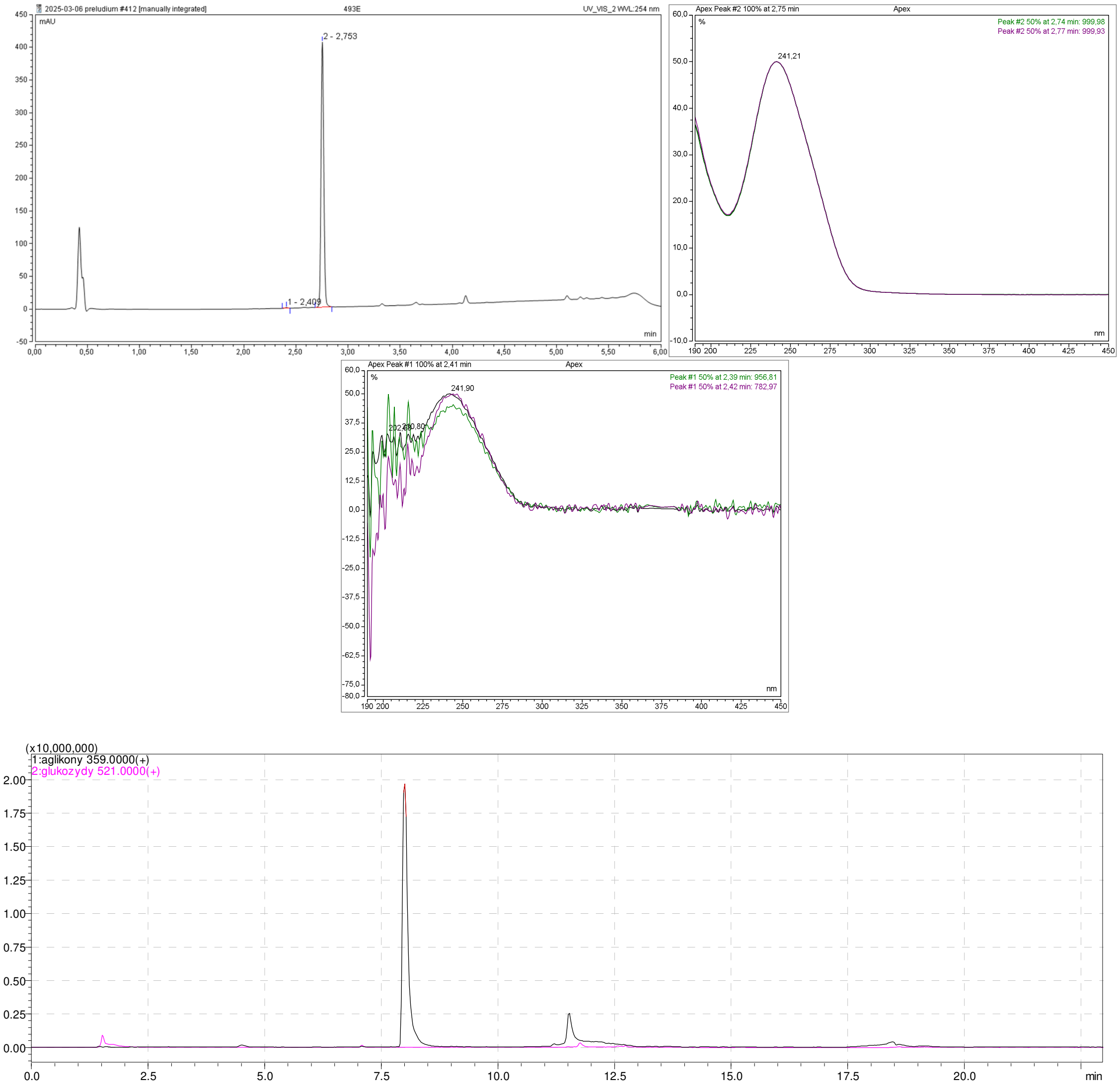

Figure S94. UPLC-DAD and LC-MS analysis of glucosylation of Prednisone by SgUGT74AC1_M7 GT.

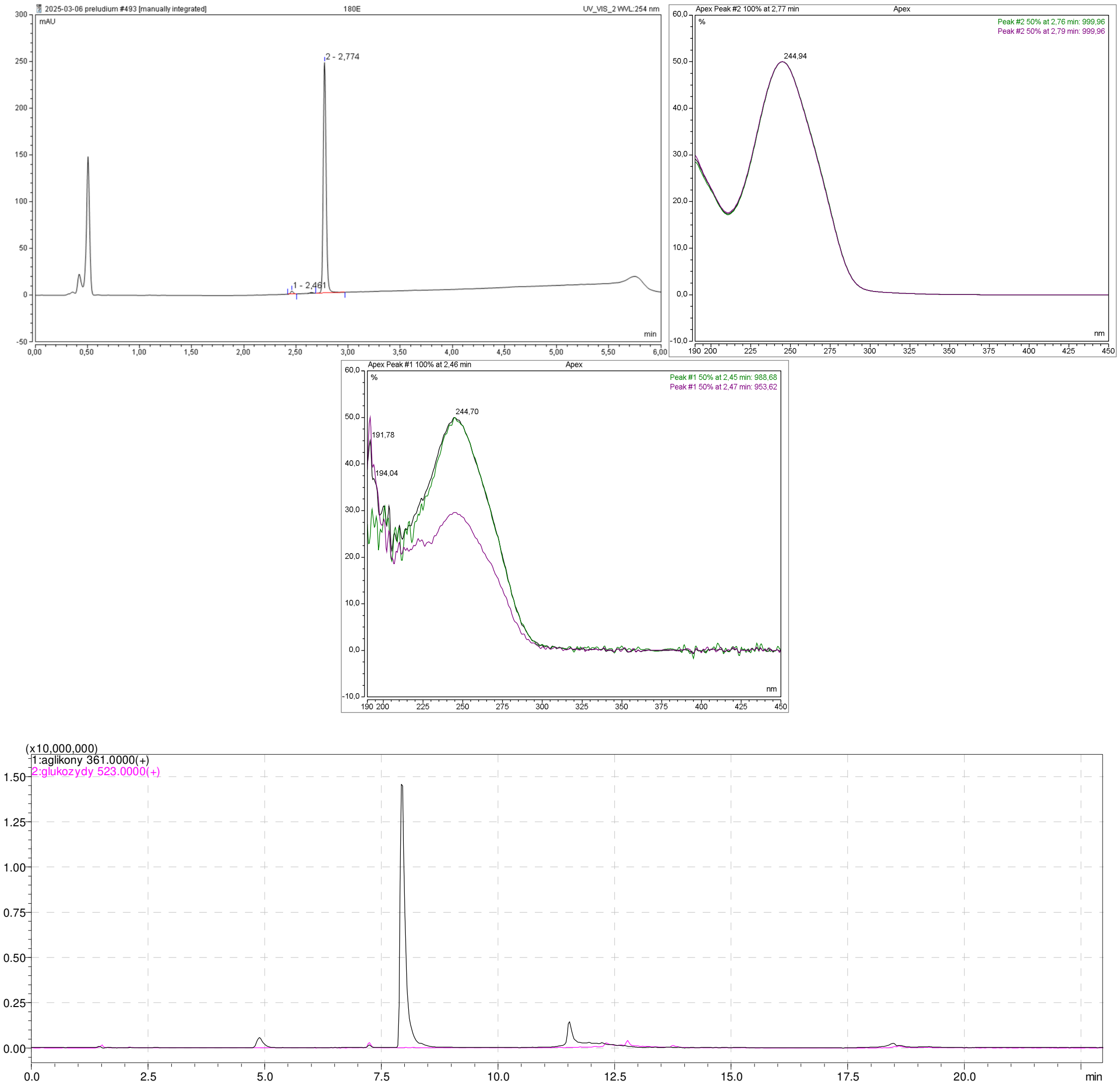

Figure S95. UPLC-DAD and LC-MS analysis of glucosylation of Prednisolone by YjiC GT.

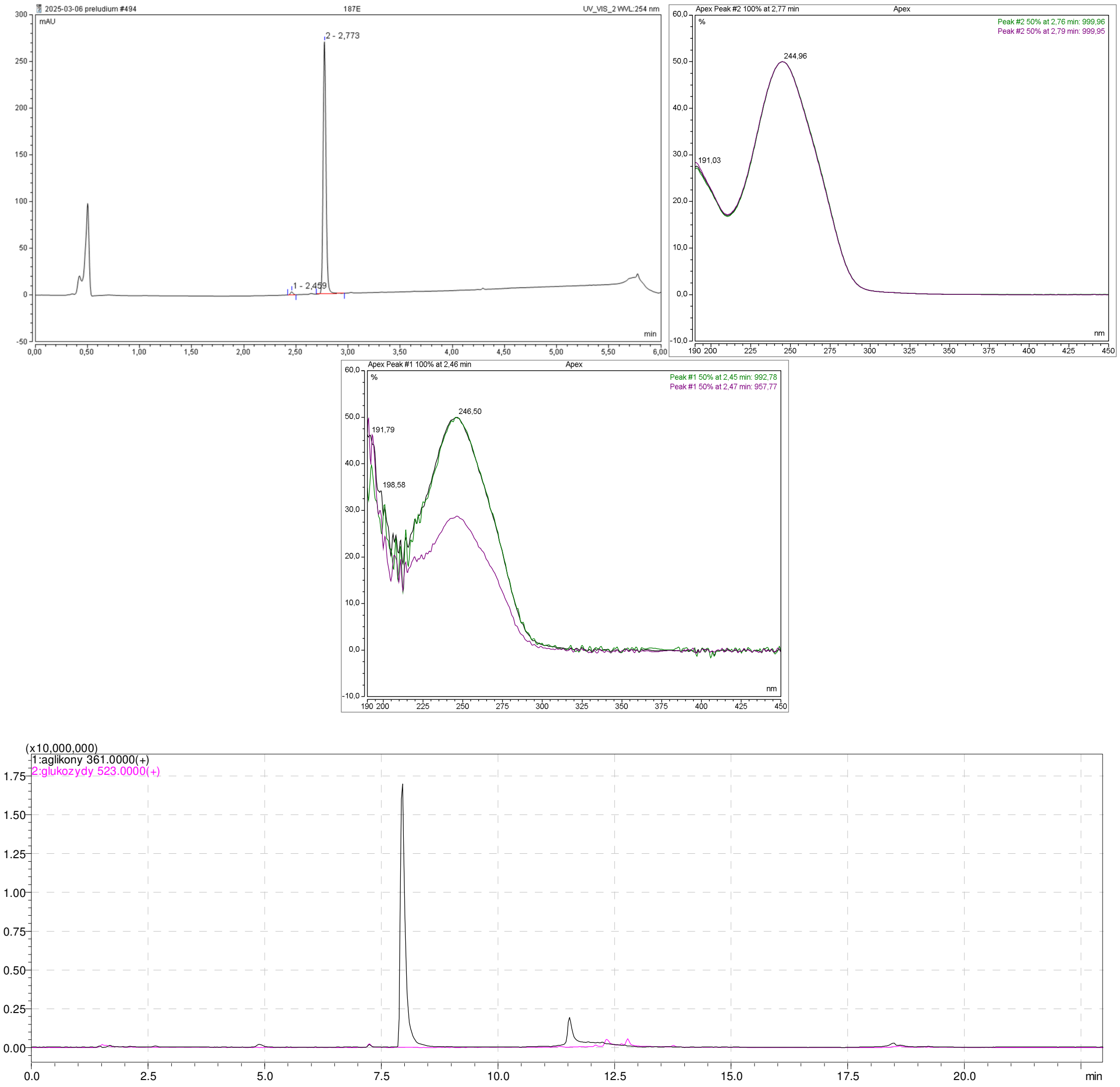

Figure S96. UPLC-DAD and LC-MS analysis of glucosylation of Prednisolone by OleD GT.

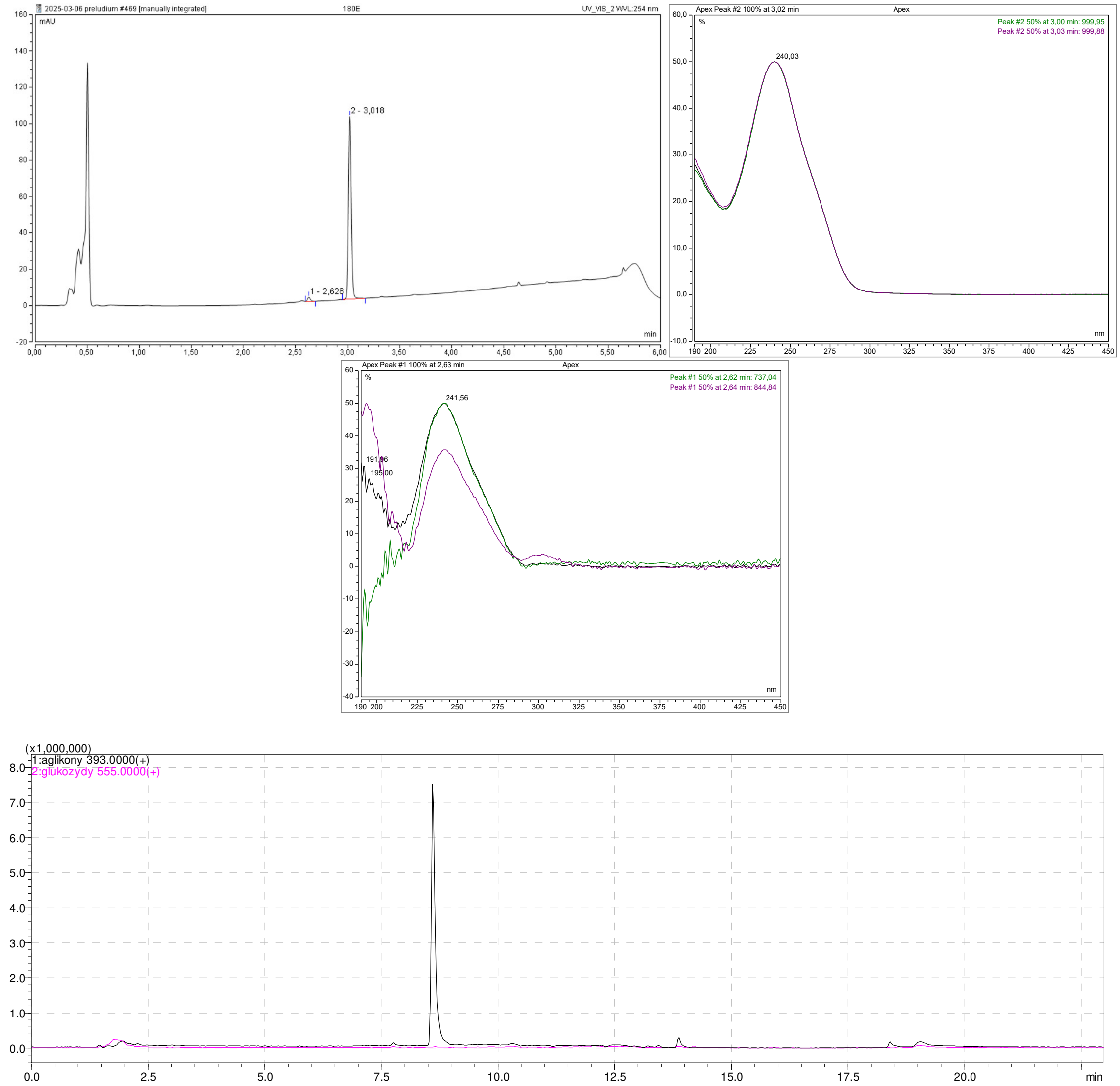

Figure S97. UPLC-DAD and LC-MS analysis of glucosylation of Dexamethasone by YjiC GT.

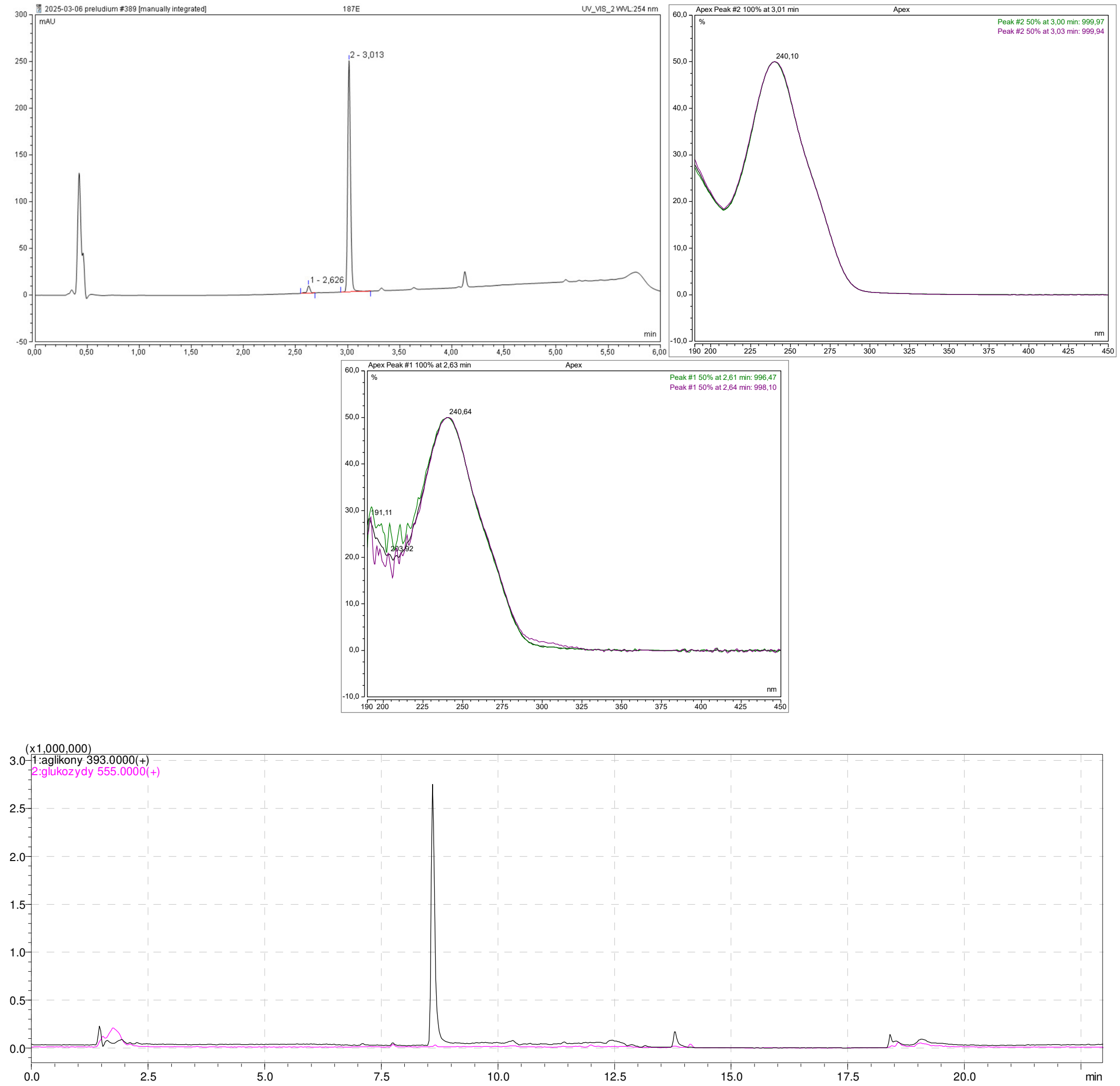

Figure S98. UPLC-DAD and LC-MS analysis of glucosylation of Dexamethasone by OleD GT.

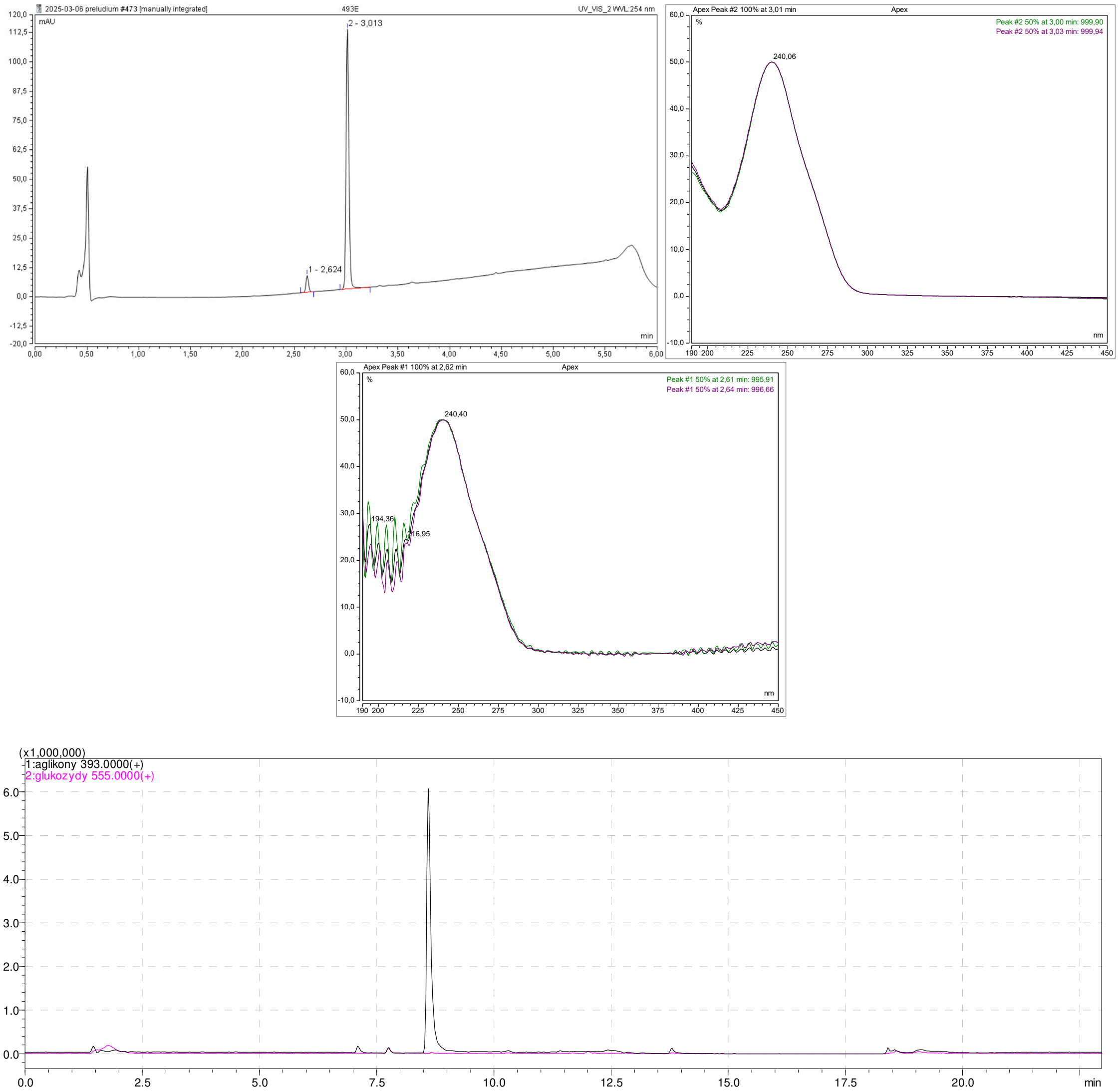

Figure S99. UPLC-DAD and LC-MS analysis of glucosylation of Dexamethasone by SgUGT74AC1_M7 GT.

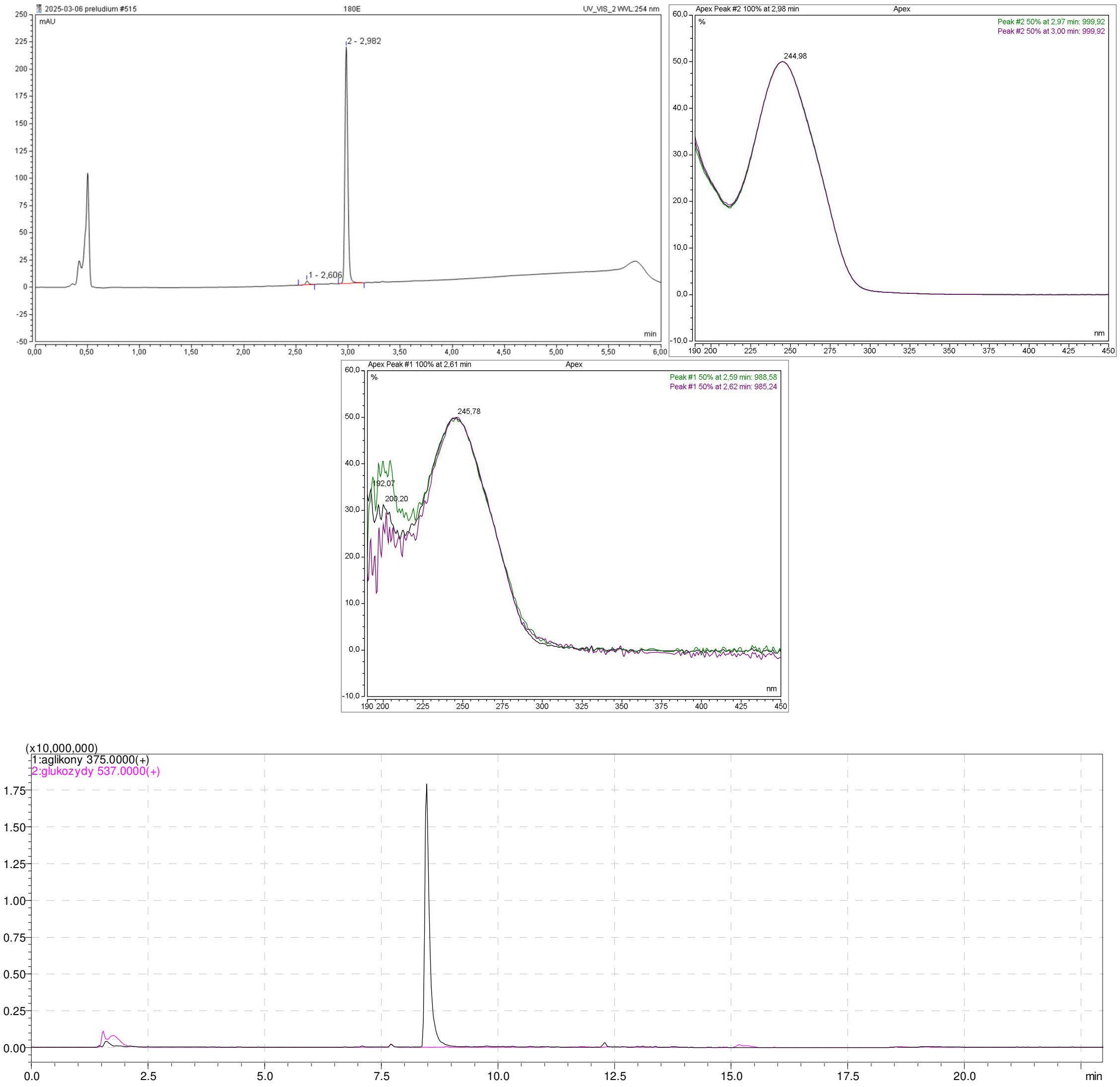

Figure S100. UPLC-DAD and LC-MS analysis of glucosylation of 6α-Methylprednisolone by YjiC GT.

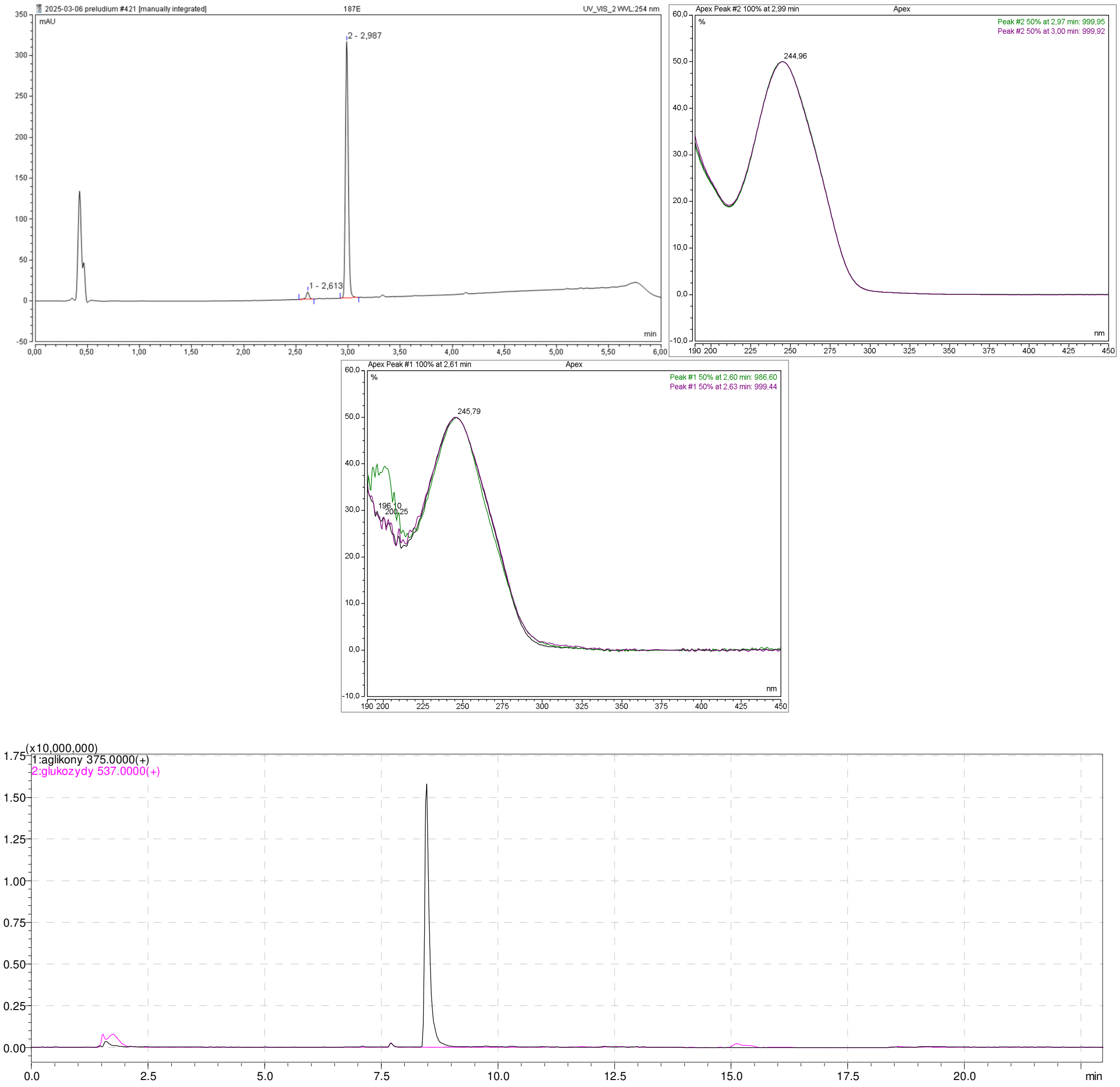

Figure S101. UPLC-DAD and LC-MS analysis of glucosylation of 6α-Methylprednisolone by OleD GT.

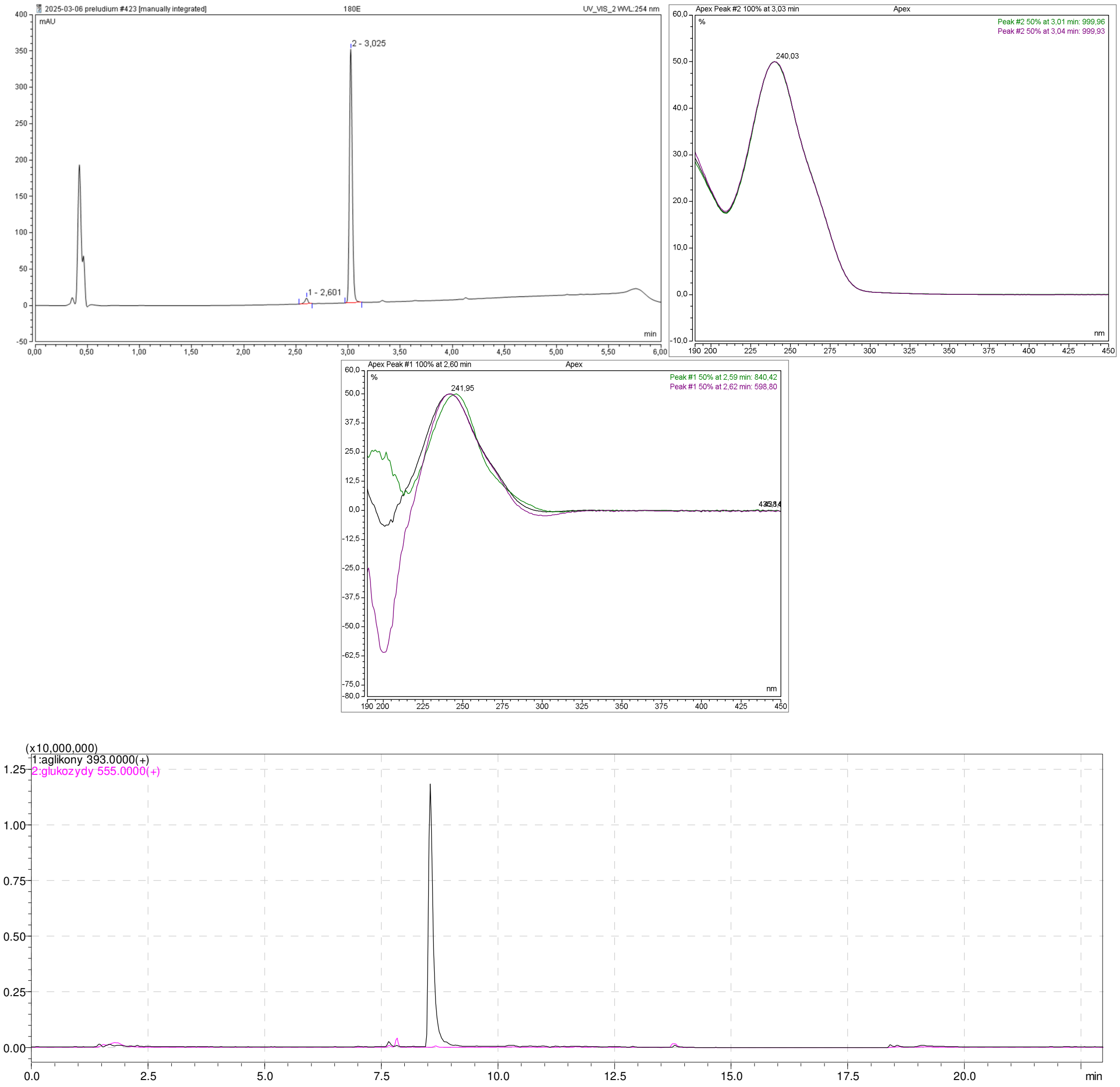

Figure S102. UPLC-DAD and LC-MS analysis of glucosylation of Betamethasone by YjiC GT.

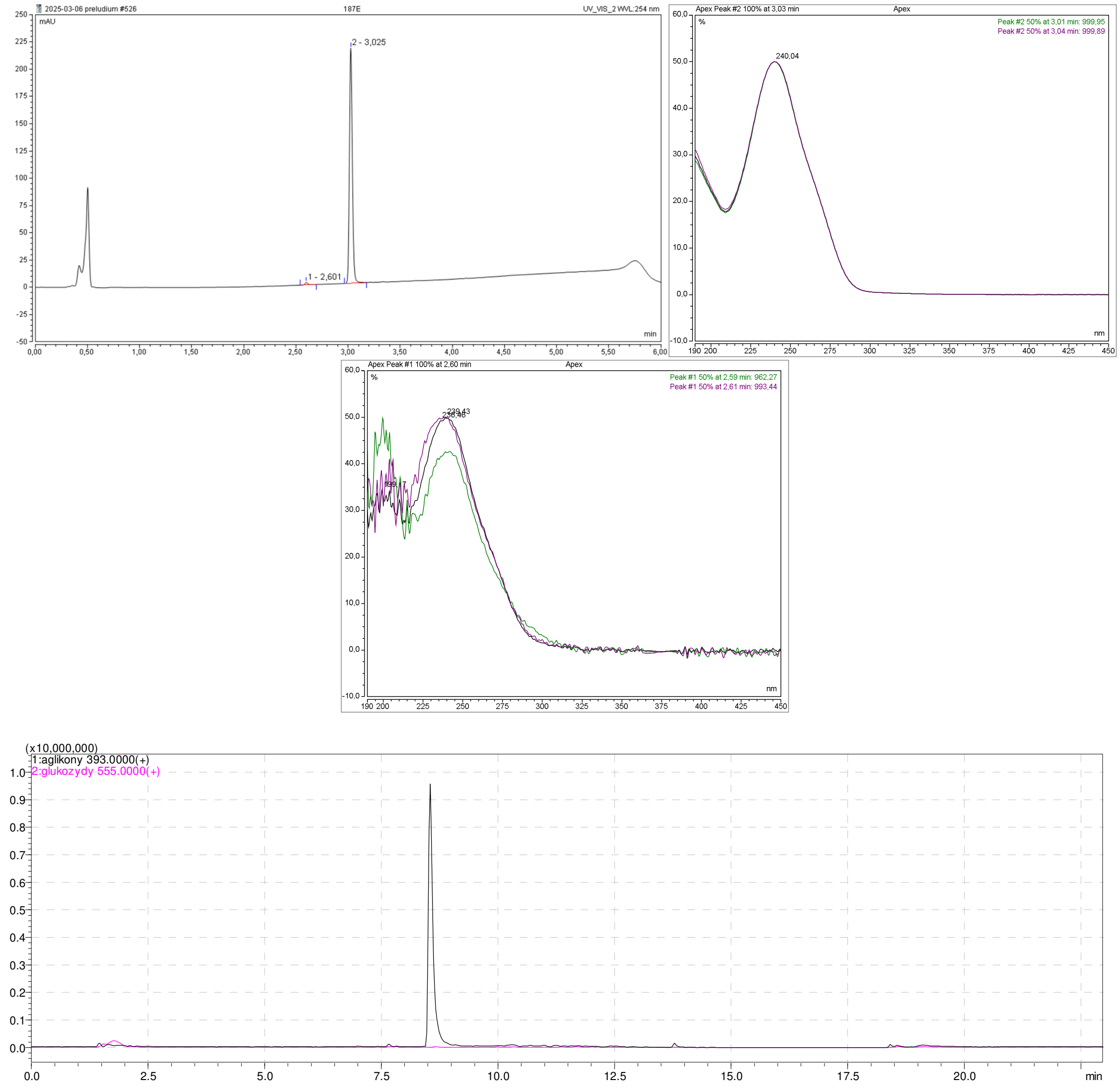

Figure S103. UPLC-DAD and LC-MS analysis of glucosylation of Betamethasone by OleD GT.

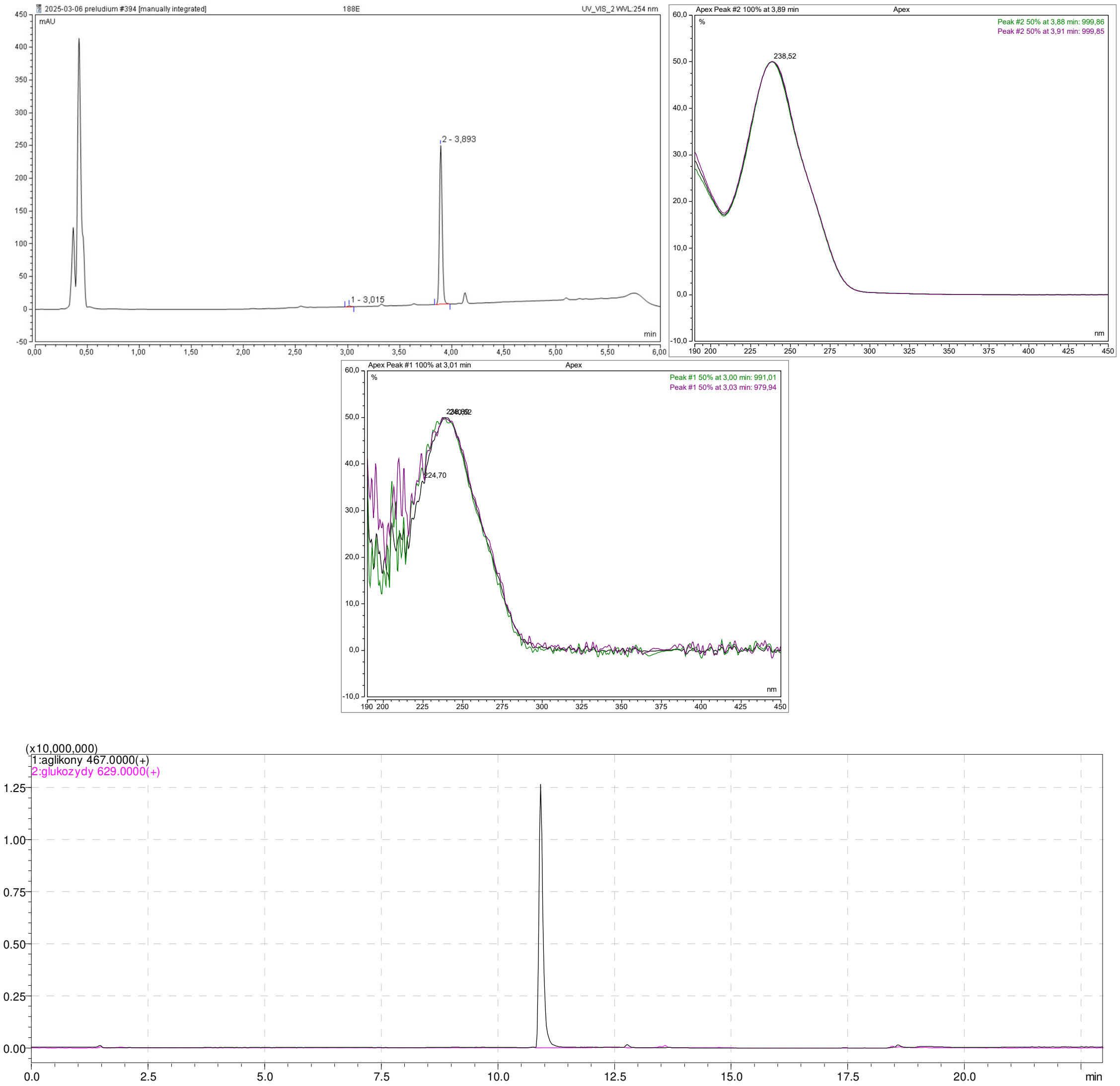

Figure S104. UPLC-DAD and LC-MS analysis of glucosylation of Clobetasol propionate by Sbaic7OGT GT.

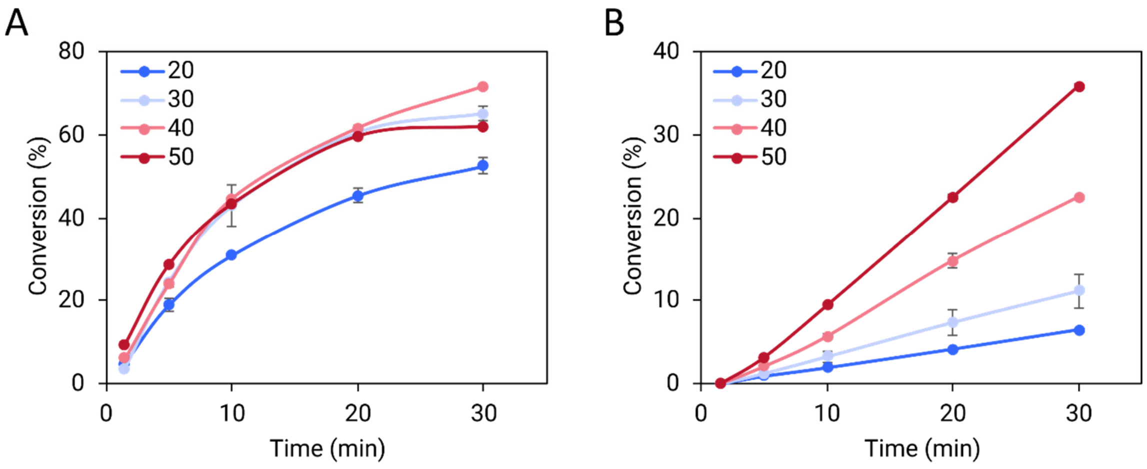

Figure S105. Time-course of (**A**) nandrolone glucosylation by OleD and (**B**) 17α-Testosterone glucosylation by SgUGT74AC1_M7 at different temperatures. Error bars represent the standard deviations obtained from three individual replicates.

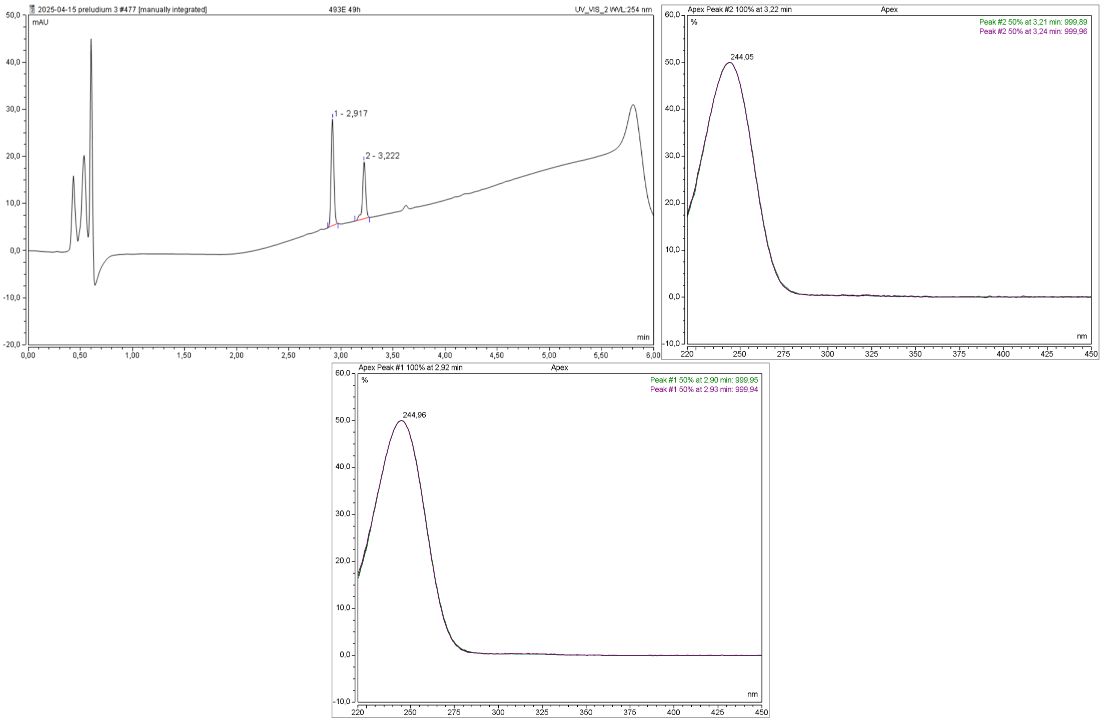

Figure S106. UPLC-DAD analysis of semi-preparative glucosylation of 17α-testosterone by SgUGT74AC1_M7 GT.

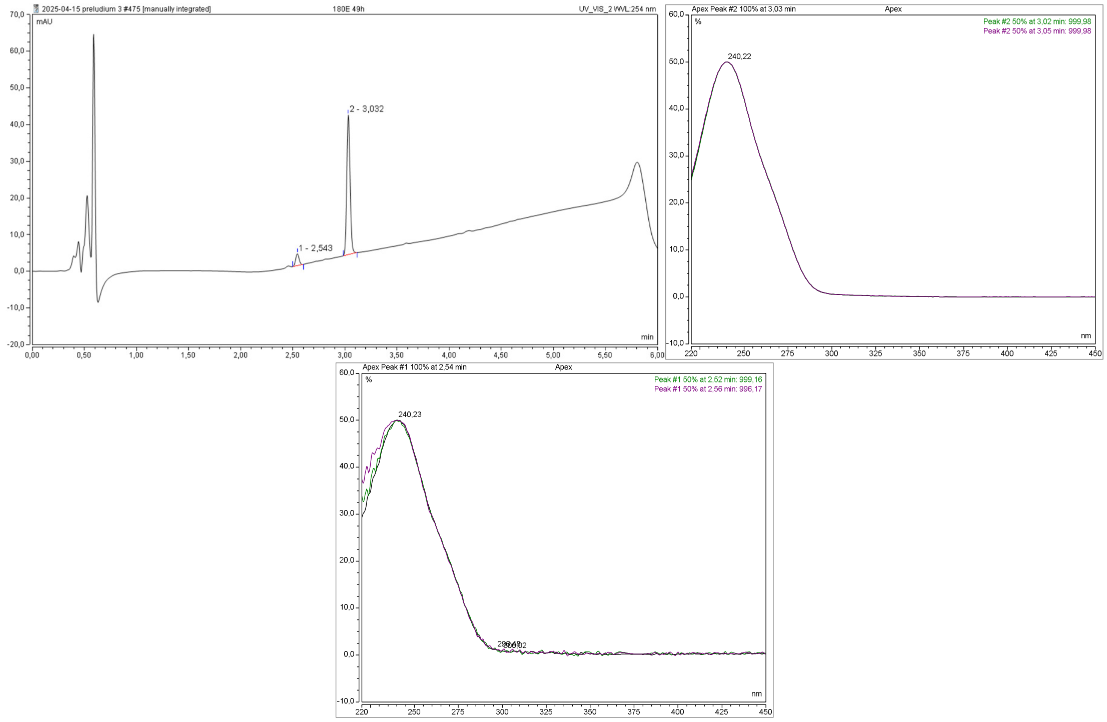

Figure S107. UPLC-DAD analysis of semi-preparative glucosylation of Betamethasone by YjiC GT.

Reference standards and NMR data

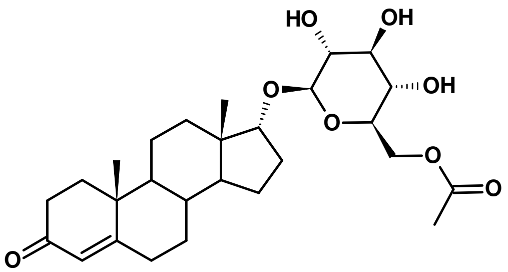

17α-*O*-β-D-(6′-*O*-acetylglucopyranosyl)-epitestosterone. ^{1}H-NMR (600 MHz) (ppm) (DMSO-d_6) δ: 0.67 (s, 3H, 18-H); 0.86 (td, 1H *J* = 11.4, 4.3 Hz, 9-Hα); 0.99 (qd, 1H, *J* = 13.2, 4.2 Hz, 7-Hα); 1.14 (s, 3H, 19-H); 1.14-1.19 (m, 1H, 11-Hβ); 1.32-1.41 (m, 3H, 12-Hα, 14-Hα and 15-Hβ); 1.47-1.55 (m, 2H, 8-Hβ and 15-Hα); 1.56-1.71 (m, 4H, 1-Hα, 11-Hα, 12-Hβ and 16-Hβ); 1.79-1.84 (m, 1H, 7-Hβ); 1.93-2.00 (m, 2H, 1-Hβ and 16-Hα); 2.00 (s, 3H, 2″-H); 2.15 (ddd, 1H, *J* = 16.7, 3.8, 3.3 Hz, 2-Hα); 2.23 (dq, 1H, *J* = 10.9, 2.5 Hz, 6-Hα); 2.35-2.43 (m, 2H, 2-Hβ and 6-Hβ); 2.90 (ddd, 8.8, 7.9, 5.0 Hz, 2′-H); 3.04 (td, 1H, *J* = 9.5, 5.5 Hz, 4′-H); 3.13 (td, 1H, *J* = 8.9, 5.0 Hz, 3′-H); 3.28 (ddd, 1H, *J* = 9.2, 6.8, 2.1 Hz, 5′-H); 3.63 (d, 1H, *J* = 5.7 Hz, 17-Hβ); 4.07 (dd, 1H, *J* = 12.3, 7.4 Hz, one of 6′-H); 4.09 (d, 1H, *J* = 7.8 Hz, 1′-H); 4.12 (dd, 1H, *J* = 11.7, 2.1 Hz, one of 6′-H); 4.89 (d, 1H, *J* = 5.0 Hz, C-2′-O*H*); 4.98 (d, 1H, *J* = 5.0 Hz, C-3′-O*H*); 5.12 (d, 1H, *J* = 5.0 Hz, C-4′-O*H*); 5.62 (s, 1H, 4-H).

^{13}C NMR (151MHz) (ppm) (DMSO-d_6) δ: 35,21 (C-1); 33,68 (C-2); 198,18 (C-3); 123,13 (C-4); 171,24 (C-5); 32,14 (C-6); 32,08 (C-7); 35,31 (C-8); 53,23 (C-9); 38,28 (C-10); 24,33 (C-11); 31,53 (C-12); 44,26 (C-13); 48,63 (C-14); 20,73 (C-15); 29,11 (C-16); 85,42 (C-17); 16,94 (C-18); 17,02 (C-19); 101,31 (C-1′); 73,34 (C-2′); 76,64 (C-3′); 70,27 (C-4′); 73,44 (C-5′); 63,70 (C-6′); 170,28 (C-1″); 20,17 (C-2″).

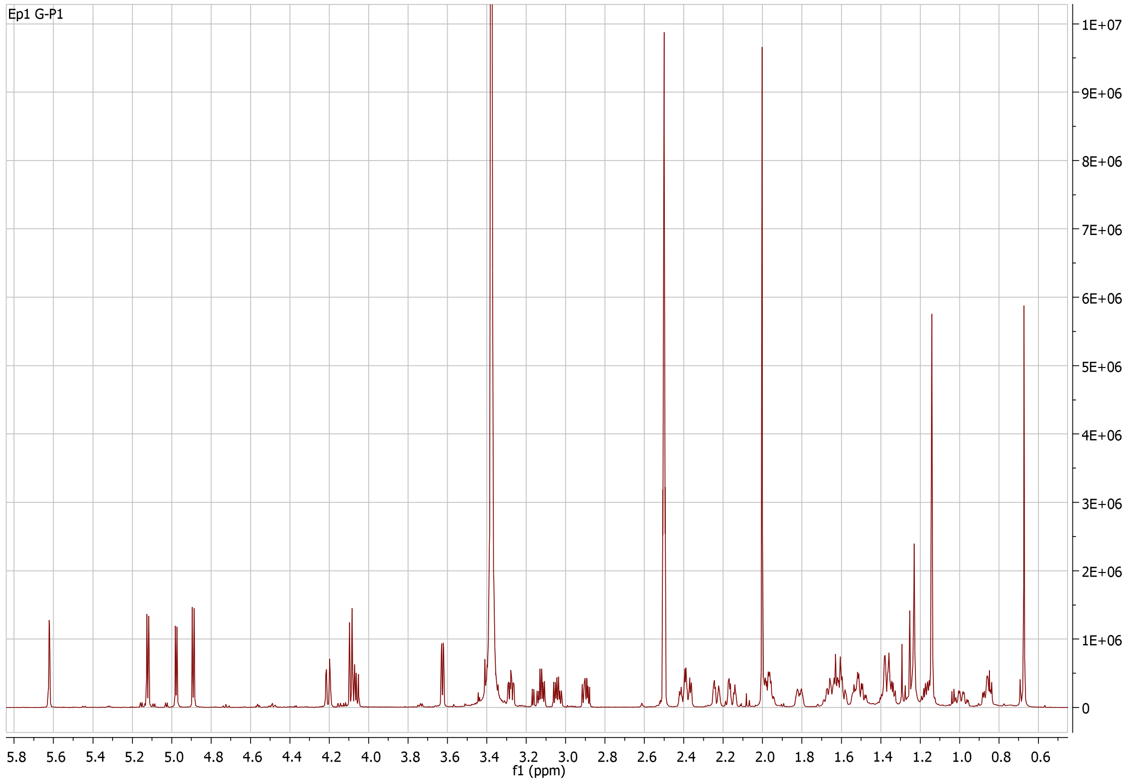

Figure S108. ^{1}H NMR spectra 17α-*O*-β-D-(6′-*O*-acetylglucopyranosyl)-epitestosterone (DMSO-d_6, 600 MHz)

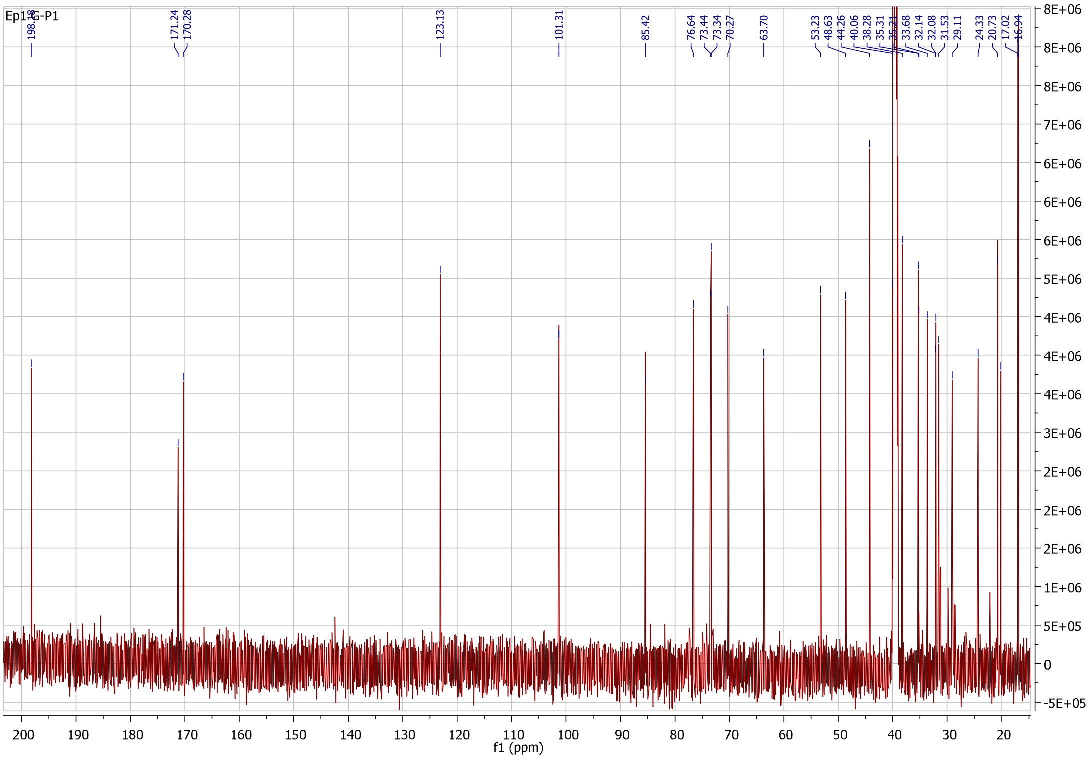

Figure S109. ^{13}C NMR spectra of 17*α*-*O*-β-D-(6′-*O*-acetylglucopyranosyl)-epitestosterone (DMSO-d_6, 151 MHz)

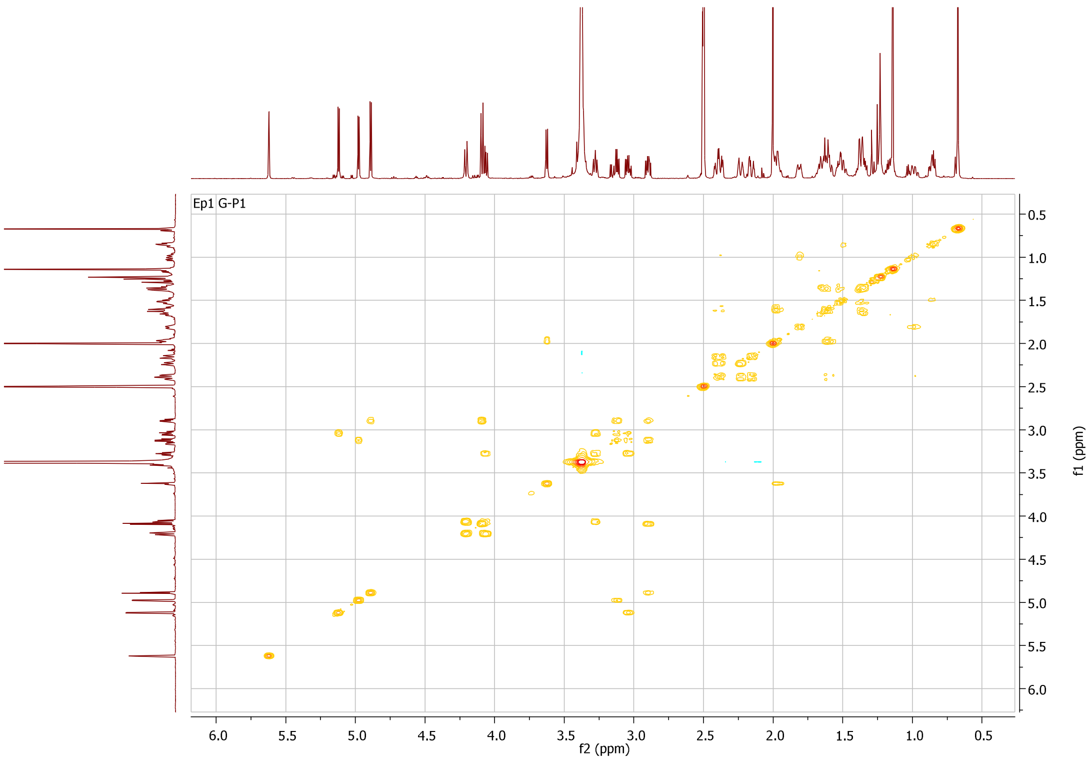

Figure S110. COSY spectrum of 17*α*-*O*-β-D-(6′-*O*-acetylglucopyranosyl)-epitestosterone (DMSO-d_6, 600 MHz)

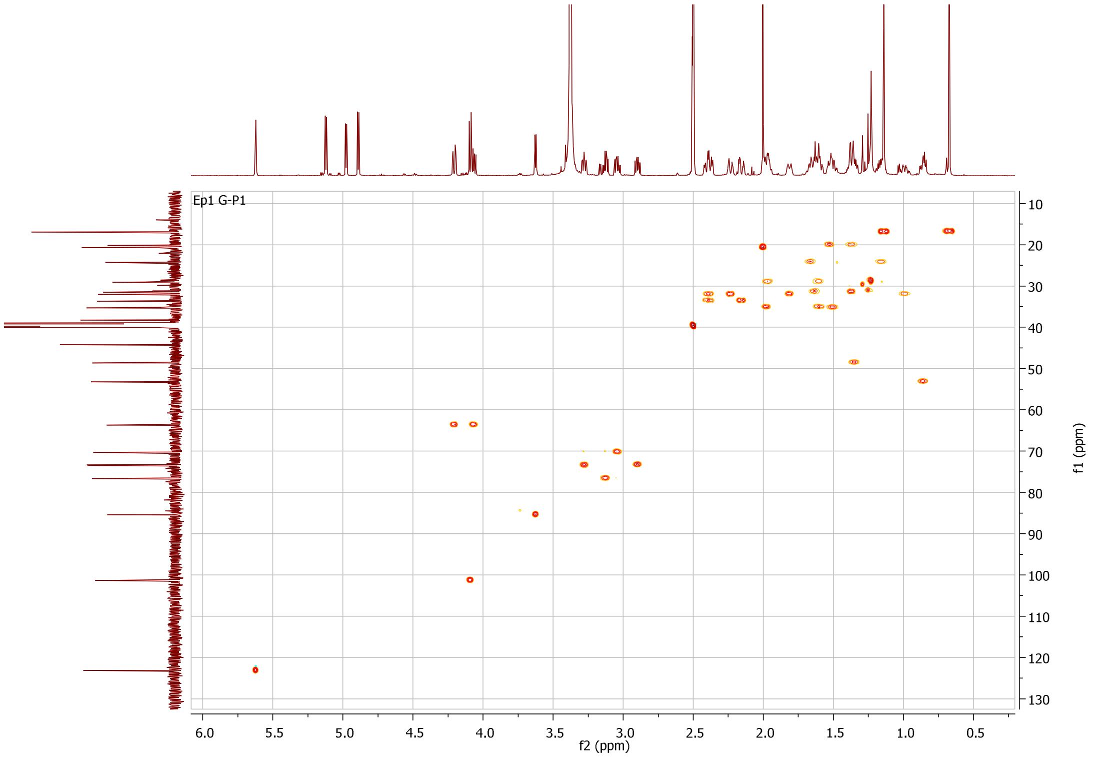

Figure S111. HSQC spectrum of 17α-*O*-β-D-(6′-*O*-acetylglucopyranosyl)-epitestosterone (DMSO-d_6, 600/151 MHz)

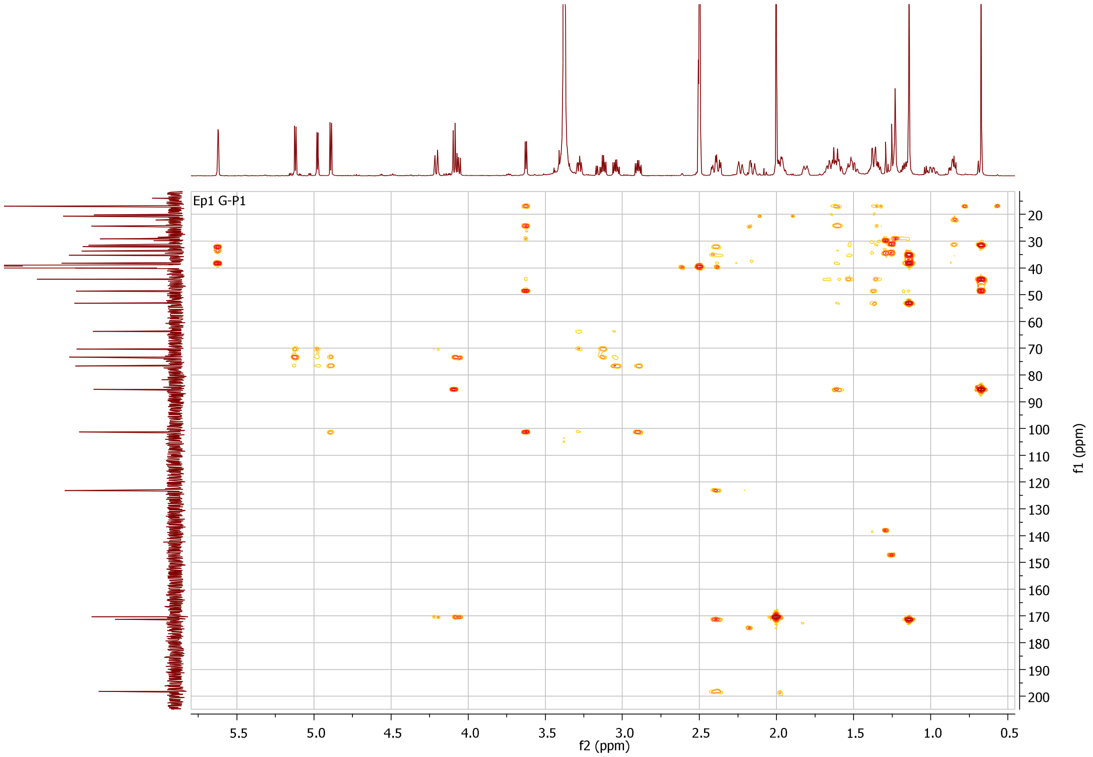

Figure S112. HMBC spectrum of 17α-*O*-β-D-(6′-*O*-acetylglucopyranosyl)-epitestosterone (DMSO-d_6, 600/151 MHz)

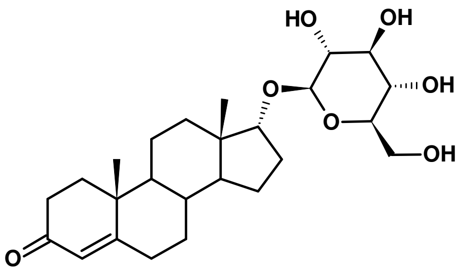

17α-*O*-β-D-glucopyranosyl-epitestosterone. ^{1}H-NMR (600 MHz) (ppm) (DMSO-*d*$_6$) δ: 0.69 (s, 3H, 18-H); 0.87 (td, 1H *J* = 12.0, 3.6 Hz, 9-Hα); 1.00 (qd, 1H, *J* = 12.3, 3.6 Hz, 7-Hα); 1.14 (s, 3H, 19-H); 1.15-1.20 (m, 1H, 11-Hβ); 1.35-1.43 (m, 3H, 12-Hα, 14-Hα and 15-Hβ); 1.45-1.56 (m, 2H, 8-Hβ and 15-Hα); 1.58-1.70 (m, 4H, 1-Hα, 11-Hα, 12-Hβ and 16-Hβ); 1.79-1.84 (m, 1H, 7-Hβ); 1.89-1.95 (m, 1H, 16-Hα); 1.96-2.02 (m, 1H, 1-Hβ); 2.14 (dt, 1H, *J* = 15.6, 3.4, 3.3 Hz, 2-Hα); 2.23 (ddd, 1H, *J* = 11.7, 3.9, 2.6 Hz, 6-Hα); 2.36-2.43 (m, 2H, 2-Hβ and 6-Hβ); 2.88 (td, 8.6, 3.3 Hz, 2'-H); 3.01 (dbrs, 1H, *J* = 8.8 Hz, 4'-H); 3.04 (dd, 1H, *J* = 9.6, 1.8 Hz, 5'-H); 3.11 (t, 1H, *J* = 8.5 Hz, 3'-H); 3.39-3.44 (m, 1H, one of 6'-H); 3.65 (dd, 1H, *J* = 11.4, 3.6 Hz, one of 6'-H); 3.73 (d, 1H, *J* = 5.6 Hz, 17-Hβ); 4.06 (d, 1H, *J* = 7.8 Hz, 1'-H); 4.40 (t, 1H, *J* = 5.4 Hz, C-6'-O*H*); 4.77 (d, 1H, *J* = 4.4 Hz, C-2'-O*H*); 4.86 (brs, 2H, C-3'-O*H* and C-4'-O*H*); 5.62 (s, 1H, 4-H).

^{13}C NMR (151MHz) (ppm) (DMSO-*d*$_6$) δ: 35,24 (C-1); 33,68 (C-2); 198,18 (C-3); 123,11 (C-4); 171,28 (C-5); 32,16 (C-6); 32,10 (C-7); 35,31 (C-8); 53,22 (C-9); 38,29 (C-10); 24,39 (C-11); 31,36 (C-12); 44,26 (C-13); 48,52 (C-14); 20,18 (C-15); 28,72 (C-16); 84,14 (C-17); 16,88 (C-18); 17,03 (C-19); 100,55 (C-1'); 73,43 (C-2'); 76,85 (C-3'); 70,28 (C-4'); 76,95 (C-5'); 61,21 (C-6').

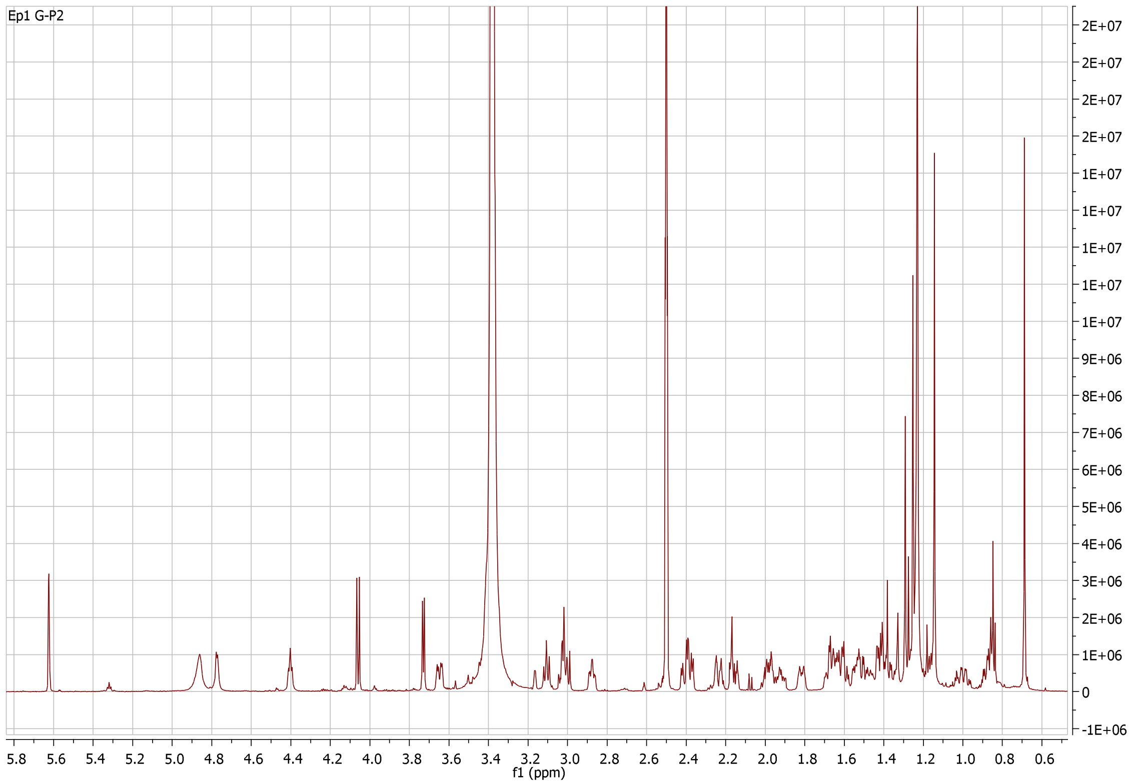

Figure S113. ^{1}H NMR spectra 17α-*O*-β-D-glucopyranosyl-epitestosterone (DMSO-*d*$_6$, 600 MHz)

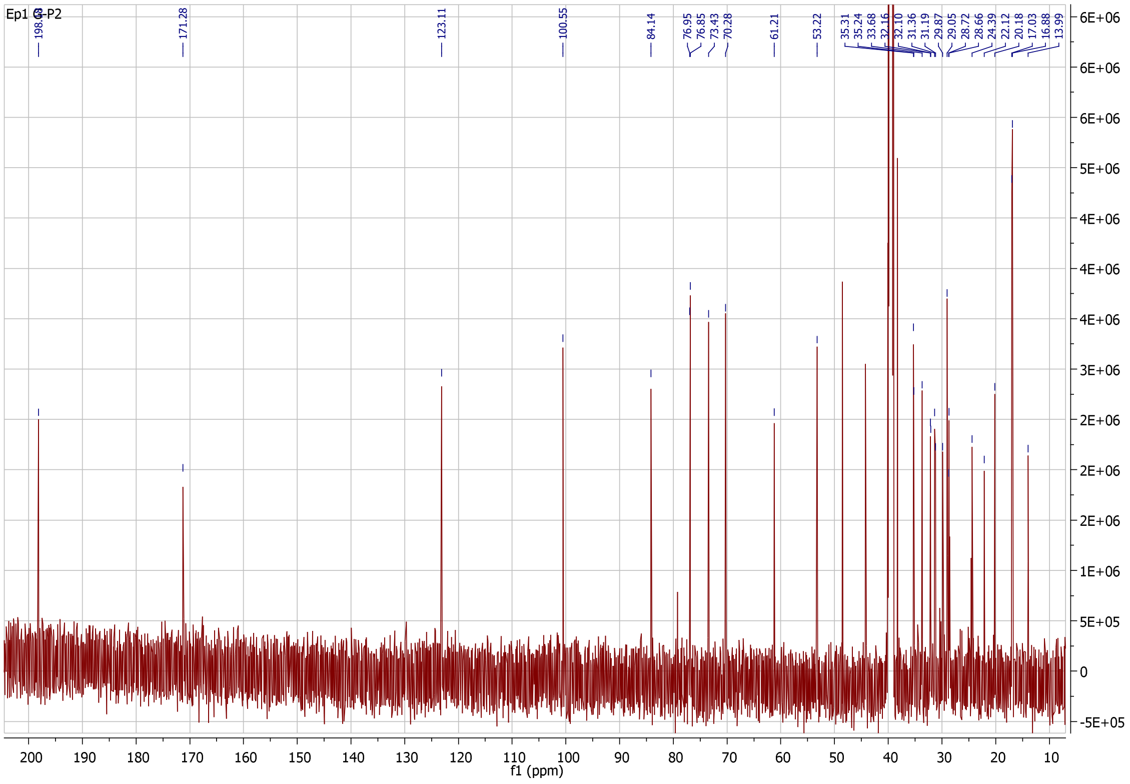

Figure S114. ^{13}C NMR spectra of 17α-*O*-β-D-glucopyranosyl-epitestosterone (DMSO-d_6, 151 MHz)

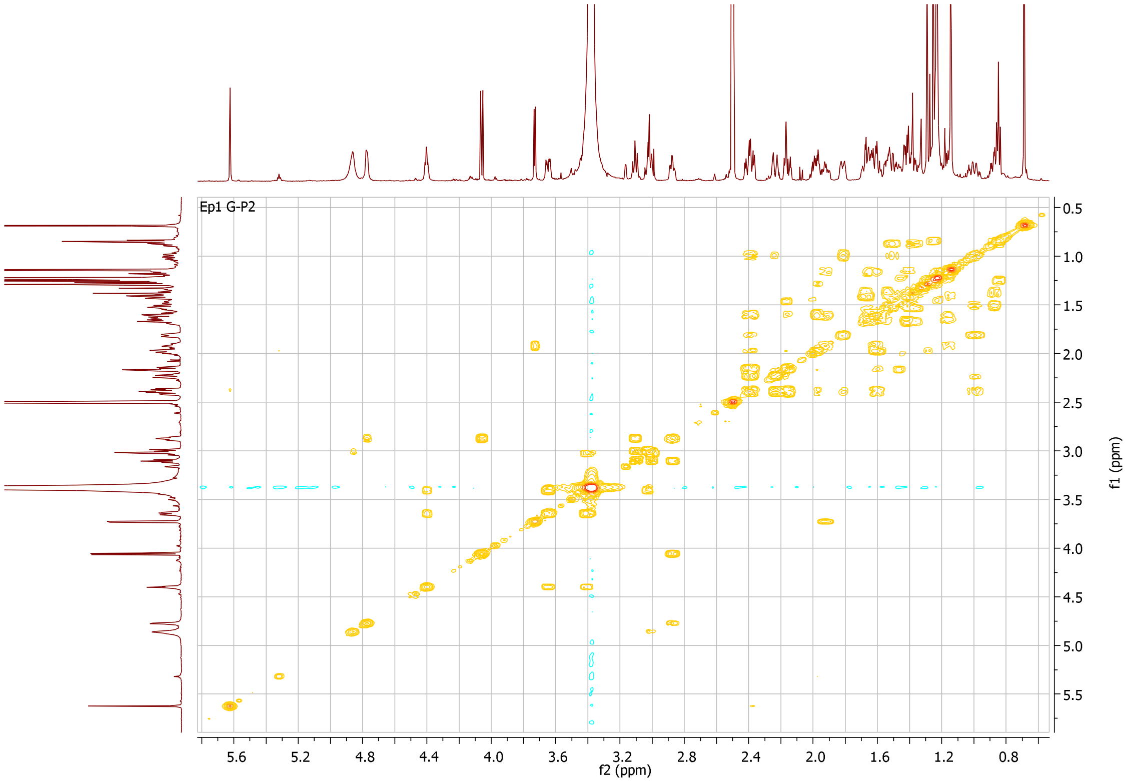

Figure S115. COSY spectrum of 17α-*O*-β-D-glucopyranosyl-epitestosterone (DMSO-d_6, 600 MHz)

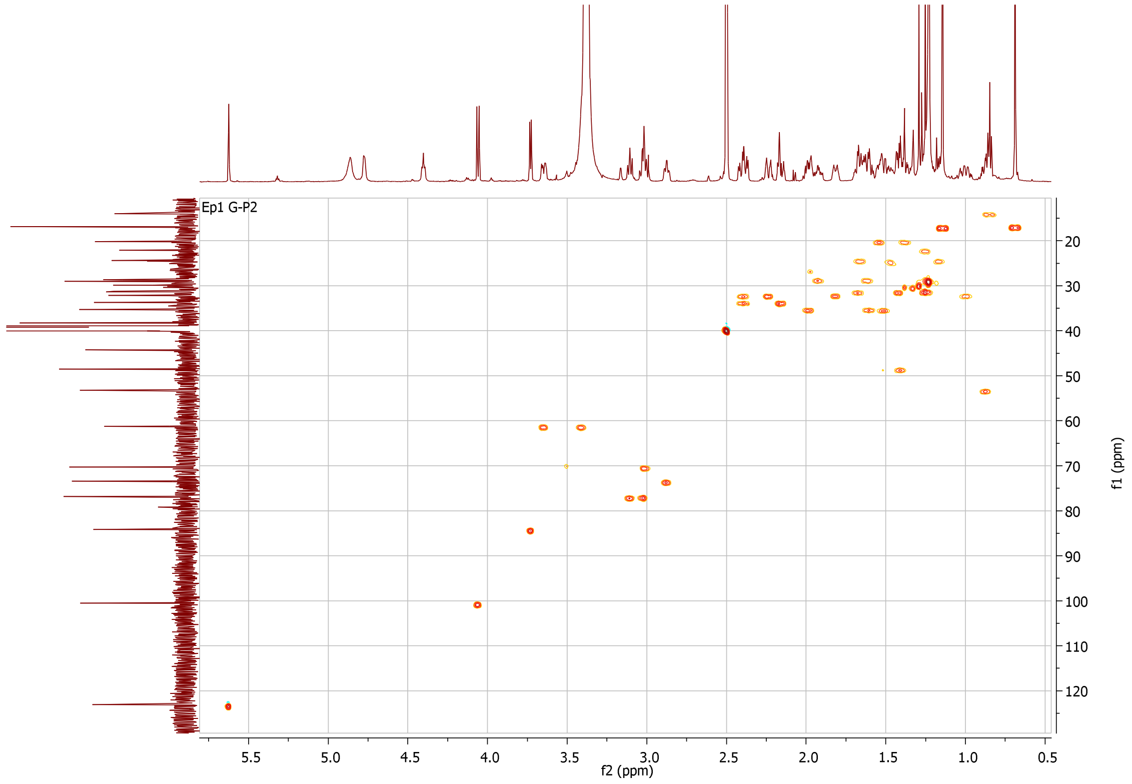

Figure S116. HSQC spectrum of 17α-*O*-β-D-glucopyranosyl-epitestosterone (DMSO-d_6, 600/151 MHz)

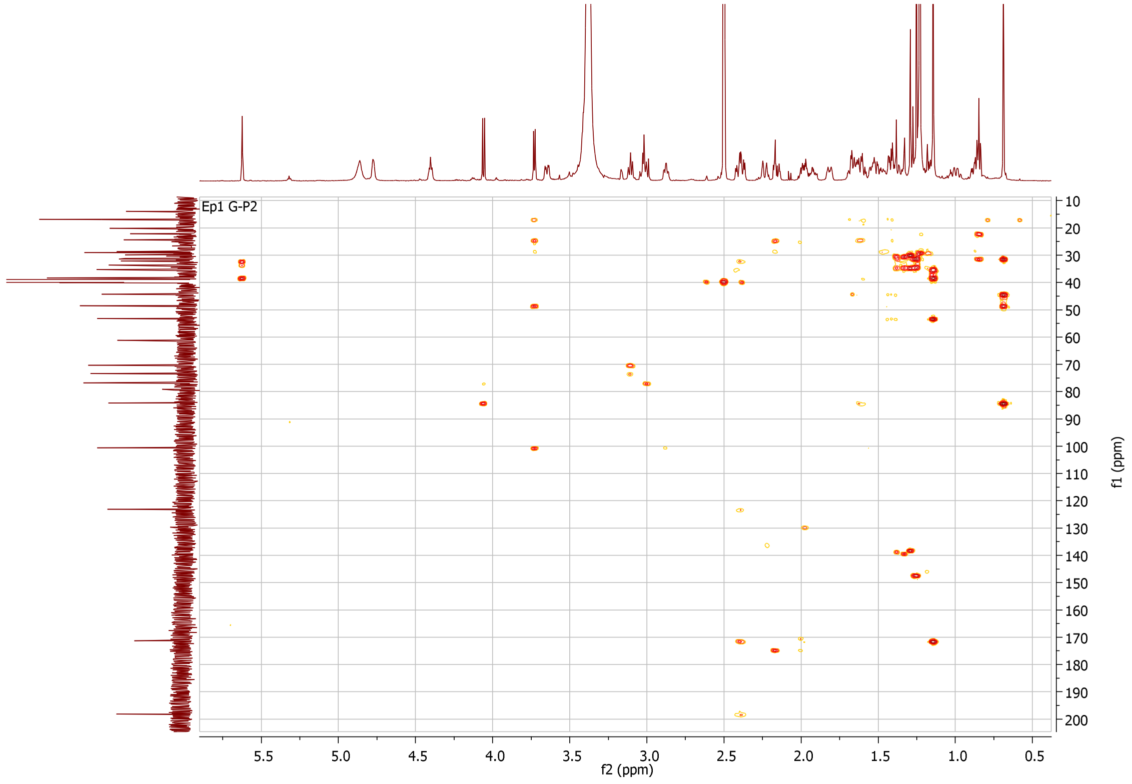

Figure S117. HMBC spectrum of 17α-*O*-β-D-glucopyranosyl-epitestosterone (DMSO-d_6, 600/151 MHz)

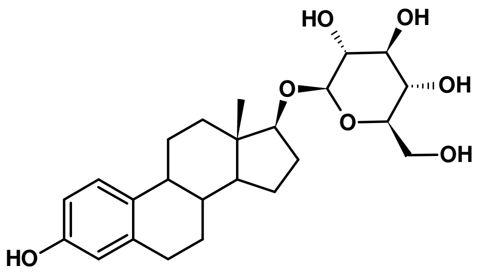

17β-*O*-β-D-glucopyranosyl-estradiol. ^{1}H NMR (600 MHz, Acetone-d_6): δ 7.97 (s, 1H, C-3-O*H*); 7.05 (d, 1H, *J* = 8.4 Hz, H-1), 7.03 (d, 1H, *J* = 8.5 Hz, H-1), 6.58 (dd, 1H, *J* = 8.4, 2.6 Hz, H-2), 6.52 (d, 1H, *J* = 2.6 Hz, H-4), 4.39 (d, 1H, *J* = 7.7 Hz, H-1′), 4.19 (d, 1H, *J* = 2.3 Hz, C-3′-O*H*); 4.16 (d, 1H, J = 3.5 Hz, C-4′-O*H*); 4.14 (d, 1H, *J* = 3.9 Hz, C-2′-O*H*); 3.84 (ddd, 1H, *J* = 11.5, 6.2, 2.5 Hz, one of H-6′), 3.78 (dd, 1H, *J* = 9.0, 8.2 Hz, H-17), 3.65 (dt, 1H, *J* = 11.5, 5.7 Hz, one of H-6′), 3.50 (d, 1H, *J* = 6.2 Hz, C-6′-O*H*); 3.38 (t, 1H, *J* = 7.9 Hz, H-3′), 3.27 (td, 1H, *J* = 9.2, 3.5 Hz, H4′), 3.25–3.28 (m, 1H, H-5′), 3.17 (td, 1H, *J* = 8.6, 3.7 Hz, H-2′), 2.70–2.79 (m, 2H, H-6α and H-6β), 2.25-2.32 (m, 1H, H-11α), 2.09–2.16 (m, 3H, H-9, H-12α, H-16α), 1.81-1.88 (m, 1H, H-7a), 1.59–1.69 (m, 2H, H-15a, H-16b), 1.29–1.47 (m, 5H, H-7b, H-8, H-11b, H-12b, H-15b), 1.20 (ddd, 1H, *J* = 12.3, 10.8, 6.8 Hz, H-14), 0.84 (s, 3H, H-18).

^{13}C NMR (151 MHz, Acetone-d_6): δc 156.01 (C-3), 138.44 (C-5), 131.99 (C-10), 127.13 (C-1), 115.99 (C-4), 113.65 (C-2), 104.59 (C-1′), 89.14 (C-17), 78.17 (C-3′), 77.42 (C-5′), 75.27 (C-2′), 71.95 (C-4′), 63.20 (C-6′), 50.72 (C-14), 44.90 (C-9), 44.20 (C-13), 39.82 (C-8), 38.32 (C-12), 30.36 (C-6), 29.88 (C-16), 28.11 (C-7), 27.24 (C-11), 23.74 (C-15), 12.02 (C-18).

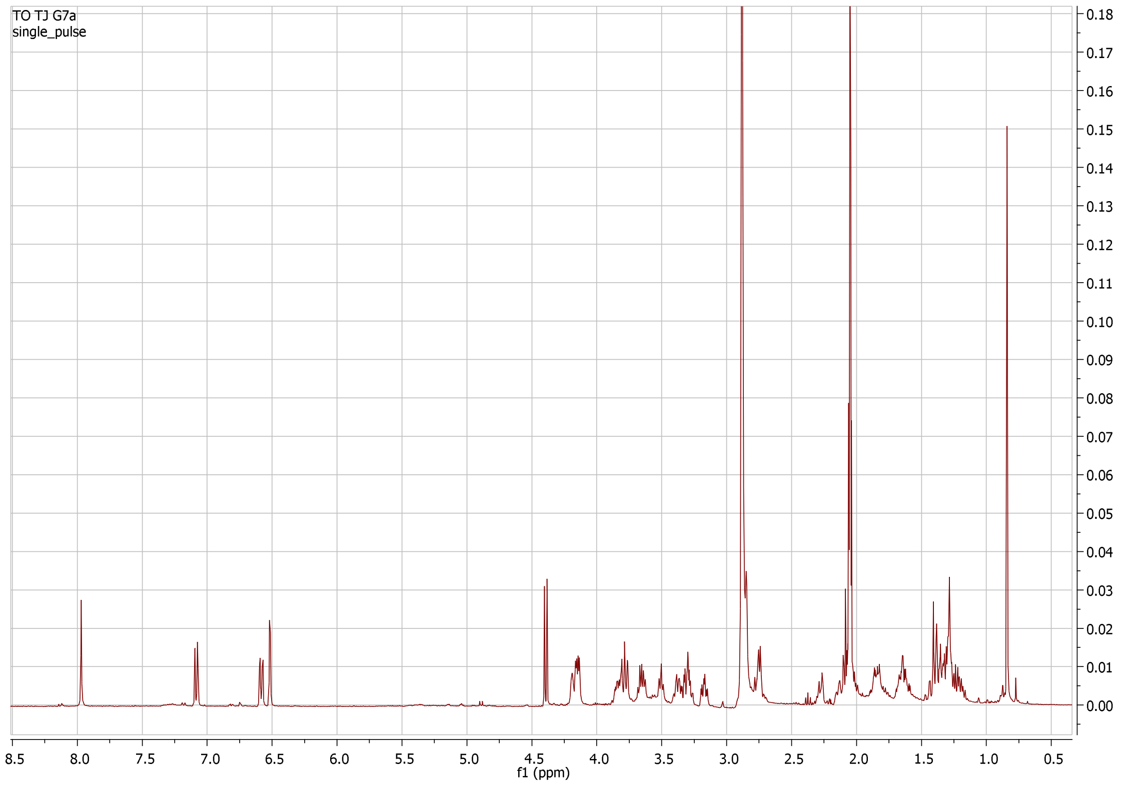

Figure S118. ^{1}H NMR spectra 17β-*O*-β-D-glucopyranosyl-estradiol (Acetone-d_6, 600 MHz)

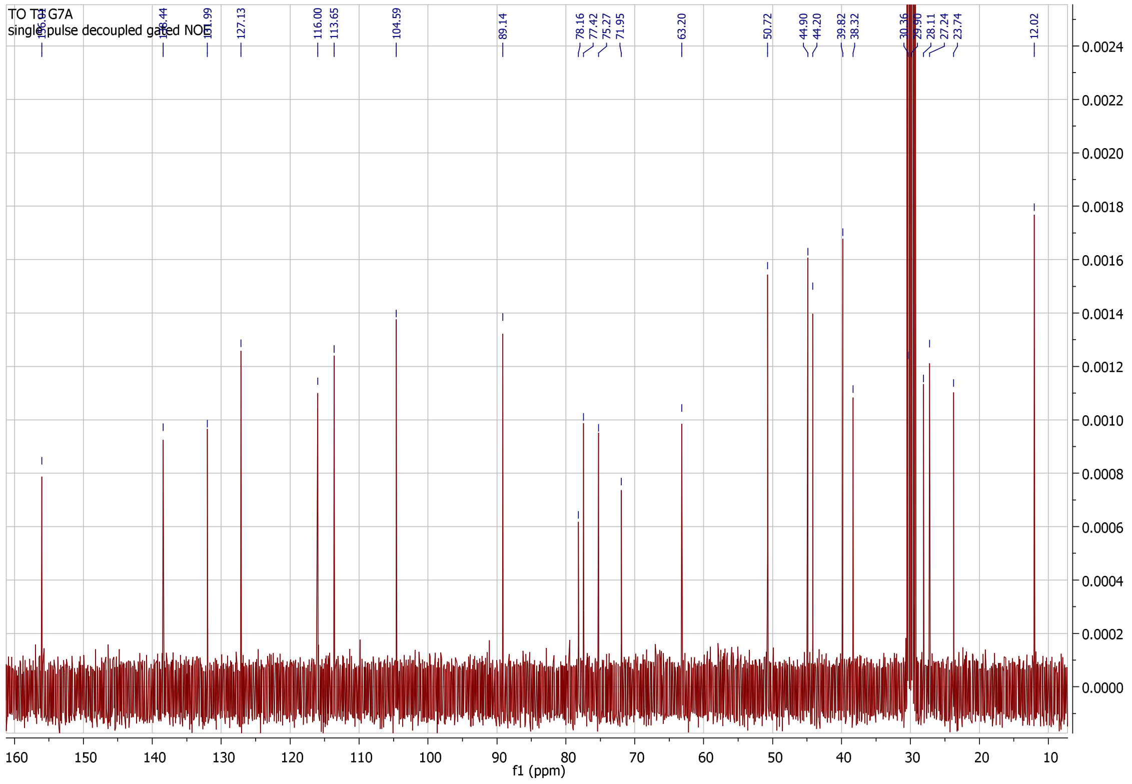

Figure S119. ^{13}C NMR spectra of 17β-*O*-β-D-glucopyranosyl-estradiol (Acetone-d_6, 151 MHz)

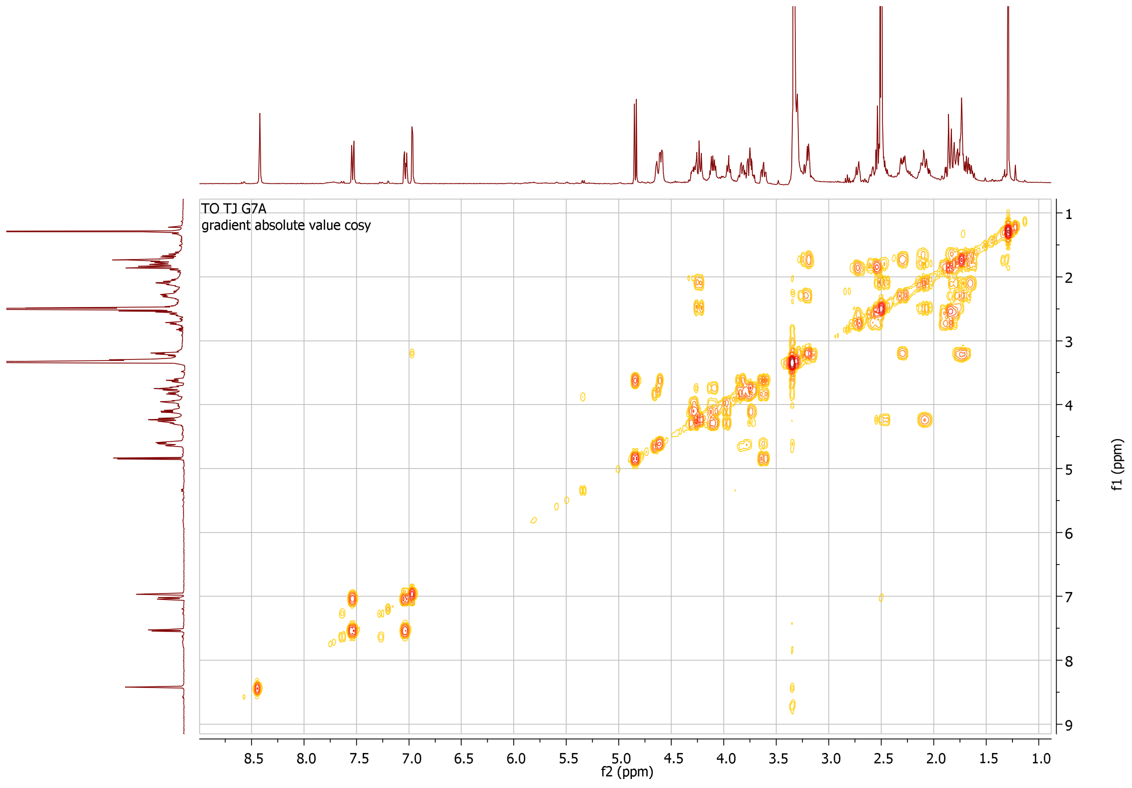

Figure S120. COSY spectrum of 17β-*O*-β-D-glucopyranosyl-estradiol (Acetone-d_6, 600 MHz)

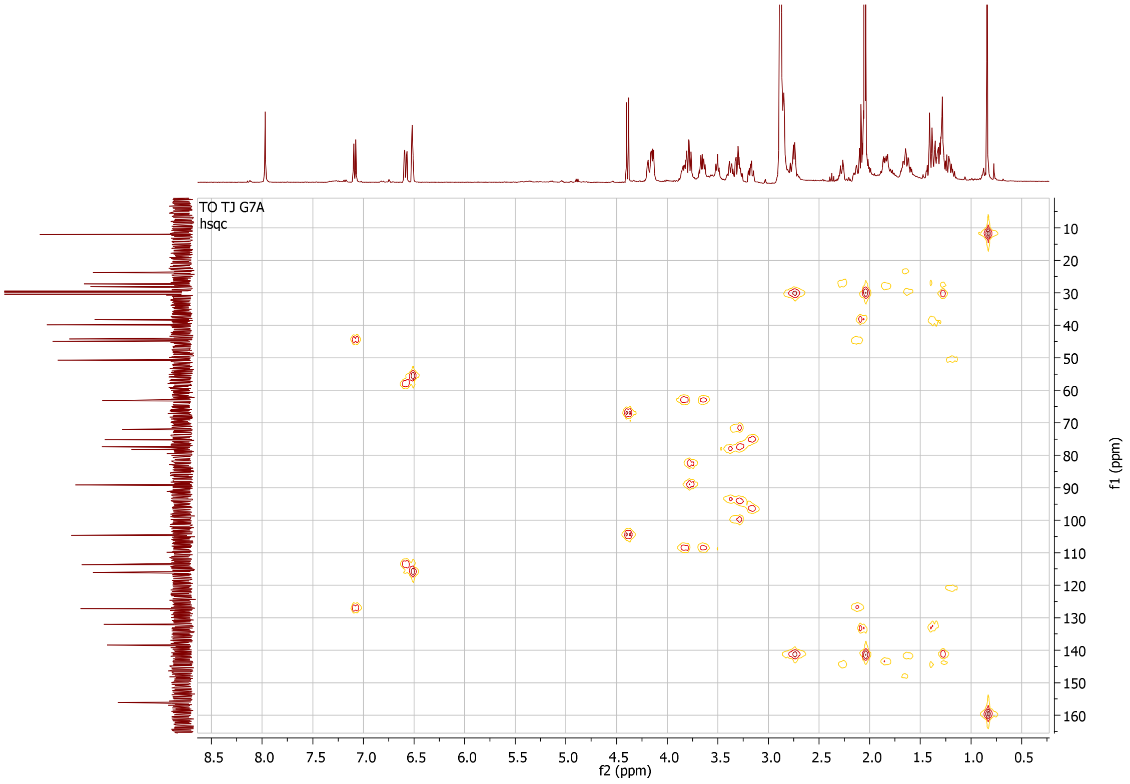

Figure S121. HSQC spectrum of 17β-*O*-β-D-glucopyranosyl-estradiol (Acetone-d_6, 600/151 MHz)

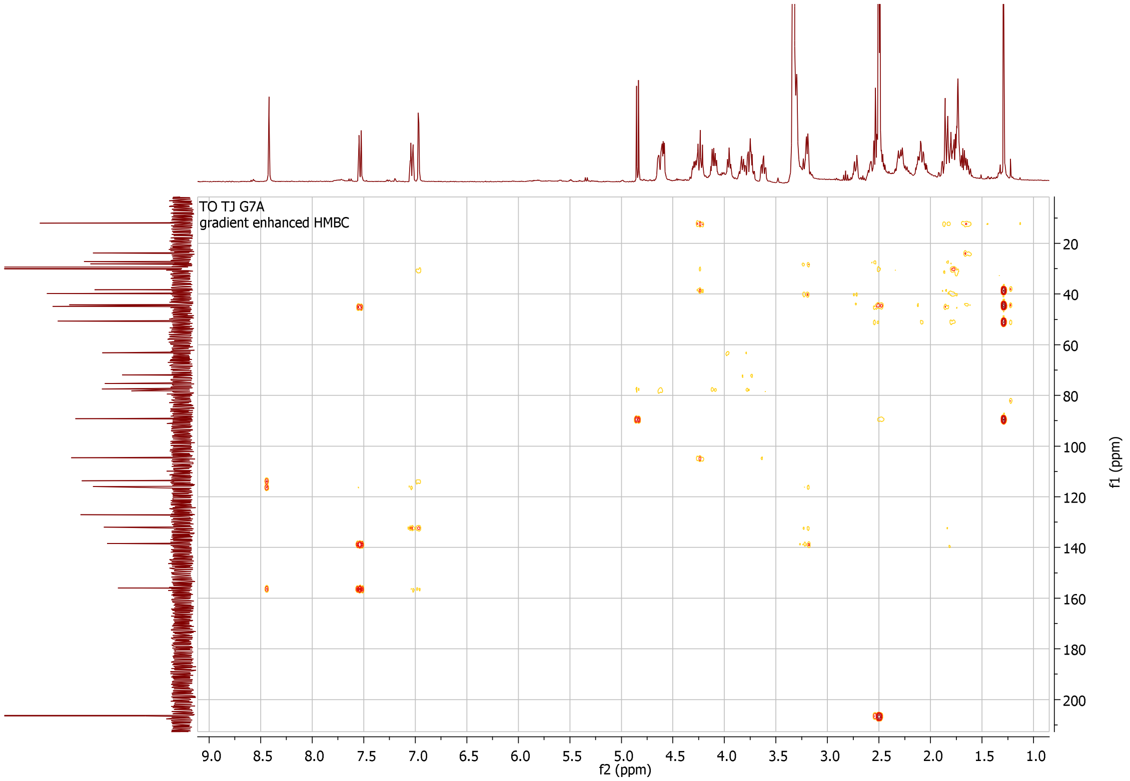

Figure S122. HMBC spectrum of 17β-*O*-β-D-glucopyranosyl-estradiol (Acetone-d_6, 600/151 MHz)

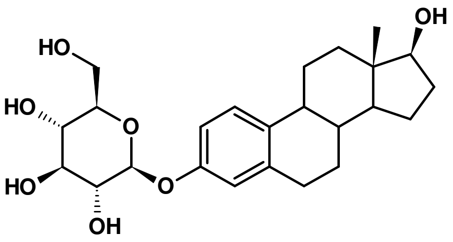

3-*O*-β-D-glucopyranosyl-estradiol. ^{1}H NMR (700 MHz, DMSO-d_6): δ 7.16 (d, 1H, J = 8.6 Hz, H-1), 6.77 (dd, 1H, J = 8.6, 2.6 Hz, H-2), 6.69 (d, 1H, J = 2.6 Hz, H-4), 5.27 (d, 1H, J = 5.0 Hz, C-3'-O*H*); 5.07 (d, 1H, J = 4.6 Hz, C-2'-O*H*); 5.01 (d, 1H, J = 5.3 Hz, C-4'-O*H*); 4.75 (d, 1H, J = 7.5 Hz, H-1'), 4.57 (t, 1H, J = 5.8 Hz, C-6'-O*H*); 4.54 (d, 1H, J = 4.9 Hz, C-17-O*H*); 3.63-3.71 (m, 1H, one of H-6'), 3.52 (td, 1H, J = 8.4, 4.7 Hz, H-17), 3.35-3.41 (m, 1H, one of H-6'), 3.08-3.29 (m, 4H, H-2', H-3', H-4' and H-5'), 2.71-7.79 (m, 2H, H-6a and H-6b), 2.26 (dq, 1H, J = 13.3, 3.2 Hz, H-11a), 2.05 – 2.14 (m, 1H, H-9), 1.68 – 1.93 (m, 3H, H-7a, H-12a and H-16a), 1.52-1.63 (m, 1H, H-15a), 1.13–1.43 (m, 7H, H-7b, H-8, H-11b, H-12b, H-15b and H-16b), 1.06 – 1.14 (m, 1H, H-14), 0.66 (s, 3H, H-18).

^{13}C NMR (151MHz, DMSO-d_6): δc 126.15 (C-1), 113.80 (C-2), 155.31 (C-3), 116.15 (C-4), 137.44 (C-5), 29.36 (C-6), 26.92 (C-7), 38.63 (C-8), 43.67 (C-9), 133.64 (C-10), 26.10 (C-11), 36.72 (C-12), 42.89 (C-13), 49.60 (C-14), 22.87 (C-15), 29.95 (C-16), 80.14 (C-17), 11.35 (C-18), 100.72 (C-1'), 73.35 (C-2'), 76.72 (C-3'), 69.86 (C-4'), 77.09 (C-5'), 60.83 (C-6').

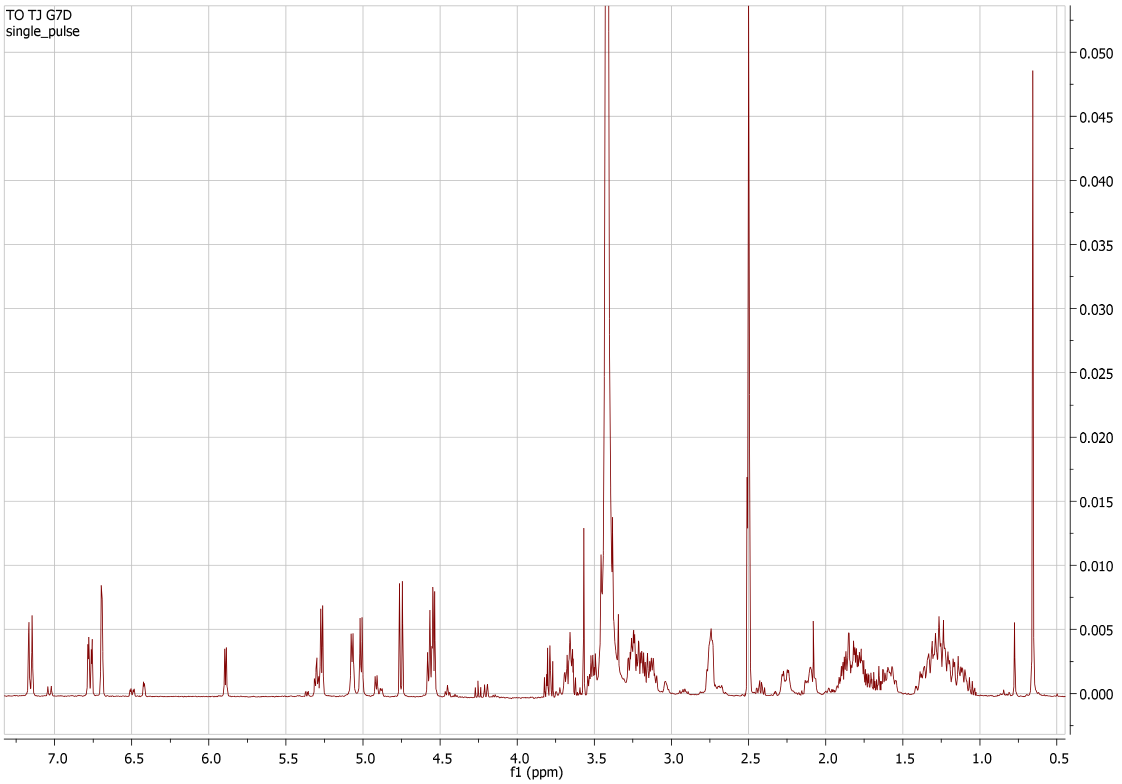

Figure S123. ^{1}H NMR spectra 3-*O*-β-D-glucopyranosyl-estradiol (DMSO-d_6, 600 MHz)

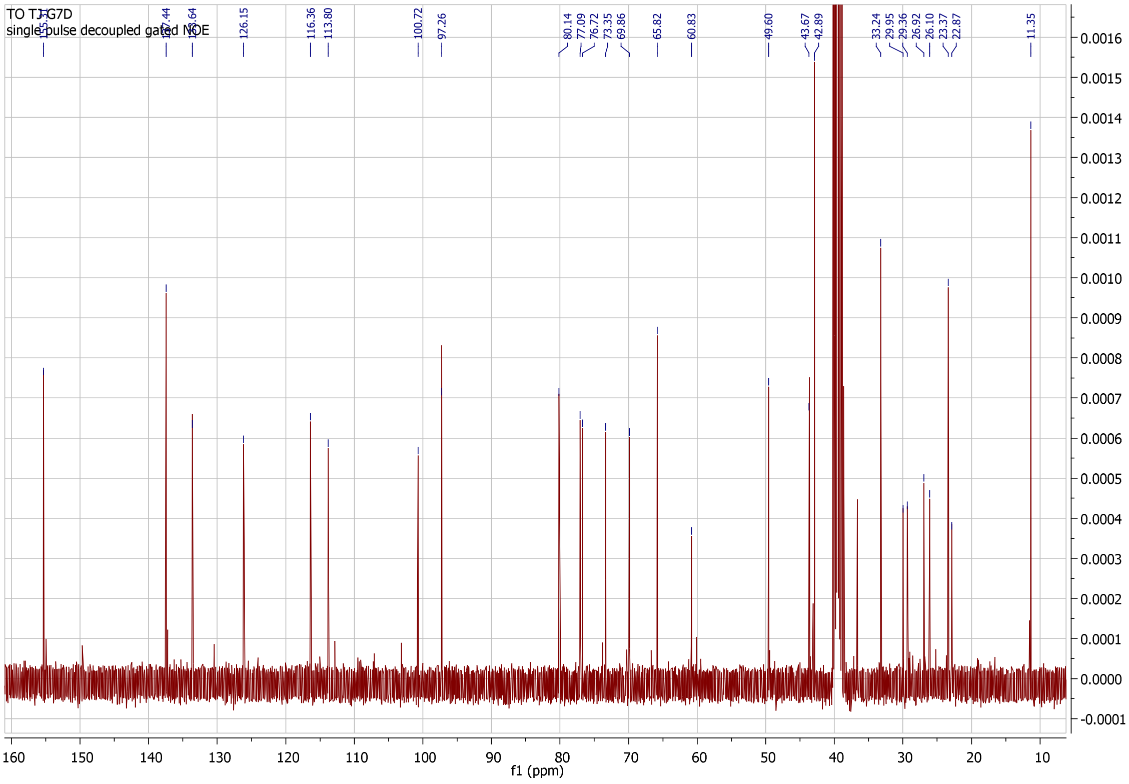

Figure S124. ^{13}C NMR spectra of 3-*O*-β-D-glucopyranosyl-estradiol (DMSO-*d*$_6$, 151 MHz)

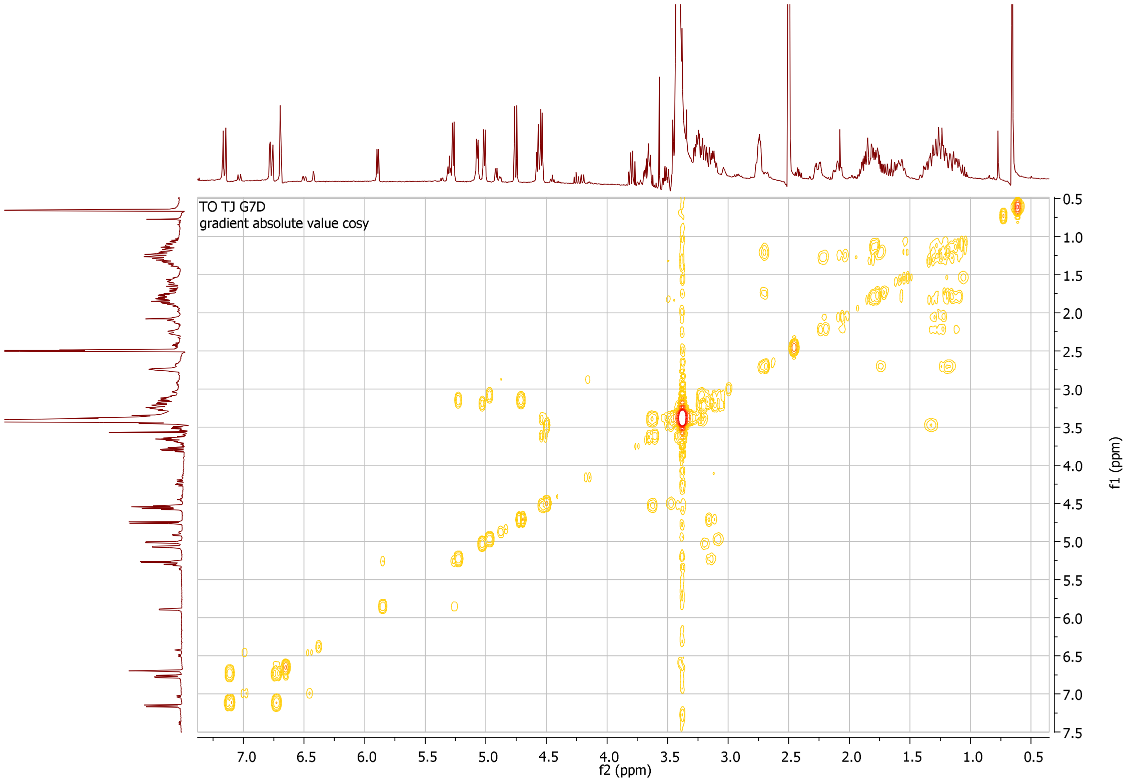

Figure S125. COSY spectrum of 3-*O*-β-D-glucopyranosyl-estradiol (DMSO-*d*$_6$, 600 MHz)

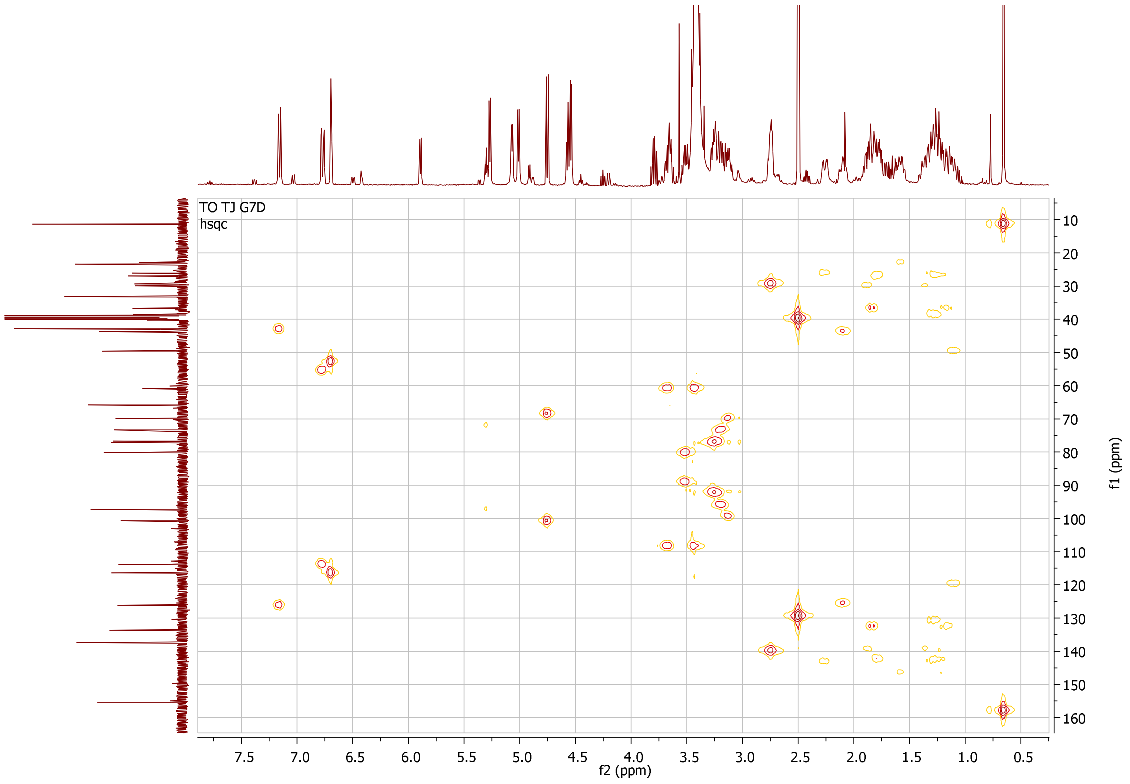

Figure S126. HSQC spectrum of 3-*O*-β-D-glucopyranosyl-estradiol (DMSO-d_6, 600/151 MHz)

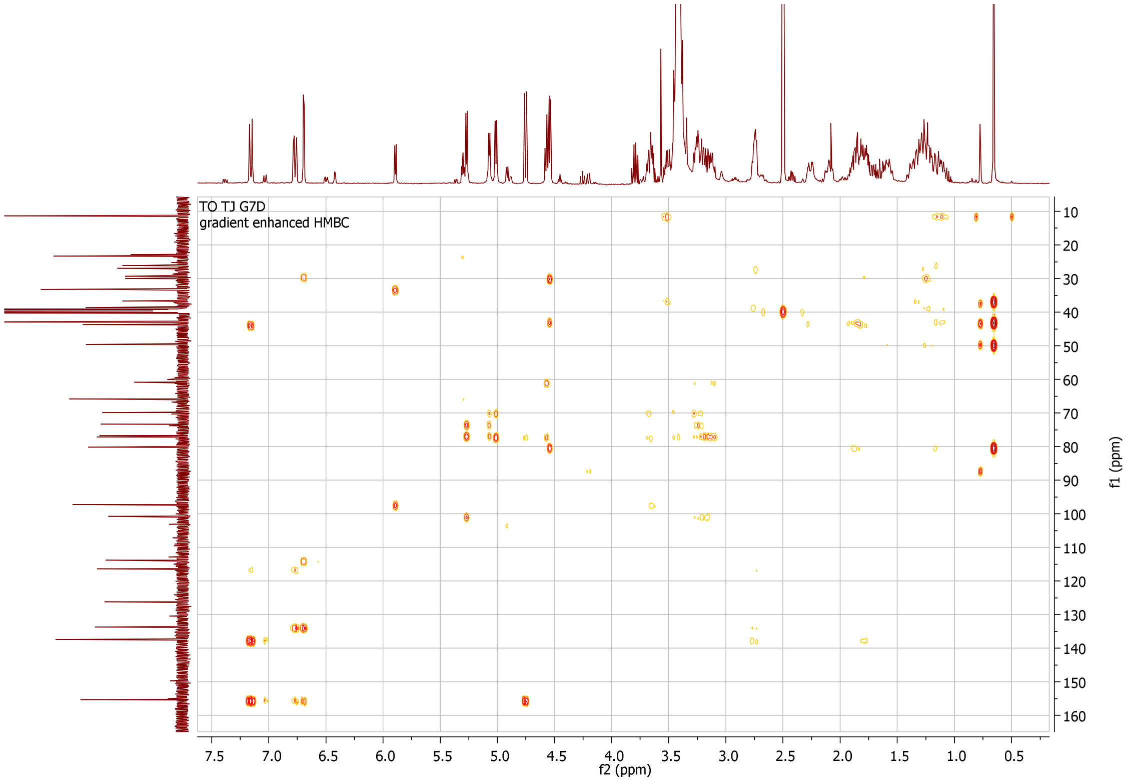

Figure S127. HMBC spectrum of 3-*O*-β-D-glucopyranosyl-estradiol (DMSO-d_6, 600/151 MHz)

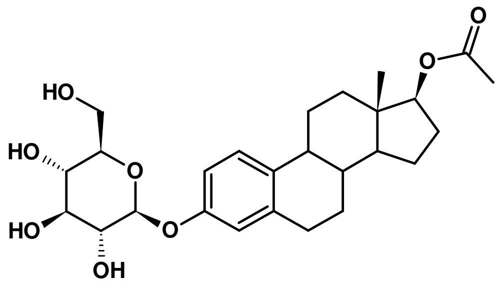

3-*O*-β-D-glucopyranosyl-estradiol 17β-acetate. ^{1}H NMR (700 MHz, Acetone-d_6): δ 7.18 (d, 1H, *J* = 8.6 Hz, H-1), 6.77 (dd, 1H, *J* = 8.6, 2.6 Hz, H-2), 6.69 (d, 1H, *J* = 2.6 Hz, H-4), 4.89 (d, 1H, *J* = 7.5 Hz, H-1′), 3.52 (dd, 1H, *J* = 9.2, 7.7 Hz, H-17), 4.51 (d, 1H, *J* = 3.9 Hz, C-3′-O*H*); 4.32 (d, 1H, *J* = 3.5 Hz, C-2′-O*H*); 4.26 (d, 1H, *J* = 3.8 Hz, C-4′-O*H*); 3.83–3.90 (m, 1H, one of H-6′), 3.64–3.73 (m, 2H, one of H-6′ and C-6′-O*H*); 3.38-3.53 (m, 4H, H-2′, H-3′, H-4′ and H-5′), 2.79–7.86 (m, 2H, H-6a and H-6b), 2.27–2.36 (m, 1H, H-11a), 2.11–2.24 (m, 2H, H-7a and H-9), 1.82–1.94 (m, 2H, H-12a and H-16a), 1.71–1.80 (m, 1H, H-15a), 1.49–1.59 (m, 1H, H-16a), 1.27–1.49 (m, 6H, H-7b, H-8, H-11b, H-12b, H-14 and H-15b), 0.85 (s, 3H, H-18).

^{13}C NMR (151MHz, Acetone-d_6): δc 126.15 (C-1), 113.96 (C-2), 155.88 (C-3), 116.56 (C-4), 137.60 (C-5), 30.44 (C-6), 27.14 (C-7), 38.70 (C-8), 43.91 (C-9), 134.61 (C-10), 26.16 (C-11), 36.92 (C-12), 42.82 (C-13), 49.68 (C-14), 22.99 (C-15), 27.41 (C-16), 82.23 (C-17), 11.58 (C-18), 170.12 ($COCH_3$), 20.58 ($COCH_3$), 101.19 (C-1′), 73.92 (C-2′), 76.92 (C-3′), 70.61 (C-4′), 77.20 (C-5′), 61.91 (C-6′).

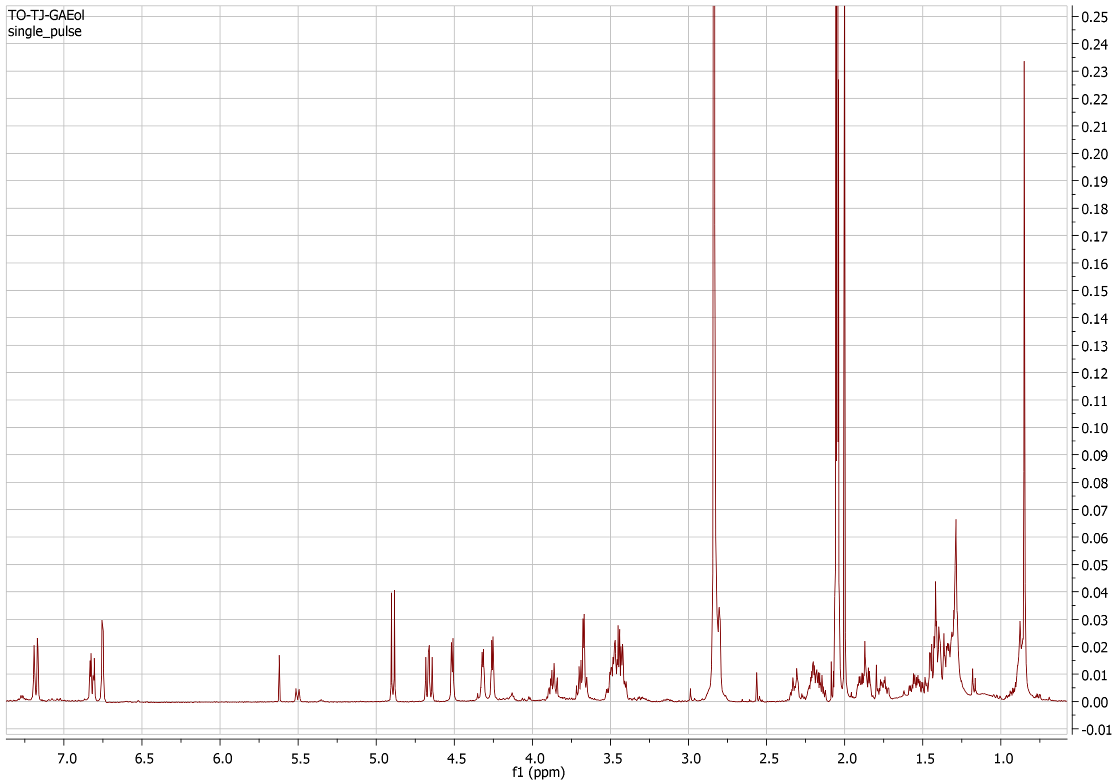

Figure S128. ^{1}H NMR spectra 3-*O*-β-D-glucopyranosyl-estradiol 17β-acetate (Acetone-d_6, 600 MHz)

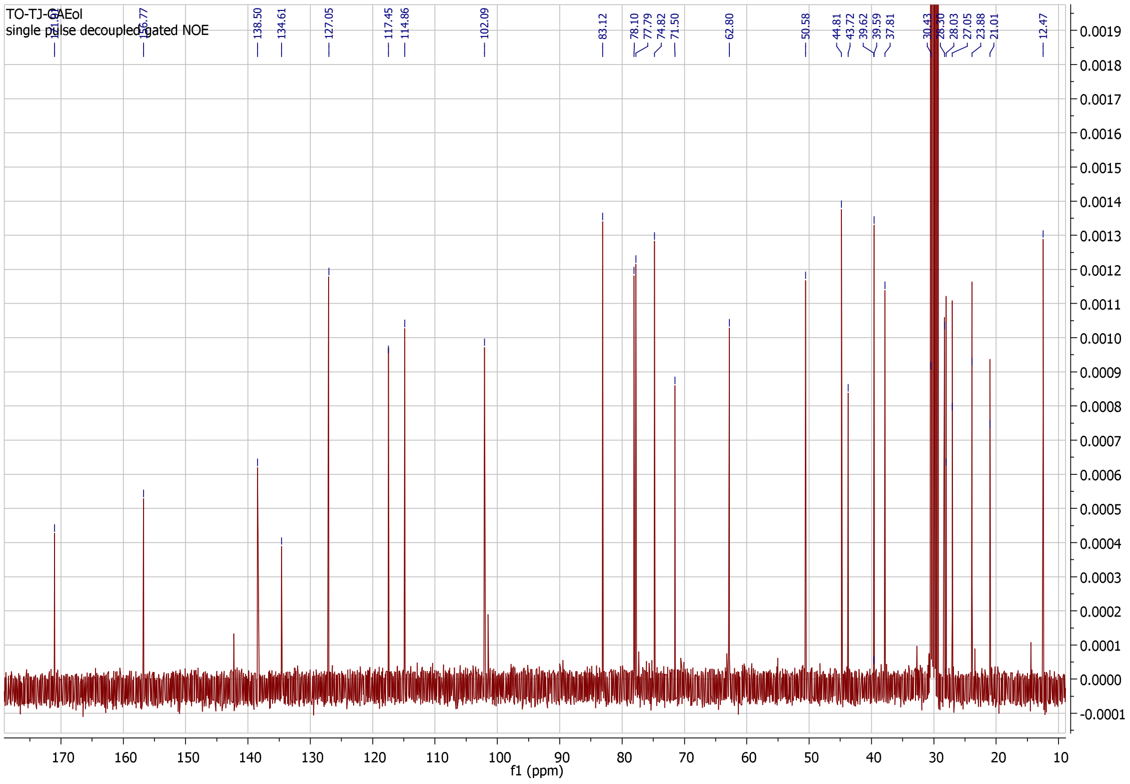

Figure S129. ^{13}C NMR spectra of 3-*O*-β-D-glucopyranosyl-estradiol 17β-acetate (Acetone-*d*6, 151 MHz)

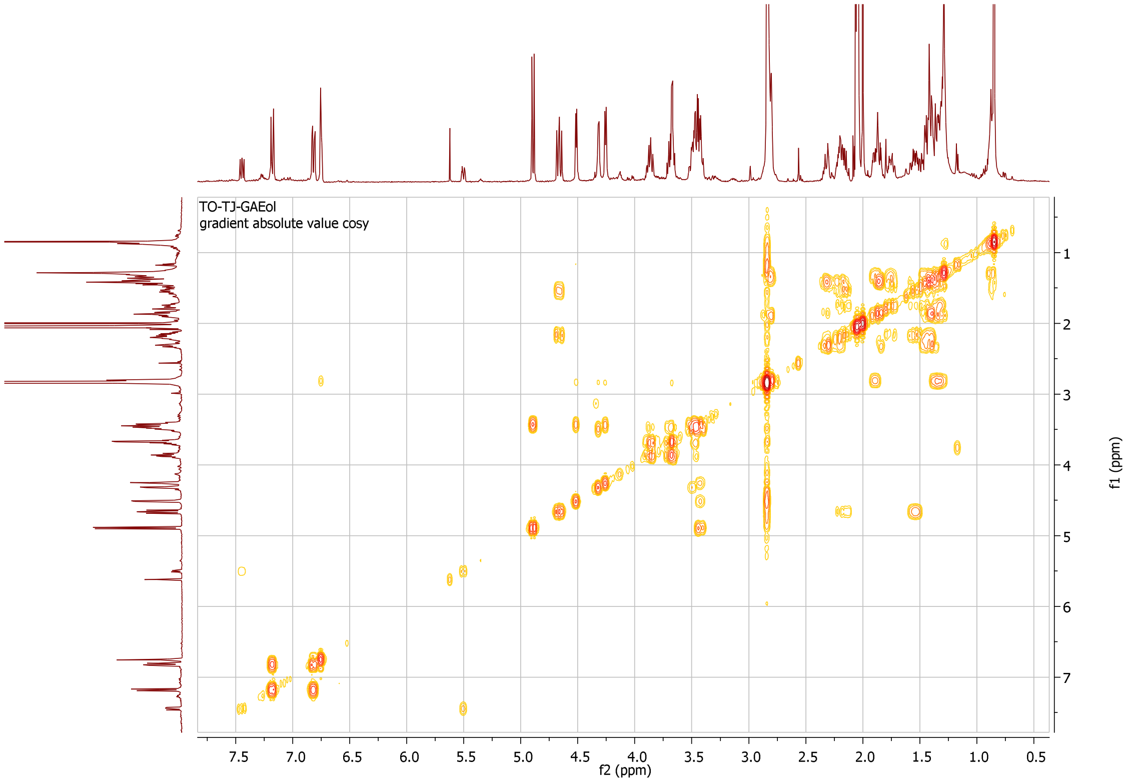

Figure S130. COSY spectrum of 3-*O*-β-D-glucopyranosyl-estradiol 17β-acetate (Acetone-*d*6, 600 MHz)

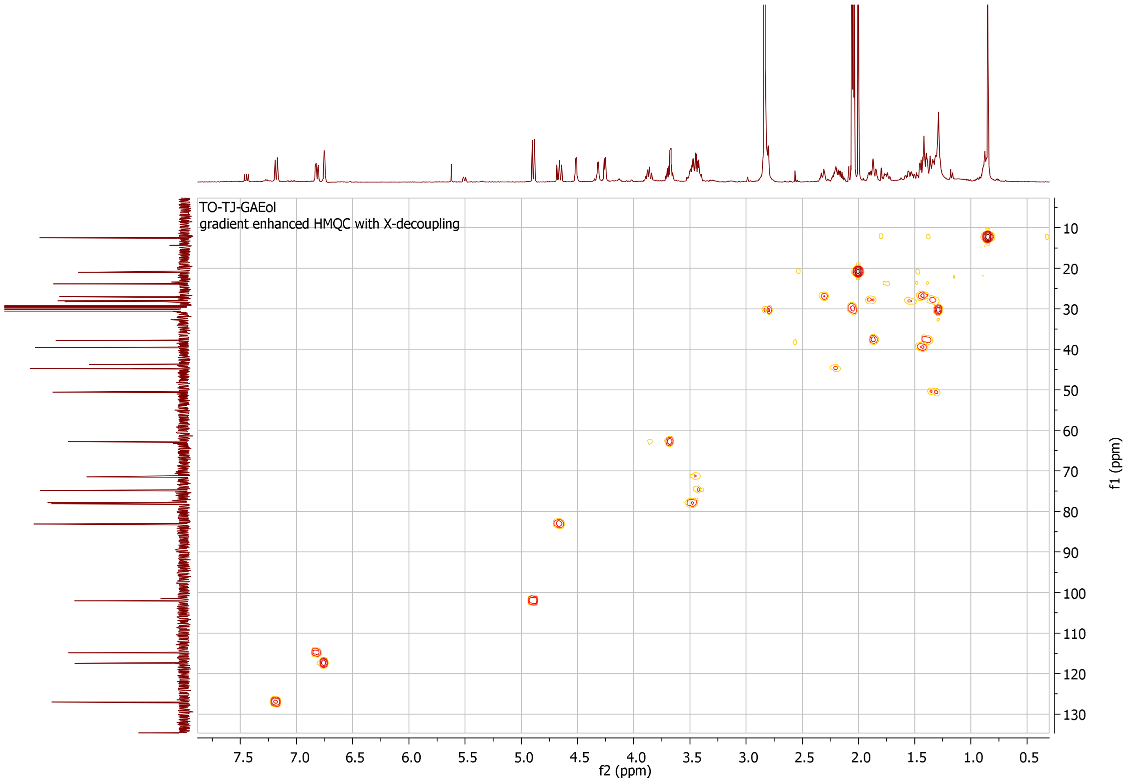

Figure S131. HSQC spectrum of 3-*O*-β-D-glucopyranosyl-estradiol 17β-acetate (Acetone-d_6, 600/151 MHz)

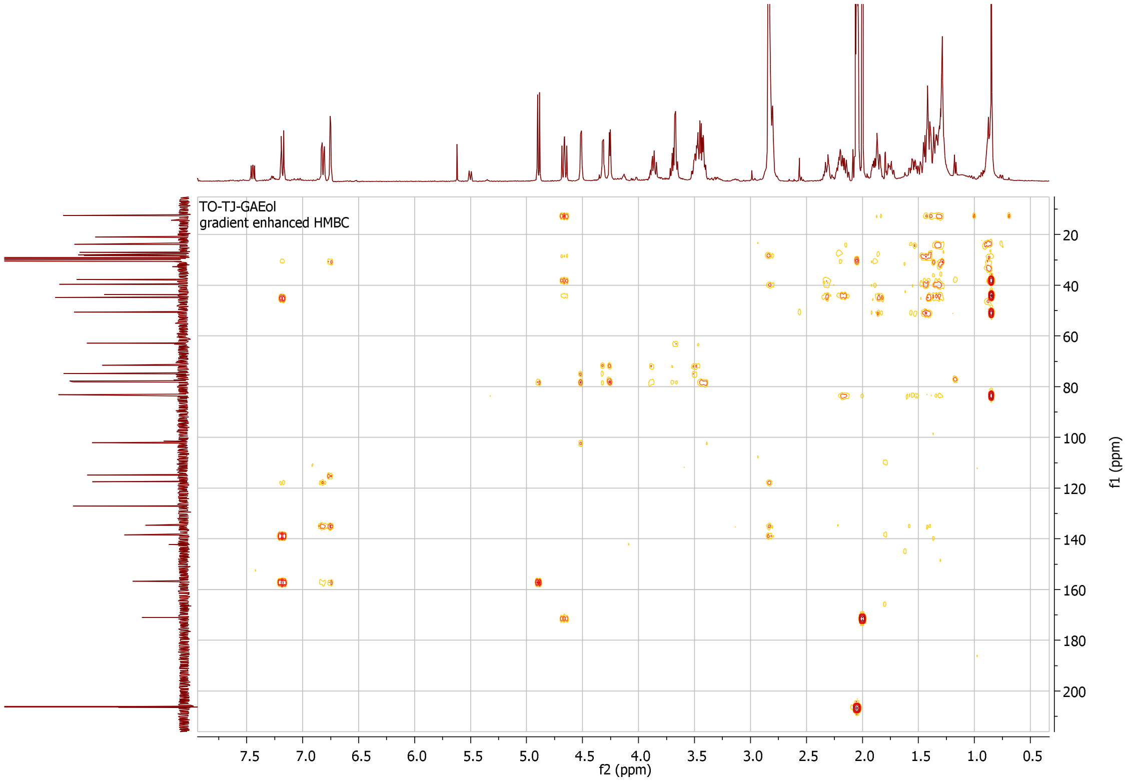

Figure S132. HMBC spectrum of 3-*O*-β-D-glucopyranosyl-estradiol 17β-acetate (Acetone-d_6, 600/151 MHz)

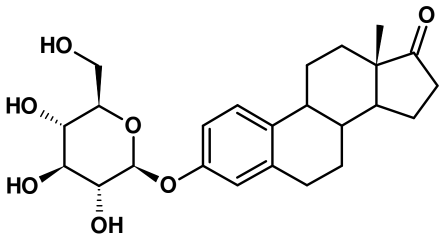

3-*O*-β-D-glucopyranosyl-estrone. ^{1}H NMR (600 MHz, Acetone-d_6): δ 7.20 (d, 1H, *J* = 8.6 Hz, H-1), 6.83 (dd, 1H, *J* = 8.6, 2.7 Hz, H-2), 6.77 (d, 1H, *J* = 2.6 Hz, H-4), 4.90 (d, 1H, *J* = 7.6 Hz, H-1'), 4.54 (d, 1H, J = 3.9 Hz, C-2'-O*H*); 4.33 (d, 1H, *J* = 3.6 Hz, C-3'-O*H*); 4.26 (d, 1H, *J* = 3.9 Hz, C-4'-O*H*); 3.84-3.91 (m, 1H, one of H-6'), 3.64-3.73 (m, 2H, one of H-6' and C-6'-O*H*); 3.39-3.54 (m, 4H, H-2', H-3', H-4' and H-5'), 2.79-7.90 (m, 2H, H-6a and H-6b), 2.44 (dd, 1H, *J* = 17.7, 8.8 Hz, H-16a), 2.37-2.42 (m, 1H, H-11a), 2.20–2.29 (m, 1H, H-9), 2.07–2.12 (m, 1H, H-16), 1.82-1.97 (m, 1H, H-12a), 1.64-1.70 (m, 1H, H-15a), 1.48-1.56 (m, 2H, H-8 and H-14), 1.34–1.47 (m, 3H, H-7b, H-11b, and H-12b), 0.89 (s, 3H, H-18).

^{13}C NMR (151MHz, Acetone-d_6): δc 127.75 (C-1), 115.63 (C-2), 157.53 (C-3), 118.20 (C-4), 139.20 (C-5), 31.06 (C-6), 28.02 (C-7), 40.01 (C-8), 45.71 (C-9), 135.10 (C-10), 27.43 (C-11), 33.37 (C-12), 49.17 (C-13), 51.87 (C-14), 22.90 (C-15), 36.83 (C-16), 220.25 (C-17), 14.87 (C-18), 100.79 (C-1'), 75.51 (C-2'), 78.48 (C-3'), 72.20 (C-4'), 78.79 (C-5'), 63.50 (C-6').

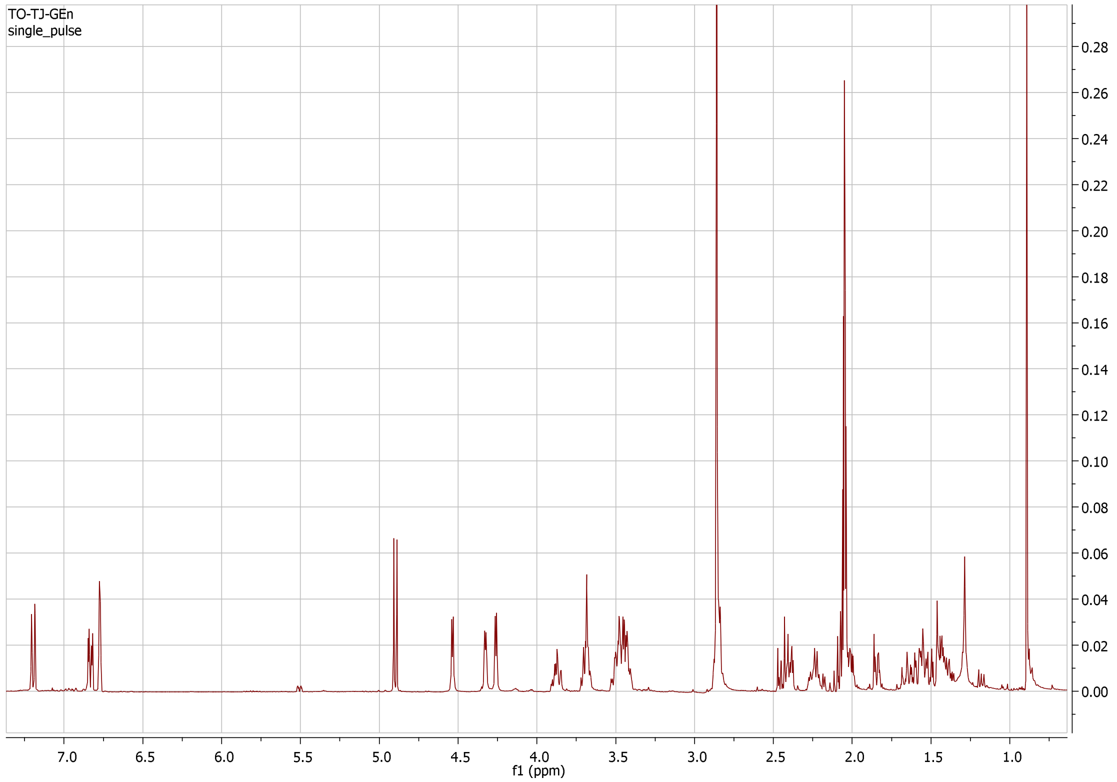

Figure S133. ^{1}H NMR spectra 3-*O*-β-D-glucopyranosyl-estrone (Acetone-d_6, 600 MHz)

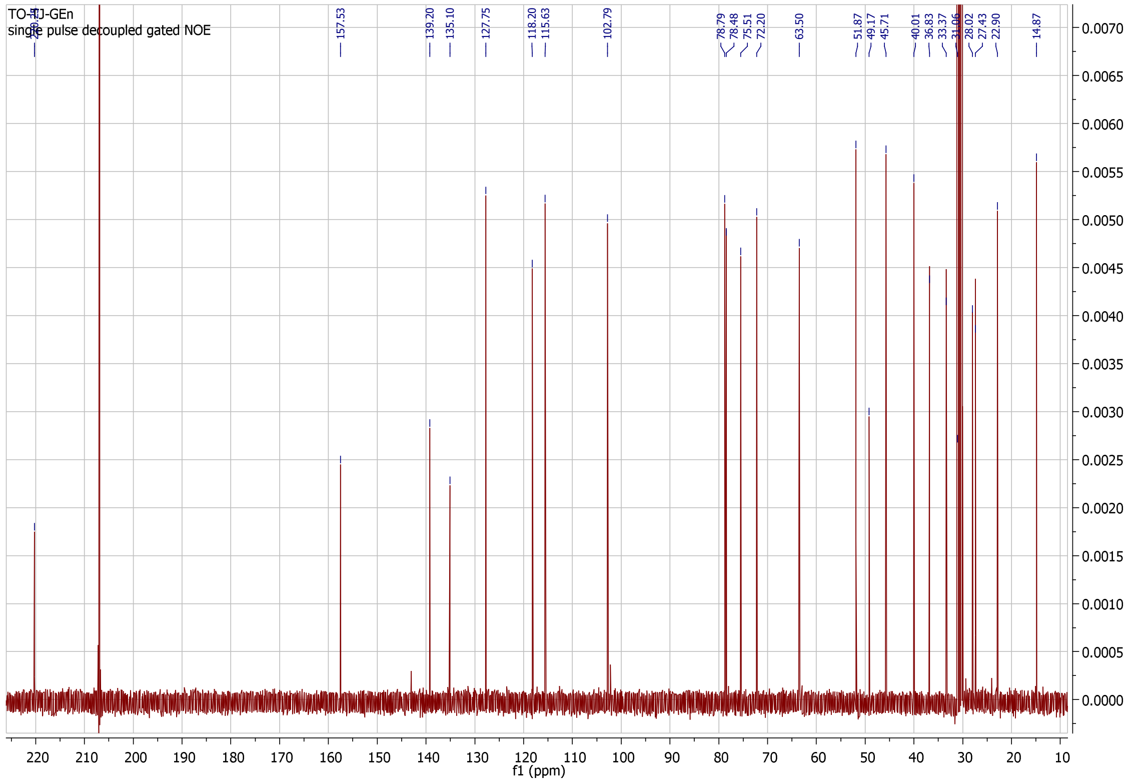

Figure S134. ^{13}C NMR spectra of 3-*O*-β-D-glucopyranosyl-estrone (Acetone-d_6, 151 MHz)

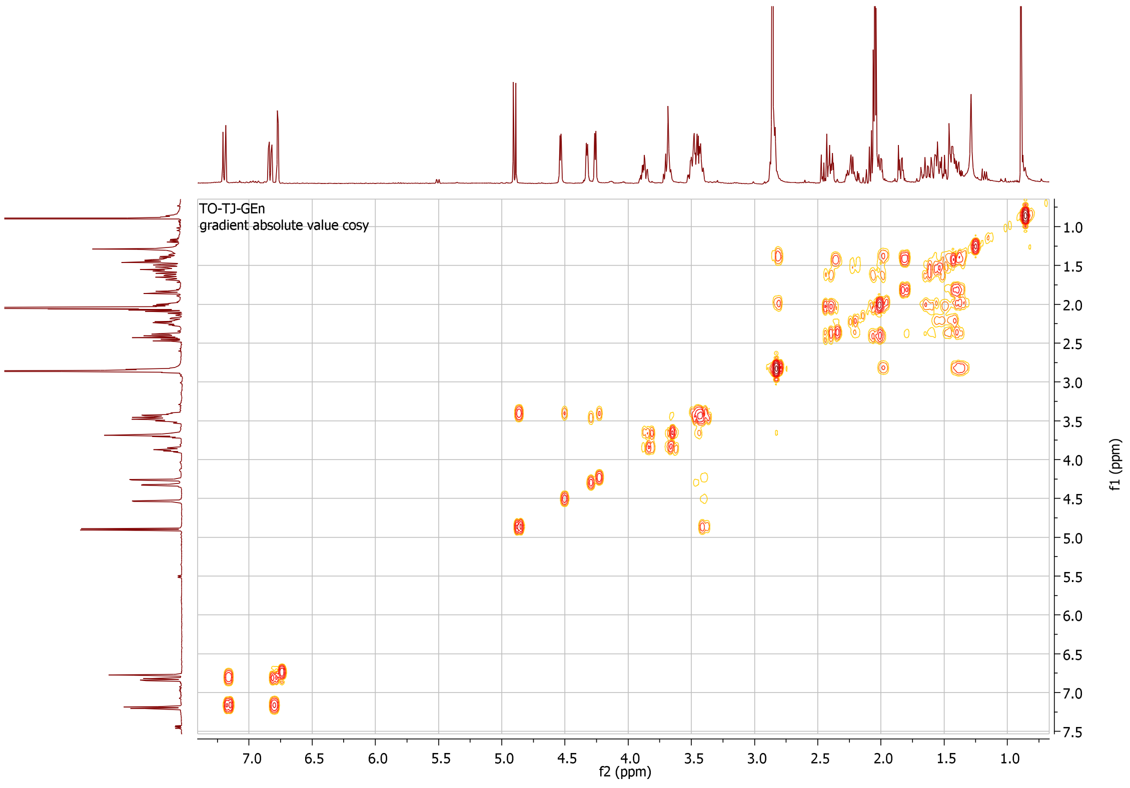

Figure S135. COSY spectrum of 3-*O*-β-D-glucopyranosyl-estrone (Acetone-d_6, 600 MHz)

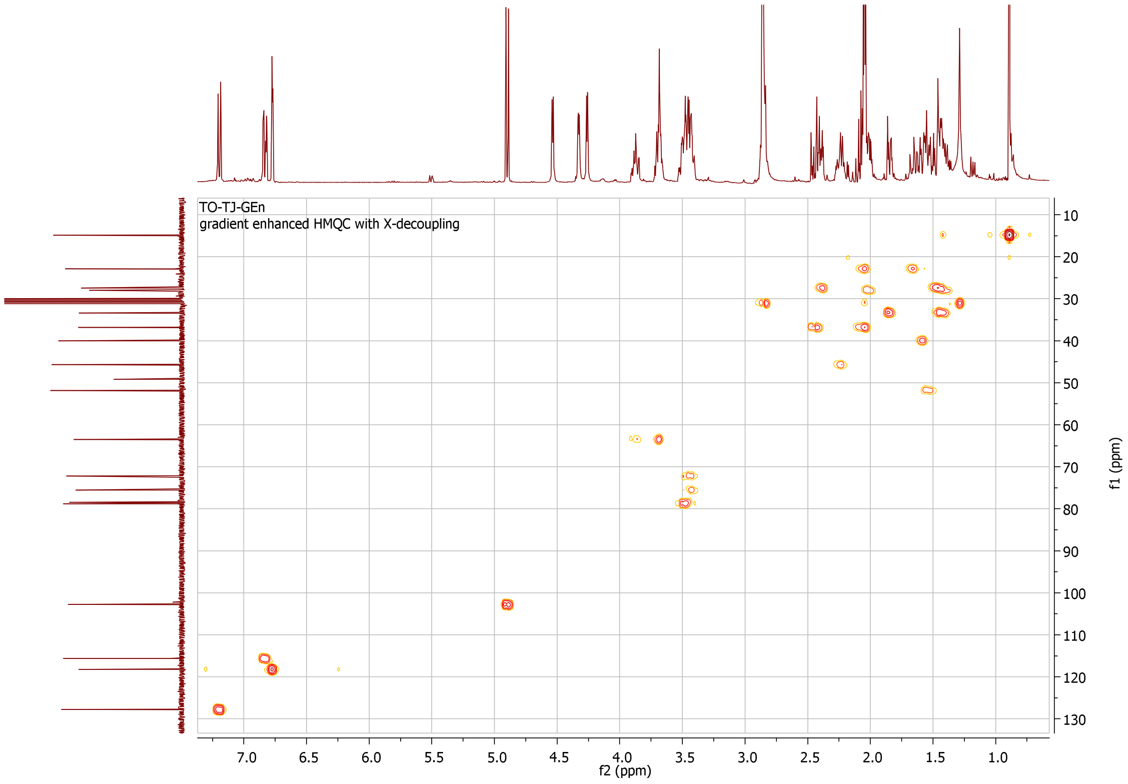

Figure S136. HSQC spectrum of 3-*O*-β-D-glucopyranosyl-estrone (Acetone-d_6, 600/151 MHz)

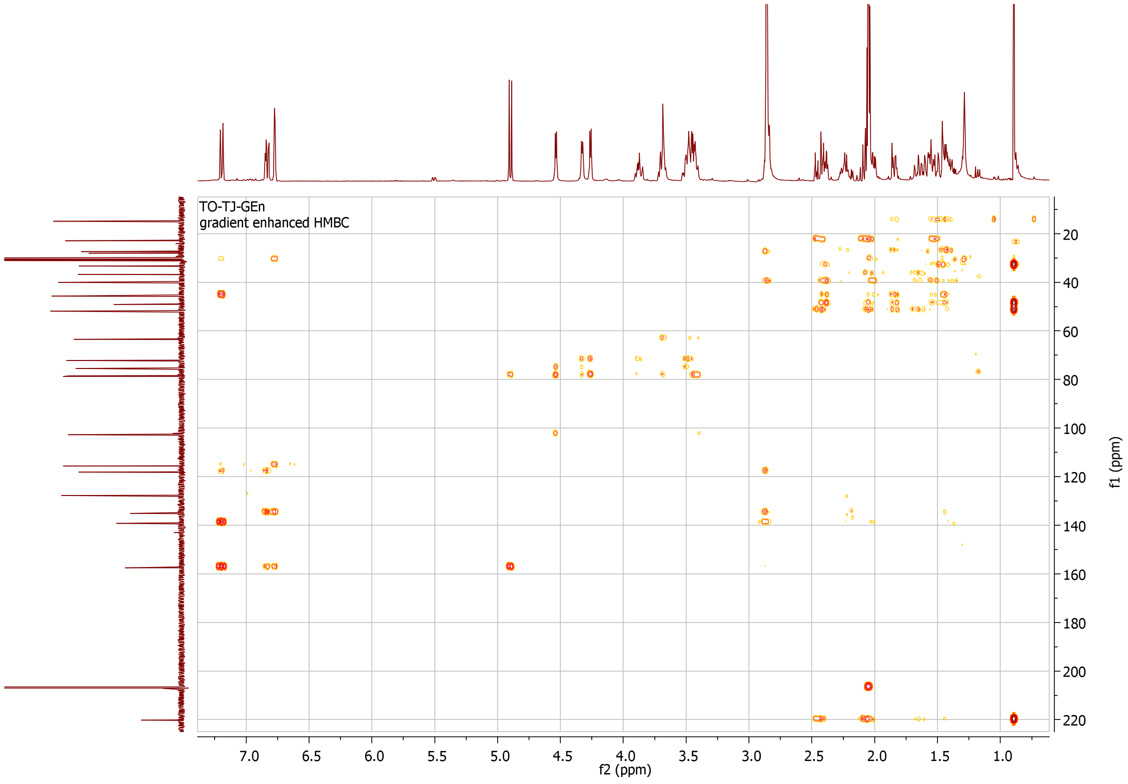

Figure S137. HMBC spectrum of 3-*O*-β-D-glucopyranosyl-estrone (Acetone-d_6, 600/151 MHz)
